# Gender-related factors affecting health seeking for neglected tropical diseases: findings from a qualitative study in Ethiopia

**DOI:** 10.1371/journal.pntd.0007840

**Published:** 2019-12-12

**Authors:** Alexandra Wharton-Smith, Christian Rassi, Esey Batisso, Giuseppina Ortu, Rebecca King, Misganu Endriyas, Helen Counihan, Prudence Hamade, Dawit Getachew

**Affiliations:** 1 Malaria Consortium, London, United Kingdom; 2 Malaria Consortium, Hawassa, Ethiopia; 3 The Nuffield Centre for International Health & Development, University of Leeds, Leeds, United Kingdom; 4 Regional Health Bureau, Hawassa, Ethiopia; 5 Malaria Consortium, Addis Ababa, Ethiopia; Liverpool School of Tropical Medicine, UNITED KINGDOM

## Abstract

**Background:**

Despite known gender-specific differences in terms of prevalence, transmission and exposure to neglected tropical diseases (NTDs), there is limited discussion of the influence of gender in NTD programmes and interventions. There is a paucity of research on how gender interacts with NTD service provision and uptake. This study, part of broader implementation research in Ethiopia, applied a gender lens to health seeking for five NTDs: lymphatic filariasis, podoconiosis, schistosomiasis, soil-transmitted helminth infection and trachoma.

**Methodology/principal findings:**

The study was conducted in a district of the Southern Nations, Nationalities, and Peoples' Region of Ethiopia where the five NTDs are prevalent. A qualitative methodology was adopted to explore participants’ perspectives and experiences. Data generation methods included 20 interviews and four focus group discussions. Community members, volunteer Health Development Army leaders, Health Extension Workers and a range of health workers at the health post, health centre and hospital level (n = 59) were purposively sampled. Interviews and focus group discussions were audio recorded, transcribed verbatim into English then analysed through open coding, drawing on constant comparative methods.

Gender related factors affected care seeking for NTDs and were described as reasons for not seeking care, delayed care seeking and treating NTDs with natural remedies. Women faced additional challenges in seeking health care due to gender inequalities and power dynamics in their domestic partnerships. Participants recommended raising community awareness about NTDs, however this remains problematic due to gender and social norms around appropriate discourse with members of the opposite gender.

**Conclusions/significance:**

The findings from this study provide crucial insights into how gender interacts with accessing health services, at different levels of the health system. If we are committed to leaving no one behind and achieving universal health coverage, it is essential to address gender disparities to access and utilisation of interventions delivered by national NTD programmes.

## Author summary

This study provides insights into how gender influences peoples’ access to health services for neglected tropical diseases (NTDs). There is considerable evidence to show that women and girls suffer a greater NTD burden due to their higher levels of poverty, lower education and social status. The psychological and social consequences of NTDs also tend to be worse for women. There is an urgent need to integrate NTDs into basic healthcare services, yet there is little evidence that can support a gender-based approach of how best to do so. This study addresses that gap by exploring how gender affects health seeking behaviour for NTDs in Ethiopia through a series of focus group discussions and interviews with community members and health service providers. The findings provide new insights into why women might not seek care or delay care seeking. In particular, there are reservations about disclosing diseases that affect “hidden” body parts, for example in the case of schistosomiasis, a parasitic infection that can affect the urogenital system. Men, on the other hand, may delay seeking care if they are affected by hydrocele, scrotal swelling that can be caused by lymphatic filariasis. There is therefore a need to adopt a gendered approach to NTD health service delivery.

## Introduction

There is limited discussion of the role of gender in neglected tropical disease (NTD) programmes, despite known gender-specific differences in terms of prevalence [1–3], transmission and exposure to NTDs [4]. These differences have been attributed to biological vulnerability and gender roles [5–8]. Often, women and girls experience a greater share of the NTD burden due to their disproportionate poverty, illiteracy, lower education and social status [9,10]. For example, in societies where women are the primary caregivers, they experience an increased risk of exposure and severe disease. A well-documented example is trachoma, where female caregivers come into more frequent contact with infected children than male caregivers and are thus more likely to become infected themselves [5,11]. In the case of podoconiosis, women have been found to be more at risk of acquiring the disease because they are less likely to own or wear shoes, reflecting cultural traditions and because available shoes were perceived to be more suitable for men [12,13]. Other studies have remarked on the underestimation and underreporting of NTDs in women, specifically schistosomiasis and lymphatic filariasis (LF), as diagnosis of these diseases can require women to comply with activities that are perceived as culturally inappropriate or taboo, such as providing a urine or stool sample or allowing intimate physical examination [14,15].

The psychological and social consequences of NTDs, which can be mutually reinforcing, especially of diseases that cause disfigurement, also tend to be worse for women [16]. Several studies of different NTDs found that males reported significantly lower levels of enacted and experienced stigma [17–19]. Other studies have illustrated how women’s experiences of physical impairment from NTDs exacerbate existing socio-cultural and economic inequalities [20–23]. Physical disfigurement is particularly detrimental for women, as their social acceptance and marriageability may rely on their physical appearance more than men’s [24,18,25], which can lead to social reclusiveness [25, 26]. Women are also at an increased risk of NTD-related mental distress and intimate partner violence (IPV) [27, 16, 28]. A recent study in Ethiopia found that podoconiosis in women increases the frequency and severity of IPV, including physical, psychological and financial violence and was attributed to “men’s frustration at being married to an ‘unhealthy’ woman who cannot always fulfil household and work expectations” [28]. Men, on the other hand, are uniquely affected by stigma associated with the disability of hydrocele, which has been linked with dissatisfaction with patients’ and partners’ sex lives, and, additionally, a belief that it causes impotence [24].

While gender-related differences in terms of NTD burden are well documented, there is a paucity of research on how gender interacts with NTD service provision and uptake. Of the limited literature available, studies have mainly explored access to mass drug administration (MDA) and how gender relates to the experience of being an NTD volunteer [29–36]. For example, that female NTD volunteers uniquely contend with the challenges of balancing work and family commitments [31]. In terms of MDA coverage, a study in Uganda found that men may miss out on treatment as they were more likely to be away from their homes due to occupational roles, whilst some Community Drug Distributors (CDDs) withheld drugs from pregnant or breastfeeding women due to drug safety concerns [29]. However, a recent study of MDA coverage in 16 countries suggested that NTD programmes “consistently achieve at least equal levels of coverage for women” [30]. Study findings on NTD volunteer performance, specifically, the effectiveness and coverage of MDA by female versus male NTD volunteers and recipient compliance, varied by setting and treatment [31–37].

Few studies have addressed gender and health care seeking for NTDs. This literature highlights gender inequalities in terms of delayed care seeking [16, 5], and coverage of treatment and services [18, 19, 38, 39]. The majority of studies have concluded that female gender is disadvantageous [40–42]. However, much of the research on gender and NTDs to date has neglected the experience of men [43,44].

There is a clear research need for in-depth, qualitative exploration of gender as a factor in health seeking for NTDs to inform health programme design and improve gender equity, especially in the context of the “leaving no one behind” agenda [45] and achieving universal health coverage (UHC) [46]. As we move beyond the 2020 NTD roadmap targets [47], there is a need to integrate NTDs into routine primary health care (PHC) services, yet little evidence exists on how this can be designed taking gender-specific health seeking behaviours into account.

This qualitative study aimed to address this gap by applying a gender lens to health seeking for five NTDs: lymphatic filariasis, podoconiosis, schistosomiasis (intestinal and urogenital), soil-transmitted helminth (STH) infections and trachoma. The data presented in this paper was collected as part of a broader implementation research study. The findings provide crucial insights on how gender interacts with accessing care for NTDs at different levels of the PHC system in Ethiopia.

## Methods

### Study design

In 2017 and 2018, we conducted the research in collaboration with the Ethiopian Federal Ministry of Health (FMoH) and the Regional Health Bureau (RHB) in the Southern Nations, Nationalities, and Peoples' Region (SNNPR). The broader study explored feasibility and acceptability of an intervention designed to strengthen detection, management and recording of the five target NTDs at the different levels of the PHC system in Ethiopia: hospital, health centre, health posts and the community volunteer Health Development Army (HDA). The intervention comprised the following components (see S2 Appendix for a more detailed description of the intervention, informed by the template for intervention description and replication (TIDieR) checklist and guide) [48]:

- Harmonised case definitions for the five target NTDs
- Definition of roles and responsibilities for the detection, management and recording of target NTDs at the different levels of the PHC system
- Training for health workers and supervisors on case definitions, roles and responsibilities and provision of practical job aids
- Training for HDA volunteers and provision of simple visual tools to detect community members with signs and symptoms of NTDs and encourage them to seek care

As part of a systematic evaluation of the intervention, health workers, HDA volunteers and community members were invited to participate in focus group discussions (FGDs) and key informant interviews (KIIs). The objectives of this study were to capture participants’ perceptions of the intervention, as well as their experience of NTDs and NTD care more generally. Broader evaluation findings have been published elsewhere [49]; this paper focuses on data relating to gender and health care seeking. The reporting of qualitative methods in this paper has been cross-checked with the consolidated criteria for reporting qualitative research (COREQ) [50].

### Study setting

The study was conducted in a rural *kebele* (the smallest administrative structure within a district) located in SNNPR, the third largest administrative region of Ethiopia and the country’s most diverse in terms of language and ethnicity. Most of the population reside in rural areas, where the main economic activity is agriculture. The language most widely spoken in the study area is Sidamigna, the language spoken by the Sidama people. All five target NTDs are endemic in the study area, which was selected in consultation with RHB and for operational reasons. Health centres were purposively sampled based on whether they were active at the time of data collection and are assumed to be representative of similar health centres in rural areas in Ethiopia. All of the Health Extension Workers (HEW) and HDA leaders included in the study area were female, in line with national policy. The following inclusion criteria were applied to determine health centres’ eligibility for inclusion in the study:

- Access to electricity (grid, solar or generator)
- Access to clean water and acceptable level of sanitation (e.g. presence of latrines and wells)
- Availability of rooms to perform simple laboratory analyses
- Reported cases of target NTDs in the recent past
- Not enrolled in other research studies
- Does not receive dedicated support from non-government organisations (NGO)
- Does not expect disruption to service delivery during intervention period

Of the six health centres in the study area, three were excluded based on the research team’s knowledge of their location and circumstances. The remaining three health centres were visited by the study in June 2017 to determine their eligibility. Only one health centre in the study area met all inclusion criteria and was therefore selected as the study site. It serves a population of approximately 21,000. The health centre is overseen by a General Hospital, which was also included in the study. There was one active and functional health post in the health centre’s catchment area at the time of data collection, which was staffed by Health Extension Workers (HEWs), a cadre of salaried, female community health workers. The health post supports a network of community-based female HDA volunteers.

All health workers at the health centre and hospital levels responsible for the detection, management and recording of NTDs in the study area were invited to participate in the study, along with all HEWs and HDA volunteers in the catchment area.

### Data collection

Study tools for FGDs and KIIs were developed and tailored to each strata of the sample (see S3 Appendix). Semi-structured topic guides were used to elicit data on the following themes:

- NTD management at the community, health post, health centre and hospital level
- Gender
- Stigma
- Perceptions of the different components of the intervention

All topic guides were developed in English by CR and EB, and subsequently translated into Amharic (the country’s official language) by a professional translator. During FGDs with HDA volunteers and community members, researchers translated verbally into Sidamigna, the local language commonly spoken in the study area. The topic guides could not be pre-tested. However, the transcripts were reviewed by CR at regular intervals and the topic guides subsequently refined based on emerging themes and gaps in the data.

Data collection was conducted between August and September 2018 by six field researchers (two female, four male) who were recruited for this exercise from local and regional health authorities, health teaching institutes and health consultancy firms. All except one had a master’s degree in public health or related field. All researchers had previously been involved in field research and, again with one exception, had some prior experience of conducting qualitative research. All researchers attended a three-day training conducted by CR, which introduced the study design and objectives, an overview of qualitative research principles and techniques, ethical considerations and opportunities to practice qualitative research skills. Field work procedures and topic guides were discussed in detail, and the accuracy of the translations into Amharic was checked. While in the field, the researchers worked in pairs: one conducted the KII or moderated the FGD, whilst the other assisted and recorded notes. The researchers had regular quality assurance meetings with CR during the fieldwork to refine interview techniques and discuss edits to the topic guides.

### Sampling of participants

#### Key informant interviews

Semi-structured interviews were used to explore themes in more depth [51] with health workers and laboratory staff in a private setting. This method minimised service disruption at participating health facilities, as FGDs would require several health workers to be away from their work station simultaneously. Conducting KIIs also offered the benefit of focussing on health workers’ unique duties, as these differed across occupational groups. Health staff at the health centre and hospital levels were identified in consultation with the acting facility in-charge and approached on the day of data collection based on their availability. Only health workers with responsibility for detecting, managing and recording NTDs were eligible, with separate additional sampling criteria for each level of PHC system, across specialties and departments. At the health centre level, this included:

- At least one health worker from each of the following sections: Paediatrics, Outpatient Department (OPD), *kebele*/Health Post Support
- At least two nurses
- One Health Officer
- Two laboratory technicians
- One Health Management Information System (HMIS) focal person

At the hospital level, the following were sampled:

- At least one representative from each of the following case teams/wards was sought: paediatric, under-five OPD, adult OPD, Triage and Medical
- Two representatives from each of the following cadres: Health Officer, nurse, doctor
- Two laboratory staff
- One HMIS focal person

At both levels, the team aimed to sample roughly equal numbers of female and male participants. Within those criteria, sampling was based on convenience. At health post level, only two HEWs were included in the study, and both were interviewed. In total, KIIs were conducted with 20 health workers and laboratory staff: 9 at the hospital level, 9 from health centres and 2 from the health post (Table 1).

**Table 1:** Interview sample by primary healthcare system level, cadre and sex.

Primary healthcare system level	Cadre	Sex
Hospital	Laboratory technician	Female
Hospital	Laboratory technician	Male
Hospital	Medical doctor, Triage	Male
Hospital	Nurse, Adult Outpatient Department	Male
Hospital	Health Officer, Triage	Male
Hospital	Health Officer, Triage	Female
Hospital	Medical Doctor, Paediatrics	Male
Hospital	Nurse, Triage	Female
Hospital	HMIS Focal Person	Male
Health Centre	Nurse, Kebele Health Post Support	Male
Health Centre	Health Officer, Outpatient Department	Female
Health Centre	Health Officer, Emergency and Kebele support	Male
Health Centre	HMIS Focal Person	Male
Health Centre	Midwife, Kebele/Health Post Support, Family Planning and Women Care	Female
Health Centre	Nurse, Kebele/Health Post Support	Male
Health Centre	Nurse, Paediatrics	Female
Health Centre	Laboratory technician	Male
Health Centre	Laboratory technician	Male
Health Post	Health Extension Worker	Female
Health Post	Health Extension Worker	Female

#### Focus group discussions

FGDs were conducted with community members and HDA volunteers. This included two FGDs with community members (one male group with 10 participants and one female group with 9 participants), and two FGDs with HDA volunteers (all female, with 10 participants in each). This type of data collection facilitated interaction between participants and the expression of norms and consensus among peers [52]. Additionally, FGDs were considered appropriate for these strata given the large number of potential participants in the study area.

Separate FGDs were conducted with female and male community members to encourage open discussion. FGDs with community members comprised of a culturally appropriate mix of participants who were considered influential in terms of community members’ health seeking behaviour. This included *kebele* leaders, religious leaders and representatives from women’s groups. Only HDA volunteers known to be active were recruited for the FGDs with this target group. The sample included HDA members from different villages in each FGD. All FGD participants were identified in consultation with HEWs and health centre staff.

One deviation from the sampling criteria was the accidental inclusion of three female HDA volunteers in the FGD with female community members. In this paper, participants from that FGD whose quotes are reported are accurately tagged as either “HDA” or “Community Member”.

### Data analysis

All KIIs and FGDs were audio recorded, translated and transcribed verbatim into English by the researchers who had conducted the KII or FGD immediately after data collection.

CR developed an initial coding frame based on close reading of the transcripts. The coding frame was discussed and agreed with RK based on close reading of a sample of five transcripts with different target groups. All transcripts were analysed by AWS using a modified thematic content analysis approach, which involved inductive identification of themes through open coding by close line-by-line interrogation, drawing on constant comparative methods [53]. Emerging themes were discussed with CR who also reviewed and commented on an early draft report which was written by AWS and later reviewed by EB, HC and PH. Data saturation was likely reached on the themes covered in the topic guides. Data extracts used in the report are tagged with the data source (KII or FGD), participant type (e.g. community member), and sex.

Note that the authors recognise the distinction between “sex” as a biologically determined classification based on chromosomal derived reproductive organs, and “gender” as an individual’s self-identity and self-representation, which translates into a social, cultural and legal status. However “sex”, specifically described as female or male, is the standard distinction used in the majority of the scientific literature to date. This traditional classification was also used by the data collectors and the participants in this study when discussing gender, which is reflected in the way gender is discussed in this paper.

### Ethics

Ethical approval for the study was granted by the Ethiopian Southern Nations Nationalities and Peoples Regional Health Bureau Research Ethical Review Committee (reference number PMB-19/22566) and the University of Leeds Faculty of Medicine and Health Research Office, School of Medicine Research Ethics Committee (reference number: MREC16-146). Informed written consent was given by all participants, who were adults (aged 18 or above). The data was anonymised prior to analysis to protect confidentiality.

## Results and discussion

### Local conceptualisation and gendering of NTDs

Understanding local beliefs and discourse on disease can shed light on how NTDs are conceptualised, classified and experienced *in situ*. Discussions with both community members, HDA leaders and HEWs revealed a low awareness of “neglected tropical diseases” in the manner that they are named and conceptualised in biomedicine or classified in public health. Community members sometimes described and named diseases in the local language with symptoms that were similar to those of the five NTDs targeted by the intervention. Participants generally discussed and categorised diseases in terms of the symptoms they observed or experienced and the body parts that were affected. Conceptualising diseases by body part meant that those involving the genitals or breasts were treated as gender-specific.

*“Daniichu Xibba* [literally “the disease of the elephant”]: *a disease that makes the legs swell for men and a disease that makes women have swollen breasts*.*” G17*, *Male Community Member*, *FGD*

“I heard that the disease that affects women’s wombs makes the women infertile. The disease is transmitted by a snail that is found in the water. When people come into contact with the water containing the snail and the snail enters the body through people’s skin, it causes men to urinate red urine and women to be infertile.” G17, Male Community Member, FGD

This local sorting of diseases into categories based on symptoms and affected body parts by community members contrasts with how the World Health Organisation and public health community groups diseases together according to different criteria from a global health perspective. Therefore, it is important to understand community health beliefs and engage in dialogue as part of NTD interventions.

When discussing the five target NTDs, participants also implicitly sorted these diseases into those which affected the genitals or “hidden” body parts (urogenital schistosomiasis, LF) from those that did not (trachoma, soil-transmitted helminth infection, intestinal schistosomiasis and podoconiosis). “Hidden” body parts tended to refer to genitals in both genders and breasts in women; body parts that may engage in sexual activity, which induced a social taboo. This distinction is important as the diseases which were perceived as affecting “private areas” were negotiated differently in terms of interactions with health services compared to diseases affecting other non-private, “visible” body parts due to this sexual, social taboo.

Participants explained that it is more socially acceptable to speak about diseases which affect body parts that are “visible” compared to diseases that affect body parts that are “hidden” or that are “sex organ[s].” It is noted that for a body part to be considered “hidden” participants were not necessarily referring to body parts that would conventionally be covered by clothing. The term was also used euphemistically to body parts that may play a role in sexual contact. Similarly, for “visible,” this was more used to mean socially acceptable to discuss openly, between genders, rather than a body part that is physically visible in public. For the diseases related to the genitals or urinary tract, a few participants suggested that sufferers may hide their disease or be afraid to discuss it even though the disease might be common in the community.

“If the disease is around the genital area, they are afraid of talking with men. And sometimes diseases around women’s womb are not easily identified because women hide the disease fearing their secret may be revealed.” P01, Female HEW, Interview

“You know, there are people who are affected by urinary diseases. A lot of people are affected by those diseases and some even had surgery. Even I am affected by this disease. You see, the disease very much exists in the community, but no one dares to speak frankly about it. People do not talk about it with others, especially when the disease is related to the genital area. They hide the disease from the people around them.” G12, Male Community Member, FGD

HEWs and HDA volunteers responsible for raising awareness about these diseases in the community lamented the challenge of asking community members about their “hidden” body parts or urination. The reluctance to discuss hidden body parts hinders open dialogue on NTDs

*G02*: *Those body parts are not seen*. *They are hidden*. *The question is*, *how can we ask them*?

*G03*: *This picture shows a man’s swollen sex organ*. *And you men*, *you keep that swollen thing inside your trousers*. *So how can we ask him*? *I think this will be difficult*.

*Facilitator*: *Have you tried asking people by showing this picture [shows picture in the pictorial tool]*?

*G04*: *It will be very difficult to ask people who have a swelling in that area*.

*G05*: *If a woman has a swollen breast*, *we can ask her about her pain during our coffee ceremonies*.

*G06*: *For example*, *if people have pain in the breast area*, *people may not understand…For those who have breast pain or pain in the uterus…it is difficult to ask those who have swollen sex organs*. *For the others*, *we can ask them what kind of pain they have because the breast area is visible and also the abdomen is visible*.

*Female HDA leaders*, *FGD*

HDA volunteers acknowledged the reluctance to speak about these diseases and emphasised the importance of public discussion to raise awareness and encourage disclosure.

*“When we teach about the disease in public to the community*, *those who hide their disease can disclose easily and get treatment for their diseases*. *People may hide this kind of disease but understanding about the disease can help us to disclose and make people aware of the diseases*.” *E07*, *Female HDA leader*, *FGD*

Both female and male participants explained that talking about genitals, even in the context of disease that affect these body parts is socially taboo and culturally unacceptable between women and men, even if they are community health workers. This rule applied to both genders. It was not deemed socially acceptable for men to bring the topic up with women, nor for women to discuss such diseases or body parts with men. An exception to this was that it was deemed acceptable to discuss any body part or disease affecting urination when speaking about children, in contrast to sexually mature adults, possibly because such body parts in adults are related to sexual contact.

Talking openly about diseases that affect hidden areas with members of the opposite sex, regardless of profession, challenged socio-cultural norms and for women, risked the social consequences of being perceived as “ill-mannered.”

“[After some hesitation] I saw a man with a swollen scrotum [all participants laughed]. At that time, we raised the question to the Health Extension Workers saying, ‘You know, it is difficult to disclose and discuss this kind of issue with a man.’ You know, in our culture, women do not dare to talk with a man even about usual business. So how can we teach and discuss this kind of issue? …culturally, we find it difficult to reach the men with such a problem, because we fear to talk to men.” E03, Female HDA leader, FGD

“You know, female Health Development Army leaders may find it challenging to teach and detect the diseases that affect the male sex organs. I am absolutely sure that they are even afraid of naming those diseases that the males have. For example, they will be afraid to name the disease of the scrotum because in our culture, if the female names this disease frankly, she will be considered an ill-mannered woman.” G17, Female Community Member, FGD

The reluctance to speak openly about hidden body parts and the NTDs that affect them has implications for raising awareness and care seeking, especially as all HDA volunteers are female. Because of how NTDs were conceptualised, and in some cases, gendered, discussing some NTDs at the community level as envisaged by our intervention is challenging.

HDA leaders stressed the importance of educating women on diseases that can affect their sexual and reproductive organs and promoting open discussion, despite cultural taboos, to encourage timely uptake of health services.

“If women are educated, they teach those women who have problems with their uterus to get better treatment. And also by learning all of this, we prevent disease and death among children and adults from neglected diseases. We also encourage those women who hide their problems to talk about their problems.” E18, Female HDA leader, FGD

Suggestions from participants on how to communicate information about NTDs affecting men included training male community volunteers; communicating health messages to men through their wives, although this may be problematic if the man suspects a sexually transmitted infection; or family members and through male networks in the community, and to involve men in training on NTDs. The acceptability and feasibility of these proposed interventions would need to be explored through further research.

“For people with swollen testicles, it would be better to train males to educate those men. I’m afraid that will not be practical for us. It would be better to educate some men together with us. It is important to identify people with this problem through them. Because they may hide their problems from us women—they may hide the swelling.” E18, Female HDA leader, FGD

“But the Health Extension Worker replied to us that a person with such a problem, you can approach the man through a family member of the person. For example, if you discuss the issue with the wife of that individual, that woman can directly talk to him and he can disclose his issue.” E03, Female HDA leader, FGD

Hiding the disfigurement caused by NTDs was not limited to diseases affecting the breasts and genitals due to sexual connotations, which induced a social taboo; female community members also shielded other affected body parts from public view, as the disfigurement was perceived as a separate, additional, cultural taboo. This suggests that any swollen body part, for women at least, may become a “hidden” area because of the stigma associated with NTDs, whether related to sex or disfigurement, and may hinder detection of cases and care seeking.

*E17*: *There is one person who has a swollen scrotum in my village*, *but he always hides it*.

*E15*: *In my village people also have eye problems and there is an old woman with leg swelling*. *There’s another lady with a swollen leg*, *but she hides it under long dresses*, *so people don’t see it as it is seen as a taboo*.*” Female HDA leaders*, *FGD*

### Gender and health care seeking for NTDs

Gender as it related to health care seeking was generally discussed along two themes: 1) shame and fear of disclosing certain NTD symptoms and 2) power dynamics.

#### Shame and fear of disclosing symptoms

People discussed a number of gender related factors as challenges to care seeking. In some cases, these were discussed as deterring people from seeking care at all; in other cases, they were seen as barriers to timely care seeking. Women found it more challenging to disclose swellings of the limbs due to shame and social taboos. This finding resonates with the literature on NTD-related disfigurement, stigma and lowered social status for women compared to men [42]. The stigma around hidden body parts poses challenges to HDA leaders’ and HEWs’ work in identifying and discussing symptoms that affect private body parts, particularly in discussions between female community health workers and male community members. A few people mentioned natural remedies (fruit, vegetables and tree leaves) as preferable to seeking care at a health facility, specifically for a disease affecting the female breast that was perceived as difficult to discuss. The data on preferring natural remedies to accessing biomedical health services, possibly from health workers of a different gender, were not elaborated on but are significant, as this might be the preferred treatment for both men and women who wish to avoid seeking health care from someone of a different gender for sensitive health problems. For women, due to their lower socio-economic status, financial control and power dynamics in the study setting, as referred to by other participants, these inequalities may be reinforced by stigmatising and sexualised diseases, which could further deter their engagement with the formal health system.

“There is a disease that affects women’s breast…Culturally, it is treated by making the affected woman chew certain leaves of a tree, which make the disease disappear. This is also a disease that is not talked about much, but that affects our women.” G12, Male Community Member, FGD

Mirroring the comments from community health workers on the difficulties of discussing culturally sensitive symptoms with community members, on the theme of “hiding” disease, several participants explained how the reluctance to reveal or disclose deters men from accessing care from the exclusively female HDA volunteers.

“*I think people are afraid and they can only talk about the disease when they are at the hospital*. *So I’m worried that if the person affected by those diseases is a man*, *they will not tell the* [female] *Health Development Army member and she won’t be able to identify those cases easily*.*” G12*, *Male Community Member*, *FGD*

The social norm of not discussing genital symptoms with the opposite sex was repeatedly cited as a challenge to NTD service utilisation; care seeking for NTDs that affect the genitals from health workers of the opposite gender was considered problematic for all genders, though did not apply to children. Within the health facility setting, this social norm persisted, with women and men expressing a preference to interact with health workers of the same gender. These findings resonate with studies conducted in Ethiopia and other countries which found that women prefer interacting with female health professionals rather than males for “intimate care” in both community and health facility settings [54–59]. Women reported experiences of discomfort, fear and shame in situations where the patient was seen by a health worker of the opposite gender for diseases affecting the genitalia. When a disease manifested in the body part of an adult that was related to sexual activity, it was more difficult to talk about between genders due to the sexual connotations of that body part compared to diseases that do not, such as those which affect the gastrointestinal tract, for example. This resulted in stigma related to a sexual taboo.

“*Most patients prefer female health workers for female diseases and male health workers for male related diseases*.*” C02*, *Male Nurse*, *Kebele Health Post Support*, *Interview*

“If the disease is around the genital area, they [women] are afraid of talking with men.” P01, Female HEW, Interview

“The patient was female and the doctor was male. She was ashamed, tried to cover herself with a cloth, and she was talking more with me than with the doctor.” B06, Female Nurse, Hospital, Interview

Health workers themselves commented that gender does not affect how they treat patients.

“Gender issues, yes—sometimes it is difficult for female patients to talk to male health workers, but the main thing is that the health worker’s attitude in handling those cases.” C19, Female Midwife, Kebele and Health Post Support, Interview

One recommendation that emerged from a female health worker was to establish separate consultation areas and offer patients the option to request a health professional of the same gender for diseases affecting the breasts or genitals, such as NTDs and sexually transmitted infections, with the acknowledgement that this would be challenging at the hospital level due to predominance of male doctors and the heavy workload. The majority of comments on this theme suggests that the gender norms of socially acceptable talk transcend community settings and persist in the clinic. This was expressed through repeated statements on the preference for a health worker of the same sex for both genders, from the community to the hospital level. Women’s perceived shame and fear of receiving treatment from a male clinician may also deter women from seeking services from health facilities where the doctors are predominantly male. This finding is particularly important to consider when offering services at sites with all female community health workers, also when training health workers on patient rights, such as same-sex chaperones and sensitivities around managing ‘taboo’ diseases in health facilities in patients of the opposite gender.

#### Power dynamics

People generally agreed that women seek care later than men or not at all, in the context of unequal gender relations. The examples cited included the power dynamics between women and men, such as: requiring a husband’s permission to disclose their disease and seek care; economic dependency on men to pay for health services; and the threat of violence if women disclose symptoms, especially for socially stigmatising conditions that affect the genitals or cause disfigurement.

**“***Even if I don’t have evidence to say that*, *but females are not coming to health facility*. *For example*, *trichiasis is common in females*. *Females are more exposed in my experience*, *but they only come when the case is complicated and they may not come at all because of economic related issues*.*” B37*, *Male Paediatrician*, *Hospital*, *Interview*

“Males and females are not equally faced with the diseases. The women need permission to get care from her husband, because the females are influenced by males at home whether or not to disclose the disease. Fear and stigma is there. They have no money to go for treatment and come to the health centre on their own. I have seen cases who feared to come here to the health centre with signs and symptoms in the genital area, so the gender issue was big here, yes.” C02, Male Nurse, Kebele Health Post Support, Interview

**“***I think male cases are treated earlier than females because most of the time*, *females tolerating even if they have a lot of diseases*, *especially in the rural area*. *This may be due to gender violence in my opinion*. *I have seen that of those that come to get services*, *almost all are male*.*” B34*, *Female Health Officer*, *Triage*, *Hospital*, *Interview*

Although the economic costs of NTDs, such as loss of earnings due to disability, have been documented [60,61] there is a lack of evidence on how this may impact different genders and factor into relational gender dynamics and health seeking behaviours.

### Conclusion

Diseases may be conceptualised and gendered according to local nosologies, with important implications for health seeking practices in rural communities. Gender-related factors affect care seeking for NTDs and are sometimes described as reasons for not seeking care at all, delayed care seeking or treating NTDs with natural remedies. Moreover, health workers reiterated that women face additional challenges in seeking care due to power dynamics and access services at later stages of disease when complications are likely to be more severe. The findings echo previous studies which found that women may experience a greater NTD burden, with both social and physical consequences [9]. Also, that gender plays a role in access to financial resources, in the case of women negatively and the uptake and timeliness of care seeking [5,16], as identified in other studies [18,19, 38, 39]. Participants recommended raising community awareness about NTDs, however this approach would need to consider the gender and social norms around appropriate talk in order to be socially acceptable. Participants recommended training men to be HDA leaders to overcome this challenge, which would also support community level care for male patients with hydrocele.

Contributing to the limited literature on gender and equity in NTD research, this study provides insights on the important influence of gender in health care seeking for NTDs in local contexts. The results demonstrate that gender is significant in health seeking for NTDs, particularly those which cause genitourinary tract symptoms and are perceived as socially taboo to discuss. These findings can be useful for informing programme design towards ensuring the promotion of equitable implementation. Yet there remains a need for in-depth, qualitative exploration of gender, how this intersects with or reinforces other social determinants, and health seeking for NTD care in different socio-economic and cultural settings. In future studies and discourse, the public health research community could progress how gender is conceptualised and discussed, for example moving away from limited descriptions of gender as binary to encompass the range of gender identities. NTD programmes which neglect gender considerations in their design and implementation risk exacerbating gender and health and health system, social and structural inequalities [44]. Those which adopt a gendered approach to NTD health service delivery can enhance acceptability and uptake, in line with the provision of universal health coverage.

### Study limitations

A number of limitations of this study should be noted. Some of the data collectors had limited experience of conducting qualitative interviews and writing in English. Also, participant age was not considered in the selection criteria. No efforts were made to recruit marginalised community members such as adolescents, the elderly, or people who are stigmatised, therefore their views may not be represented by the findings. Due to limitations in terms of time and budget and as the tools could only be used meaningfully with participants who had been exposed to the small-scale intervention, it was also not possible to pre-test the topic guides. However, quality assurance was provided by a senior member of the study team throughout the field work and data were collected over the course of three iterative rounds, with improvements and adaptations made to the tools throughout the process. As the evaluation was focussed on the broader implementation of the programme, the topic guides did not include open-ended questions on the theme of equity; specifically in terms of barriers to service uptake. Lastly, the results represent the local area where the study was conducted, therefore the findings are limited in their transferability and generalisability to other contexts.

Based on our findings, we have identified the following implications for policy, practice and research:

- Consider grouping NTDs by symptoms (including similar non-NTDs where appropriate) to better accommodate how communities conceptualise the diseases.
- Include gender issues in health worker and community health worker training.
- At the national policy and local levels, promote gender balance in the selection of health workers for all NTD related training programmes, particularly HDA volunteers and Health Extension Workers.
- Design gendered health outreach activities that follow culturally appropriate norms, for example employing community health workers of different genders to lead sessions on sensitive health topics and diseases.
- Utilise safe, private and appropriate times and locations for knowledge sharing, such as coffee mornings, in homes, with neighbours, people who are well known, within existing social networks, to encourage people to share their own experiences with disease, if this is socially acceptable, as determined by further research.
- Mobilise male NTD champions targeting hydrocele and urinary tract symptoms in men.
- Promote the de-stigmatisation of NTDs in the community and public discourse.
- Promote timely uptake of health services among women with a focus on community dialogues about gender inequalities
- Provide NTD services from health workers of any gender when possible, based on the patient’s preference.
- At the health facility level and above, provide gender appropriate areas for examination and collection of sample.
- Provide NTD services in a welcoming, safe environment at health facilities.
- Conduct a larger study which represents the country's diverse regions to inform national policy and practice on gender mainstreaming within national NTD programmes.
- Conduct further ethnographic research on gender, gender relations between community member and health workers, intersectionality and NTD services.
- Future research could explore local and contextual conceptualisations of gender. The authors encourage a shift away from narrow, binary definitions of gender to encompass all gender expressions in public health research and programming.

## Supporting information

S1 Appendix STROBE checklist. (DOC)

S2 Appendix Intervention description. The description of the intervention is informed by the template for intervention description and replication (TIDieR) checklist and guide.
(PDF)

S3 Appendix Topic guides. (PDF)

S4 Appendix Raw data. (PDF)
