# Supplementary material for: Gender-related factors affecting health seeking for neglected tropical diseases: findings from a qualitative study in Ethiopia
Source: PLoS Negl Trop Dis. 2019 Dec 12;13(12):e0007840. doi: 10.1371/journal.pntd.0007840 (PMC6907747; doi:10.1371/journal.pntd.0007840)
Supplement: S1 Appendix — (DOC) [file pntd.0007840.s001.doc]

STROBE Statement—Checklist of items that should be included in reports of ***cross-sectional studies***

|  | Item No | Recommendation |
| --- | --- | --- |
| **Title and abstract** | 1 | 1. Indicate the study’s design with a commonly used term in the title or the abstract   Title indicates that results from a cross-sectional qualitative study are reported |
|  |  | 1. Provide in the abstract an informative and balanced summary of what was done and what was found  \| Abstract reports key information about the methodology used and summary of main results \| \| --- \| |
| Introduction | | |
| Background/rationale | 2 | Explain the scientific background and rationale for the investigation being reported  Introduction, paragraphs 1-6 |
| Objectives | 3 | State specific objectives, including any prespecified hypotheses  Introduction, paragraph 6 |
| Methods | | |
| Study design | 4 | Present key elements of study design early in the paper  Introduction, paragraph 6  Methods, paragraphs 1-2 |
| Setting | 5 | Describe the setting, locations, and relevant dates, including periods of recruitment, exposure, follow-up, and data collection  Methods, study setting, paragraphs 1-3  Methods, data collection, paragraphs 1-3 |
| Participants | 6 | 1. Give the eligibility criteria, and the sources and methods of selection of participants   Methods, study setting, paragraphs 1-3  Methods, sampling of participants, paragraphs 1-6 |
| Variables | 7 | Clearly define all outcomes, exposures, predictors, potential confounders, and effect modifiers. Give diagnostic criteria, if applicable  Methods, sampling of participants, paragraphs 1-6, data analysis, paragraph 1 |
| Data sources/ measurement | 8* | For each variable of interest, give sources of data and details of methods of assessment (measurement). Describe comparability of assessment methods if there is more than one group  Methods, data collection, paragraphs 1-2, sampling of participants, paragraphs 1-6, data analysis, paragraph 1 |
| Bias | 9 | Describe any efforts to address potential sources of bias  Methods, sampling of participants, paragraphs 1-6 |
| Study size | 10 | Explain how the study size was arrived at  Methods, sampling of participants, paragraph 2 |
| Quantitative variables | 11 | Explain how quantitative variables were handled in the analyses. If applicable, describe which groupings were chosen and why  Not applicable |
| Statistical methods | 12 | 1. Describe all statistical methods, including those used to control for confounding   Not applicable |
|  |  | 1. Describe any methods used to examine subgroups and interactions   Not applicable |
|  |  | 1. Explain how missing data were addressed   Not applicable |
|  |  | 1. If applicable, describe analytical methods taking account of sampling strategy   Not applicable |
|  |  | 1. Describe any sensitivity analyses   Not applicable |
| Results | | |
| Participants | 13* | 1. Report numbers of individuals at each stage of study—eg numbers potentially eligible, examined for eligibility, confirmed eligible, included in the study, completing follow-up, and analysed   Methods, sampling of participants, paragraphs 1-6 |
|  |  | 1. Give reasons for non-participation at each stage   Methods, study setting, paragraph 3 |
|  |  | 1. Consider use of a flow diagram   Not applicable |
| Descriptive data | 14* | 1. Give characteristics of study participants (eg demographic, clinical, social) and information on exposures and potential confounders   Methods, study setting, paragraphs 1-3  Methods, sampling of participants, paragraphs 1-6 |
|  |  | 1. Indicate number of participants with missing data for each variable of interest   Methods, sampling of participants, paragraphs 1-6 |
| Outcome data | 15* | Report numbers of outcome events or summary measures  Not applicable |
| Main results | 16 | 1. Give unadjusted estimates and, if applicable, confounder-adjusted estimates and their precision (eg, 95% confidence interval). Make clear which confounders were adjusted for and why they were included   Not applicable |
|  |  | 1. Report category boundaries when continuous variables were categorized   Not applicable |
|  |  | 1. If relevant, consider translating estimates of relative risk into absolute risk for a meaningful time period   Not applicable |
| Other analyses | 17 | Report other analyses done—eg analyses of subgroups and interactions, and sensitivity analyses  Qualitative analysis: Results and Discussion, Lines 301-537 |
| Discussion | | |
| Key results | 18 | Summarise key results with reference to study objectives  Results and Discussion, Lines 301-537  Conclusion, paragraphs 1-2 |
| Limitations | 19 | Discuss limitations of the study, taking into account sources of potential bias or imprecision. Discuss both direction and magnitude of any potential bias  Conclusion, paragraph 3 |
| Interpretation | 20 | Give a cautious overall interpretation of results considering objectives, limitations, multiplicity of analyses, results from similar studies, and other relevant evidence  Results and Discussion, Lines 301-537  Conclusion |
| Generalisability | 21 | Discuss the generalisability (external validity) of the study results  Results and Discussion, Lines 301-537  Conclusion |
| Other information | | |
| Funding | 22 | Give the source of funding and the role of the funders for the present study and, if applicable, for the original study on which the present article is based  Information about funding has been provided |

*Give information separately for exposed and unexposed groups.

**Note:** An Explanation and Elaboration article discusses each checklist item and gives methodological background and published examples of transparent reporting. The STROBE checklist is best used in conjunction with this article (freely available on the Web sites of PLoS Medicine at http://www.plosmedicine.org/, Annals of Internal Medicine at http://www.annals.org/, and Epidemiology at http://www.epidem.com/). Information on the STROBE Initiative is available at www.strobe-statement.org.
