# Supplementary material for: Gender-related factors affecting health seeking for neglected tropical diseases: findings from a qualitative study in Ethiopia
Source: PLoS Negl Trop Dis. 2019 Dec 12;13(12):e0007840. doi: 10.1371/journal.pntd.0007840 (PMC6907747; doi:10.1371/journal.pntd.0007840)
Supplement: S2 Appendix — The description of the intervention is informed by the template for intervention description and replication (TIDieR) checklist and guide. (PDF) [file pntd.0007840.s002.pdf]

TIDieR intervention description

**NAME**	Strengthening primary healthcare for five neglected tropical diseases
**WHY**	Global efforts to reduce the burden of neglected tropical diseases (NTDs) have largely focused on developing and distributing safe and effective drugs to at-risk populations, often through mass drug administration (MDA). While MDA remains a cost-effective prevention and control strategy, there is growing recognition that alternative and complementary tools and approaches are needed to advance the NTD agenda, especially for the many NTDs that are not treatable through MDA. There is also an urgent need to address the acute and chronic suffering, including long-term disabilities, associated with many NTDs. A cornerstone of the post-2020 NTD agenda is therefore to ensure that health services meet the needs of those living with NTDs. This intervention aimed to enable health workers to detect, manage and record NTDs, defining appropriate roles and responsibilities at the different levels of Ethiopia's primary healthcare (PHC) system: hospital, health centre, Health Extension Workers (HEWs) and the Health Development Army (HDA). Five NTDs endemic in the intervention area were targeted: trachoma, schistosomiasis, soil-transmitted helminth infections, podoconiosis and lymphatic filariasis.
**WHAT - MATERIALS**	• Case definitions, appropriate to the different levels of the PHC for the five target NTDs<br>• A health worker training manual<br>• Simple visual representations of key signs and symptoms of NTDs for use by the HDA in detecting suspected cases at community level, designed to integrate with an existing Family Health booklet<br>• Practical job aids for health personnel at hospital, health centre and health posts, designed to guide health workers through the detection, management and recording of NTDs, linking to existing guidelines (e.g. Integrated Community Case Management) and reference documents (e.g. WHO trachoma grading card) where appropriate<br>• Copies of relevant existing guidelines and reference documents<br>• Drugs and equipment not routinely available at PHC level but required to fulfil the roles and responsibilities introduced for this intervention, e.g. equipment for clinical examinations and laboratory equipment
**WHAT - PROCEDURES**	• Four-day training of trainers<br>• 2-day training for health workers (hospital, health centre, HEWs) and their supervisors (regional and local health authorities)<br>• 1-day training for laboratory staff (health centre)<br>• Training of HEWs included module on cascading information to the HDA
**WHO PROVIDED**	• Case definitions, training manual, visual tool for the HDA and practical job aids for health workers developed by Malaria Consortium, led by an NTD Specialist and with input from technical experts and Ethiopia's Federal Ministry of Health.

	• Visual tool for the HDA and practical job aids for health workers pre-tested with prospective users<br>• Training-of-trainers delivered by master trainers comprising Malaria Consortium's NTD Specialist and Capacity Building Specialist, as well as national NTD experts<br>• Health worker trainings delivered by trainers recruited from regional health authorities and senior health workers from participating PHC facilities<br>• Laboratory staff training delivered by a regional expert<br>• HEWs cascaded information to the HDA<br>• Drugs and equipment distributed by Malaria Consortium
**HOW**	• Training-of-trainers and health worker trainings delivered as classroom trainings, including presentations, group work and individual exercises<br>• Laboratory staff training delivered as classroom training, including practical exercises<br>• HDA briefed about the intervention by HEWs as part of their routine interactions<br>• Copies of the visual tool for the HDA provided to HEWs during the health worker training for further distribution to HDA<br>• Copies of practical job aids distributed to training participants<br>• Additional copies of practical job aids distributed to participating health facilities for distribution among health workers who did not attend the training<br>• Copies of relevant existing guidelines and reference documents distributed to participating health facilities<br>• Drugs and equipment distributed to participating PHC facilities
**WHERE**	The intervention was implemented in the catchment area of one health centre in a rural *kebele* (district) in Hawela Tula sub-city. This included one health post overseen by the health centre and its HDA network, as well as the hospital to which the health centre reports. Hawela Tula is located in Ethiopia's Southern Nations, Nationalities, and Peoples Region (SNNPR).
**WHEN AND HOW MUCH**	• Training-of-trainers (April 2018): 1 four-day training; 6 trainers trained<br>• Health worker training (May 2018): 5 two-day trainings comprising between 16 and 25 participants; total of 97 health workers (approx. 50% of the health workforce at participating PHC facilities) and 2 regional supervisors trained<br>• Laboratory training (August 218): 1 one-day training; 2 laboratory staff trained (approx. 30% of laboratory staff at the participating health centre)<br>• Information cascade to HDA (June-September 2018): 180 HDA members (approx. 97% of the HDA network in the catchment area) trained as part of routine interactions between HEWs and HAD<br>• Visual tool provided to each HDA member in the catchment area by HEWs as part of the information cascade (June - September 2018)<br>• Practical job aids distributed to all training participants during the health worker training (May 2018)

	• Additional copies of practical job aids distributed to health facilities for further distribution among health workers who did not attend the training based on known numbers of health workers at participating PHC facilities (June-July 2018)<br>• Relevant existing guidelines and reference documents distributed to participating health facilities distributed to participating PHC facilities based on known number of wards/case teams/consultation rooms (June-July 2018)<br>• Drugs and equipment distributed to participating PHC facilities based on estimated patient numbers and disease prevalence (June-July 2018)
