# Supplementary material for: Gender-related factors affecting health seeking for neglected tropical diseases: findings from a qualitative study in Ethiopia
Source: PLoS Negl Trop Dis. 2019 Dec 12;13(12):e0007840. doi: 10.1371/journal.pntd.0007840 (PMC6907747; doi:10.1371/journal.pntd.0007840)
Supplement: S3 Appendix — (PDF) [file pntd.0007840.s003.pdf]

S3 Appendix. Topic guides: interview and focus group discussion

Key Informant Interviews – data management (hospital)

Interview details

**Interview number**				
**Interviewee code**				
**Sex**		Female		Male
**Attended classroom training**		Yes		No
**Job title**				
**Name of interviewer**				
**Name of notetaker**				
**Date**				
**Time started**				
**Time finished**				
**Venue**				

Consent

Before starting the interview, informed written consent from the participant must be obtained. Please follow the script provided:

Good morning/ good afternoon

My name is [*name*] and this is my colleague [*names*]. We work for Malaria Consortium, a not-for-profit organisation that specialises in the prevention, control and treatment of malaria and other diseases. In collaboration with the Federal Ministry of Health in Ethiopia, we are currently carrying out a study to explore ways of improving care for a group of infectious diseases called 'neglected tropical diseases' or 'NTDs'. We are testing an intervention that we hope will improve health workers' and Health Extension Workers' ability to detect, manage, record and report those diseases. The intervention has been implmented at your health facility over the last few months and we would like to get your feedback on some aspects of the intervention. Your responses will help us to evaluate the intervention, find out what worked and what didn't and improve the intervention in the future.

All the information you provide will be strictly confidential and your replies will only be shared with the other researchers working on this study. You will be identified by a number, not your name. We have put together an information sheet which summarises the purpose of the interview, why you've been selected and what happens if you agree to participate. Please take a moment to read through the information provided on the sheet.

Do you have any questions about the information provided on this sheet?

Remember that you do not have to take part in the interview if you prefer not to. You do not have to answer questions you don't feel comfortable with and can end the interview at any time. If you agree to participate, we will ask you to sign a consent form. We will audio-record the interview and take notes. This will help us remember what you are telling us. We will destroy the audio-recording once the report for this project has been written. Can I please ask you to read this consent form?

Do you have any questions about the information in this form?

If you consent to participating in the interview, can I please ask you to sign the form?

Thank you very much for agreeing to participate in the interview.

Topic guide

A Training

1. **In May, training was offered to health workers from your hospital about nelected tropical diseases. Did you attend this training?**
2. *[If training attended]* **Can you tell me what you learned in this training?**
    - What was the most interesting thing you learned at the training?
    - Do you remember which diseases this training covered?
    - What did you like about the training?
    - Was there anything you didn't like about the training?
    - How could the training have been improved?
    - Did you think the training was relevant for your job? Why or why not?
    - After the training, what is your understanding of your role with regard to recording and reporting neglected tropical diseases?
3. *[If training not attended]* **Did anyone tell you about this training?**
    - What did others tell you about this training?
    - Do you know what the training was about? Which tests were covered and which diseases are detected with those tests?
    - Do you think the training might have been relevant for your job? Why or why not?
    - Were you told what, according to this training, laboratory staff's role would be with regard to recording and reporting neglected tropical diseases?

C Recording and reporting

4. **The training covered six diseases: trachoma, urinary schistosomiasis, intestinal schistosomiasis, soil-transmitted helminth infections, lymphatic filariasis and podoconiosis. Do you ever record and report any of those diseases?**
    - *[For the diseases the interviewee records/reports]* How often does this happen?
    - How exactly do you record and report those diseases?
    - *[For the diseases the interviewee does not record/report]* Why do you think this is the case?
    - How would patients with those diseases be recorded/reported?
5. **The intervention we are testing suggests that trachoma cases should be recorded as "confirmed trachoma" if diagnosed based on a list of signs and symptoms. In your experience, does this currently happen at this hospital?**
    - If no, why not?

- In your opinion, are there any problems with recording trachoma cases as suggested by the intervention?
- How could the problems you mentioned be addressed? What alternatives or improvements would you suggest?

6. **The intervention suggests that urinary schistosomiasis cases should be recorded as "confirmed urinary schistosomiasis" if confirmed by a urinary sedimentation test or "suspected urinary schistosomiasis" if the disease is suspected but not confirmed through a laboratory test. In your experience, does this currently happen at this hospital?**
    - If no, why not?
    - In your opinion, are there any problems with recording urinary schistosomiasis cases as suggested by the intervention?
    - How could the problems you mentioned be addressed? What alternatives or improvements would you suggest?
7. **The intervention suggests that intestinal schistosomiasis cases should be recorded as "confirmed intestinal schistosomiasis" if confirmed by a CCA or stool concentration test or "suspected helminthiasis" if the disease is suspected but not confirmed through a laboratory test. In your experience, does this currently happen at this hospital?**
    - If no, why not?
    - In your opinion, are there any problems with recording intestinal schistosomiasis cases as suggested by the intervention?
    - How could the problems you mentioned be addressed? What alternatives or improvements would you suggest?
8. **The intervention suggests that soil-transmitted helminth infection cases should be recorded as "confirmed soil-transmitted helminth infections" if confirmed by a stool concentration test or "suspected helminthiasis" if the disease is suspected but not confirmed through a laboratory test. In your experience, does this currently happen at this hospital?**
    - If no, why not?
    - In your opinion, are there any problems with recording urinary schistosomiasis cases as suggested by the intervention?
    - How could the problems you mentioned be addressed? What alternatives or improvements would you suggest?
9. **The intervention we are testing suggests that lymphatic filariasis cases should be recorded as "morbidity related to lymphatic filariasis" if diagnosed based on a list of signs and symptoms. In your experience, does this currently happen at this hospital?**
    - If no, why not?
    - In your opinion, are there any problems with recording trachoma cases as suggested by the intervention?

- How could the problems you mentioned be addressed? What alternatives or improvements would you suggest?

10. The intervention we are testing suggests that podoconiosis cases should be recorded as "morbidity related to podoconiosis" if diagnosed based on a list of signs and symptoms. In your experience, does this currently happen at this hospital?

- If no, why not?
- In your opinion, are there any problems with recording trachoma cases as suggested by the intervention?
- How could the problems you mentioned be addressed? What alternatives or improvements would you suggest?

C Overall perception of the intervention

11. What do you generally think of tasking data management staff at hospital level with recording and reporting neglected tropical diseases?

- In your opinion, what are the implications of performing this task in terms of time and workload?
- Do you think the data recorded and reported will be reliable?
- How could the problems you mentioned be addressed? What alternatives or improvements would you suggest?

12. And more generally, what do you think about the idea of tasking health workers at primary health care level (including health posts, health centres and hospitals) with detecting, managing, recording and reporting neglected tropical diseases?

- Do you think the primary health care system has the capacity to take on these roles and responsibilities?
- What kind of challenges do you foresee?
- Do you think there would be any challenges with regard to gender? Would women and men face different challenges in accessing care for NTDs at the hospital?
- Do you think there would be any challenges with regard to stigma? Would those affected by disabilities caused by NTDs access care at the hospital?
- What could be done to address those challenges?
- What would your recommendations with regard to integrating the detection, management, recording and reporting of NTDs into primary health care?

13. Is there anything else you would like to share with the research team?

This concludes the interview. Many thanks for taking the time to participate.

Key Informant Interviews – health workers (hospital)

Interview details

**Interview number**				
**Interviewee code**				
**Sex**		Female		Male
**Attended classroom training?**		Yes		No
**Case team/ward**		Paediatrics		
		Under-five OPD		
		Adult OPD		
		Triage and Medical		
		Other (specify:)		
**Cadre**		Doctor		
		Nurse		
		Health Officer		
		Other (specify:)		
**Job title**				
**Name of interviewer**				
**Name of notetaker**				
**Date**				
**Time started**				
**Time finished**				
**Venue**				

Consent

Good morning/ good afternoon

My name is [*name*] and this is my colleague [*names*]. We work for Malaria Consortium, a not-for-profit organisation that specialises in the prevention, control and treatment of malaria and other diseases. In collaboration with the Federal Ministry of Health in Ethiopia, we are currently carrying out a study to explore ways of improving care for a group of infectious diseases called 'neglected tropical diseases' or 'NTDs'. We are testing an intervention that we hope will improve health workers' and Health Extension Workers' ability to detect, manage, record and report those diseases. The intervention has been implmented at your health facility over the last few months and we would like to get your feedback on some aspects of the intervention. Your responses will help us to evaluate the intervention, find out what worked and what didn't and improve the intervention in the future.

All the information you provide will be strictly confidential and your replies will only be shared with the other researchers working on this study. You will be identified by a number, not your name. We have put together an information sheet which summarises the purpose of the interview, why you've been selected and what happens if you agree to participate. Please take a moment to read through the information provided on the sheet.

Do you have any questions about the information provided on this sheet?

Remember that you do not have to take part in the interview if you prefer not to. You do not have to answer questions you don't feel comfortable with and can end the interview at any time. If you agree to participate, we will ask you to sign a consent form. We will audio-record the interview and take notes. This will help us remember what you are telling us. We will destroy the audio-recording once the report for this project has been written. Can I please ask you to read this consent form?

Do you have any questions about the information in this form?

If you consent to participating in the interview, can I please ask you to sign the form?

Thank you very much for agreeing to participate in the interview.

Topic guide

A Training

14. In May this year, classroom training was offered to health workers from your hospital about nelected tropical diseases. Did you attend this training?

15. *[If training attended]* **Can you tell me what you learned in this training?**

- Do you remember which diseases this training covered?
- What did you like about the training?
- Was there anything you didn't like about the training?
- How could the training have been improved?
- Did you think the training was relevant for your job? Why or why not?

16. *[If training not attended]* **Did anyone tell you about this training?**

- What did others tell you about this training?
- Do you know what the training was about? Which diseases were covered?
- Do you think the training might have been relevant for your job? Why or why not?

17. **What is your understanding of your role with regard to detecting and managing NTDs?**

- Is recognising the signs and symptoms of NTDs part of your role?
- Is diagnosing NTDs part of your role, for example performing clinical examinations or requesting laboratory tests?
- Is managing NTDs part of your role, for example providing treament or referring patients?

B Job aids

18. Were you given any materials during or after the training to help you remember what was taught and to help you detect and manage NTDs?

- Did you receive those materials?
- Do you actually use those job aids in your job? How often? If not, why not?
- *[if job aids not used]* Could the job aids have been developed in a way that would have made it more likely for you to use them?
- *[if job aids used]* What do you like and dislike about the job aids?
- *[if job aids used]* How could the job aids be improved?

C Disease detection and management

19. The training and the materials that were distributed provide suggestions for how health workers at hospital level should detect and manage six diseases: trachoma, urinary schistosomiasis, intestinal schistosomiasis, soil-transmitted helminth infection, lymphatic

filariasis and podoconiosis. Do you ever see patients with signs and symptoms of any of the diseases?

- *[For the diseases the health worker encounters]* How often does this happen?
- *[For the diseases the health worker does not encounter]* Why do you think this is the case?

[For the next section of the interview, pick questions relating to <u>*two*</u> *diseases per interview, at least one from each of the following two groups:*

1) Group 1: trachoma, lymphatic filariasis, podoconiosis;

2) Group 2: urinary schistosomisis, intestinal schistosomiasis, soil-transmitted helminth infection.

Preference should be given to diseases the health worker has indicated they encounter in their job. Over the course of all interviews with health workers, each disease should be covered at least once.]

Trachoma

20. **Do you know how health workers at hospital level usually detect trachoma?**
    - What signs and symptoms would you look out for?
21. **If a patient has signs and symptoms of trachoma, do you know how health workers at hospital level usually reach a diagnosis?**

    [only probe if participant has indicated their role involves diagnosis of NTDs]
    - Would you perform any particular clinical examination?
22. **If a patient is diagnosed with trachoma, do you know how those cases are usually managed at hospital level?**

    [only probe if participant has indicated their role involves management of NTDs]
    - Would you provide any treatment? If yes, what treatment would you provide?
    - Under what circumstances would you refer the patient? Where would you refer the patient to?
    - Would you recommend any kind of follow-up?
23. **The intervention we are testing suggests that health workers at hospital level should detect trachoma based on the following symptoms: irritated red eye, mucopurulant discharge, eye pain or inverted eye lashes. On further examination of eyelashes, eyeball and conjunctiva, health workers should be able to diagnose trachomatous inflammation or trichiasis. Do you think health workers at hospital level can perform these responsibilities?**
    - If no, why not?
    - How would you say the intervention is different from your current practice?
    - In your opinion, are there any problems with tasking health workers at health hospital with detecting trachoma as suggested by the intervention?

- In your opinion, what are the implications of performing this task in terms of time and workload?
- In your experience, are infastructure and equipment required to perform this task normally available at this hospital?
- How could the problems you mentioned be addressed? What alternatives or improvements would you suggest?

24. The intervention suggests that patients diagnosed with trachomatous inflammation should receive azithromycin or tetracycline. In addition, patients with trichiasis should receive surgery. Do you think health workers at hospital level can perform these responsibilities?

- If no, why not?
- How would you say the intervention is different from your current practice?
- In your opinion, are there any problems with tasking health workers at hospital level with managing trachoma as suggested by the intervention?

[only use the following probes if participant has indicated their role involves management of NTDs]

- In your opinion, what are the implications of performing this task in terms of time and workload?
- In your experience, are drugs, infastructure and equipment required to perform this task normally available at this hospital?
- How could the problems you mentioned be addressed? What alternatives or improvements would you suggest?

Urinary schistosomiasis

25. Do you know how health workers at hospital level usually detect urinary schistosomiasis?

- What signs and symptoms would you look out for?

26. If a patient has signs and symptoms of trachoma, do you know how health workers at hospital level usually reach a diagnosis?

[only probe if participant has indicated their role involves diagnosis of NTDs]

- Would you perform any particular clinical examination?
- Would you request a particular laboratory test?

27. If a patient is diagnosed with urinary schistosomiasis, do you know how those cases are normally managed at hospital level?

[only probe if participant has indicated their role involves management of NTDs]

- Would you provide any treatment? If yes, what treatment would you provide?
- Under what circumstances would you refer the patient? Where would you refer the patient to?
- Would you recommend any kind of follow-up?

28. **The intervention we are testing suggests that health workers at hospital level should detect urinary schistosomiasis based on the following symptoms: inchildren up to five years: blood in urine; in children over five and adults: blood in urine, pain while urinating, pelvic pain, genital itching or burning sensation, involuntary urination when coughing, laughing or jumping. Do you think health workers at hospital level can perform these responsibilities?**

- If no, why not?
- How would you say the intervention is different from your current practice?
- In your opinion, are there any problems with tasking health workers at hospital level with detecting urinary schistosomiasis as suggested by the intervention?
- In your opinion, what are the implications of performing this task in terms of time and workload?
- How could the problems you mentioned be addressed? What alternatives or improvements would you suggest?

29. The intervention suggests that a urinary schistosomiasis diagnosis should be confirmed through a urine sedimentation test. Is this a test you think can be performed at the hospital?

- If not, why not?

[only probe if participant has indicated their role involves diagnosis of NTDs]

- If yes, in your opinion, are there any challenges with performing urine sedimentation tests at hospital level as suggested by the intervention?
- How could those problems be addressed? What alternatives would you suggest?

30. The intervention suggests that patients diagnosed with urinary schistosomiasis should receive praziquantel if the urine sedimentation test is positive. Patients over five years with any of the symptoms of urinary schistosomiasis should be given praziquantel presumptively if it is not possible to perform a urine sedimentation test. Do you feel confident that health workers at hospital level can perform these responsibilities?

- If no, why not?
- How would you say the intervention is different from your current practice?
- In your opinion, are there any problems with tasking health workers at hospital level with managing urinary schistosomiasis as suggested by the intervention?

[only use the following probes if participant has indicated their role involves management of NTDs]

- In your opinion, what are the implications of performing this task in terms of time and workload?
- In your experience, are drugs and equipment required to perform this task normally available at this hospital?
- How could the problems you mentioned be addressed? What alternatives or improvements would you suggest?

Intestinal schistosomiasis

31. Do you know how health workers at hospital level usually detect intestinal schistosomiasis?

- What signs and symptoms would you look out for?

32. If a patient has signs and symptoms of trachoma, do you know how health workers at hospital level usually reach a diagnosis?

[only probe if participant has indicated their role involves diagnosis of NTDs]

- Would you perform any particular clinical examination?
- Would you request a particular laboratory test?

33. If a patient is diagnosed with intestinal schistosomiasis, do you know how those cases are usually managed at hospital level?

[only probe if participant has indicated their role involves management of NTDs]

- Would you provide any treatment? If yes, what treatment would you provide?
- Under what circumstances would you refer the patient? Where would you refer the patient to?
- Would you recommend any kind of follow-up?

34. The intervention we are testing suggests that health workers at hospital level should detect intestinal schistosomiasis based on whether the area where the patient resides is known to be endemic for the disease and the following symptoms: diarrhoea, anaemia, malnutrition, abdominal pain, jaundice,ascites, intestinal blockage. Do you feel confident that health workers at hospital level can perform these responsibilities?

- If no, why not?
- How would you say the intervention is different from your current practice?
- In your opinion, are there any problems with tasking health workers at hospital level with detecting intestinal schistosomiasis as suggested by the intervention?
- In your opinion, what are the implications of performing this task in terms of time and workload?
- How could the problems you mentioned be addressed? What alternatives or improvements would you suggest?

35. The intervention suggests that an intestinal schistosomiasis diagnosis should be confirmed through a CCA test for patients up to five years or a stool concentration test for patients over five. Are these tests you think can be performed at the hospital?

- If not, why not?

[only probe if participant has indicated their role involves diagnosis of NTDs]

- In your opinion, are there any problems with performing CCA and stool concentration tests at hospital level as suggested by the intervention?
- How could those problems be addressed? What alternatives would you suggest?

36. **The intervention suggests that patients diagnosed with intestinal schistosomiasis should receive praziquantel if the CCA or stool concentration tests are positive, including patients up to five years. Patients over five years with any of the symptoms of intestinal schistosomiasis should be given praziquantel presumptively if it is not possible to perform a CCA or stool concentration test. Do you think health workers at hospital level have can perform these responsibilities?**

    - If no, why not?
    - How would you say the intervention is different from your current practice?
    - In your opinion, are there any problems with tasking health workers at hospital level with managing intestinal schistosomiasis as suggested by the intervention?

    [only use the following probes if participant has indicated their role involves management of NTDs]

    - In your opinion, what are the implications of performing this task in terms of time and workload?
    - In your experience, are drugs and equipment required to perform this task normally available at this hospital?
    - How could the problems you mentioned be addressed? What alternatives or improvements would you suggest?

Soil-transmitted helminth infection

37. **Do you know how health workers at hospital level usually detect soil-transmitted helminth infections?**

    - What signs and symptoms would you look out for?

38. **If a patient has signs and symptoms of soil-transmitted helminth infection, do you know how health workers at hospital level usually reach a diagnosis?**

    [only probe if participant has indicated their role involves diagnosis of NTDs]

    - Would you perform any particular clinical examination?
    - Would you request a particular laboratory test?

39. **If a patient is diagnosed with soil-transmitted helminth infection, do you know how those cases are usually managed at hospital level?**

    [only probe if participant has indicated their role involves management of NTDs]

    - Would you provide any treatment? If yes, what treatment would you provide?
    - Under what circumstances would you refer the patient? Where would you refer the patient to?
    - Would you recommend any kind of follow-up?

40. **The intervention suggests that health workers at hospital level should detect soil-transmitted helminth infections based on whether the area where the patient resides is known to be**

endemic for the disease and the following symptoms: worms coming out of mouth/nose/anus, non-malaria fever and vomit, diarrhoea, anaemia, malnutrition, abdominal pain, jaundice, ascites, intestinal blockage, rectal prolapse, finger or nail clubbing. Do you think health workers at hospital level can perform these responsibilities?

- If no, why not?
- How would you say the intervention is different from your current practice?
- In your opinion, are there any problems with tasking health workers at hospital level with detecting soil-transmitted helminth infections as suggested by the intervention?
- In your opinion, what are the implications of performing this task in terms of time and workload?
- How could the problems you mentioned be addressed? What alternatives or improvements would you suggest?

41. **The intervention suggests that soil-transmitted helminth infections should be confirmed through a a stool concentration test. Is this a test you think can be performed at the hospital?**

- If not, why not?

[only probe if participant has indicated their role involves diagnosis of NTDs]

- In your opinion, are there any problems with performing stool concentration tests at hospital level as suggested by the intervention?
- How could those problems be addressed? What alternatives would you suggest?

42. **The intervention suggests that patients diagnosed with soil-transmitted helminth infection should receive albendazole if the stool concentration test is positive for ascariasis & hookworm infection. Other drugs are recommended if the stool concentration test is positive for other worm infections. Albendazole should be given presumptively if it is not possible to perform a stool concentration test. Do you think health workers at hospital level can perform these responsibilities?**

- If no, why not?
- How would you say the intervention is different from your current practice?

[only use the following probes if participant has indicated their role involves management of NTDs]

- In your opinion, are there any problems with tasking health workers at hospital level with managing soil-transmitted helminth infections as suggested by the intervention?
- In your opinion, what are the implications of performing this task in terms of time and workload?
- In your experience, are the drugs required to perform this task normally available at this hospital?
- How could the problems you mentioned be addressed? What alternatives or improvements would you suggest?

Lymphatic filariasis

43. Do you know how health workers at hospital level usually detect lymphatic filariasis?

- What signs and symptoms would you look out for?

44. If a patient has signs and symptoms of lymphatic filariasis, do you know how health workers at hospital level usually reach a diagnosis?

[only probe if participant has indicated their role involves diagnosis of NTDs]

- Would you perform any particular clinical examination?

45. If a patient is diagnosed with lymphatic filariasis, do you know how those cases are usually managed at hospital level?

[only probe if participant has indicated their role involves management of NTDs]

- Would you provide any treatment? If yes, what treatment would you provide?
- Under what circumstances would you refer the patient? Where would you refer the patient to?
- Would you recommend any kind of follow-up?

46. The intervention we are testing suggests that health workers at hospital level should detect lymphatic filariasis based on whether the area where the patient resides is known to be endemic for the disease the following symptoms: non-malaria fever and chills and redness of the leg, swelling of a lower limb, pain in a lower limb, elephantiasis. In patients over five years, the disease should also be detected based on the following symptoms: swelling of breast, swelling of an upper limb, swelling of the scrotum or vulva, hydrocele. Do you think health workers at hospital level can perform these responsibilities?

- If no, why not?
- How would you say the intervention is different from your current practice?
- In your opinion, are there any problems with tasking health workers at hospital level with detecting lymphatic filariasis as suggested by the intervention?
- In your opinion, what are the implications of performing this task in terms of time and workload?
- In your experience, are infastructure and equipment required to perform this task normally available at this hospital?
- How could the problems you mentioned be addressed? What alternatives or improvements would you suggest?

47. The intervention suggests that patients diagnosed with acute lymphatic filariasis should receive pain relief with paracetamol, and be advised to hydrate and rest. All patients should be referred to a health post for advise on home management of lymphedoema. Patients with hydrocele should be referred to an appropriate hospital for surgery.Do you think health workers at hospital level can perform these responsibilities?

- If no, why not?

- How would you say the intervention is different from your current practice?

[only probe if participant has indicated their role involves diagnosis of NTDs]

- In your opinion, are there any problems with tasking health workers at hospital level with managing lymphatic filariasis as suggested by the intervention?
- In your opinion, what are the implications of performing this task in terms of time and workload?
- In your experience, are drugs, infastructure and equipment required to perform this task normally available at this hospital?
- How could the problems you mentioned be addressed? What alternatives or improvements would you suggest?

Podoconiosis

48. Do you know how health workers at hospital level usually detect podoconiosis?

- What signs and symptoms would you look out for?

49. If a patient has signs and symptoms of podoconiosis, do you know how health workers at hospital level usually reach a diagnosis?

[only probe if participant has indicated their role involves diagnosis of NTDs]

- Would you perform any particular clinical examination?

50. If a patient is diagnosed with podoconiosis, do you know how those cases are usually managed at hospital level?

[only probe if participant has indicated their role involves management of NTDs]

- Would you provide any treatment? If yes, what treatment would you provide?
- Under what circumstances would you refer the patient? Where would you refer the patient to?
- Would you recommend any kind of follow-up?

51. The intervention we are testing suggests that health workers at hospital level should detect podoconiosis based on whether the area where the patient resides is known to be endemic for the disease the following symptoms: non-malaria fever and chills and redness of the leg, swelling of a lower limb, pain in a lower limb, itching/burning sensation of the skin on leg, enlargement of the foot, plantar oedema, skin nodules, hyperkeratosis, moss-like papillomata, joint fixation. Do you think health workers at hospital level can perform these responsibilities?

- If no, why not?
- How would you say the intervention is different from your current practice?
- In your opinion, what are the implications of performing this task in terms of time and workload?

- In your experience, are infastructure and equipment required to perform this task normally available at this hospital?
- How could the problems you mentioned be addressed? What alternatives or improvements would you suggest?

52. The intervention suggests that patients diagnosed with acute podoconiosis should receive pain relief with paracetamol, and be advised to hydrate and rest. All patients should be referred to a health post for advise on home management of lymphedoema. Do you think health workers at hospital level can perform these responsibilities?

- If no, why not?
- How would you say the intervention is different from your current practice?

[only probe if participant has indicated their role involves diagnosis of NTDs]

- In your opinion, are there any problems with tasking health workers at hospital level with managing podoconiosis as suggested by the intervention?
- In your opinion, what are the implications of performing this task in terms of time and workload?
- In your experience, are drugs, infastructure and equipment required to perform this task normally available at this hospital?
- How could the problems you mentioned be addressed? What alternatives or improvements would you suggest?

D Overall perception of the intervention

53. What do you generally think of the idea of tasking health workers at primary health care level (including health posts and health centres) with detecting, managing, recording and reporting neglected tropical diseases?

- Do you think the primary health care system has the capacity to take on these roles and responsibilities?
- What kind of challenges do you foresee?
- Do you think there would be any challenges with regard to gender? Would women and men face different challenges in accessing care for NTDs at the hospital?
- Do you think there would be any challenges with regard to stigma? Would those affected by disabilities caused by NTDs access care at the hospital?
- What could be done to address those challenges?
- What would your recommendations with regard to integrating the detection, management, recording and reporting of NTDs into primary health care?

54. Is there anything else you would like to share with the research team?

This concludes the interview. Many thanks for taking the time to participate.

Key Informant Interviews – laboratory staff (hospital)

Interview details

**Interview number**				
**Interviewee code**				
**Sex**		Female		Male
**Attended classroom training?**		Yes		No
**Job title**				
**Name of interviewer**				
**Name of notetaker**				
**Date**				
**Time started**				
**Time finished**				
**Venue**				

Consent

Before starting the interview, informed written consent from the participant must be obtained. Please follow the script provided:

Good morning/ good afternoon

My name is [*name*] and this is my colleague [*names*]. We work for Malaria Consortium, a not-for-profit organisation that specialises in the prevention, control and treatment of malaria and other diseases. In collaboration with the Federal Ministry of Health in Ethiopia, we are currently carrying out a study to explore ways of improving care for a group of infectious diseases called 'neglected tropical diseases' or 'NTDs'. We are testing an intervention that we hope will improve health workers' and Health Extension Workers' ability to detect, manage, record and report those diseases. The intervention has been implmented at your health facility over the last few months and we would like to get your feedback on some aspects of the intervention. Your responses will help us to evaluate the intervention, find out what worked and what didn't and improve the intervention in the future.

All the information you provide will be strictly confidential and your replies will only be shared with the other researchers working on this study. You will be identified by a number, not your name. We have put together an information sheet which summarises the purpose of the interview, why you've been selected and what happens if you agree to participate. Please take a moment to read through the information provided on the sheet.

Do you have any questions about the information provided on this sheet?

Remember that you do not have to take part in the interview if you prefer not to. You do not have to answer questions you don't feel comfortable with and can end the interview at any time. If you agree to participate, we will ask you to sign a consent form. We will audio-record the interview and take notes. This will help us remember what you are telling us. We will destroy the audio-recording once the report for this project has been written. Can I please ask you to read this consent form?

Do you have any questions about the information in this form?

If you consent to participating in the interview, can I please ask you to sign the form?

Thank you very much for agreeing to participate in the interview.

Topic guide

A Training

55. **In May, training was offered to health workers from your hospital about nelected tropical diseases. Did you attend this training?**

56. *[If training attended]* **Can you tell me what you learned in this training?**
    - What was the most interesting thing you learned at the training?
    - Do you remember which laboratory test this training covered and which diseases are detected with those tests?
    - What did you like about the training?
    - Was there anything you didn't like about the training?
    - How could the training have been improved?
    - Did you think the training was relevant for your job? Why or why not?
    - After the training, what is your understanding of your role with performing those tests?

57. *[If training not attended]* **Did anyone tell you about this training?**
    - What did others tell you about this training?
    - Do you know what the training was about? Which tests were covered and which diseases are detected with those tests?
    - Do you think the training might have been relevant for your job? Why or why not?
    - Were you told what, according to this training, laboratory staff's role would be with regard to performing those tests?

C Laboratory tests

58. **The training covered thee laboratory tests: urinary sedimentation, stool concentration and Circulating Cathodic Antigen (CCA) test. Do you ever perform those tests?**
    - *[For the tests the interviewee performs]* How often does this happen?
    - *[For the diseases the interviewee does not perform]* Why do you think this is the case?

59. **Do you know how to perform a urine sedimentation test?**
    - Which diseases can be detected with this test?
    - What kind of materials and equipment do you need to perform the test?
    - How much time does it take to perform the test?
    - How difficult is it to perform the test?

60. **The intervention we are testing suggests that laboratory staff at hospital level should use the urine sedimentation test to detect urinary schistosomiasis. Do you feel confident that you have the knowledge and skills to perform this test?**
    - If yes, what gives you the confidence?

- If no, why not?
- Do health workers at this hospital ever request this test? If no, why not?
- If you have performed tests to detect urinary schistosomiasis recently, did you use the urinary sedimentation test? If not, why not and what did you do instead?
- In your opinion, are there any problems with performing urinary sedimentaiton tests at hospital level as suggested by the intervention?
- In your opinion, what are the implications of performing this test in terms of time and workload?
- In your experience, are infastructure and equipment required to perform this test normally available at this hospital?
- Do you think the test results are reliable?
- How could the problems you mentioned be addressed? What alternatives or improvements would you suggest?

61. Do you know how to perform a stool concentration test?

- Which diseases can be detected with this test?
- What kind of materials and equipment do you need to perform the test?
- How much time does it take to perform the test?
- How difficult is it to perform the test?

62. The intervention suggests that laboratory staff at hospital level should use the stool concentration test to detect intestinal schistosomiasis in patients over five and and soil-transmitted helminth infections in all patients. Do you feel confident that you have the knowledge and skills to perform these tests?

- If yes, what gives you the confidence?
- If no, why not?
- Do health workers at this health hospital ever request this test? If no, why not?
- If you have performed tests to detect intestinal schistosomiasis or soil-transmitted helminth infections recently, did you use the stool concentration test? If not, why not and what did you do instead?
- In your opinion, are there any problems with performing stool concentration tests at hospital level as suggested by the intervention?
- In your opinion, what are the implications of performing this test in terms of time and workload?
- In your experience, are infastructure and equipment required to perform this test normally available at this hospital?
- Do you think the test results are reliable?

- How could the problems you mentioned be addressed? What alternatives or improvements would you suggest?

63. Do you know how to perform a Circulating Cathodic Antigen (CCA) test?

- Which diseases can be detected with this test?
- What kind of materials and equipment do you need to perform the test?
- How much time does it take to perform the test?
- How difficult is it to perform the test?

64. The intervention suggests that laboratory staff at hospital level should use a Circulating Cathodic Antigen (CCA) test to detect intestinal schistosomiasis in patients up to five years. Test kits were provided to the hospital as part of the intervention. Do you feel confident that you have the knowledge and skills to perform this test?

- If yes, what gives you the confidence?
- If no, why not?
- Do health workers at this hospital ever request this test? If no, why not?
- If you have performed CCA tests to detect intestinal schistosomiasis, did you use the CCA test? If not, why not and what did you do instead?
- In your opinion, are there any problems with performing CCA tests at hospital level as suggested by the intervention?
- In your opinion, what are the implications of performing this test in terms of time and workload?
- Do you think the test results are reliable?
- How could the problems you mentioned be addressed? What alternatives or improvements would you suggest?

C Overall perception of the intervention

65. What do you generally think of tasking laboratory staff at hospital level with performing tests to detect neglected tropical diseases?

66. And more generally, what do you think about the idea of tasking health workers at primary health care level (including health posts and health centres) with detecting, managing, ercording and reporting neglected tropical diseases?

- Do you think the primary health care system has the capacity to take on these roles and responsibilities?
- What kind of challenges do you foresee?
- Do you think there would be any challenges with regard to gender? Would women and men face different challenges in accessing care for NTDs at the hospital?
- Do you think there would be any challenges with regard to stigma? Would those affected by disabilities caused by NTDs access care at the hospital?

- What could be done to address those challenges?
- What would your recommendations with regard to integrating the detection and management of NTDs into primary health care?

67. Is there anything else you would like to share with the research team?

This concludes the interview. Many thanks for taking the time to participate.

<u>Health Centre Level</u>

Key Informant Interviews – data management staff (health centre)

Interview details

**Interview number**				
**Interviewee code**				
**Sex**		Female		Male
**Attended classroom training**		Yes		No
**Job title**				
**Name of interviewer**				
**Name of notetaker**				
**Date**				
**Time started**				
**Time finished**				
**Venue**				

Consent

Before starting the interview, informed written consent from the participant must be obtained. Please follow the script provided:

Good morning/ good afternoon

My name is [*name*] and this is my colleague [*names*]. We work for Malaria Consortium, a not-for-profit organisation that specialises in the prevention, control and treatment of malaria and other diseases. In collaboration with the Federal Ministry of Health in Ethiopia, we are currently carrying out a study to explore ways of improving care for a group of infectious diseases called 'neglected tropical diseases' or 'NTDs'. We are testing an intervention that we hope will improve health workers' and Health Extension Workers' ability to detect, manage, record and report those diseases. The intervention has been implmented at your health facility over the last few months and we would like to get your feedback on some aspects of the intervention. Your responses will help us to evaluate the intervention, find out what worked and what didn't and improve the intervention in the future.

All the information you provide will be strictly confidential and your replies will only be shared with the other researchers working on this study. You will be identified by a number, not your name. We have put together an information sheet which summarises the purpose of the interview, why you've been selected and what happens if you agree to participate. Please take a moment to read through the information provided on the sheet.

Do you have any questions about the information provided on this sheet?

Remember that you do not have to take part in the interview if you prefer not to. You do not have to answer questions you don't feel comfortable with and can end the interview at any time. If you agree to participate, we will ask you to sign a consent form. We will audio-record the interview and take notes. This will help us remember what you are telling us. We will destroy the audio-recording once the report for this project has been written. Can I please ask you to read this consent form?

Do you have any questions about the information in this form?

If you consent to participating in the interview, can I please ask you to sign the form?

Thank you very much for agreeing to participate in the interview.

Topic guide

A Training

68. **In May, training was offered to health workers from your health centre about nelected tropical diseases. Did you attend this training?**

69. *[If training attended]* **Can you tell me what you learned in this training?**

    - What was the most interesting thing you learned at the training?
    - Do you remember which diseases this training covered?
    - What did you like about the training?
    - Was there anything you didn't like about the training?
    - How could the training have been improved?
    - Did you think the training was relevant for your job? Why or why not?
    - After the training, what is your understanding of your role with regard to recording and reporting neglected tropical diseases?

70. *[If training not attended]* **Did anyone tell you about this training?**

    - What did others tell you about this training?
    - Do you know what the training was about? Which tests were covered and which diseases are detected with those tests?
    - Do you think the training might have been relevant for your job? Why or why not?
    - Were you told what, according to this training, laboratory staff's role would be with regard to recording and reporting neglected tropical diseases?

C Recording and reporting

71. **The training covered six diseases: trachoma, urinary schistosomiasis, intestinal schistosomiasis, soil-transmitted helminth infections, lymphatic filariasis and podoconiosis. Do you ever record and report any of those diseases?**

    - *[For the diseases the interviewee records/reports]* How often does this happen?
    - How exactly do you record and report those diseases?
    - *[For the diseases the interviewee does not record/report]* Why do you think this is the case?
    - How would patients with those diseases be recorded/reported?

72. **The intervention we are testing suggests that trachoma cases should be recorded as "confirmed trachoma" if diagnosed based on a list of signs and symptoms. In your experience, does this currently happen at this health centre?**

    - If no, why not?

- In your opinion, are there any problems with recording trachoma cases as suggested by the intervention?
- How could the problems you mentioned be addressed? What alternatives or improvements would you suggest?

73. **The intervention suggests that urinary schistosomiasis cases should be recorded as "confirmed urinary schistosomiasis" if confirmed by a urinary sedimentation test or "suspected urinary schistosomiasis" if the disease is suspected but not confirmed through a laboratory test. In your experience, does this currently happen at this health centre?**
    - If no, why not?
    - In your opinion, are there any problems with recording urinary schistosomiasis cases as suggested by the intervention?
    - How could the problems you mentioned be addressed? What alternatives or improvements would you suggest?
74. **The intervention suggests that intestinal schistosomiasis cases should be recorded as "confirmed intestinal schistosomiasis" if confirmed by a CCA or stool concentration test or "suspected helminthiasis" if the disease is suspected but not confirmed through a laboratory test. In your experience, does this currently happen at this health centre?**
    - If no, why not?
    - In your opinion, are there any problems with recording intestinal schistosomiasis cases as suggested by the intervention?
    - How could the problems you mentioned be addressed? What alternatives or improvements would you suggest?
75. **The intervention suggests that soil-transmitted helminth infection cases should be recorded as "confirmed soil-transmitted helminth infections" if confirmed by a stool concentration test or "suspected helminthiasis" if the disease is suspected but not confirmed through a laboratory test. In your experience, does this currently happen at this health centre?**
    - If no, why not?
    - In your opinion, are there any problems with recording urinary schistosomiasis cases as suggested by the intervention?
    - How could the problems you mentioned be addressed? What alternatives or improvements would you suggest?
76. **The intervention we are testing suggests that lymphatic filariasis cases should be recorded as "morbidity related to lymphatic filariasis" if diagnosed based on a list of signs and symptoms. In your experience, does this currently happen at this health centre?**
    - If no, why not?
    - In your opinion, are there any problems with recording trachoma cases as suggested by the intervention?

- How could the problems you mentioned be addressed? What alternatives or improvements would you suggest?

77. The intervention we are testing suggests that podoconiosis cases should be recorded as "morbidity related to podoconiosis" if diagnosed based on a list of signs and symptoms. In your experience, does this currently happen at this health centre?

- If no, why not?
- In your opinion, are there any problems with recording trachoma cases as suggested by the intervention?
- How could the problems you mentioned be addressed? What alternatives or improvements would you suggest?

C Overall perception of the intervention

78. What do you generally think of tasking data management staff at health centre level with recording and reporting neglected tropical diseases?

- In your opinion, what are the implications of performing this task in terms of time and workload?
- Do you think the data recorded and reported will be reliable?
- How could the problems you mentioned be addressed? What alternatives or improvements would you suggest?

79. And more generally, what do you think about the idea of tasking health workers at primary healh care level (including health posts and hospitals) with detecting, managing, recording and reporting neglected tropical diseases?

- Do you think the primary health care system has the capacity to take on these roles and responsibilities?
- What kind of challenges do you foresee?
- Do you think there would be any challenges with regard to gender? Would women and men face different challenges in accessing care for NTDs at the health centre?
- Do you think there would be any challenges with regard to stigma? Would those affected by disabilities caused by NTDs access care at the health centre?
- What could be done to address those challenges?
- What would your recommendations with regard to integrating the detection, management, recording and reporting of NTDs into primary health care?

80. Is there anything else you would like to share with the research team?

This concludes the interview. Many thanks for taking the time to participate

Key Informant Interviews – health workers (health centre)

Interview details

**Interview number**				
**Interviewee code**				
**Sex**		Female		Male
**Attended classroom training?**		Yes		No
**Section/department**		Paediatrics		
		OPD		
		kebele/Health Post Support		
		Other (specify:)		
**Cadre**		Nurse		
		Health Officer		
		Other (specify:)		
**Job title**				
**Name of interviewer**				
**Name of notetaker**				
**Date**				
**Time started**				
**Time finished**				
**Venue**				

Consent

Good morning/ good afternoon

My name is [*name*] and this is my colleague [*names*]. We work for Malaria Consortium, a not-for-profit organisation that specialises in the prevention, control and treatment of malaria and other diseases. In collaboration with the Federal Ministry of Health in Ethiopia, we are currently carrying out a study to explore ways of improving care for a group of infectious diseases called 'neglected tropical diseases' or 'NTDs'. We are testing an intervention that we hope will improve health workers' and Health Extension Workers' ability to detect, manage, record and report those diseases. The intervention has been implmented at your health facility over the last few months and we would like to get your feedback on some aspects of the intervention. Your responses will help us to evaluate the intervention, find out what worked and what didn't and improve the intervention in the future.

All the information you provide will be strictly confidential and your replies will only be shared with the other researchers working on this study. You will be identified by a number, not your name. We have put together an information sheet which summarises the purpose of the interview, why you've been selected and what happens if you agree to participate. Please take a moment to read through the information provided on the sheet.

Do you have any questions about the information provided on this sheet?

Remember that you do not have to take part in the interview if you prefer not to. You do not have to answer questions you don't feel comfortable with and can end the interview at any time. If you agree to participate, we will ask you to sign a consent form. We will audio-record the interview and take notes. This will help us remember what you are telling us. We will destroy the audio-recording once the report for this project has been written. Can I please ask you to read this consent form?

Do you have any questions about the information in this form?

If you consent to participating in the interview, can I please ask you to sign the form?

Thank you very much for agreeing to participate in the interview.

Topic guide

A Training

81. In May this year, classroom training was offered to health workers from your health centre about nelected tropical diseases. Did you attend this training?

82. *[If training attended]* **Can you tell me what you learned in this training?**

- Do you remember which diseases this training covered?
- What did you like about the training?
- Was there anything you didn't like about the training?
- How could the training have been improved?
- Did you think the training was relevant for your job? Why or why not?

83. *[If training not attended]* **Did anyone tell you about this training?**

- What did others tell you about this training?
- Do you know what the training was about? Which diseases were covered?
- Do you think the training might have been relevant for your job? Why or why not?

84. **What is your understanding of your role with regard to detecting and managing NTDs?**

- Is recognising the signs and symptoms of NTDs part of your role?
- Is diagnosing NTDs part of your role, for example performing clinical examinations or requesting laboratory tests?
- Is managing NTDs part of your role, for example providing treament or referring patients?

B Job aids

85. Were you given any materials during or after the training to help you remember what was taught and to help you detect and manage NTDs?

- Did you receive those materials?
- Do you actually use those job aids in your job? How often? If not, why not?
- *[if job aids not used]* Could the job aids have been developed in a way that would have made it more likely for you to use them?
- *[if job aids used]* What do you like and dislike about the job aids?
- *[if job aids used]* How could the job aids be improved?

C Disease detection and management

86. The training and the materials that were distributed provide suggestions for how health workers at health centre level should detect and manage six diseases: trachoma, urinary schistosomiasis, intestinal schistosomiasis, soil-transmitted helminth infection, lymphatic

filariasis and podoconiosis. Do you ever see patients with signs and symptoms of any of the diseases?

- *[For the diseases the health worker encounters]* How often does this happen?
- *[For the diseases the health worker does not encounter]* Why do you think this is the case?

[For the next section of the interview, pick questions relating to two diseases per interview, at least one from each of the following two groups:

1) Group 1: trachoma, lymphatic filariasis, podoconiosis;

2) Group 2: urinary schistosomisis, intestinal schistosomiasis, soil-transmitted helminth infection.

Preference should be given to diseases the health worker has indicated they encounter in their job. Over the course of all interviews with health workers, each disease should be covered at least once.]

Trachoma

87. **Do you know how health workers at health centre level usually detect trachoma?**
    - What signs and symptoms would you look out for?
88. **If a patient has signs and symptoms of trachoma, do you know how health workers at health centre level usually reach a diagnosis?**

    [only probe if participant has indicated their role involves diagnosis of NTDs]
    - Would you perform any particular clinical examination?
89. **If a patient is diagnosed with trachoma, do you know how those cases are usually managed at health centre level?**

    [only probe if participant has indicated their role involves management of NTDs]
    - Would you provide any treatment? If yes, what treatment would you provide?
    - Under what circumstances would you refer the patient? Where would you refer the patient to?
    - Would you recommend any kind of follow-up?
90. **The intervention we are testing suggests that health workers at health centre level should detect trachoma based on the following symptoms: irritated red eye, mucopurulant discharge, eye pain or inverted eye lashes. On further examination of eyelashes, eyeball and conjunctiva, health workers should be able to diagnose trachomatous inflammation or trichiasis. Do you think health workers at health centre level can perform these responsibilities?**
    - If no, why not?
    - How would you say the intervention is different from your current practice?
    - In your opinion, are there any problems with tasking health workers at health health centre with detecting trachoma as suggested by the intervention?

- In your opinion, what are the implications of performing this task in terms of time and workload?
- In your experience, are infastructure and equipment required to perform this task normally available at this health centre?
- How could the problems you mentioned be addressed? What alternatives or improvements would you suggest?

91. **The intervention suggests that patients diagnosed with trachomatous inflammation should receive azithromycin or tetracycline. In addition, patients with trichiasis should receive surgery. Do you think health workers at health centre level can perform these responsibilities?**
    - If no, why not?
    - How would you say the intervention is different from your current practice?
    - In your opinion, are there any problems with tasking health workers at health centre level with managing trachoma as suggested by the intervention?

[only use the following probes if participant has indicated their role involves management of NTDs]

- In your opinion, what are the implications of performing this task in terms of time and workload?
- In your experience, are drugs, infastructure and equipment required to perform this task normally available at this health centre?
- How could the problems you mentioned be addressed? What alternatives or improvements would you suggest?

Urinary schistosomiasis

92. **Do you know how health workers at health centre level usually detect urinary schistosomiasis?**
    - What signs and symptoms would you look out for?
93. **If a patient has signs and symptoms of trachoma, do you know how health workers at health centre level usually reach a diagnosis?**

    [only probe if participant has indicated their role involves diagnosis of NTDs]
    - Would you perform any particular clinical examination?
    - Would you request a particular laboratory test?
94. **If a patient is diagnosed with urinary schistosomiasis, do you know how those cases are normally managed at health centre level?**

    [only probe if participant has indicated their role involves management of NTDs]
    - Would you provide any treatment? If yes, what treatment would you provide?
    - Under what circumstances would you refer the patient? Where would you refer the patient to?

- Would you recommend any kind of follow-up?

95. **The intervention we are testing suggests that health workers at health centre level should detect urinary schistosomiasis based on the following symptoms: inchildren up to five years: blood in urine; in children over five and adults: blood in urine, pain while urinating, pelvic pain, genital itching or burning sensation, involuntary urination when coughing, laughing or jumping. Do you think health workers at health centre level can perform these responsibilities?**

    - If no, why not?
    - How would you say the intervention is different from your current practice?
    - In your opinion, are there any problems with tasking health workers at health centre level with detecting urinary schistosomiasis as suggested by the intervention?
    - In your opinion, what are the implications of performing this task in terms of time and workload?
    - How could the problems you mentioned be addressed? What alternatives or improvements would you suggest?

96. **The intervention suggests that a urinary schistosomiasis diagnosis should be confirmed through a urine sedimentation test. Is this a test you think can be performed at the health centre?**

    - If not, why not?

    [only probe if participant has indicated their role involves diagnosis of NTDs]

    - If yes, in your opinion, are there any challenges with performing urine sedimentation tests at health centre level as suggested by the intervention?
    - How could those problems be addressed? What alternatives would you suggest?

97. **The intervention suggests that patients diagnosed with urinary schistosomiasis should receive praziquantel if the urine sedimentation test is positive. Patients over five years with any of the symptoms of urinary schistosomiasis should be given praziquantel presumptively if it is not possible to perform a urine sedimentation test. Do you feel confident that health workers at health centre level can perform these responsibilities?**

    - If no, why not?
    - How would you say the intervention is different from your current practice?
    - In your opinion, are there any problems with tasking health workers at health centre level with managing urinary schistosomiasis as suggested by the intervention?

    [only use the following probes if participant has indicated their role involves management of NTDs]

    - In your opinion, what are the implications of performing this task in terms of time and workload?
    - In your experience, are drugs and equipment required to perform this task normally available at this health centre?

- How could the problems you mentioned be addressed? What alternatives or improvements would you suggest?

Intestinal schistosomiasis

98. Do you know how health workers at health centre level usually detect intestinal schistosomiasis?

- What signs and symptoms would you look out for?

99. If a patient has signs and symptoms of trachoma, do you know how health workers at health centre level usually reach a diagnosis?

[only probe if participant has indicated their role involves diagnosis of NTDs]

- Would you perform any particular clinical examination?
- Would you request a particular laboratory test?

100. If a patient is diagnosed with intestinal schistosomiasis, do you know how those cases are usually managed at health centre level?

[only probe if participant has indicated their role involves management of NTDs]

- Would you provide any treatment? If yes, what treatment would you provide?
- Under what circumstances would you refer the patient? Where would you refer the patient to?
- Would you recommend any kind of follow-up?

101. The intervention we are testing suggests that health workers at health centre level should detect intestinal schistosomiasis based on whether the area where the patient resides is known to be endemic for the disease and the following symptoms: diarrhoea, anaemia, malnutrition, abdominal pain, jaundice,ascites, intestinal blockage. Do you feel confident that health workers at health centre level can perform these responsibilities?

- If no, why not?
- How would you say the intervention is different from your current practice?
- In your opinion, are there any problems with tasking health workers at health centre level with detecting intestinal schistosomiasis as suggested by the intervention?
- In your opinion, what are the implications of performing this task in terms of time and workload?
- How could the problems you mentioned be addressed? What alternatives or improvements would you suggest?

102. The intervention suggests that an intestinal schistosomiasis diagnosis should be confirmed through a CCA test for patients up to five years or a stool concentration test for patients over five. Are these tests you think can be performed at the health centre?

- If not, why not?

[only probe if participant has indicated their role involves diagnosis of NTDs]

- In your opinion, are there any problems with performing CCA and stool concentration tests at health centre level as suggested by the intervention?
- How could those problems be addressed? What alternatives would you suggest?

103. The intervention suggests that patients diagnosed with intestinal schistosomiasis should receive praziquantel if the CCA or stool concentration tests are positive, including patients up to five years. Patients over five years with any of the symptoms of intestinal schistosomiasis should be given praziquantel presumptively if it is not possible to perform a CCA or stool concentration test. Do you think health workers at health centre level have can perform these responsibilities?

- If no, why not?
- How would you say the intervention is different from your current practice?
- In your opinion, are there any problems with tasking health workers at health centre level with managing intestinal schistosomiasis as suggested by the intervention?

[only use the following probes if participant has indicated their role involves management of NTDs]

- In your opinion, what are the implications of performing this task in terms of time and workload?
- In your experience, are drugs and equipment required to perform this task normally available at this health centre?
- How could the problems you mentioned be addressed? What alternatives or improvements would you suggest?

Soil-transmitted helminth infection

104. Do you know how health workers at health centre level usually detect soil-transmitted helminth infections?

- What signs and symptoms would you look out for?

105. If a patient has signs and symptoms of soil-transmitted helminth infection, do you know how health workers at health centre level usually reach a diagnosis?

[only probe if participant has indicated their role involves diagnosis of NTDs]

- Would you perform any particular clinical examination?
- Would you request a particular laboratory test?

106. If a patient is diagnosed with soil-transmitted helminth infection, do you know how those cases are usually managed at health centre level?

[only probe if participant has indicated their role involves management of NTDs]

- Would you provide any treatment? If yes, what treatment would you provide?
- Under what circumstances would you refer the patient? Where would you refer the patient to?

- Would you recommend any kind of follow-up?

107. **The intervention suggests that health workers at health centre level should detect soil-transmitted helminth infections based on whether the area where the patient resides is known to be endemic for the disease and the following symptoms: worms coming out of mouth/nose/anus, non-malaria fever and vomit, diarrhoea, anaemia, malnutrition, abdominal pain, jaundice, ascites, intestinal blockage, rectal prolapse, finger or nail clubbing. Do you think health workers at health centre level can perform these responsibilities?**
    - If no, why not?
    - How would you say the intervention is different from your current practice?
    - In your opinion, are there any problems with tasking health workers at health centre level with detecting soil-transmitted helminth infections as suggested by the intervention?
    - In your opinion, what are the implications of performing this task in terms of time and workload?
    - How could the problems you mentioned be addressed? What alternatives or improvements would you suggest?

108. **The intervention suggests that soil-transmitted helminth infections should be confirmed through a a stool concentration test. Is this a test you think can be performed at the health centre?**
    - If not, why not?

    [only probe if participant has indicated their role involves diagnosis of NTDs]

    - In your opinion, are there any problems with performing stool concentration tests at health centre level as suggested by the intervention?
    - How could those problems be addressed? What alternatives would you suggest?

109. **The intervention suggests that patients diagnosed with soil-transmitted helminth infection should receive albendazole if the stool concentration test is positive for ascariasis & hookworm infection. Other drugs are recommended if the stool concentration test is positive for other worm infections. Albendazole should be given presumptively if it is not possible to perform a stool concentration test. Do you think health workers at health centre level can perform these responsibilities?**
    - If no, why not?
    - How would you say the intervention is different from your current practice?

    [only use the following probes if participant has indicated their role involves management of NTDs]

    - In your opinion, are there any problems with tasking health workers at health centre level with managing soil-transmitted helminth infections as suggested by the intervention?

- In your opinion, what are the implications of performing this task in terms of time and workload?
- In your experience, are the drugs required to perform this task normally available at this health centre?
- How could the problems you mentioned be addressed? What alternatives or improvements would you suggest?

Lymphatic filariasis

110. Do you know how health workers at health centre level usually detect lymphatic filariasis?

- What signs and symptoms would you look out for?

111. If a patient has signs and symptoms of lymphatic filariasis, do you know how health workers at health centre level usually reach a diagnosis?

[only probe if participant has indicated their role involves diagnosis of NTDs]

- Would you perform any particular clinical examination?

112. If a patient is diagnosed with lymphatic filariasis, do you know how those cases are usually managed at health centre level?

[only probe if participant has indicated their role involves management of NTDs]

- Would you provide any treatment? If yes, what treatment would you provide?
- Under what circumstances would you refer the patient? Where would you refer the patient to?
- Would you recommend any kind of follow-up?

113. The intervention we are testing suggests that health workers at health centre level should detect lymphatic filariasis based on whether the area where the patient resides is known to be endemic for the disease the following symptoms: non-malaria fever and chills and redness of the leg, swelling of a lower limb, pain in a lower limb, elephantiasis. In patients over five years, the disease should also be detected based on the following symptoms: swelling of breast, swelling of an upper limb, swelling of the scrotum or vulva, hydrocele. Do you think health workers at health centre level can perform these responsibilities?

- If no, why not?
- How would you say the intervention is different from your current practice?
- In your opinion, are there any problems with tasking health workers at health centre level with detecting lymphatic filariasis as suggested by the intervention?
- In your opinion, what are the implications of performing this task in terms of time and workload?
- In your experience, are infastructure and equipment required to perform this task normally available at this health centre?
- How could the problems you mentioned be addressed? What alternatives or improvements would you suggest?

114. The intervention suggests that patients diagnosed with acute lymphatic filariasis should receive pain relief with paracetamol, and be advised to hydrate and rest. All patients should be referred to a health post for advise on home management of lymphedoema. Patients with hydrocele should be referred to an appropriate health centre for surgery.Do you think health workers at health centre level can perform these responsibilities?

- If no, why not?
- How would you say the intervention is different from your current practice?

[only probe if participant has indicated their role involves diagnosis of NTDs]

- In your opinion, are there any problems with tasking health workers at health centre level with managing lymphatic filariasis as suggested by the intervention?
- In your opinion, what are the implications of performing this task in terms of time and workload?
- In your experience, are drugs, infastructure and equipment required to perform this task normally available at this health centre?
- How could the problems you mentioned be addressed? What alternatives or improvements would you suggest?

Podoconiosis

115. Do you know how health workers at health centre level usually detect podoconiosis?

- What signs and symptoms would you look out for?

116. If a patient has signs and symptoms of podoconiosis, do you know how health workers at health centre level usually reach a diagnosis?

[only probe if participant has indicated their role involves diagnosis of NTDs]

- Would you perform any particular clinical examination?

117. If a patient is diagnosed with podoconiosis, do you know how those cases are usually managed at health centre level?

[only probe if participant has indicated their role involves management of NTDs]

- Would you provide any treatment? If yes, what treatment would you provide?
- Under what circumstances would you refer the patient? Where would you refer the patient to?
- Would you recommend any kind of follow-up?

118. The intervention we are testing suggests that health workers at health centre level should detect podoconiosis based on whether the area where the patient resides is known to be endemic for the disease the following symptoms: non-malaria fever and chills and redness of the leg, swelling of a lower limb, pain in a lower limb, itching/burning sensation of the skin on leg, enlargement of the foot, plantar oedema, skin nodules, hyperkeratosis, moss-like papillomata, joint fixation. Do you think health workers at health centre level can perform these responsibilities?

- If no, why not?
- How would you say the intervention is different from your current practice?
- In your opinion, what are the implications of performing this task in terms of time and workload?
- In your experience, are infastructure and equipment required to perform this task normally available at this health centre?
- How could the problems you mentioned be addressed? What alternatives or improvements would you suggest?

119. The intervention suggests that patients diagnosed with acute podoconiosis should receive pain relief with paracetamol, and be advised to hydrate and rest. All patients should be referred to a health post for advise on home management of lymphedoema. Do you think health workers at health centre level can perform these responsibilities?

- If no, why not?
- How would you say the intervention is different from your current practice?

[only probe if participant has indicated their role involves diagnosis of NTDs]

- In your opinion, are there any problems with tasking health workers at health centre level with managing podoconiosis as suggested by the intervention?
- In your opinion, what are the implications of performing this task in terms of time and workload?
- In your experience, are drugs, infastructure and equipment required to perform this task normally available at this health centre?
- How could the problems you mentioned be addressed? What alternatives or improvements would you suggest?

D Overall perception of the intervention

120. What do you generally think of the idea of tasking health workers at primary health care level (including health posts and health centres) with detecting, managing, recording and reporting neglected tropical diseases?

- Do you think the primary health care system has the capacity to take on these roles and responsibilities?
- What kind of challenges do you foresee?
- Do you think there would be any challenges with regard to gender? Would women and men face different challenges in accessing care for NTDs at the health centre?
- Do you think there would be any challenges with regard to stigma? Would those affected by disabilities caused by NTDs access care at the health centre?
- What could be done to address those challenges?
- What would your recommendations with regard to integrating the detection, management, recording and reporting of NTDs into primary health care?

121. Is there anything else you would like to share with the research team?

This concludes the interview. Many thanks for taking the time to participate.

Key Informant Interviews – laboratory staff (health centre)

Interview details

**Interview number**				
**Interviewee code**				
**Sex**		Female		Male
**Attended laboratory training?**		Yes		No
**Job title**				
**Name of interviewer**				
**Name of notetaker**				
**Date**				
**Time started**				
**Time finished**				
**Venue**				

Consent

Before starting the interview, informed written consent from the participant must be obtained. Please follow the script provided:

Good morning/ good afternoon

My name is [*name*] and this is my colleague [*names*]. We work for Malaria Consortium, a not-for-profit organisation that specialises in the prevention, control and treatment of malaria and other diseases. In collaboration with the Federal Ministry of Health in Ethiopia, we are currently carrying out a study to explore ways of improving care for a group of infectious diseases called 'neglected tropical diseases' or 'NTDs'. We are testing an intervention that we hope will improve health workers' and Health Extension Workers' ability to detect, manage, record and report those diseases. The intervention has been implmented at your health facility over the last few months and we would like to get your feedback on some aspects of the intervention. Your responses will help us to evaluate the intervention, find out what worked and what didn't and improve the intervention in the future.

All the information you provide will be strictly confidential and your replies will only be shared with the other researchers working on this study. You will be identified by a number, not your name. We have put together an information sheet which summarises the purpose of the interview, why you've been selected and what happens if you agree to participate. Please take a moment to read through the information provided on the sheet.

Do you have any questions about the information provided on this sheet?

Remember that you do not have to take part in the interview if you prefer not to. You do not have to answer questions you don't feel comfortable with and can end the interview at any time. If you agree to participate, we will ask you to sign a consent form. We will audio-record the interview and take notes. This will help us remember what you are telling us. We will destroy the audio-recording once the report for this project has been written. Can I please ask you to read this consent form?

Do you have any questions about the information in this form?

If you consent to participating in the interview, can I please ask you to sign the form?

Thank you very much for agreeing to participate in the interview.

Topic guide

A Training

122. **A few weeks ago, training was offered to health workers from your health centre about nelected tropical diseases. Did you attend this training?**

123. *[If training attended]* **Can you tell me what you learned in this training?**

    - What was the most interesting thing you learned at the training?
    - Do you remember which laboratory test this training covered and which diseases are detected with those tests?
    - What did you like about the training?
    - Was there anything you didn't like about the training?
    - How could the training have been improved?
    - Did you think the training was relevant for your job? Why or why not?
    - After the training, what is your understanding of your role with performing those tests?

124. *[If training not attended]* **Did anyone tell you about this training?**

    - What did others tell you about this training?
    - Do you know what the training was about? Which tests were covered and which diseases are detected with those tests?
    - Do you think the training might have been relevant for your job? Why or why not?
    - Were you told what, according to this training, laboratory staff's role would be with regard to performing those tests?

C Laboratory tests

125. **The training covered two laboratory tests: urinary sedimentation and stool concentration. Do you ever perform those tests?**

    - *[For the tests the interviewee performs]* How often does this happen?
    - *[For the diseases the interviewee does not perform]* Why do you think this is the case?

126. **Do you know how to perform a urine sedimentation test?**

    - Which diseases can be detected with this test?
    - What kind of materials and equipment do you need to perform the test?
    - How much time does it take to perform the test?
    - How difficult is it to perform the test?

127. **The intervention we are testing suggests that laboratory staff at health centre level should use the urine sedimentation test to detect urinary schistosomiasis. Do you feel confident that you have the knowledge and skills to perform this test?**

    - If yes, what gives you the confidence?

- If no, why not?
- Do health workers at this health centre ever request this test? If no, why not?
- If you have performed tests to detect urinary schistosomiasis recently, did you use the urinary sedimentation test? If not, why not and what did you do instead?
- In your opinion, are there any problems with performing urinary sedimentaiton tests at health centre level as suggested by the intervention?
- In your opinion, what are the implications of performing this test in terms of time and workload?
- In your experience, are infastructure and equipment required to perform this test normally available at this health centre?
- Do you think the test results are reliable?
- How could the problems you mentioned be addressed? What alternatives or improvements would you suggest?

128. **The intervention suggests that laboratory staff at health centre level should use the stool concentration test to detect intestinal schistosomiasis in patients over five and and soil-transmitted helminth infections in all patients. Do you feel confident that you have the knowledge and skills to perform these tests?**
    - If yes, what gives you the confidence?
    - If no, why not?
    - Do health workers at this health centre ever request this test? If no, why not?
    - If you have performed tests to detect intestinal schistosomiasis or soil-transmitted helminth infections recently, did you use the stool concentration test? If not, why not and what did you do instead?
    - In your opinion, are there any problems with performing stool concentration tests at health centre level as suggested by the intervention?
    - In your opinion, what are the implications of performing this test in terms of time and workload?
    - In your experience, are infastructure and equipment required to perform this test normally available at this health centre?
    - Do you think the test results are reliable?
    - How could the problems you mentioned be addressed? What alternatives or improvements would you suggest?

129. **The intervention also suggests that laboratory staff at health centre level should use a Circulating Cathodic Antigen (CCA) test to detect intestinal schistosomiasis in patients up to five years. Test kits were provided to the health centre as part of the intervention. Do you feel confident that you have the knowledge and skills to perform this test?**
    - If yes, what gives you the confidence?

- If no, why not?
- Do health workers at this health centre ever request this test? If no, why not?
- If you have performed CCA tests to detect intestinal schistosomiasis, did you use the CCA test? If not, why not and what did you do instead?
- In your opinion, are there any problems with performing CCA tests at health centre level as suggested by the intervention?
- In your opinion, what are the implications of performing this test in terms of time and workload?
- Do you think the test results are reliable?
- How could the problems you mentioned be addressed? What alternatives or improvements would you suggest?

C Overall perception of the intervention

130. What do you generally think of tasking laboratory staff at health centre level with performing tests to detect neglected tropical diseases?

131. And more generally, what do you think about the idea of tasking health workers at primary health care level (including health posts and hospitals) with detecting, managing, recording and reporting neglected tropical diseases?

- Do you think the primary health care system has the capacity to take on these roles and responsibilities?
- What kind of challenges do you foresee?
- Do you think there would be any challenges with regard to gender? Would women and men face different challenges in accessing care for NTDs at the health centre?
- Do you think there would be any challenges with regard to stigma? Would those affected by disabilities caused by NTDs access care at the health centre?
- What could be done to address those challenges?
- What would your recommendations with regard to integrating the detection, management, recording and reporting of NTDs into primary health care?

132. Is there anything else you would like to share with the research team?

This concludes the interview. Many thanks for taking the time to participate.

Health post level

Key Informant Interviews – Health Extension Workers

Interview details

**Interview number**				
**Interviewee code**				
**Sex**		Female		Male
**Attended classroom training?**		Yes		No
**Health Post**				
**Name of interviewer**				
**Name of notetaker**				
**Date**				
**Time started**				
**Time finished**				
**Venue**				

Consent

Good morning/ good afternoon

My name is [*name*] and this is my colleague [*names*]. We work for Malaria Consortium, a not-for-profit organisation that specialises in the prevention, control and treatment of malaria and other diseases. In collaboration with the Federal Ministry of Health in Ethiopia, we are currently carrying out a study to explore ways of improving care for a group of infectious diseases called 'neglected tropical diseases' or 'NTDs'. We are testing an intervention that we hope will improve health workers' and Health Extension Workers' ability to detect, manage, record and report those diseases. The intervention has been implmented at your health facility over the last few months and we would like to get your feedback on some aspects of the intervention. Your responses will help us to evaluate the intervention, find out what worked and what didn't and improve the intervention in the future.

All the information you provide will be strictly confidential and your replies will only be shared with the other researchers working on this study. You will be identified by a number, not your name. We have put together an information sheet which summarises the purpose of the interview, why you've been selected and what happens if you agree to participate. Please take a moment to read through the information provided on the sheet.

Do you have any questions about the information provided on this sheet?

Remember that you do not have to take part in the interview if you prefer not to. You do not have to answer questions you don't feel comfortable with and can end the interview at any time. If you agree to participate, we will ask you to sign a consent form. We will audio-record the interview and take notes. This will help us remember what you are telling us. We will destroy the audio-recording once the report for this project has been written. Can I please ask you to read this consent form?

Do you have any questions about the information in this form?

If you consent to participating in the interview, can I please ask you to sign the form?

Thank you very much for agreeing to participate in the interview.

Topic guide

A Training

133. In May this year, classroom training was offered to Health Extension Workers from your health post about neglected tropical diseases. Did you attend this training?

134. Can you tell me what you learned in this training?

- Do you remember which diseases this training covered?
- What did you like about the training?
- Was there anything you didn't like about the training?
- How could the training have been improved?
- Did you think the training was relevant for your job? Why or why not?

135. After the training, what is your understanding of your role with regard to detecting and managing NTDs?

B Job aids

136. Were you given any materials during or after the training to help you remember what was taught and to help you detect and manage NTDs?

- Did you receive those materials?
- *[if job aids not used]* Could the job aids have been developed in a way that would have made it more likely for you to use them?
- *[if job aids used]* What do you like and dislike about the job aids?
- *[if job aids used]* How could the job aids be improved?

C Disease detection and management

137. The training and the materials that were distributed provide suggestions for how Health Extension Workers should detect and manage six diseases: trachoma, urinary schistosomiasis, intestinal schistosomiasis, soil-transmitted helminth infection, lymphatic filariasis and podoconiosis. Do you ever see patients with signs and symptoms of any of the diseases?

- *[For the diseases the interviewee encounters]* How often does this happen?
- *[For the diseases the interviewee does not encounter]* Why do you think this is the case?

[For the next section of the interview, pick questions relating to two diseases per interview, at least one from each of the following two groups:

1) Group 1: trachoma, lymphatic filariasis, podoconiosis;

2) Group 2: urinary schistosomisis, intestinal schistosomiasis, soil-transmitted helminth infection.

Preference should be given to diseases the interviewee has indicated they encounter in their job.]

Trachoma

138. Do you know how Health Extension Workers should detect trachoma?

- What signs and symptoms would you look out for?

139. If you suspect a patient might have trachoma, what would you do?

- Would you provide any treatment?
- Under what circumstances would you refer the patient? Where would you refer the patient to?
- Would you recommend any kind of follow-up?

140. The intervention we are testing suggests that Health Extension Workers should detect trachoma based on the following symptoms: irritated red eye, mucopurulant discharge or inverted eye lashes. Do you think it is possible for Health Extension Workers to perform these responsibilities?

- If no, why not?
- How would you say the intervention is different from what you normally do?
- In your opinion, are there any problems with tasking Health Extension with detecting trachoma as suggested by the intervention?
- In your opinion, what are the implications of performing this task in terms of time and workload?
- How could the problems you mentioned be addressed? What alternatives or improvements would you suggest?

141. The intervention suggests that patients with signs and symptoms of trachoma should be referred to the health centre for further assessment. Do you think people will follow your advice?

- Why or why not?
- What could be done to ensure people with signs and symptoms of trachoma receive good care?

Urinary schistosomiasis

142. Do you know how Health Extension Workers should detect urinary schistosomiasis?

- What signs and symptoms would you look out for?

143. If you suspect a patient might have urinary schistosomiasis, what would you do?

- Would you provide any treatment?
- Under what circumstances would you refer the patient? Where would you refer the patient to?
- Would you recommend any kind of follow-up?

144. The intervention we are testing suggests that Health Extension Workers detect urinary schistosomiasis based on the following symptoms: in children up to five years: blood in urine;

in children over five and adults: blood in urine, pain while urinating, pelvic pain, genital itching or burning sensation, involuntary urination when coughing, laughing or jumping. Do you think it is possible for Health Extension Workers to perform these responsibilities?

- If no, why not?
- How would you say the intervention is different from what you normally do?
- In your opinion, are there any problems with tasking Health Extension Workers with detecting urinary schistosomiasis as suggested by the intervention?
- In your opinion, what are the implications of performing this task in terms of time and workload?
- How could the problems you mentioned be addressed? What alternatives or improvements would you suggest?

145. **The intervention suggests that patients over five years with blood in the urine should receive praziquantel. All other patients with signs and symptoms should be referred to the health centre for further assessement. Do you think it is possible for Health Extension Workers to perform these responsibilities?**

- If no, why not?
- How would you say the intervention is different from what you normally do?
- In your opinion, are there any problems with tasking Health Extension Workers with managing urinary schistosomiasis as suggested by the intervention?
- In your opinion, what are the implications of performing this task in terms of time and workload?
- In your experience, are drugs and equipment required to perform this task normally available at this health post?
- How could the problems you mentioned be addressed? What alternatives or improvements would you suggest?

Intestinal schistosomiasis

146. Do you know how Health Extension Workers should detect intestinal schistosomiasis?

- What signs and symptoms would you look out for?

147. If you suspect a patient might have intestinal schistosomiasis, what would you do?

- Would you provide any treatment?
- Under what circumstances would you refer the patient? Where would you refer the patient to?
- Would you recommend any kind of follow-up?

148. **The intervention we are testing suggests that Health Extension Workers should detect intestinal schistosomiasis based on whether the area where the patient resides is known to be endemic for the disease and the following symptoms: diarrhoea, anaemia, malnutrition,**

abdominal pain, jaundice, ascites, intestinal blockage. Do you think it is possible for Health Extension Workers to perform these responsibilities?

- If no, why not?
- How would you say the intervention is different from what you normally do?
- In your opinion, are there any problems with tasking Health Extension Workers with detecting intestinal schistosomiasis as suggested by the intervention?
- In your opinion, what are the implications of performing this task in terms of time and workload?
- How could the problems you mentioned be addressed? What alternatives or improvements would you suggest?

149. The intervention suggests that patients with signs and symptoms of intestinal schistosomiasis should be referred to the health centre for further assessment. Patients over five years, except those with jaundice, ascites, intestinal blockage, who state that they do not want to be referred should be given praziquantel presumptively. Do you think it is possible for Health Extension Workers to perform these responsibilities?

- If no, why not?
- How would you say the intervention is different from what you normally do?
- In your opinion, are there any problems with tasking Health Extension Workers with managing intestinal schistosomiasis as suggested by the intervention?
- In your opinion, what are the implications of performing this task in terms of time and workload?
- In your experience, are drugs and equipment required to perform this task normally available at this health post?
- How could the problems you mentioned be addressed? What alternatives or improvements would you suggest?

Soil-transmitted helminth infection

150. Do you know how Health Extension Workers should detect soil-transmitted helminth infections?

- What signs and symptoms would you look out for?

151. If you suspect a patient might have a soil-transmitted helminth infection, what would you do?

- Would you provide any treatment?
- Under what circumstances would you refer the patient? Where would you refer the patient to?
- Would you recommend any kind of follow-up?

152. The intervention suggests that Health Extension Workers should detect soil-transmitted helminth infections based on whether the area where the patient resides is known to be endemic for the disease and the following symptoms: worms coming out of

mouth/nose/anus, non-malaria fever and vomit, diarrhoea, anaemia, malnutrition, abdominal pain, jaundice, ascites, intestinal blockage, rectal prolapse, finger or nail clubbing. Do you think it is possible for Health Extension Workers to perform these responsibilities?

- If no, why not?
- How would you say the intervention is different from what you normally do?
- In your opinion, are there any problems with tasking Health Extension Workers with detecting soil-transmitted helminth infections as suggested by the intervention?
- In your opinion, what are the implications of performing this task in terms of time and workload?
- How could the problems you mentioned be addressed? What alternatives or improvements would you suggest?

153. The intervention suggests that patients with worms coming out of their mouth/nose/anus, as well as patients up to five years with mild abdominal pain should receive albendazole. All otehr patients should be referred to the health centre for further assessment. Patients who state that they do not want to be referred, except those who are in the first trimester of their pregnancy and those with fever and vomit, jaundice, ascites, intestinal blockage and rectal prolapse, should be given albendazole presumptively. Do you think it is possible for Health Extension Workers to perform these responsibilities?

- If no, why not?
- How would you say the intervention is different from what you normally do?
- In your opinion, are there any problems with tasking Health Extension Workers with managing soil-transmitted helminth infections as suggested by the intervention?
- In your opinion, what are the implications of performing this task in terms of time and workload?
- In your experience, are the drugs required to perform this task normally available at this health post?
- How could the problems you mentioned be addressed? What alternatives or improvements would you suggest?

Lymphatic filariasis

154. Do you know how Health Extension Workers should detect lymphatic filariasis?

- What signs and symptoms would you look out for?

155. If you suspect a patient might have lymphatic filariasis, what would you do?

- Would you provide any treatment?
- Under what circumstances would you refer the patient? Where would you refer the patient to?
- Would you recommend any kind of follow-up?

- Do you know how to advise patients on the home management of the disabilities casued by lymphatic filariasis?

156. The intervention we are testing suggests that Health Extension Workers should detect lymphatic filariasis based on whether the area where the patient resides is known to be endemic for the disease the following symptoms: swelling of a lower limb, elephantiasis, swelling of scrotum/vulva, hydrocele. Do you think it is possible for Health Extension Workers to perform these responsibilities?

- If no, why not?
- How would you say the intervention is different from what you normally do?
- In your opinion, are there any problems with tasking Health Extension Workers with detecting lymphatic filariasis as suggested by the intervention?
- In your opinion, what are the implications of performing this task in terms of time and workload?
- How could the problems you mentioned be addressed? What alternatives or improvements would you suggest?

157. The intervention suggests that patients with acute lymphatic filariasis should be referred to the health centre for further assessment. Patients diagnosed with chronic lymphatic filariasis will be referred back from the health centre to the health post, where they will receive guidance on home management of the disabilities caused by the disease. Do you think it is possible for Health Extension Workers to perform these responsibilities?

- If no, why not?
- How would you say the intervention is different from what you normally do?
- In your opinion, are there any problems with tasking Health Extension Workers with managing lymphatic filariasis as suggested by the intervention?
- In your opinion, what are the implications of performing this task in terms of time and workload?
- In your experience, are infastructure and equipment required to perform this task normally available at this health post?
- How could the problems you mentioned be addressed? What alternatives or improvements would you suggest?

Podoconiosis

158. Do you know how Health Extension Workers should detect podoconiosis?

- What signs and symptoms would you look out for?

159. If you suspect a patient might have lymphatic filariasis, what would you do?

- Would you provide any treatment?
- Under what circumstances would you refer the patient? Where would you refer the patient to?

- Would you recommend any kind of follow-up?
- Do you know how to advise patients on the home management of the disabilities casued by podoconiosis?

160. The intervention we are testing suggests that Health Extension Workers should detect podoconiosis based on whether the area where the patient resides is known to be endemic for the disease the following symptoms: swelling of a lower limb, pain in a lower limb, itching/burning sensation of the skin on leg, enlargement of the foot, plantar oedema, skin nodules, hyperkeratosis, moss-like papillomata, joint fixation. Do you think it is possible for Health Extension Workers to perform these responsibilities?

- If no, why not?
- How would you say the intervention is different from what you normally do?
- In your opinion, are there any problems with tasking Health Extension Workers with detecting podoconiosis as suggested by the intervention?
- In your opinion, what are the implications of performing this task in terms of time and workload?
- How could the problems you mentioned be addressed? What alternatives or improvements would you suggest?

161. The intervention suggests that patients with acute podoconiosis should be referred to the health centre for further assessment. Patients diagnosed with chronic podoconiosis will be referred back from the health centre to the health post, where they will receive guidance on home management of the disabilities caused by the disease. Do you think it is possible for Health Extension Workers to perform these responsibilities?

- If no, why not?
- How would you say the intervention is different from what you normally do?
- In your opinion, are there any problems with tasking Health Extension Workers with managing podoconiosis as suggested by the intervention?
- In your opinion, what are the implications of performing this task in terms of time and workload?
- In your experience, are infastructure and equipment required to perform this task normally available at this health post?
- How could the problems you mentioned be addressed? What alternatives or improvements would you suggest?

D Health Development Army

162. The intervention we are testing also assigns certain tasks to the Health Development Army. After the classroom training on neglected tropical diseases for Health Extension Workers, you were asked to inform the Health Development Army of their roles and responsibilities with regard to neglected tropical diseases in this intervention. Did you share this information with the Health Development Army?

- How did you organise this? Was it possible to do this as part of your regular interactions? If not, why not?
- Were there many Health Development Army members with whom the information was not shared? Why?
- How could the process of sharing information about neglected tropical diseases with the Health Development Army have been improved?

163. In your understanding, what is the role of the Health Development Army with regard to neglected tropical diseases?

- In your experience, is this happening in the catchment area of this health post?
- Do you ever see patients who have been encouraged to seek care at the health post by the Health Development Army? How often does this happen?

164. In your experience, what challenges are the Health Development Army facing in detecting signs and symptoms of NTDs and ecouraging people to seek care?

- Do you discuss the challenges the Health Development Army are facing with regard to neglected tropical diseases during your regular interactions with the Health Development Army?
- How could the problems you mentioned be addressed? What alternatives or improvements would you suggest?

165. What do you generally think of the idea of tasking the Health Development Army with detecting people with signs and symptoms of neglected tropical diseases?

- Do you think the Health Development Army has the capacity to take on these roles and responsibilities?
- What kind of challenges do you foresee?
- Do you think there are any challenges with regard to gender? Would women and men face different challenges in interacting with the Health Development Army about NTDs?
- Do you think there would be any challenges with regard to stigma? Would those affected by disabilities caused by NTDs be detected by the Health Development Army?
- What could be done to address those challenges?

E Overall perception of the intervention

166. What do you generally think of the idea of tasking health workers at primary health care level (including health centres and hospitals) with detecting, managing, recording and reporting neglected tropical diseases?

- Do you think the primary health care system has the capacity to take on these roles and responsibilities?
- What kind of challenges do you foresee?
- What are your recommendations with regard to integrating the detection, management, recording and reporting of NTDs into primary health care?

167. Is there anything else you would like to share with the research team?

This concludes the interview. Many thanks for taking the time to participate.
