# Supplementary material for: Gender-related factors affecting health seeking for neglected tropical diseases: findings from a qualitative study in Ethiopia
Source: PLoS Negl Trop Dis. 2019 Dec 12;13(12):e0007840. doi: 10.1371/journal.pntd.0007840 (PMC6907747; doi:10.1371/journal.pntd.0007840)
Supplement: S4 Appendix — (PDF) [file pntd.0007840.s004.pdf]

S4 Appendix. Raw interview and focus group discussion data

FGD transcripts

FGD 1

R: In May this year, Health Extension Workers and the Health Development Army in this area were trained on neglected tropical diseases as part of the intervention we are testing. Are you aware of the roles and responsibilites with regard to neglected tropical diseases the intervention assigns to Health Extension Workers and the Health Development Army?

G12: No, I 'm not aware of a training for Health Extension Workers and the Health Development Army. What was the training about? Even me, I'd like to be trained. **[all laugh]**

G14: I'm also not aware of the training because nobody told me.

G16: In my village, I heard about the training from one of the HDA leaders. Moreover, I even observed certain papers in her hand and when I asked her, " What are those papers?", she told me, "The paper is a reporting form, in wich I record the people who have diseases." Then when I asked her, "Why do you record this?", she replied to me, "The Health Extension Worker ordered me to cellect the data."

G13: My wife is pregnant, so when the Health Extension Worker came to my house, she gave her certain papers and I also heard that she told he, "Please register the peaple." Actually, I don't know what is to be registered.

R: So who is aware of the training and who is not?

[Four participants stated that they knew about the training and six said that they were not aware. The researchers then read out the NTD fact sheet which contained pictures of signs and symptoms of NTDs taken from the pictorial tool developed for the Health Development Army.]

R: Do you know some of the neglected tropical diseases that Health Extension Workers and the Health Development Army should cover as part of this intervention?

G17: Yes, I know about some of the diseases like Daniichu Xibba **[literally "the disease of the elephant"]**. *I also know about a disease that makes the legs swell for men and a disease that makes women have swollen breasts.*

G15: I am aware of the disease that can be tranmitted through soil and the disease that affects the eye called trachoma. As I am a school teacher, I know that school children sometimes miss class complaining about a disease that affects their abdomen, which makes them have diarrhoea. I heard about this disease from the Health Extension Worker.

G20: I heard about the disease called trachoma. As you know, most of the time children who do not keep their face and eyes clean are affecteded by trachoma. In our community, I see that the people who do not wash their faces are children from poor families.

G18: I know about the disease that affects the genital area.

R: What do you call this disease that affects the genital area?

G18: A disease that affects the penis and sometimes the scrotum.

G17: I heard that the disease that affects women's womb makes the women in fertile. The disease is transmited by a snail that is found in the water. When people come into contact with the water containing the snail and the snail enters the body through people's skin, it causes men to urinate red urine and women to be infertile.

R: What is your understanding of the roles and responsibilities with regard to neglected tropical diseases of Health Extension Workers?

G20: In our village there are 39 Health Development Army members. They all need help from the Health Extension Worker. As you know, even we **[participant represents kebele administration]** *have responsiblity to help the Health Development Army and Health Extension Workers. So with regard to the question you asked, each Health Extension Worker has the responsiblity to teach about 500 households found in each catchment area. So I know that Health Extension Workers teach the community about nutrition, sanitation, latrin use – and also, now they teach the Health Development Army and the households about those negleceted diseases.*

G14: Health Extension Workers teach community memebers about the neglected diseases by gatherining all 1-to-5 leaders in a meeting. They also share knowledge by gathering all the 39 health Development Army leaders in the meeting they have every month. As you know, those who acquired knowledge during this meeting, can simply share the knowledge with other people through their networks. For example, when a Health Development Army leader gets knowldge from the Health Extension Worker, she is responsible for teaching or raising awareness among at least six 1-to-5 leaders. At the same time, one trained 1-to-5 leader can share the same knowldge with five people in her team. This is the way knowldge is shared in our community.

R: What is your understanding of the roles and responsibilities with regard to neglected tropical diseases of the Health Development Army?

G15: In our community, health Development Army leaders moblise the community about sanitation and hygiene issues and also sometimes they gather people to clean their neighbourhoods.

R: Those are all good points you are raising, but what we want to discuss are the roles and responsblities of the Health Development Army leaders regarding the control of neglected tropical diseases.

G15: Okay, when there is something new, they create awareness among the community.

G12: In our community, Health Development Army leaders gather women and teach about sanitation and nutrition, children's nutrition -- they visit households to teach what they understood to each other.

G20: As we know, Health Development Army leaders are community members, so they have frequent contact with all the peaple who live around them. They meet people in different meetings, like women's religious meetings every Wednesday called "hamusse", or *when they are having coffee together in a household, they create awareness among all households gathered there.*

G13: As you know, health Extension Workers only work in the community certain limited hours, but Health Development Army leaders live in the community. Therefore, they teach community members all the time. They live in the community day and night, so they have a very good opportunity to teach about the neglected diseases. But I fear that not all Health Development Army members share the knowledge equally because some are motivated to share, but some are not. As they are not paid by the goverment, unless there is something that motivates them, they may not teach others as expected. But those who are motivated and enjoy volunteering can teach the community about the disease you **[the researchers]** *and the government are concerned about.*

R: Do you ever see people in your community with signs and symptoms of those diseases?

G16: Yes, I saw this disaese, even though it is not common now in our community -- I saw the one that affects young boys and making them urinate. It is usually calded syphilis. It is also a kind of disease that can be transmitted when young boys have sexual intercousrse with multiple partners. I also observed on the pictures you showed us a disease that makes the belly swell. This disease can be transmitted when a person eats food without washing hands and with dirty fingers.

G20: I also saw a child with puffy face and children with eye disase. I think this disease can be caused when a child does not have sufficient food. People who lack sufficient food can develop eye disease and their face looks like an old man's face.

G11: You know, most of the time, the diseases that affect our children are diseases that cause belly cramps and stomach pain, which make children have diarrhoea or vomit. Even my own children are affeted by these diseases very often.

G14: Yes, the disease that affects all people all time is diarrhoea. In addition, in my community, there is a woman with an enlarged abdomen. Actulally, I don't know the name of the disease.

R: Thank you, let's again have a look at the pictures and select two diseases that are common in your community.

G12: My son **[referring to the researcher]***, actually I did not see that disase on the paper you gave us, but the disease that affects all age groups in our community is -- I don't know the name, but in our language it is called "Fullaticho"* **[boils]***, a disease that makes certain body parts swell and finally the affected parts contain pus and cause people a lot of pain. In the health centres, the care and treatment are not so good. Also, culturally, it causes the affected people to stay at home and eat friuts and vegetables until finally the pus comes out. This is the disease that affects our community most. Why is there no good treatment for this disease at the health center? Also, there is a disease that affects women's breast, which is called "Gamitoke" in our local language* **[a disease that affects the breast after child birth]***. Culturally, it is treated by making the affected woman chew certain leaves of a tree, which make the disease disappear. This is also a disease that is not talked about much, but that affects our women.*

G20: There is also a disease called "ATET" **[acute watery diarrhoea]***, which affects our people every year.*

G17: There is also a disease that affects the eye. Actually this disease affects children rather than older persons and it also affects poor peaple.

R: Why does it affect the poor?

G17: Because they don't get clean water.

[The participants eventually agreed to discuss urinary schistosomiasis in more detail.]

R: Do you think the Health Development Army has the capacity to detect people with those signs and symptoms in their communities?

G16: Yes, they can identtfy those cases. You know why? Once they have understood the signs and symptoms of the disease, they can share information with others easily through the 1-to-5 networks.

G14: Yes, they can. I think I myself could now identfy the sign and symptoms of the disease **[having discussed them with the group and looked at the fact sheet]**. *As you know, the diseases exist in the community. The only thing that's needed is the motivation of individuals to teach others, because they are simple to identify. Our community talks about a lot of things when they have coffee together, so if this information is shared and discussed at those events, it is easy to create awarrness.*

G12: You know, there are people who are affected by urinary diseases. A lot of people are affected by those diseases and some even had surgery. Even I am affected by this disease. Sometimes when I urinate, my urine is interrupted and sometimes the colour of my urine changes. You see, the disease very much exists in the community, but no one dares to speak frankly about it. People do not talk about it with others, especially when the disease is related to the genital area. They hide the disease from the people around them.

R: Why do you think people are afraid of speaking frankly with other people?

G12: I think people are afraid and they can only talk about the disease when they are at the hospital. So I'm worried that if the person affected by those diseases is a man, they will not tell the **[female]** *Health Development Army member and she won't be able to identify those cases easily.*

G15: I also think that it is challenging for the Health Development Army to idenfy diseases affecting men's genital area, so I recommend that men should teach and create awareness of this kind of diseases among men. Culturally, men do not disclose this kind of disease to women.

G12: Sorry for my interuption. You know, as I told you earlier, it is challenging to detect this kind of disease through the Health Development Army. However, if the Health Development

Army create awareness in the prescence and the support from you **[Malaria Consortium]***, they can detect those patients.*

R: What kind of support?

G12: For example, if there is **[financial]** *support for treatment for those diseases, people can accept the teaching from the Health Development Army***.**

G17: You know, in our community people call the disease that makes their urine red "Magarto" **[literally "the disease that is transmitted by bats]***, so the community may not know that the disease is actually caused by other factors. Therefore, I think what is needed is to teach the community about the disease. I think, in the future, if the community starts to discuss about this issue, then Health Development Army leaders can identify those cases. But for now, I don't think they can, because those diseases have different meanings in the community.*

R: In your opinion, are there any problems with tasking the Health Development Army with detecting urinary schistosomiasis as suggested by the intervention?

G20: I don't think they will face any challneges in detecting this disease. The Health Development Army are used to multi –tasking. They have the ability to reach the community, like the police. They can reach every case in the community. In addition, there are 22 1-to-5 networks comprsing males **[an administrative network not related to the female 1-to-5 networks that are part of the Health Development Army structure]***, so it is possible to create awareness using the male team members we have in the village.*

G15: I think they can detect this disease without difficulty. You know why? For example, if the Health Development Army teach me or other men, it is possible to share that knowledge with other persons. She can get information about those cases from me or other men who are close to her. To make detection more sucessful, our male involvement is required, because culturally, females cannot reach out directly to men.

[Participants then agreed to discuss group of signs and symptoms relating to intestinal schistosomiasis and soil-transmitted helminth infection in more detail.]

R: Do you think the Health Development Army has the capacity to detect people with those signs and symptoms in their communities?

G11: Yes, they can detect this disease. I think this is simpler than the disease we discussed earlier because the signs are common in the village where we live and our mothers have a habit of talking about their children's problems openly with everyone. Therefore, the information can be shared between people and the Health Development Army leaders can identify the disease. This diesease is common and peaple are not afraid to talk about it. The Health Development Army can screen the community without any challenges.
G15: Yes, they can detec this disease. You know, the disease is more common among children than among adults, so detecting and approaching households to identfy this disase is simple. In addition, the signs and symptoms to identify this disease are easy to spot. Even illiterate people can identify the signs and the symptoms.
G20: Yes, they can identfy the disease, but the signs and symptoms are similar to those of diseases caused by bacteria and also they look like a disease of poor sanitation and hygiene, so I think to make it easier for the Health Development Army, you need to train the Health Development Army leaders.
G17: Also, to make it easier for the Health Development Army, they and the Health Extension Workers should move through the village together. As you know, the health Extension Workers are more respected than the Health Development Army. It would be better if the health Extension Workers visited households together with the Health Development Army.
G14: Yes, they can detect this disease, but not all of them. As you know, the understanding of the disease can be different among Health Development Army leaders. There are some very educated and active women among the leaders. On the other hand, there are also some with medium knowledge, so it would be better to task the active leaders with identfying those diseases in the community. Sometimes, some of those who are tasked with this may not be able to idetfy the disease in the community and may find it challenging to share knowledge with others because they themselves may have a knowledge gap.
G16: Sometimes the community may also not respect and receive the Health Development Army's advice very well.

R: Why do you think households may not accept the advice they get from the Health Development Army?

G16: As you know, the Health Development Army leaders and the households live in the same village and they know each other, so some households may not appriaciate the knowldge the Health Development Army. They know each other, so for that reason,

households may not tell their problems to the Health Development Army leaders and detection of the diseases may be difficult for the Health Development Army.

G17: To make disease detection easier, you need to create awareness among all community members through the Health Extension Workers before you task the Health Development Army with detecting those diseases. Then the Health Development Army will be welcomed easily when they reach out to each household to detect the diseases.

R: Is there anything else you would like to share with the research team?

G16: Actually, I think we are now also more aware of some aspects of those diseases and hence we also need to support the Health Development Army and Health Extension Workers in informing people who suffer from those diseases.

G13: As we discussed, there are diseases that are difficult for female Health Development Army members to detect, so as my friend said, we have to help the Health Development Army in detecting those diseases.

G17: You know, female Health Development Army leaders may find it chakllenging to teach and detect the diseases that affect the male sex organs. I am absolutely sure that they are even afraid of naming those diseases that the males have.

R: For example what kind of disease will the Health Development Army find difficult to name?

G17: For example, they will be afraid to name the disease of the scrotum because in our culture, if the female names this disease frankly, she will be considered an ill-mannered woman.

R: This concludes the ifocus group. Many thanks for taking the time to participate.

FGD 2

R: First of all, I would like to thank you for volunteering to take part in this discussion. The intervention we are testing assigns certain tasks to Health Extension Workers and the Health Development Army. We are talking about neglected tropical diseases, a group of diseases that receive little focus, which Health Extenion Workers and the Health

Development Army have been dealing with. Do you have regular meetings or discussions with Health Extenion Workers and the Health Development Army?

G03: Yes, we do.

R: Are your meetings regular?

G03: Yes.

G06: Yes, we interact regularly.

R: How often?

G06: Every 15 days.

R: With whom?

G06: With the Health Extension Workers. We have meetings here **[at the health post]** *where she calls us to discuss, but* **[name of Health Extension Worker]** *had an accident. For this reason, we missed one or two meetings. Now she is well again, we've had one meeting. The next meeting has been postponed due to a Muslim holiday. So we came back without having had a meeting. But we have a meeting with the Health Extension Worker every 15 days.*

R: When you meet, what do you talk about? What issues are discussed?

G06: We discuss about bringing pregant women **[to the health post]**. *We also discussed how I broght a pregnant woman and she received services for pregnant women. The Health Extension Worker also had a leaflet that was important to read. The leaflet shows a pregnant mother that says "No mother should die while giving birth". So we receive education and awareness raising about not giving birth at home but rather at the health facility. I even broght a pregnant mother from the market. I advised her about the importance of giving birth at a health facility, so I brought her to* **[name of health centre]** *where she gave birth safely after a long time. She did not need surgery but gave birth naturally. They also have the phone number to call an ambulance.*

R: You are talking about the importance of giving birth at the health centre?

G06: Yes, they save their lives.

R: What other issues do you disscuss with Health Extension Workers?

G04: We also discuss about our health. For example, if someone doesn't know how to clean cooking utensils, she will show them how to wash dishes. She knows about diseases like typhoid, which is caused by not maintaining good personal hygiene. Or washing hands after

using the toilet. We get such rich information from her. When we go home, we act according to the information we received.

R: What else? Can other parcipants tell me more?

G05: Another issue we discussed is that during the rainy season there may be stagnant water bodies around us.We try to dry the environment, because the mosquitoes that transmit malaria breed in that water. And we also had a discussion about using latrines. And we learned how to use a bednet. We even followed up about who had a bednet and uses it properly. This is what we discuss every 15 days.

R: What else?

G02: We learned about blood tests for HIV/AIDS and how to avoid becoming infected. They taught us that a blood test should be done before getting married and people should avoid unwanted environments where they are exposed to HIV/AIDS. So protect your life from bad things and take care of your family.

G07: Most points have been mentioned now, but we also learn about harmful traditional practices, like female genital cutting. We go back to the community to check on such things. Another thing is the transmission of tuberculosis. And handwashing with soap after using the toilet. Or if you don't have soap, wash your hand with ash.

R: Good, so this is what you discuss with the Health Extension Worker. What do you discuss with the Health Development Army? Do you have regular meetings?

[All respondents agree that they do.]

G03: Yes, we have regular meetings. We met with the network of 30 households and we discussed about children's hygiene and about washing hands after using the toilet. If they do not wash their hands, that may cause eye disease. And if we don't prepare water with soap for the children, that causes abdominal problems and the anus my protrude to the outside of the body.

R: What else can other tell us? What do you discuss with the Health Development Army?

G07: We meet near our homes with the 1-to-5 network and the 30-household network. We talk about our lives and then, as my friend said, we learned about handwashing and cutting our nails to prevent dirt entering our mouths via the hand. And we accept that advice.

G03: We learned about cholera -- the causes of cholera, handwashing, taking care of food hygiene and so on.

G05: We talk about hygiene and also about mothers delivering at the health facility, immunisation and antnatal follow-up. And we are even responsible for providing the phone number to call an ambulance.

R: What are Health Extension Workers' main roles and responsibilities with regard to the Health Development Army?

G06: Their main roles are – previously, people did not use family planning. They used to have a child now and then without spacing the next one. Now the Health Extension Workers provide family planning for us. We use long-acting and short-acting family planning. They also give health education about immunisation in the first 45 days of a child's life. And if there is a mass drug administration campaign, they deliver the drugs to the community accordingly. They also visit homes and do blood tests for diseases.

G03: They visit homes to check who has not had the immunisation. They also call meetings to discuss our health and they lead the pregnant mothers' forum.

R: How would you describe the main roles and responsibilities of the Health Development Army?

G03: We work with the community, for example those who are pregnant and need antenatal care. We encourage them to come to the health post. We teach the communtiy to have their children immunised and we also advise them if they have water stored that exposes them to the mosquitoes that cause malaria.

G01: We are responsible for the distribution of bednets, becasue some may not receive them. We help Health Extension workers during the distribution after they have been registered.

R: Do people in your communities use the services of Health Extension Workers the Health Development Army?

[All participants agree that people use the services.]

G04: These days, people in the community have good awareness of their health. For example, they know about epidemics, can identify the causes for the epidemic early and teach the community about the cases that happen by going from home to home. And also, they appreciate those who are in the early stages of pregnancy, which in our culture is a taboo, but the Health Extension Workers ask about this during their home visits. Our people are using the services given by the Health Development Army and Health Extension Workers.

R: Is there anyone in the community who does not use the services given by the Health Development Army and the Health Extension Workers?

G04: No, everybody is using the services these days.

R: Does the community have a positive attitude towards the services given by the Health Development Army and the Health Extension Workers? What they are saying?

G04: Maybe some people said that the drugs given for the different types of family planning have negative side effects. They ask how long this family planning effect will last or if it casues infertility. So we send them to the Health Extension Worker about those questions they had. Mostly, they complain about the impact on their menstruation cycle.

[Several participants responded that in general, communtiy members have a positive attitude to the services provided.]

R: In May this year, Health Extension Workers and the Health Development Army in this area were trained on neglected tropical diseases as part of the intervention we are testing. Are you aware of the roles and responsibilites with regard to neglected tropical diseases the intervention assigns to Health Extension Workers and the Health Development Army?

[Most of the participants said that they had not heard of the training, but some indicated that they had.]

G03: I have received a leaflet with pictures of neglected tropical diseases. They gave me the leaflet and asked me to record those who have swollen legs. I recorded a case of a man who has swollen legs and reported him to the Health Extension Worker.

R: Do you know some of the neglected tropical diseases that Health Extension Workers and the Health Development Army should cover as part of this intervention?

G05: Yes, they brought us thos ematerials which are important for identifying cases which are shown in the pictures.

[Four participants said that they knew of the pictorial tool for the Health Development Army. Some had brought it along to the focus group discussion.]

R: What is your understanding of Health Extension Workers' roles and responsibilities with regard to neglected tropical diseases?

G04:I think they are identifying those with intestinal problems that cause diarrhoea, mothers who have a prolapse of the uterus or vagina and for men, those who have swollen testicles. They are responsible for referring those cases, but they are difficult to manage at health post level. They tried to help those cases, but the people who have those problems did not wait for the services at the health post. The patients tried to get treatment for their problems rather than expecting help from the health post. No one had a solution for those cases at the health post, but they have those cases. Their health is not good.

HDA: G03: Our understanding is that we identify the cases that have the same symptoms as in the pictures and refer them to the health post so they can be further identified and receive services for their disease. The Health Extension Worker told us to record the cases we find.

R: How do know if there is a case?

G03: I know who has those neglected problems. I can identify them through the 1-to-5 network with my neighbours. Sometimes, we hear about those cases during coffee ceremonies. I try to ask the person who has those problems and I record and report them to the health post. Our role is just to record and report them.

R: The intervention we are testing suggests that the Health Development Army should detect people with signs and symptoms of five diseases: trachoma, schistosomiasis, soil-transmitted helminth infection, lymphatic filariasis and podoconiosis. People who have those signs and symptoms should be encouraged to seek care at the health post, where they can be further assessed and either receive care or be referred to a health centre. Let's look at each of the diseases in more detail. We have prepared a fact sheet that provides some information about each of the diseases.

What is the first picture shows? All the participant said about eye problems.

[Participants spend several minutes reading through the fact sheet.]

R: Do you ever see people in your community with signs and symptoms of trachoma? How common is this?

G03: Yes, there is a child in my communtiy who has eye problems. His eye was swollen and I sent him to the health post. They referred him to the hospital and he is better now. There is also a woman who has eye problems. Her eyes are burning and there is a man with eye problems whose vision is blurred sometimes – it is on and off. So no one is blind in my community, but there are a lot of problems.

G08: In my neighbourhood, there is a woman with eye problems. Her eye is red, she bleeds from the eye, her head aches and her eye lashes are broken. She is very concerned about her problems.

G01: There is an old man in my neighbourhood. His eye looks like she can see, but it became worse and now he can't see anything. He tried different health facilities and even had surgery, but there was no cure for his eye and he became blind.

G06: There is a mother who had eye problems who always had eye **[unintelligable]**. *Slowly, the problem worsened and she finally became blind. There is also an old man who has eye problems. He cannot see objects in the distance. He tried to find a solution, but he is still waiting for surgery at a missionary health facility*

R: So there are many people in your neighbourhoods who have those sign and syptoms?

[All the participant agreed that this is commn.]

R: Do you think the Health Development Army has the capacity to detect people with those signs and symptoms in their communities?

G02: No, they cannot identify.

R: Why not?

G02: They did not have enough knowledge. It depends on their education level.

R: Why?

G05: Because this kind of education only starts now. In the past, there was no such education for them. We only heard about it from you.

R: Are there any other problems?

[All participants agreed that the only problem was lack of education and training.]

R: In your opinion, are there any problems with tasking the Health Development Army with detecting trachoma as suggested by the intervention?

G02: It's just the lack of education. If they educate us, we can be responsible for identifying and reporting. There are a lot of problems in our community.

R: Let's talk about schistosomiasis now. Can you identify cases of schistosomiasis?

Go1: We can see and ask children who have problems urinating, but it is difficult for us to ask adults if they have problems urinating.

G02: Those body parts are not seen. They are hidden. The question is, how can we ask them?

G03: This picture shows a man's swollen sex organ. And you men, you keep that swollen thing inside your trousers. So how can we ask him? I think this will be difficult.

R:Have you tried asking people by showing this piture [shows picture in the pictorial tool]?

G04: It will be very difficult to ask people who have a swelling in that area.

G05: If a woman has a swollen breast, we can ask her about her pain during our coffee ceremonies.

G06: For example, if people have pain in the breast area, people may not understand. We sleep during the night and wake up early in the morning. It is not understood by our husbands and, as you said before, this pain increases slowly because we are working here and there without complaining, so people may not understand. For those who have breast pain or pain in the uterus, the government says – it is difficult to ask those who have swollen sex organs. For the others, we can ask them what kind of pain they have because the breast area is visible and also the abdomen is visible.

R: In this area, do children have worm infections and vomiting?

[All participants agree that this is common.]

R: Which is more common -- those who have worms and abdominal swelling or those with swelling of the breast? Which do people complain aboout more in your area?

G04: Those with swelling of the legs is more of a problem.

R: You see more people with swollen legs?

G04: Yes.

G03: We are talking about our area. For example, in **[name of area]** *there are many people who complain of breast pain. They must have support after they have been informed of this. We must look everywhere, not just focus on one area. We must look in all directions if there are cases.*

R: You said you see swollen legs in your area. What signs and symptoms have you seen?

G03: We have seen swollen legs.

R: There are different diseases that cause leg swelling. Some are communcable and some non-communicable. The communicable one is caused by flies like malaria is caused by mosquitoes. After a certain time, it can cause the legs to swell up and after a long time, hands and legs will also cause problems and the male scrotum can also swell up or the female breast. Have you seen these signs and symptoms in your area?

G08: Yes, these cases exist in my area.

G01: In my area as well. It can spread from one family member to another. In one family, there are four individuals with swellings and in another there are two. The disease can spread within the family.

R: Which body parts are affected?

G01: The legs.

G07: In my area also, one mother and her daughter have swollen legs.

R: So a mother and her child have swellins. Have any of the others seen those who have swelling of the male genital area?

G: No, we haven't seen swellings of the genital area. But there is a mother whose eye has become blind.

R: You have not seen swelling of the breast?

[Several particpants stated that they had.]

R: So can you send these cases to the health facility immediately?

G: No, that's not possible.

R: Because those diseases are hidden?

G: Yes, that's right.

R: If it was malaria, you could send them to the health center?

G: Yes.

FGD 3

R1: The intervention we are testing assigns certain tasks to health Extension Workers and the Health Development Army. Do you interact regularly with Health Extension Workers?

E07: Yes, we regularly interact with Health Extension Workers every fifty days to discuss about differrent issues **[some participants indicated that they disagree with this statement, especially that they interact every 50 days].**

R: What are those different issues you discuss?

E07: Like, about hand washing practice, dish washing and construction of the latrine.

E09: Yes, we gather for a meeting every fifty days to discuss with Health Extension Workers, but there are also times when we do not discuss due to different situations and reasons. When we come together, the Health Extension Workers teach us about all 16 health extension packages, like about nutrition, personal hygine and others.

*E010: Let me tell you a story. Our peaple say: "telling truth does not kill a person who speaks truth". **T**o tell you frankly sister* **[name of Healthe Etension Worker]** *is my role model, because I remember once upon a time, she arrived at my home with an injured leg wich was bleeding because she had injured a toe nail. She came to give me the registration book wich I use to record children who were not immunised. Hence I learned that she is the woman God gave us. Sometimes she teaches us about money management, beyond teaching on health issues.*

What are Health Extension Workers' main roles and responsibilities with regard to the Health Development Army?

E08: They teach us about immunization of children and mothers.

R: What do they tell you about the immunization?

E08: They teach us all children have to be immunised with all the 11 immunizations that are reqired for the child. All the immunizations have to be finished when the children are at nine months old. I forgot many things they teach us but these are the few I remember.

E06: They visit each household and teach us not to give birth at home and during pregenacy, to follow the antenatal care.

E09: Health Extension Workers also teach us about female genital mutiltion, telling us not to circumsize your daughter.

E04: She teaches us how to transfer knowldge we have to other households. Also, we are the the bridge **[laughter among participants]** *to pass all the information to the households as*

well. Yes, I am right – to moblise and teach about what he wants, when she wants to gather every household, she has to pass through me.

E07: In adddition, she treats patients at the health post. You know, we like **[name of Health Extension Worker]** *because she is always with us. When we want her, we can get her easily and get advice from her for any issue we may have.*

E06: She also gives immunization for our children free of cost.

E08: *As we are the leaders of the 30 households* **[Health Development Army leaders are responsible for 30 households]***, we are reoponsible for teaching other households in our network to implement the health extension packages. Especially we teach the peaple in our network to construct latrines. Also, if I find a pregnant mother in my network, I advice her to go to health center for antenatal care. And I have six 1-to-5 networks* **[the functional unit of the Health Development Army]** *under me and hence I teach the 1-to-5 leaders as well to implement the health extension packages.*

E05: Health Extension Workers advise households to use long acting family planning and to follow the antenatal care at health facilty.

E010: They vist the latrines that are found at the back of the our housholds. Also, sometimes when we find it difficult to convince peaple to implement certain packages of the health extension programme, they vist households with us. You know, previously pregnant woman were dying at their home when they faced emergency labour, but now according to what the Health Extension Worker taught us, we distributed a mobile phone number of a car wich was prepared to transport pregnat mothers to the health facility when they face emergency labour. They call the the car wich is ready to pick such women. They are using the ambulance car and that saves their life.

E05: She teaches us about family health using the book wich is coulourful -- Sorry I forgot the exact name of the book.

E07: The family health guide.

E05: Yes, that is the name of the book she uses to teach us. And also, we use the book to teach our memebers when we vist their households.

R: Do health Extension Workers sometimes provide training or supervision to the Health Development Army? How is this typically organised?

E03: Health Extension Workers provide training for us when they are ordered by higher officials, but also, sometimes they teach us when they get new information from the Ministry of Health health ministry.

R: Do Health Extension Workers provide you supervision or training regularly?

P: They do not give us traininig regularly, but sometimes, when they are ordered from somebody higher than them, they train us by gathering us in one place. But more often, they communicate us the information at regular meetings we have.

R: What kind of meetings do you have wich help you to contact Healthe Extension Workers?

We have a pregnant mother confenrence monthly. Also, the HDA meeting every fifty days and they communicate with us when we go to the health post to report certain things we have.

E02: She has contact with us regularly. As she is with us, we can have meetings wich are not regular. She even teaches us along the way.

R: In May this year, classroom training was offered to Health Extension Workers from your health post about neglected tropical diseases. The Health Extension Workers were tasked with sharing information about those diseases with the Health Development Army. Did you receive this information from a Health Extension Worker?

E08: Yes, she taught us something using pictures.

R: What is it you learned or the Health Extension Worker told you?

E08: She taught us about the disease wich affects the womb of the mother, the scrotum disease of the male **[laughter among participants]***, about swelling of the leg, peaple who vomit and a disease which makes the person have bloody diarrhoea. Those were diseases she taught us about using the picture. Also, she told us to please teach the households in our 1-to-5 networks and to put a mark when you get people with signs and symptoms of the diseases. Additionally, she gave us a six-month reporting format and she told us to report monthly the disease we may find in the community. Already we delivered the January report for her. If there is anything I missed, others may add.*

E01: To add something not mentioned before, she taught us about a disease wich makes the eyes not see clearly, people whose defecation contains worms. Accordingly, I identified four peaple from my village and reported them to the Health Extension Worker.

R: How was this organised? Was it part of your regular interactions with Health Extension Workers?

E07: I dont know how the Health Extension Worker organised the training for others, but for some of us, she arranged a place in our village and taught us about the the diseases in the village we live. I think we were six in number. **[other participants agreed that this was how they were trained]**

R: How could the process of sharing information about neglected tropical diseases with the Health Development Army have been improved?

E09: You know, there are 1-to-30 and 1-to-5 networks in every village, hence I think to make information sharing stronger, we have to strenthen the discuusion we may have in our networks. As you know, people in our community hide diseases they have from other people, hence unless we talk about the disease in public, in the meetings we have among the HDA and in 1-to-5 network discusions, people will continue to not disclose the disease.

E03: I think what also motivates us is when people we detected get treated and cured from the disease they had. Unless the people we are refering get treatment for the disease, people we refer and teach may not come to the health facility.

R: Why do you think people may not come to the health facility?

P: My son **[referring to the researcher]***, most of the people in their home know about their disease, but they are still not going to the health facility because they are poor. They do not have money to pay for the teatment. If your organisation has support to treat such people, other people who have not disclosed their disease can visit the health facility. Even we are motivated when people we refer are treated and get cured, and hence your organisation must support those people.*

R: What kind of support do you expect?

P: At least treatment expenses. People appriciate the service when they get treatment and a cure for their disease. For example, if a person with eye disease gets eye drop drugs for free from you and they get cured, others may see that and appreciate it and vist the health facility.

E05: I also support her idea. For example, I taught two people in my village and reported that to a Health Extension Worker, but those who see me registering those people expect something fom me. For example, if the peaple I selected get treatment and cured that attracts people to the services and makes the community respect you and believe in our work. Unless those peaple get treatment, our acceptance from the community will be low and the people we teach will not attend the health facility. You know, peaple disseminate information easily when there is something new, but unless treatment is given free of cost, people may not appreciate the service.

E02: To add to this, more people can be aware when the meeting is organised. Training has to be organised for all people under the 1-to-5 networks. The same training we got from the Health Extension Worker has to be delivered to all because the information can be easily transfered to all in a short period of time. **[There was agreement among the group about this suggestion.]**

E03: As you know, the government has already prepared a transportation car free of cost for the pregnant mothers. Similar to that, you have to arrange cost-free services for the people who have deseases. Previously, pregnant mothers were mentioning lack of transportation made them not give birth at the health center, but now, every pregnant mother can call the car and go to the health facility. Hence you have to arrange this for our community. **[general agreement]**

E04: Thank you, let me tell you one thing I experienced. Last week, I was in a household to teach and detect the persons who have those diseases. When I reached the home, one child was ill in his eye, His eyes were full of tears. When I observed the child and I told the mother to bring him to the health facility, the mother asked me, "Is their tretment free?" I said, "No". Then she replied that she knows that her child has a problem, but real problem is "Birr" **[name of Ethiopia's currency]**. *"I dont have the money to treat my child." You see, our community problem is not only knowledge, but also they seek free treatment as they are poor. That's why I reccomend to arrange the treatment for the cases we refer. Unless the people get teatment,*

they do not accept our teaching and consider our detection and registration as something that is useless.

R: Can you tell me what you learned about neglected tropical diseases?

E04: Glory to the Lord, the government is facilitating everytime meetings for us to to gain knowledge. You know, sight is basic for human beings, and I learned a lot from the training about this eye disease wich I did not give attention before. You know, in our community a lot people lost their eye sight, hesitating to seek health care at the health facilty and considering the disease as a simple thing. I know people who had surgery at hopital, but were not cured because they went to the hospital only after they lost their eye sight. So I learned that early tretment is good for our eyes.

E09: What made me happy was, as I said earlier, that the dieseases we learned about are not common. Even though those diseases are with us, we do not speak in public about them. But during the training, we learned that those diseases we did not think of as important before -- I know now that those diseases are affecting our society even if we are silent about the disease. So understanding about the diseases and getting attention from the public and the government made me happy.

E07: For example, when she **[the Health Extension Worker]** *taught us, we observed a picture wich shows a woman with breast disease -- a woman without a breast who lost her breast, and a waman with a swollen breast. You know, this kind of disease can be found in our society and community members cannot disclose this kind of disease. But when we teach about the disease in public to the community, those who hide their disease can disclose easily and get treatment for their diseases. People may hide this kind of disease, but understanding about the disease can help us to disclose and make peaple aware of the diseases.* **[other participants agreed with this statement]**

R: Can you remember which diseases were discussed?

E03: Yes, for example I saw one picture when the Health Extension Worker taught us wich was terrific.

R: What was that terrefic picture you observed?

E03: **[after some hesitation]** I saw a man with a swollen scrotum **[all participants laughed]***. At that time, we raised the question to the Health Extension Workers saying, "You know, it is difficult to disclose and discuss this kind of issue with a man." You know, in our culture, women do not dare to talk with a man even about usual business. So how can we teach and discuss this kind of issue? But the Health Extension Worker replied to us that a person with such a problem, you can approach the man through a family member of the person. For example, if you discuss the issue with the wife of that individual, that woman can directly talk to him and he can disclose his issue. But culturallly, we find it difficult to reach the men with such a problem, because we fear to talk to men.*

E02: In addition, we talked about an eye disease, and accordingly I identified three people with eye problems. Those who cannot see things from a distance.

E04: And we talked about a disease which affects a woman's womb. **[several participants consented]**

E07: I would like to appreciate the person who sketched the pictures of those diseases. It enabled us to discuss the issue freely with other persons and also it helps us to clearly understand the diseases that exist in the community. Let me tell you one thing I did in my village. One day, I was near my house to have a coffee with my neighbour. As you know, in a coffee gathering, men and women gather together, and I tought I can make peaple aware of the diseases. I took the pictures out of my bag and distributed them to the men to see the pictures and to talk about that. Eventually, people gathered around and were surprised about my action. One individual among them said that "it is interesting the government is doing that because a lot of the people are not discussing this issue in our community. But starting to talk about this disease helps those who are affected to make themselves known. You know, there are people in our community with this disease, but we don't speak about it."

E06: We learned in the training about a disease wich makes the urine red. Previosly we thought when the colour of the urine changes to red or another colour -- our community considered this as a symptom of syphils, not other disease.

E04: We also discussed about a disease that makes peaple vomit worms.

E01: I have one question. You know, there is a person in my village with a problem of genital swelling. How can I approach that person? You know, there is no problem in approaching the female, but teaching a man would be difficult.

R: Was there anything you didn't like about this training?

E010: No, everything we learned was important to us, because we learned about a new thing, wich was not commonly spoken about in our community. **[all participants agreed that the training was relevant]**

R: After you received this information, what is your understanding of your role with regard to neglected tropical diseases?

E09: During the training, the Health Extension Workers told us that we should teach and discuss about these diseases in every meeting we attend. In addtion, the Health Extension Workers told us to refer patients with signs when we find them in the community we live in.

E07: For example, my role is teaching the community abaut diseases, but in addition I learned that I have to give emphasis to thoses deseases. And according to the reporting format, I report monthly to the Health Extension Worker.

[Two other participants reiterated that their responsibility is to detect people with signs and symptoms of NTDs and send them to the health post.]

R: Did the Health Extension Workers give you any tools when they shared information about neglected tropical diseases with you?

E07: Yes, she gave us materials like a reporting form, pencil, a pencil sharpener and pictures.

[All participant confirmed that they had received those materials, except one who had not received a pencil sharpener.]

[At this point, the researchers distributed hard copies of the pictorial tool developed for the Health Development Army and gave participants a few minutes to study the pictures.]

R: How is the tool to be used? What does it depict?

E04: As we see, the picture it is about diseases wich affect both males and females. The pictures indicates a disease which affects the male genital area, a disease wich makes people urinate blood, a disease wich makes females to have a swollen breast.

E01: We also see in the pictures a disease wich affects the eye.

E06: We use the pictures to teach others. It helps us to teach hoseholds using the pictures when we gather at our HDA meeting.

R: In your opinion, is the tool easy to use? Why or why not?

E03: What do you say my doughter **[referring to the researcher]**. *I like the the pictures very much because they allow me to understand things and to teach others easily too*. *I would even like the other health extension packages to be prepared like this, because it is so easy to teach.*

[Several participants reiterated that the pictures are helpful and easy to use.]

E09: The pictures are interesting. I would even like those pictures to be posted like a billboard in a public place to make them visible to all. Because people learn when they see things. When people see the pictures, the individuals realise the problem and may prompt them to search their home and their village as well when they see the pictures.

R: Do you actually use the tool in your role as a Health Development Army member? How do you use it? How often?

E02: Yes, we use the pictures.

R: When do you use them?

E02: We use the pictures when we visit the households to teach the community and when we have a HDA meeting.

E010: We use the pictures always when we vist households, because as the diseases are new to us, I teach showing the picture to all households I vist.

R: How could the tool be improved?

E07: I think the pictures are clear -- no need to improve it. However, I would like the pictures to be distributed to each household because it helps each household to realise the the problem.

E09: I would like it if it could be possible to hang the picture in public places to make them visible to all. Because when it is shown in public places, like market places and by the side of the road, people easily observe them and learn easily.

[This idea was supported by several participants.]

R:The intervention we are testing suggests that the Health Development Army should detect people with signs and symptoms of five diseases: trachoma, schistosomiasis, soil-transmitted helminth infection, lymphatic filariasis and podoconiosis. We want to discuss some diseases in more detail.

[At this point, participants were invited to select two groups of symptoms they would like to discuss in more detail. The first topic selected was trachoma.]

R: This disease affects the eye. What do you know about this disease? What about the signs and symptoms of trachoma?

E04: People who have trachoma cannot see things in the distance. Sometimes they are visually impaired and can't identfy colours. Their tears flow continuously and people are not able to read books.

E08: People who have trachoma find it challenging to identfy things close to them. Sometimes they see things duplicated. Sometimes they see a black point in the middle of their eyeball. Sometimes their eye will be filled with a white thing.

E03: When people have trachoma, their eyelids break continuously. Their eyelids sometimes swell up, sometimes their eye is filled with a white liquid.

E04: To tell you the truth, I don't know what exactly the signs of trachoma are, but I hope you will tell us the signs, as you are an educated person.

E06: I remeber there was a girl when I was at elementary school who couldn't read what was written on the blackboard. Some pupils said then that the girl had this trachoma disease and that this can be caused when someone is exposed to kerosen lamps for a long period of time. I think it is a disease wich affects people who use kerosene lamps for a long period of time.

E010: Trachoma makes all parts of the eyes ball white.

E09: I think trachoma causes eye pain and headaches.

R: Do you feel confident that you have the knowledge and skills to detect people who have signs and symptoms of trachoma in your community?

E09: Yes, I can identify the disease.

R: What gives you the confidence?

E09: Because I am trained on the signs of the disease and also I have a picture with me, so I can say I have knowledge to detect the disease. **[other participants signalled that they agree with E09.]**

R: Do you think you could detect trachoma patients without having the pictorial tool?

P: Ehh, atually, it will be easy to detect patients using the pictures

R: In your opinion, are there any problems with tasking the Health Development Army with detecting trachoma as suggested by the intervention?

E09: I don't think there will be a problem in identfying and detecting trachoma, but the challenge I expect is that unless there is free treatment given for the persons we identfy, the people will ask us, "Are the services given for free?" At that time, we may not have the confidence to respond to the patient. **[all participants agreed]**

P: There is a certain organisation wich provide free services for trachoma and accordingly, when they moblise the community, everybody goes there and gets treatment. Our confidence and motivation to work in the community would be strengthened, so your organisation needs to find a solution to provide free services for neglected diseases.

R: Do you ever see people in your community with signs and symptoms of trachoma?

E03: Yes, I have seen three patients in my village who had signs of not being able to see things in the distance and two children whose eye lids break continuously.

E010: I saw an old woman in my village -- when somebody greeted her, she had to ask the person who greeted her who she was becasue she was not able to identfy the person.

E03: I think we all forgot the issue we are discusing. Is there a person who doesn't have trachoma in our villlage with all those signs we mentioned earlier? I think if we checked our eyes at the health centre, we may all have tarchoma. For example, I cannot see at a distance. What about you? **[all participants laughed]**.

R: If you find a community member with signs and symptoms of trachoma, what would you do?

E03: I *advise the patient with these signs to go to a health facilty and also, I record and report the patient*. **[All indicated agreement that this is what they would do.]**

R: Do you think people will follow your advice?

E01: Yes.

R: Why?

E01: In our community, people most of the time follow what we advise them. That's why I believe the community can follow our advice.

R: If you have seen people with signs and symptoms of trachoma recently, did you encourage them to seek care? What happened next?

E07: I identfied two patients from the community and reported them to the Health Extension Worker.

R: Did those patients go to the Health Extension Worker or another health facility?

E07: Actuallly I did not contact the individual after.

R: Why did you not contact them?

E07: Because I was busy I did not contact them.

E02: I also identfied one patient in my village and advised her to go to the health facility, but she did not go to the health center.

R: Why not?

E02: She told me that unless somebody helps her, she cannot go to the health center as she is poor.

E09: I identified two patients in my village and one of them went to the health center and bought the medicine. It was 75 birr **[approximately £2]** *and now she is fine. However, the second person did not go. I think he does not have the money to pay for treatment.*

R: If you detect a community member with any of the signs and symptoms of neglected tropical diseases, do you record and report this?

E02: Yes, we record the case on the reporting form the Health Extension Worker gave us.

R: How do you record cases of communtiy members with signs and symptoms of NTDs?

E02: In our recording form there is a picture, so when we detect a patient with a certain disease, we simply make a mark on it. **[All agreed that thisis what they do to record cases they detect.]**

R: How do you report this? Who do you report it to? How often?

E09: As the Health Extension Worker told us, we report monthly for the next six months starting from June.

E07: When we find a case in the community, we write the name of the patient with the signs of the disease and finally report to the Health Extension Worker.

R: What are the main challenges with this recording and reporting process?

E01: Our challenge is the lack of pencil sharpeners. **[all laughed]**

E04: Actually we are following the Health Extension Worker's instructions. The challenge I observed is that the form does not identify the village. Unless the village is identfied, it is not possible to monitor the patient where he or she lives. The only thing we write is the name of the patient.

R: What do you generally think of the idea of tasking the Health Development Army with detecting people with signs and symptoms of neglected tropical diseases?

E01: I think the government is aware of our roles and responsibilities. We are there to reach the community. We have been working as leaders in our community for a long time and teaching our communities about health for last five years and more. Some of us have worked for about seven years.

R: So you think the Health Development Army has the capacity to take on these roles and responsibilities?

E010: Yes, why not? A lot of *work has been implemented through us so far, so I believe we have the capacity to do any activity.*

R: What kind of challenges do you foresee?

E02: Actually I dont think we will face any problems to implement the neglected diseases.

E09: We will not face many challenges, but I have doubts about two issues. The first one is, how can we approach men when they have the genital disease? My second question is, unless

free treatment is given for the patients, we are recording and reporting people with signs of the diseases who may not visit the health center for treatment. I think you or the government has to prepare something that supports the people in visting the health centre.

R: Do you think there would be any challenges with regard to stigma? Would those affected by disabilities caused by NTDs be detected by the Health Development Army?

E07: I dont think we will face any challenges in detecting the disabled people. Certain NGOs have worked a lot on stigma in our **[district]**. *In addition, I don't think in our community there are people who stigmatise those individuals. Everyone in our community is Christian and our religion does not allow followers to stigmatise people.*

E02: Since we are involved in detecting the people who are affected by neglected diseases -- since we are in the community, we can find people easily. We know each other's homes when we live in one village for a long period of time.

R: What do you generally think of the idea of tasking health workers at primary health care level (including health posts, health centres and hospitals) with detecting, managing, recording and reporting neglected tropical diseases?

E09: I think they can do everything, because there are doctors in the health centers. **[All participants agreed.]**

R:Is there anything else you would like to share with the research team?

E03: I think it is important to learn about these diseases and save lives in our community, but as the diseases are complex, we need some training. We would like work with you.

E08: What I want to say is, as you know, our community is poor, so please, if you can, try to arrange for those people we detect to provide free treatment.

R: This concludes the interview. Many thanks for taking the time to participate.

FGD 4

R: In May this year, classroom training was offered to Health Extension Workers from your health post about neglected tropical diseases. The Health Extension Workers were tasked with sharing information about those diseases with the Health Development Army. Did you receive this information?

E11 .It was about those neglected tropical diseases, such as eye problems, ear problems and also abdominal problems exist in my kebele, but they did not go to the health facility as soon as possible with this problem. Maybe because the abdominal cramps they have are on and off, they'll say that it is normal—"I'll be better soon."

R: So you got this information from a Health Extension Worker?

[Several participants confirmed that they had received information from Health Extension Workers.]

R: How many of you received this information?

[About half of the participants confirmed by show of hands that they had received information, but the others had not.]

R: For those who said no, do you know why not?

E14: Because no one told or called us during that time about it. I only heard about this now when you told us. You said it was in May, but no one has told me about it.

R: For those who said yes, how was this organised? Was it part of your regular interactions with Health Extension Workers?

E11: Let me tell you about my case. As usual, the health workers came to visit me and educate me. They both came to educate and talk with me in my home and they told me to share this information with those who are under my supervision in the 1-to-5 network. It was about those diseases which seems easy, but they stress people, so we need to create awareness about the diseases and make people appreciate the need to seek care.

E18: In my case, the Health Education Worker educated me and gave me a leaflet about those diseases. She said there may be people who have eye problems like broken eyelashes -- or for males, those who have swellings in the groin area. And she told me that since we are the Health Development Army, we lead 30 people, there is a 1-to-5 network under my supervision – you also have to teach the community about those problems and if there is a case, identify and report it to the health post.

E16: My situation is similar. I learned from the Health Extension Worker about a problem with urination – blood in the urine. If a person urinates blood and if the bladder doesn't empty all at once. She said that if we have such problems, we need to refer them to the health post.

R: Was this in your home?

E16: Yes, she educates me in my home.

R: How could the process of sharing information about neglected tropical diseases with the Health Development Army have been improved?

E18: I want to say that in the past, we were doing another activity, which wasn't as easy as we thought. Even though our people did not have knowledge about vaccinations, we mobilised

the community and for those who don't have a toilet, we advised them to have a toilet when we visited their homes. When there was no knowledge about how to use bed nets, we educated people about the proper way to use the nets by visiting their homes. So, in the future, if they train us and supervise us and send us to serve our community, we can bring about change in our community. We also have the opportunity to discuss things with our friends during coffee ceremonies, or in the edir meetings **[savings groups]** *or when we go to the market. What we discuss with our friends reaches people's homes indirectly. The government is working for us and the people for whom we are working are with us, we discuss with them day and night. If somebody gives us training, supervises us and the government receives our reports of what we have achieved and accept them, then nothing shall be kept from the community. If you want to see change, we need training and we are ready.*

E12: As my friend is saying, a lot of work has been done through our efforts and our environment has seen a lot of change. We conducted different activities such as bed nets, toilet use and how to care for children by doing home-to-home visits. So if you train us, we will cascade the information to those who are under us. We are ready to educate those who are under us in our community.

R: Can you tell me what you learned about neglected tropical diseases? For example, do you remember the names of the diseases?

E18: For females, they may have itching and pain around the genital area – that is a neglected disease. Also those people who have weight loss, those who are urinating blood -- those diseases result in swelling of the testicles.

R: Would anyone like to add to this?

E20: There are diseases which result in swelling of the breast -- one breast becomes large while the other is small. There may be a burning sensation during urination and dripping of urine or vaginal bleeding.

E19: What I can add is swelling of the abdomen, swellings on the finger, leg swelling, vomiting and 31diarrhoea.

R: What did you like about receiving this information?

E11: I liked the information on problems affecting women's uterus, which are not given enough attention and those who have such problems do not disclose their problems because of our culture. Women may not feel comfortable telling even a doctor, so we have this information, we can discuss it openly and ensure we do not miss women with those problems. We can get medicine for this neglected disease and we can get health education from the Health Extension Workers and go to the facility for treatment. It is good information to go to the facility early with this problem.

R: Was there anything you didn't like?

E18: There was nothing I disliked because that is my work, but for people with swollen testicles, it would be better to train males to educate those men. I'm afraid that will not be practical for us.

R: What do you suggest?

E18: It would be better to educate some men together with us. It is important to identify people with this problem through them. Because they may hide their problems from us women -- they may hide the swelling.

R: Did you think the information you received was relevant for your role as a Health Development Army member?

[Those who had received the information signaled that they thought it was relevant.]

R: Why do you think it is important?

E16: Because this neglected disease harms slowly. Other communicable diseases need immediate action, but this one does not. Our reward is seeing a healthy person after they have been educated, get treatment and become healthy.

R: Any other thoughts?

E18: If women are educated, they teach those women who have problems with their uterus to get better treatment. And also by learning all of this, we prevent disease and death among children and adults from neglected diseases. We also encourage those women who hide their problems to talk about their problems.

R: Did the Health Extension Workers give you any tools when they shared information about neglected tropical diseases with you?

E19: Yes, we received these tools. We tried to find people with the problems the pictures show, but we did not find any who have those problems and we reported that to the Health Extension Worker.

R: How do you use the tool?

E19: We have been searching cases by showing these pictures and educating about those diseases. If there is a person who has breast problems, we advise them -- especially women -- by saying, "We are working for your health not to mistreat you." And for those who have swollen feet inside their shoes, we advise them not to hide their problems – "If you hide your problems, in the long run, you may experience unexpected crises." Or for those who have urinary problems, "While you are hiding the problem, your smell may turn into a bad odour. If you have such problems, tell me without fear." I do that while showing the pictures.

R: How often do use the tools?

E12: We used the tool twice in our community. That was the assignment given to us by the Health Extension Workers -- to educate and search cases and to report them.

E11: The Health Extension Worker came to me and discussed this with me. And I also discussed it with those under me. I told them the situation about those neglected tropical diseases -- not to hide the problems in the community. So I told the 1-to-5 network that the Health Extension Worker told me these things and we shall all do this together by discussing if there is anyone in the community who has such problems. Then after discussing with them, we report to the health post on a monthly basis. We read the paper **[the pictorial tool]** *together. I distributed the paper to them and I filled the form accordingly. Whether or not there are cases, I report to them. I even reported last month.*

R: How do you use the tools?

E19: Since we are the Health Development Army, we have a meeting once a month and with those who are in my 1-to-5 network, we have a meeting every 15 days. We discussed this problems in 1-to-5 network and in Health Development Army meetings.

R: Was there anything else you didn't like about the tools?

[No further challenges were raised.]

R: What did you like about those tools?

E19: What I like is that our people may hide those problems, but now we learned a lot about the problem and they have a chance to get information from us and go to the health facility.

R: How could the tool be improved?

E17: What I want to say on the tools and the information what received – in my opinion, it is better to be trained with the 1-to-5 network members and in the presence of health workers.

R: Do have anything to add? Is the tool easy to use?

E17: We have nothing to add. It is clear to use.

R: Do you ever see people in your community with signs and symptoms of neglected tropical diseases? What are the signs and symptoms people have?

E12: In my village, there are people who have swollen legs.

R. How many?

E12: There are two individuals with leg swelling.

E20: In my village, there are three individuals with swollen legs and tree individuals who have eye problems.

R: What is wrong with their eyes?

E20: I think they have whitish spots on the black part of their eyes. Though they are not blind, their vision is blurred. There are three or four people who have such problems.

E17: There is one person who has a swollen scrotum in my village, but he always hides it. And there is one lady who has swellings all over her body. There is another person who has a damaged eye from an accident. He became blind despite doctors' best efforts and now he is using glass eyes..

R: Anyone else?

E15: In my village people also have eye problems and there is an old women with leg swelling. There's another lady with a swollen leg, but she hides it under long dresses so people don't see it as it is seen as a taboo.

E11: There is a women who has problems with her uterus. She went to the health facility repeatedly, but the problem recurred after three or four months. She also has abdominal cramps and loss of appetite, so she is very thin.

R: You told me about different signs and symptoms, but which ones are the most common?

E19: Swollen legs are a problem. Swelling of the breast is another issue. If someone loses an eye, they may lose everything, because eyesight is the most important thing for human beings.

R: One of disease this intervention is interested in is called schistosomiasis. One form of the disease affects the urinary system. What do you know about this disease? What are the signs and symptoms of urinary schistosomiasis?

E16: What I know is that it is a urinary problem. There is a child who has these symptoms and he also had itching around the groin region. I am planning to send him to the health facility.There is also one woman who has vaginal bleeding. I also want to send her to the health facility.

E14: I know one boy -- when he urinates, he feels a lot of urgency. The child is my son, but we still haven't taken him to a health facility. But I am planning to consult a health worker.

R: Do you feel confident that you have the knowledge and skills to detect people who have signs and symptoms of urinary schistosomiasis in your community?

E11: Yes, I have enough knowlegde.

R: How so?

E11: We identify the signs and symptom in the pictures and we searching according to this tool. If there are people who hide the problem they have, we have the knowlegde to identify the problem.

E19: With the help of the pictures on the tools, we ask the 1-to-5 network members, "Please tell us if there are symptoms like these." They then tell us about people who have this problem.We can then offer a solution for the problem.

E15: I say I have enpough knowledge. When I see a child urinating with my naked eye, I have the knowledge to identify the disease.

R: What kind of symptoms do you see?

E15: Children with blood in the urine and not being able to empty the bladder as usual.

E19: I can identify it if the mother tells me about those urinary problems. When the mother tells me, I write it on the report form next to the picture with those symptoms. So that's how I can identify the symptoms.

E18: For me, I just have the tool with information about those diseases, but I've not had any training on identifying the signs and symptoms. When the problem occurs, I simply read the information in the tool and then I work as usual. In the future, I will be even better because I have enough knowledge now.

E17: The problem I face is with those who are difficult to talk or ask about.

R: What are those problems?

E17: In our culture, it is difficult with those who are older and also with men. Otherwise we can talk about the diseases with the other communtiy members when we visit their homes.

E11: For me, since I'm the leader of 30 households, I have 29 mothers in my network, so they are responsible for telling me about the problems they have and then I pass these on the the Health Extension Worker.

R: What do you do if you have a person who has a problem?

E18: If i have a person who has those problems, I will send them to the Health Extension Worker. First I advise the person to pay attention to their problem and then I tell the Health Extension Worker about their problems.

R: In your opinion, are there any problems with tasking the Health Development Army with detecting urinary schistosomiasis as suggested by the intervention?

E19: I do not think there will be any problems in identifying and detecting the urinary problems, except difficulties in asking older people about those problems.

R: If you find a community member with signs and symptoms of intestinal schistosomiasis or soil-transmitted helminth infection, what would you do?

E11: We can advise and send them to the health centre for the treatment. I advise the patient to go to the health facilty for the treatment and also, I record and report the patient to the health post.

E18: As she told you, our work is to identify and send cases to the health facility for treatment.

R: How could the problems you mentioned be addressed? What alternatives or improvements would you suggest?

E19: My suggestion is to educate the rest of our community together with us. The government should support the Health Development Army with training.

[All participants agreed with this statement.]

R: When you advise people to seek care at the health post, do you think people follow your advice?

E18: Yes, they accept our advice. In our community, people follow our advice,follow our advice most of the time and go to health facility for treatment.

R: The final two diseases this intervention is interested in are also very similar, even though they have different causes. They are called lymphatic filariasis and podoconiosis. They mainly affect people's lower limbs and sometimes their genitals. What do you know about those diseases?

E11: People say it's like if something sharp -- like a needle – pierces the leg. It causes swelling of the legs, from the foot up to the knee.

R: What is the cause?

E11: The sharp object.

R: Do youfeel confident that you have the knowledge and skills to deetct people who have signs and symptoms of lymphatic filariasis or podoconiosis in your community?

[All agreed that they can identify the diseases.]

R: If you find a community member with signs and symptoms of lymphatic filariasis or podoconiosis, what would you do?

E19: I try to discuss with the person who has such problems and also I bring them to the health post for further treatment.

R: Where would you recommend they seek care?

E18: Previously, we did not know that the person can receive treatment and we had no solution for their leg, so we were scared of sending them to an institution where they are not responsible. We got the information from the health facility about where to send people with those problems. Once we record such a person, they will ask where and when they will receive services.

R: Do you think people will follow your advice?

E18: Yes, they accept my advice, but they need a place where they receive services.

R: If you detect a community member with any of the signs and symptoms of neglected tropical diseases, do you record and report this?

E19: Based on the picture in the tool, we will just put a tick.

R: How often do you report?

E19: We report to the health post monthly.

R: What is your overall opinion of this intervention?

E11: The intervention is good. All of us can take responsiblity, but we need regular supportive supervision and updating with training.

Interviews

Lab staff, hospital

R: In May, training was offered to health workers from your hospital about neglected tropical diseases. Did you attend this training?

B03: Yes.

R: What did you like most about that training?

B03: What I liked about the training was that previously NTDs were not given attention and were not considered important, but after the training, I understood how much those NTDs need attention and they are a cause of concern.

R: Do you remember which laboratory test this training covered and which diseases are detected with those tests?

B03: Concentration technique.

R: Stool concentration technique?

B03: Yes, stool concentration technique and direct wet mount is what we adressed during the training.

R: Okay, wet mount is the test you used previously?

B03: Yes, wet mount is what we did previouly in our routine activities. But for those diseases which are not diagnosed and it is difficult to get the parasite load with the direct test,stool concentration technique and.wet mount -- actually that is what we did previously.

R: Anything else?

B03: Eh, other things I do not remember.

R: What did you like about the training and these tests? [Interviewee does not respond] Do you remember the number of tests included in the training?

B03: Obviously, they listed them, but I do not remember them.

R: Which tests give you comfort and are easy to perform?

B03: The type of test that gives comfort is -- actually, I do not have any comfortable tests, because in this hospital where I am working now, they need time. Here we are only doing wet mount. There is a high patient flow. The tests which were included in the training need more time. Minimum they need half a day, we were told.

R: For a single person?

B03: Yes, for a single person. So it is very difficult to spend half a day on a single patient and for some of the procedures, the whole night will be needed. These things make those services more time consuming compared with the direct wet mount, which will take one or two hours. They would not be difficult to perform, but because of the patient flow, that makes them very difficult to perform for every NTD patient.

R: So even if the training was good, the tests do not consider the patient flow?

B03: Yes and the time they take.

R: And time.

B03: Yes.

R: So how could these problems be improved?

B03: The problems I mentioned earlier -- Now the tests should be done because patients with these problems should get treatment. For example, we can get schistosomiasis by direct wet mount, but there are also times we fail to detect the parasite. So if we don't want to miss cases, a responsible person should be assigned to this specific purpose, then doing the test will be fine. Specifically one, two, three persons assigned to this task will be okay.

R: Specifically for those diseases, you mean?

B03: Yes, for those diseases, if the tests are requested, there will not be a problem. But otherwise, with the patient load, it will be difficult.

R: Okay, with your other responsibilities.

B03: Yes, with all other responsiblities.

R: Do you have any other sugeggestions? You said the test is time consuming.

B03: Yes, time consuming. If we do it based on the procedural steps, it is time consuming.

R: Do you recommend other tests?

B03: With regard to NTDs, there are schistosoma, trichuris – What's it called? Anyway, there are parasites that will not be found with the direct wet mount test, so the patients will not be benefited.

R: The training covered thee laboratory tests: urinary sedimentation, stool concentration and Circulating Cathodic Antigen (CCA) test. Do you ever perform those tests?

B03: Yes, but I do not remember that much.

R: Maybe were there tests which are done using urine or blood?

B03: Yes, in urine, schistosomia heamatobium is detected. Stool is for mansoni.

R: Any others?

B03: And for other schistosoma species, the tests are not available in our country.

R: So there are tests using stool and urine concentration and CCA. Do you know how to perform a urine sedimentation test?

B03: No.

R: Is it because of the reasons mentioned before or dou you have other reasons for not performing the tests?

B03: I do not have any other reason other than the ones I mentioned earlier.

R: Can we say you do not have a skill gap to perform the tests?

B03: No, I don't lack the skills.

R: What kind of materials and equipment do you need to perform the tests?

B03: Urine sedimentation is done usually to detect schistosoma heamatobium. The sedimentation technique -- There is a urine tube. With that tube, we recieve the urine. Then we bend and let the urine stay for some hours to sediment. With the urine sediment, eh -- I do not remember the procedure clearly, but I'm trying to tell you the general principle.

R: Okay.

B03: We use the sedimented urine.

R: So the materials are...

B03: Test tube, microscope [...]

R: Okay, microscope. What other materials are needed to perform the urine sedmentation test? Do they exist in this hospital?

B03: I am not sure of the materials.

R: How many minutes does it take to perform the urine sedmentation for single person?

B03: Urine sedmentation does not take minutes

R: Sorry, I mean time.

B03: It will take almost half a day.

R: Half a day

B03: Yes.

R: Do you mean half of 12 hours or half of 24 hours?

B03: To sediment the urine, it can take 1 hour.

R: To see under the microscope?

B03: Maximum two hours, minimum one and a half hours.

R: The intervention we are testing suggests that laboratory staff at hospital level should use the urine sedimentation test to detect urinary schistosomiasis. Do you feel confident that you have the knowledge and skills to perform this test if adquate time is given and if you are assigned to perform this test?

B03: Yes, I can perform with confidence.

R: **If yes, what gives you the confidence?**

B03: *The training obviously helped me to learn. If I was assigned to do this test, I am confident I can do it by updating myself.*

R: Do health workers at this hospital ever request this test? If no, why not for example for schistosomiasis?

B03: I am not sure because we are rotating regularly every week and we are assigned one responsiblity at a time.

R: You did not come across a request?

B03: I have done direct microscope test, but not the sedmentation test.

[There was a knock on the door and the interview was intruppted for a few minutes.]

R: So it means you have never done the tests?

B03: Yes.

R: Do you think the test results are reliable compared with the direct one*?*

B03: In the direct test, the range to get the parasite is less, but in the sedimentation test, I expect there is high chance of detection.

R: How could the problems you mentioned be addressed? What alternatives or improvements would you suggest?

B03: For urine sedimentation, I don't think we need more materials, but for stool concentration, we need more materials to do the tests.

R: What do we detect with stool concentration?

B03: Stool concentration is done for mansoni, Schistosoma mansoni, Tricuria and, eh –

R: For STHs.

B03: For STHs, eh, strongloid sterocolaris, Strongloid are normally not present in stool concentration. More are detected in the direct test.

R: Others?

B03: Yes, Intestinal schistosoma.

R: What kind of additional materials will be needed to do the stool concentration?

B03: For stool concentration, we have materials for direct wet mount, like the test slide, cover slide and NS.

R: Normal saline?

B03: Yes, normal saline.

R: What about the stool concentration test?

B03: Test tube, materials to filter the stool [--]

R: Can we say stool filter?

B03: Yes, stool filter, stool measurment [--]

R: Does it take the same time as the urine test?

B03: To detect schisto the time is similar, but if we want to see other things, based on our procedure, the whole night may be needed.

R: But for NTDs it takes the same time?

B03: Yes.

R: Do you think the procedure is diffficult to perform?

B03: *No I dont think it will be difficult, but as I said before, if it is additional to the work station, that will be difficult*.

R: The intervention suggests that laboratory staff at hospital level should use the stool concentration test to detect intestinal schistosomiasis in patients over five and and soil-transmitted helminth infections in all patients. Do you feel confident that you have the knowledge and skills to perform these tests?

B03: Yes, I can perform with confidence. This is because -- first becasue of the training and secondly if the materials are available and there is no other burden, not only me, but all technicians can perform the tests.

R: Do health workers at this health hospital ever request this test? If no, why not?

B03: No, I haven't come across any request.

R: In your opinion, are there any problems with performing the stool concentration test at hospital level as suggested by the intervention. Do you have other ideas?

B03: No, it is the same as urine concentration test.

R: The materials needed for the stool concentration test are not different from the direct test?

B03: Yes, they are different, but in my opinion, the hospital should not find it difficult to afford those materials.

R: In your opinion, what are the implications of performing this test in terms of time and workload?

B03: As I have said, to maintain the quality of the test, a separate person should be assigned because it is not possible to change the work station. This test is more reliable than direct wet mount.

R: Do you know how to perform a Circulating Cathodic Antigen (CCA) test?

B03: Not at all

R: Is the test ever requested?

B03: Yes.

R: Which diseases can be detected with this test? What kind of materials and equipment do you need to perform the test?

B03: I don't remember it.

R: How much time does it take to perform the test?

B03: I don't know.

R: Because you never performed it?

B03: Yes.

R: In the training, was there a practical session?

B03: Yes, there was a demonstration, but I forgot it.

R: How difficult is it to perform the test?

B03: It was not that difficult to perform.

R: Could you perform with the guide?

B03: Yes sure.

R: What do you generally think of tasking laboratory staff at hospital level with performing tests to detect neglected tropical diseases?

B03: The training was good, because I did not expect it like that. I recommend that other professionals also have a chance to attend training and to have continuous updates, so I don't forget the procedures and understand more about NTDs.

R: What about NTDs?

B03: The tests are very important for the patient and for us it makes us confident and it will improve the working materials.

R: What do you generally think of tasking laboratory staff at health centre level with performing tests to detect neglected tropical diseases?

B03: The training should be offered for all levels and the materials are not so complex. In addition, the workload is low in health centers, so they can perform better.

R: Do you think there would be any challenges with regard to gender? Would women and men face different challenges in accessing care for NTDs at the hospital?

B03: I dont think there will be a difference.

R: Do you think there would be any challenges with regard to stigma? Would those affected by disabilities caused by NTDs access care at the hospital?

B03: Actually, this is more relevant for nurses who are giving care for disabilities. II don't think there will be a problem to treat them like other patients. As a lab technologist, this is not really my responsibility **[unintelligible]**.

R: What could be done to address those challenges?

B03: *Awarness is very important. Talking clearly with staff and with the patient. It is still possible to avoid bad odors with simple awarness, so it can be done by providing information.*

R: Is there anything else you would like to share with the research team?

B03: Conducting the research is good. There is a lake in Hawassa, so I recommend you to have some community awarness creation activities. Activities in hospitals and health centers are good, but barefoot walking and other risk factors are within the community, so the community has to be the main target of the programme.

Health worker, hospital

R: In May this year, classroom training was offered to health workers from your hospital about nelected tropical diseases. Did you attend this training?

B05: Yes, I participated. It was given at **[name of hotel where training was held]** *by exprienced professionals who shared their experience. We actually knew those diseases, but because of exposure and frequency of those problems, we used to neglect them a little bit. The training helped us to focus on those neglected problems and it woke us all up.*

R: Do you have other things to tell me about the training?

B05: The training was good, but we were saying even during the training that the frequency of the training should be increased. In this area, as you know, the most common health problems are acute febrile illness, diarrhoea, pneumonia and so on, so we are more exposed to those problems and may forget about NTDs. But if the training is given frequently until we become familiar with those diseases, that will be good.

R: So the training reminded you.

B05: Yes, it helped to be reminded of the diseases and give quick care to the patients.

R: Do you remember which diseases this training covered?

B05: Ehh, there is leishmaniasis.

R: Leishmaniasis, okay.

B05: And also --what do you call it [...]

R: Okay, no problem. What did you like about the training?

B05: What I liked is that now, when patients are coming to my office in central triage, I can distribute them to medical surgical and gynaecology. But previously, when patients came with NTD-like symptoms, for example blood in the urine, I used to consider kidney stones and then I would send them to the surgical side rather than the medical side. But because of the

training, I will now tell the patient to request NTD tests from the physician. Even I sometimes fall back into my old thinking patterns, though. But the training made us more aware.

R: Was there anything you didn't like about the training?

*B05: The training was good for education, but the payment was low in my opinion. Maybe that's because of my high expectation*s. **[laughs]**

R: How could the training have been improved?

B05: By increasing the frequency of the training and for longer period of time. It would be better than a short training to make us understand more deeply. And by increasing the per diem.

R: Did you think the training was relevant for your job?

B05: Yes, very helpful, as I said before.

R: What is your understanding of your role with regard to detecting and managing NTDs?

B05: The patients will tell us the clinical picture and additionally, we will perform a physical examination. We will see some of the signs and finally, to diagnose reliably, the laboratory will confirm the diseases.

R: Were you given any materials during or after the training to help you remember what was taught and to help you detect and manage NTDs?

B05: Pardon?

R: Did you receive those materials? Materials which can be referred to like a manual? [no response] Do you not remember or were they not given?

B05: Ehh, at the training they said it will be photocopied and given, but I have not yet recieved it.

R: The training and the materials that were distributed provide suggestions for how health workers at hospital level should detect and manage six diseases: trachoma, urinary schistosomiasis, intestinal schistosomiasis, soil-transmitted helminth infection, lymphatic filariasis and podoconiosis. Do you ever see patients with signs and symptoms of any of the diseases?

B05: Actually, my role is selecting and departmentalising the cases and send them to the right department.

R: No NTD cases faced?

B05: I have seen trachoma, filariasis [...] That's all.

R: Do you ever face schistosomiasis?

B05: I suspected one case of schistosomiasis.

R: Intestinal or urinary?

B05: Yes, it was intestinal schistosomiasis.

R: A patient with bloody diarrhoea?

B05: Yes. I even suspected a surgical problem or anal fissure.

R: Do you know how health workers at hospital level usually detect intestinal schistosomiasis?

B05: Yes, it is possible.

R: What signs and symptoms would you look out for?

B05: For example, a stool concentration test will help to detect for patients with bloody diarrhoea. And some of them present with AFI-like **[acute febrile illness]** *symptoms.*

R: If a patient has signs and symptoms of intestinal schistosomiasis, do you know how health workers at hospital level usually reach a diagnosis?

B05: Yes.

R: Would you request a particular laboratory test?

B05: I send the suspected cases to the medical side OPD. Sending to the laboratory is not my responsiblity.

R: If a patient is diagnosed with intestinal schistosomiasis, do you know how those cases are usually managed at hospital level?

B05: Yes it is possible.

R: Would you provide any treatment?

B05: Not here, but I treated cases when I worked in **[name of health centre in a rural area]** *as a Medical Director.*

R: Do you refer cases and where do you refer?

B05: I refer internally to the medical OPD.

R: Would you recommend any kind of follow-up? When you treated cases in the rural areas, did you provide follow up?

B05: Yes, I did.

R: What kind of patients need follow up?

B05: Patients who develop complications need follow up.

R: The intervention we are testing suggests that health workers at hospital level should detect intestinal schistosomiasis based on whether the area where the patient resides is known to be endemic for the disease and the following symptoms: diarrhoea, anaemia, malnutrition, abdominal pain, jaundice,ascites, intestinal blockage. Do you feel confident that health workers at hospital level can perform these responsibilities?

B05: Ehh, we usually send them to the medical OPD. But because we new equipment, we can detect this.

R: You may have a chance to do this in OPD in the future in that case?

B05: Yes, if the services are performed, I can perform them with confidence.

R: If everything is available.

B05: Yes.

R: What gives you the confidence?

B05: It is because of the training, but I would be even more confident if the training was more frequent. It would make us focus on NTDs more. Not only for me, but all health staff, including laboratory staff. Because the hospital is growing.

R: In your opinion, are there any problems with tasking health workers at hospital level with detecting intestinal schistosomiasis as suggested by the intervention?

B05: No, we definitely have enough professionals. The hospital is upgrading **[from aprimary hospital to a general hospital]***, so we can perform this task. The hospital is by definition expected to fulfil all services.*

R: So it can be performed.

B05: Yes.

R: In your opinion, what are the implications of performing this task in terms of time and workload?

B05: Of course there will be workload, but still, we need to coordinate with health centres and first-level health facilities to each take different responsiblities for treating the patients.

R: Health posts?

*B05: Yes we can liaise with them***.**

R: Okay, so it is possible to overcome time and workload problems by coordinating with the lower-level health facilities?

B05: Exactly.

R: The intervention suggests that an intestinal schistosomiasis diagnosis should be confirmed through a CCA test for patients up to five years or a stool concentration test for patients over five. Are these tests you think can be performed at the hospital?

B05: Yes, it can be performed.

R: In your opinion, are there any problems with performing CCA and stool concentration tests at hospital level as suggested by the intervention?

B05: In my understanding, all of us have promised to provide the services demanded by the community. We all swore an oath when we graduated. I always remember that oath. The government has also directed us to change. Now good things are on the way and if you work extra time, you will be paid for that, so I dont think workload will be a problem. I am not comfortable with this kind of complaint. We are free to decide to serve the community or not.

R: So you are saying it is possible to perform those services.

B05: Yes.

R: The intervention suggests that patients diagnosed with intestinal schistosomiasis should receive praziquantel if the CCA or stool concentration tests are positive, including patients up to five years. Patients over five years with any of the symptoms of intestinal schistosomiasis should be given praziquantel presumptively if it is not possible to perform a CCA or stool concentration test. Do you think health workers at hospital level have can perform these responsibilities?

B05: I think it would be better to treat after the laboratory test is positive, because sometimes the suspected case may not be confirmed by the lab, so a laboratory test is helpful to detect and treat cases. Do you understand me?

R: Yes.

B05: It would be better to treat after confirmation.

R: In your opinion, are there any problems with tasking health workers at hospital level with managing intestinal schistosomiasis as suggested by the intervention?

B05: Because we have a standard drug store, I think it is possible.

R: In your opinion, what are the implications of performing this task in terms of time and workload?

B05: Initially, there will be more workload because the hospital is performing with health centre staff. But we are building infrastructure, so in that case it will not be a problem in the future.

R: In your experience, are drugs and equipment required to perform this task normally available at this hospital?

B05: I don't know about the pharmacy.

R: Do you know how health workers at hospital level usually detect lymphatic filariasis? [participant does not respond and looks confused] It is also called elepahantiasis.

B05: Yes, it can be managed. I saw patients with the disease in the streets. They used the disease for begging and did not come for tratment, which makes the disease more complicated. So I tell them to come and seek care at this hospital.

R: You refer them here?

B05: Yes, I said, "Go and ask for the service. It is given for free." I give them health education about the risk of amputation in the future and encourage them to come here.

R: What signs and symptoms would you look out for?

B05: Oedema of the leg, itching, odor, soreness with infection because of the normal bacteria found on the skin. They can develop cellulitis, abscess and sometimes it can extend to the bone to form gangrine, which may lead to amputaion.

R: It becomes complicated.

B05: Yes.

R: If a patient has signs and symptoms of lymphatic filariasis, do you know how health workers at hospital level usually reach a diagnosis?

B05: We discussed and agreed during the training that some health workers with special and repeated training can detect it.

R: So you are recommending that this should be done by different health workers with special skills?

B05: Yes.

R: Would you perform any particular clinical examination?

B05: We have to check the blood circulation status and then the precence of gangrine

R: If a patient is diagnosed with lymphatic filariasis, do you know how those cases are usually managed at hospital level?

B05: Yes.

R: Would you provide any treatment?

B05: No.

R: But based on the complications you mentioned earlier, what kind of tratment may be needed?

B05: According to the presence of comlications, elevation of the leg during sleep, treatment with antibiotics for secondary infected wounds [...]

R: For secondary infection?

B05: Yes.

R: Under what circumstances would you refer the patient? Where would you refer the patient to?

B05: After giving them antibiotics.

R: What kind of cases need referal?

B05: If there is gangrine.

R: Would you recommend any kind of follow-up?

B05:Yes, it is needed to check for the develpoment of secondary problems. **[at this point there was some disturbance from staff entering the room where the interview was conducted]**

R: The intervention we are testing suggests that health workers at hospital level should detect lymphatic filariasis based on whether the area where the patient resides is known to be endemic for the disease the following symptoms: non-malaria fever and chills and redness of the leg, swelling of a lower limb, pain in a lower limb, elephantiasis. In patients over five years, the disease should also be detected based on the following symptoms: swelling of breast, swelling of an upper limb, swelling of the scrotum or vulva, hydrocele.

B05: Yes.

R: Do you think health workers at hospital level can perform these responsibilities?

B05: As you see, the hospital is busy on the surgical side and very crowded. There is a high patient flow with little infrastructure. As health centres have fewer in-patients, I prefer for these kinds of cases to be referred.

R: But it can be detected?

B05: Yes.

R: How could the problems you mentioned be addressed? What alternatives or improvements would you suggest?

B05: There may be a shortage of materials because the hospital is in the process of upgrading. Professional demand is also a problem and there is a space problem. But I think that after the G+8 building is finished, the problem will be solved.

R: What about workload?

B05: Yes, all of this is important. Health workers should be trained and prepared coordinating their departments. It will be easy to give the services.

R: The intervention suggests that patients diagnosed with acute lymphatic filariasis should receive pain relief with paracetamol, and be advised to hydrate and rest. All patients should be referred to a health post for advise on home management of lymphedoema. Patients with hydrocele should be referred to an appropriate hospital for surgery.Do you think health workers at hospital level can perform these responsibilities?

B05: Yes they can do this. Because if we forget to treat, the patients will be exposed to other problems. They may even die, so it is mandatory to treat the complications.

R: In your opinion, are there any problems with tasking health workers at hospital level with managing lymphatic filariasis as suggested by the intervention?

B05: No.

R: In your opinion, what are the implications of performing this task in terms of time and workload?

B05: There is no room for this kind of treatment in this hospital, but as I said before, we coordinate with the lower health facilities, so we can refer them downwards for wound care. For example, if someone has had surgery here, then he or she will be referred to a near-by by health centre for further care like wound care and stich removal.

R: What do you generally think of the idea of tasking health workers at primary health care level (including health posts and health centres) with detecting, managing, recording and reporting neglected tropical diseases?

B05: Training. It is very important to give training on detecting, diagnosis and management of NTDs. Without the training, it will be meaningless, as there is no focus on those uncommon cases.

R: So if they are trained, you are saying it is possible for them to perform those tasks.

B05: Yes.

R: Do you think there will be any challenges with regard to gender? Would women and men face different challenges in accessing care for NTDs at the hospital for example in the case of LF?

B05: We need to give advice to families on how to deal with those patients, but if we don't give them good care, the patients will be traumatised psychologically. We have to tell them about treatment options. Good health education will be one of the solutions.

R: What about you as a health professional? You are saying this is for the community.

B05: Yes, I have not experienced this kind of difference. Me and my colleague **[name of female colleague]** *work very closely together to discuss and manage problems.*

R: Do you think there will be any challenges with regard to stigma? Would those affected by disabilities caused by NTDs access care at the hospital?

B05: Yes, that is why the patients are not coming here, so health education is mandatory to change the mindset of the community.

R: What could be done to address those challenges? One thing you have said is health education, but what other options do you suggest?

B05: Early diagnosis and tratment and preventing the complications is also an option which illustrates that NTDs are curable.

R: You are saying paients witnessing that NTDs can be cured will reduce the stima?

B05: Yes, ofcourse.

R: What would your recommendations be with regard to integrating the detection, management, recording and reporting of NTDs into primary health care everywhere, even in central triage?

B05: We graduated to give demanding care for the community, so if the challenges are overcome and training is given, it can be possible and a good idea.

R: Is there anything else you would like to share with the research team?

B05: Thanks. I think early detection, diagnosis and managment and finally increasing awerness among the community are mandatory for this intervention. It will be helpful to solve the problems of the community. So I advice them to continue like this and strengthen the implementation.

Health worker, hospital

R: Thanks for your voluntary participation. In May this year, classroom training was offered to health workers from your hospital about neglected tropical diseases which was organised by Malaria Consortium. Did you attend this training?

B06: Yes, I participated.

R: Can you tell me what you learned in this training?

B06: The training was very nice. It reminded us of neglected problems and to focus on things that were little known. Most of the participants learned so many things that were new to us.

R: Do you remember which diseases were covered by the training?

B06: About the diseases -- the majority of those addressed by the training are problems affecting rural areas. Those diseases cause, for example, elephantiasis and similar problems found in areas where clay soil is found. We also tried to see how to manage those problems, how to handle treatment options and so on.

R: Was there anything you didn't like about the training?

B06: No, there was nothing I did not like. All of the things thatw ere taught were important.

R: So everything was helpful for your job?

B06: In our hospital, the cases are not common, which led to us to forget about those diseases. So the training was helpful for us. Actually, the diseases are common in rural set-ups. But if we are faced with those problems, the training helps us to manage them.

R: After the training, what is your understanding of your role with regard to detecting and managing NTDs?

B06: Yes, I understand my role. In the OPD **[Outpatient Department]***, my role is to identify patients who come with signs and symptoms of those problems. And by cooperating with the laboratory department, we are expected to report those cases to the relevant authorities. This is our responsibility and then also the management of cases.*

R: Can you tell me the signs and symptoms of NTDs?

B06: The signs and symptoms are oedema starting from the bottom and developing upwards, redness and itchiching of the area and, if the oedema grows, there will be cracking of the skin in this area.

R: Were you given any materials during or after the training to help you remember what was taught and to help you detect and manage NTDs?

B06: Yes, it was provided.

R: You have it personally?

B06: Yes, I was given those materials, but not for personal use. Rather, there is one doctor who has the job aid and we usually refer cases to her. You should meet her.

R: Have you ever used the job aids?

B06: No, I have never used the job aids.

R: In your opinion, are the job aids helpful and user-friendly? Why or why not?

B06: Yes, they are helpful and user-friendly because they help us with knowledge for our work.

R: The training and the materials that were distributed provide suggestions for how health workers at hospital level should detect and manage six diseases: trachoma, urinary schistosomiasis, intestinal schistosomiasis, soil-transmitted helminth infection, lymphatic filariasis and podoconiosis. Do you ever see patients with signs and symptoms of any of the diseases?

B06: No, I do not face patients with those signs and symptoms myself, but in the Emergency OPD they faced a case.

R: Why do you think this is the case?

B06: First of all, at this hospital, patients come from different urban areas, not from rural places, so I have never seen a patient with NTD-like problems.

R: Never?

B06: No, I've never seen it.

R: Which diseases would it be easier for you to talk about? For example STH cases or others?

B06: Ehh, I faced one case of STH. We carried out a lot of steps and even sent for sonography to detect the disease.

R: Do you know how health workers at hospital level should detect soil-transmitted helminth infections? What signs and symptoms would you look out for?

B06: Actually, it was difficult for us to detect the case as STH, because he was severely aneamic, so we considered other problems, not NTDs. The doctor was thinking about another problem, but finally we sent him for ultrasound and stool examination and it was an STH case.

R: What was the finding?

B06: The lab test result was -- ehh, I don't remember. The lab result didn't even indicate the problem that much.

R: What was that?

B06: It confirmed Trichuris, but we sent for sonography examination repeatedly to confirm the problem.

R: What type of stool test was sent to the laboratory? Do you remember it?

B06: Yes, I remember the stool test was direct microscopy.

R: If a patient is diagnosed with soil-transmitted helminth infection, do you know how those cases should be managed at hospital level?

B06: Ehh, treatment was – ehh [...]

R: For example, you got trichuris. How do you manage it?

B06: Because he was severely aneamic, we referred him.

R: For aneamia. But did you not give any treatment here?

B06: No, we referred him for management. No treatment was given here.

R: What do you recommend how this hospital should handle NTD cases?

B06: Actually, it is difficult to manage these cases in our set-up because of the workload, because it is time consuming and the high patient flow. Also, the patients are not willing to stay at the hospital for a long time, so it would be better if this was included in the liaison list and seen in the separate OPDs, otherwise it will be very difficult to dig out those cases from among all the patients.

R: Is it difficult to run the intervention at hospital level?

B06: No, it is very easy if there is one liaison -- one doctor and one nurse in separate OPDs.

R: is it time consuming?

B06: No, it does not take additional time. During working hours from 8:30am to 5:30pm the services can be given, except Saturday and Sunday.

R: The intervention suggests that soil-transmitted helminth infections should be confirmed through a stool concentration test. Is this a test you think can be performed at the hospital?

B06: Stool concentration -- I don't know whether this test is availble, but previously, when the regional laboratory was here, we requested this test. But now, we don't request it.

R: In your opinion, are there any problems with performing stool concentration tests at hospital level as suggested by the intervention?

B06: No, if supplies are available, iit is possible to perform the test.

R: What is the impact in terms of time and workload?

B06: Yes, yes, yes -- the same principle applies here. Practical training should be given for laboratory staff and one or two professionals should be assigned so the test can be performed.

R: The intervention suggests that patients diagnosed with soil-transmitted helminth infection should receive albendazole if the stool concentration test is positive for ascariasis & hookworm infection. Other drugs are recommended if the stool concentration test is positive for other worm infections. Albendazole should be given presumptively if it is not possible to perform a stool concentration test. Do you feel confident that you have the knowledge and skills to perform these responsibilities? In your experience, are the drugs required to perform this task normally available at this hospital?

B06: I don't have close contact with pharmacy staff, so I have no idea of these.

R: Okay, let's move to elephantiasis. Have yoy ever faced such a case? Do you know how health workers at hospital level should detect lymphatic filariasis?

B06: Yes, I saw a case of what we traditionally call elephantiasis. The patient came for other reasons though.

R: What were the signs and symptoms? How do you know whether it is this disease?

B06: The patient came for other reasons with symptoms of elephantiasis. It is differentiated by oedema of the leg with cracked skin and extra growths.

R: If a patient is diagnosed with lymphatic filariasis, do you know how those cases should be managed at hospital level? How did you manage this case?

B06: No, I didn't manage this case, except the existing complaint, because I faced it before I took the training.

R: Is that so?

B06: Yes, it was before the training.

R: Is it not possible to treat those cases at hospital level?

B06: Yes, they can be managed, but because of the workload, they need more concentration. Patients may not even come to the hospital with this problem. They usually come for other reasons. Still, if the nurses are active and observe and detect the problem, it can be identified and treated. At the hospital, these cases should not be missed, but as I said before, it takes time. I told you, it took us three to four days to detect the STH case, so at that time, the doctors gave more time.

R: The intervention suggests that patients diagnosed with acute lymphatic filariasis should receive pain relief with paracetamol, and be advised to hydrate and rest. All patients should be referred to a health post for advise on home management of lymphedoema. Patients with hydrocele should be referred to an appropriate hospital for surgery.Do you feel confident that you have the knowledge and skills to perform these responsibilities?

B06: I think the doctors can detect and manage those cases.

R: So you are saying there is no problem in both detecting and managing cases?

B06: Yes, they can detect and manage the cases. **[interruption due to a knock on the door]**

R: What do you generally think of the idea of tasking health workers at primary health care level (including health posts and health centres) with detecting, managing, recording and reporting neglected tropical diseases? Do you think the primary health care system has the capacity to take on these roles and responsibilities? What kind of challenges do you foresee?

B06: At health centre level, they need to have doctors and equipment to manage. **[Name of hospital]** *and the health office fulfil this. It would be good if they* **[health centres]** *can perform these roles, so there should be a system for that.*

R: Do you think health officers can handle those cases?

B06: Yes, health officers can handle the cases.

R: Do you think there would be any challenges with regard to gender? Would women and men face different challenges in accessing care for NTDs at the hospital? And what about associated stigma for example your case she was comong for other reasons.

B06: Gender?

R: For example, the sex of your patient and the sex of the doctor. What was the patient's reaction and what approach did the doctor take?

B06: The patient was female and the doctor was male. She was ashamed, tried to cover herself with a cloth, and she was talking more with me than with the doctor. The community

stigmatised them because it is considered as a disease caused by evil spirits or a hereditary problem. But it is up to us to treat and comfort the patient.

R: So there is stigma?

B06: Yes , they are stigmatised even by family members who do not want to stay with them.

R: What could be done to address those challenges?

B06: I raised this issue even during the training. A lot of work needs to be done in the community. We have to use the media. For example, we are talking about TB and STI on television, so the same principle can be applied to NTDs -- about the signs and symptoms, when to visit the health facility. Just like with leprosy and HIV, we have to educate on NTDs – how they are transmitted, stigma of considering them as hereditary problems or caused by evil spirits. Films could be produced. All levels -- hospitals, health offices and health centres, but especially health posts should consider those diseases as a major problem. It is very important that more consideration should be given to the rural areas.

R: What would your recommendations be with regard to integrating the detection, management, recording and reporting of NTDs into primary health care?

B06: Train the Health Extension Workers and use them as to raise awareness. First of all, Health Extension Workers can do their work home-to-home and secondly, the health posts are accessible to the community, so they are the best option for raising awareness of different issues around NTDs, because the patients are not coming to the hospital. Also, leaflets can be prepared and distributed.

R: What would your recommendations be with regard to integrating the detection, management, recording and reporting of NTDs into primary health care?

B06: The Regional Health Bureau should work on this. They have to consider the problem and build the capacity of hospitals and health centres. NGOs like your organisation **[Malaria Consortium]** *should design a system and give regular training as needed. The system should be integrated. Documents and materials should be available. We are only focusing on high burden killer diseases, but still NTDs also are causing disablities.*

R: Morbidity.

B06: Yes, significant morbidity, so our focus should include them.

R: Is there anything else you would like to share with the research team?

B06: As a reasearch team, it is fine, because sometimes some NGOs start something and then just leave it. But you are not like that. After three months, you come again and follow-up on the programme, which is unusual and good and it should be continued. Regarding **[name of hospital]***, including the adminstrative body, they should be included in this activity. I think the study will go ahead in a good manner and then afterwards, we want to see the changes.*

Health worker, hospital

R: First of all I would like to thank you. On the month May of this year the training was provided in the class room. Have you participated on the area of neglected tropical diseases?

B10: Yes I have been one of the training attendant fro those selected from Hospital.

R: Could you tell us as what you learned from the training provided?

B10: What I learned from the training is about neglected tropical diseases like trachoma, schistosomiasis, around podoconosis and how we manage when this kinds of diseases happen and due to neglect, there are so many problems happening. We are informed about not properly controled and at the level of the Hospital, we have to change the previous way of managment. We agreed to bring change on this with our two days training.

R: It is good. What did you find interesting from this training?

B10: From the training, actualy the training was for two days. It was short time and even if the training was conducted within short period of time, the traing idea was very good, except practical problems. And if we are trying to work like this, it is not difficult and for me, I am comfortable with the training.

R:You are said as you have attened training on the tropical diseases. Do you remember the diseases you learned?

B10: From around the 10th, I think we learned on five identified dieses. They are soil-transmited diseases, schistosomiasis – intestinal schistosoma and urinary divided into two, and also podoconosis and one is eh [...]

R: Oky it is very good. You mentioned you were trained. Which part did you like from the training?

B10: Normally, even if it is no diffirent from what we are doing routinely [...] Like trachoma and something like that. And the others also [...] Good. It was interesting.

R: It is good. When the training was conducted, there are two things that were expected and you already mentioned what you liked from the training. And probably you have some uncomfortable part of the training?

B10: Eh, the training was conducted -- I actually coudn't raise this point on the spot during the training, but it was not attractive for me, because of those people assigned as trainers for the training. Except the one from Malaria Consortium, the red one. There were two others and also the one from laboratory department assigned as trainer. That was not attractive and what we are expecting. The problem was that there was nothing nothing but reading the PowerPoint.

R:Good. If this happened, what point do you have to be improved related with the training?

B10: Related with the training, the processes to be improved -- I think as I have already mentioned -- to be added -- it will be practical things should be included, like case scenario practice and, I dont know, the training day was short, but for me, during the training, this should be included. Otherwise, it is good. Because I attend different trainings, so it is possible to do the training in an attarctive way, even to practice on the managment part by assigning from the particicipants for practice. It will be good to make the training become attractive and if these things are improved, the training will become attractive and easy to undrstand. It is good in my opinion.

R: It is good. After you were trained, do you think that this training is important for you?

B10: The training is based on our work. It is very important.

R:Why it is important?

B10: Because we give less focus on these things in our work and we can't see deeply. But they **[NTDs]** *are easy to manage and after what we have trained and practiced -- it is very important, I think.*

R:It is very good. Okay, after the training on the detecting and managing of neglected tropical diseases, what role do you have?

B10: First of all, I have a gap on the practical part of this training because I came from the Liaison Unit and I had no communication with the patients. And now also I am working in Emergency centeral triage and even if if we have this kinds of cases, we send them back to those professionals assigned for treating. There is no case I have seen while working in the Emergency Room where we did not refer.

R: You identify and then you refer.

B10: Yes. If I have these kinds of cases, we can refer to -- even if the Triage is closed, we have no right to manage except giving painkillers like paracetamol. For this reason, we are not working according to the training process.

R: You were provided with job aid materials, which help you to identify and manage the tropical diseases. What kinds of job aid you have?

B10 Normaly I have two types of job aids about the management part. ***B****ut at that time, they promised us the job aid will be distributed, but there is no distributed job aid for me. I have one and it is in my home, but in my work place, these kinds of things are not available.*

R:There is no job aid in your office?

B10: No job aid in my office for neglected diseases. I only know about my own work place. For the others, I don't know.

R: To remember the traing and help with tretment NTDs – do you have this job aid and did you receive these materials?

B1O: Yes.

R: But they are not practical?

B10:Yes. I received them during the training, which was individually given for training purposes, but I cannot put the job in our room and it is also not practical.

R: May you were informed during the training. Can you tell us how to use the job aid?

B10: The usage is -- it has eh -- there is something in the job aid about the management part, supported by pictures. How to manage, eh, lymphatic filariasis, something like that. What we are doing and the steps and what kinds of methods we use for the same age and how to apply that. It is indicated by number and we must follow the job aid accordingy, but currently I don't have it at hand now.

R: If there is a case of fever that is not malaria, what would your management related with the job aid look like?

B10: Eh, okay, if it's a febrile case when they come for traetment -- if there is no malaria? Only with fever? Eh, related with the job aid?

R: Maybe this one is related with fever caused by an NTD.

B10: Normaly we can't order BF **[blood film]**. *If we are suspecting filariasis, we can send, but as already mentioned, if there is redness and swelling on the lower extremities, we can send, depending on -- to send for BF.*

R: Good. Do youthink this job aid is easy to use?

B10: The job aid is convinent to use, but on the HMIS report part -- the time is short during that time. Other than the HMIS part, the other steps are convenient to me, but the **[HMIS]** *code part I did not understand during the training***.**

R: What are those difficult points to understand?

B10: For example, report in HMIS any soil-transmitted -- they are available on the side and I had traing before on HMIS, but this coding part, when the training was given, during that time it was not practical for me to digest and understand the knowledge. For this reason, the HMIS part was a little bit difficult for me.

R: Good. What do you think this job aid materials has regading the amount of information.Is it too much, too little or is it appropriate?

B10: Normaly, regarding the preparation for management, it is enough in my opinion

R: You said it has enough information?

B10: Yes it has enough information, especialy on the management part.

R: Do you find those job aids easy to understand?

B10: The main thing is, I am trained on it. But I'm not using it. But if I use it, it is simple because we are using – with this system, managment is very simple, because I know about under five management. For this reason, it is easy and convienent to use in my opinion.

R:For this reason, except you are not using it, it is easy to work with the job aid, according to the knowledge you have from training?

B10: Yes, it is very important.

R: Okay, if it is very important, why do you say so?

B10: The importance is to solve the problem which is happening and if I use it properly, I have a good result. Because it tells me what are the signs and what are the problems for this kinds of problems. What kinds of laboratory we need and with what tretment we treat and order the drug – these are all things available in it. I don't need to go anywhere to find everything. It is there. Even from the other system we are using, it minimises our mistakes. For this reason, it is very important.

R:Do you think the job aids give adequate concideration to other diseases that are not neglected tropical diseases?

B10: Normaly, as we have seen, there are not only five, but there are more than five. But more commenly seen or happening in this area, if they are selected, it is helpful. But it is better if it is wider, also for the other diseases. Based on the available one, it is enough in my opinion.

R: It is good. With regard to job aid usage, do you think health workers might over- or under-diagnose neglected tropical diseases?

B10: I don't think doing assessment by using this job aid has a problem because for what we found [unintelligable] -- already mentioned, for this reason, by sending for different additional laboratory tests. I don't think this kind of over- and under-diagnosis problem is happening.

R:How could the job aids be improved?

B10: About improvement -- as I have said before, practically, I am not using it. And I don't blindly want to comment about improvement. But as I have said before, those things I mentioned -- it needs to be more focused is what I wont to say.

R: I want to ask you, related with detection and management, from the training, how the health workers in this Hospital should detect and manage this six diseases:Trachoma ,Urinary Schistosomiasis, Intestinal Schistosomiasis, Soil-transmited helminth infection ,Lymphatic filariasis and Podoconosis. Did you ever see patients with signs and symptoms of any of the diseases?

B10: Eh, normally, with these kinds of diseases, like filariasis and podoconosis, I have no exposure to these kinds of cases. Ehh, I think Trachoma cases I have seen, even if we discussed this things during the traing. At the Hospital, these kinds of things are done by specialists, even if you can get this information. If you are asked as the centeral triage health worker -- because they send them back to each department for treatment. If those patients having Trichiasis – they normally have itching, redness – those patients are normally sent back with a referral to another Hospital that has this service related with this complaint. I don't have any information about these kinds of cases starting to be managed. I have no information, but when we have these kinds of problems with the eye, they are sent back.

R: You have mentioned you have seen Trachoma case management. Have you seen other cases?

B10: No, I haven't seen, but sometimes, outside the hospital, I have observed lyphatic filariasis and no other cases coming for treatment, in my experience. Also, I have only worked in this department for two months. Within this period of time, I coudnt see these kinds of cases.

R:Do you know how health workers in this Hospital level should detect trachoma?

B10: First, starting from whether the area is at risk, we take a history. And if expossed, for these kinds of diseases -- for example, like eye discharge, eehh, like trichiasis, cornial clouding. And if these things are happening and it has an effect, we can see if they have redness, discharge -- and if the eye lid is inverted to the inside, we can see this.

R:If you suspect that a patient has signs and symptoms of Trachoma, what kinds of examination do you do for diagnosis? In addition to the syptomps for diagnosis you already mentioned, we can do examinations. And what kinds of lab examinations do you need?

B10: Ehh, laboratory?

R: Laboratory. What kinds of examination do you need?

B10: I have more of a clinical -- because I know it is treated clinically.

R: If a patient is diagnosed with Trachoma, do you know how those cases should be managed at Hospital level?

B10: There is medical management and surgical management. For this reason, on the medical part, at community level – eh, antibiotics like azythromycine and if the case is at the stage of Trachoma trichiasis, it will be managed by a surgical intervention. But in our set-up -- I don't know. In my experience, I have seen that this is done at the health center level.

R: It isn't done here in the Hospital?

B10: It is not done here in the hospital.

R: For this reason, where did you send them?

B10: We send them to the referral hospital if these kinds of cases happen. And I know that all cases -- like if different cases are sent to related professionals, we also send them to the referral hospital. I know this is reccomended for all cases, except the medical cases, like conjunctivitis. The other severe eye cases are referred to the hospital. This problem also happens in the central Triage. It was asked during the training and we expect a practical change, but there are no activeties provided that I know of.

R: Would you recommend any kind of follow up if you have identified these kinds of cases?

B10: I havent seen these kinds of cases after they are once sent back. There is no follow-up because the procedure is not done here.

R: As neglected tropical diseases are detected following the signs like redness of the eye, mucopurulent discharge ,eye pain or inverted eye lashes,further examination of eye lashes, eyeball and conjuctiva, health workers should be able to diagnose trachomatous inflamation or trichiasis. Do you feel confident that you have the knowledge and skills to perform these responsibilities?

B10: We know the symptoms properly, but because the service is not provided here, we can't manage those cases here.

R: Are you confident to treat if you have these kinds of cases?

B10: Yes, I am confident enough. It is simple to manage after detection of the case for me.

R: What gives you the confidence?

B10: The first thing I have to be confident is after these things are identified and the provision of training on these cases -- one of the reasons -- and supporting these activities with preparing these kinds of job aids is increasing the confidence. It has the effect to be confident.

R: In your opinion, are there any problems with tasking health workers at hospitals with detecting trachoma as suggested by the intervention?

*B10: There is no problem. If the implementation is done with these job aid. But the majority of the health workers doesnt use this. But if we are workin following these, there are no problems in my opinion***.**

R:What are the implications of performing this task in terms of time and work load?

B10:Using these job aids is saving time and also decreases workload, because everything is well mentioned on the job aid. For this reason to perform these activities, it decreases time and workload in my opinion. There is no better option than using this option.

R: Do you think this approach results in reliable detection of trachoma?

B10: Yes, this is the best option for me to detect trachoma, in my opinion.

R:What alternatives or improvements would you sugget on the trachoma treatment?

B10: Regarding treatment, it is as it is, but regarding our activities, the problem is available trachoma cases. in my opinion, in the Hospital set-up if it gets attention. More focus to do these trachoma management activities and doing them properly will be good is my sugestion.

R:The intervention suggest thats that patients diagnosed with trachomatous inflammation should be receive azithromycine. In addition patients with trichiasis should be receive surgery.Do you feel confident that you have the knowledge and skills to perform these responsibilities?

B10: Regarding medical treatment, I have confidence because I know what is needed. But regarding the surgical intervention, if supported with training, it is easy to perform even if I have no training on surgical management. I have seen when trained professional do this at health center level for trachoma trichuriasis and if supported by training, it is easy to do.

R: What gives you confidence?

B10: Because I have seen when trained Nurses are doing the proceedure at health center when I worked at a health center. For this reason, if I'm supported with training, it is easy to perform.

R: Have you seen patients with signs and symptoms of trachoma recently? Did you proceed with them as suggested by the intervention?

B10:I haven't seen recently this case.

R: Is there any problem problem with tasking health workers at hospital level with managing trachoma as sugested by the intervention?

*B10: This activity is done at the health center level and this hospital and if they are managed, there are no problems. It helps the patient, in my opinion***.**

R: What are the implications of performing this task in terms of time and workload?

B10: Regarding man power, our hospital set-up is not convienent. If we see the emergency department, the load is very high and for this reason, problems may happen related with man power. At the health center, there was time and space, but now the hospital set-up is overcrowded.

R:What do you think related with work load?

B10: The workload here, it is very high in our set-up.

R: In your experience, are drugs,infrastracture and equipment to perform this task normally available at this Hospital?

B10: Regarding trachoma, as we have no Eye department, there aren't that many materials available. Of course regarding medicine, there is no gap in my opinion. For those problems, we have drugs.

R: Related with surgical intervention?

B10: Surgical intervention is not done here and this is the gap we have regarding surgical materials. There is a problem.

R:Do you think this approach result in good outcomes?

B10: I thik this approach gives confidence, beause as it is prepared according to the management protocol, it gives confidence. When we compare it with our country treatment guideline, it seems extracted from that guideline. For this reason, there are no problems. It gives confidence.

R: As you mentioned different problems above, can they be solved?

B10: It is possible to solve this problem.

R: What kinds of solution methods do you have?

B10: As we said, providing training only doesn't solve the existing problem. After the training , it needs follow-up and supervission to start up. They said that data will be collected within one year and the training was given in May. After that, I have not seen anything. Even though they promised to come, no one came to support this activity after the training.

R: Do you know how health workers at hospital level should detect urinary schistomiasis?

B10: Urinary schistosomiaisis will be more found at the laboratory department and they said something during training.

R: What signs and symptoms would you look out for?

B10: They have haematuria. Eh, they have lower abdominal pain.

R: If you suspect that a patient has signs and symptoms of urinary schistosomiasis, what would you do to reach a diagnosis? For example, as you said just now, if they have haematuria?

B10: Like urine to the laboratory?

R: You are sending to the laboratory for examination?

B10: Yes, we can.

R: What kinds of laboratory tests do you do?

B10: I am not focusing on this part. That is why I am trying to remeber -- like concentration method -- I remember the steps – ehh, stool and urine concentration method.

R: If a patient is diagnosed with urinary schistosomiasis ,do you know how those cases should be managed at hospital level?

B10: If they come with these kinds of problems, the best treatment is praziquantile. I think this is howit is managed.

R:Under what circumstance would you refer the patient? Where would you refer the patient to?

B10: Regarding schistosomiasis, normally I haven't seen this kind of case.

R: Which kinds of cases need referal and where are they referred?

B10: Those who have any obstractionand if neurologic evaluation is needed will be referred.

R:Would you recommend any kinds of follow up?

B10: I dont think there is a follow-up because no case has been found with this condition. I am only answering about the drugs based on your questions, but in this department where I work, we have seen those medial and surgical cases for follow-up, but I have no exposure to this case.

R: The intervention we are testing suggests that health workers at hospital level should detect urinary schistomiasis based on the following symptoms: in children up to five years:blood in urine; in children over five and adults: blood in the urine, pain while urinating, pelvic pain, genital itching or burning sensation, involuntary urination when coughing, laughing or jumping. Do you feel confident that you have the knowledge and skills to perform these responsibilities?

*B10: Based on signs and symtoms, I believe that I have knowledge on it***.**

R: If you do, what makes you confident?

B10: Around this point, to be confident **--** *In addition to our medical knowledge, we have received training on schistosomiasis. And we already discussed -- as you raised it about this knowledge. It is enough in my opinion.*

R: In your opinion, are there any problems with tasking health workers at hospital level with detecting urinary schistomiasis as suggested by the intervention?

B10: Yes, I think there is a problem because with these conditions, when the patients are coming, we might relate them with urinary conditions like UTI **[urinary tract infection]** *rather than schistosomiasis, There may be this kind of problem to detect schistosomiasis in my opinion***.**

R: What are the implications of perfrming this task in terms of time and workload? Regarding the assessement of schistosomiasis?

B10: Normally, this is our routine work and I don't think it adds workload.

R: What about related with time to do the examination?

B10: Based on our set-up, I dont think it takes time. If we stick to the manual, it doesn't take longer.

R: Do you think this approach results in reliable detection of urinary schistosomiasis?

B10: Yes, I belive this approach is reliable in my opinion.

R: How could the problems you mentioned be addressed?

B10: Okay, where we have problems or gaps, this will be solved if we are strongly engaged in the activities. We can solve them. Where there is no additional support, it will be solved by using our own efforts.

R: How can we solve them? Do you have any suggestions?

B10: Regarding schistosomiasis, the first thing is following this manual and be strongly engaged. That is one of the solutions, as it is possible to do these activities in our hospital set-up. There is no other solution rather than work hard in my opinion.

R: The intervention suggests that a urinary schistosomiasis diagnosis should be confirmed through a urine sedimentation test. Is this a test you think can be performed at the hospital?

B10: It is possible to do this test in the hospital. We discussed this with laboratory professinal during the training. He informed us that the materials will be available after this training as we start these activities. For the others, I dont know, but in this hospital, to do those activities seems easy to me.

R: Do you have knowledge and skills to interpratet the labratory result?

B10: He mentioned the report part, but I wasn't focused, so I missed it. But the technician was presenting how we are reporting and what is reported. The lab technician was informed during the training. He was clearly explaining, but as I have said to you, in this case I have no exposure, so can't say anything about this activity.

R:Would you request this test if it could be perfoermed?

B10: Yes I can reqest because that examination is needed as per the manual guide, so I would request a sedimentation test. This is what should be done.

R: Do you think the test results are reliable?

B10: The result is very effective compared with the previous test.

R:Are there any problems with performing urine sedimentation tests at hospital level as suggested by the intervention?

B10: I don't think so. If the materials are available, the professionals are already performing this test. Almost all took this training. So there is no problem in my opinion.

R: How could the problems you mentioned be addresed? What alternatives would you suggest?

B10: Normally if this test is there in the laboratory, but if the General Practicioners are not requesting this test, they can't do it and the lab technicians can't practice.

R:The intervention sugests that patient diagnosed with urinary schistosomiasis should recieve praziquantile if the urine sedimentation test is posetive.patient over five years with any of the symptoms of urinary schistosomiasis should be given praziquantel presumptvely if it is not possibleto perform a urine sedimentation test,Do you feel confident that you have the knowlwdge and skills to perform these these responsibilities?

B10: According to the traing I have recived, I do have that knowledge in my opinion.

R: What gives you the cnfidence?

B10: The first thing is recieving this training. That's what makes me confident in my opinion.

R: Is there anything else that makes you confident?

B10: I don't have anything else to mention.

R: Have you seen patients with signs and symptoms of urinary schistosomiasis recently?

B10: No , I haven't seen this kind of case.

R: Is there any problem with tasking health workers at hospital level with managing urinary schistosomiasis as suggested by the intervention?

B10: I don't think there is a problem. Because detailed information is mentioned in the right place and on the right way -- what kinds of drugs, what dose, all mentioned accordingly. And in addition to that, we are professinals and together with this training, we are able to manage in my opinion.

R: What are the implications of performing this task in terms time and workload?

B10: In my opinion, we are already doing this activity integrated with our routine work. For this reason, I am not expecting that it adds workload or takes more time.

R: According to your experience, are drugs and equipment required to perform this task normaly available at this this hospital?

B10:The drugs may be available, but I don't have information regarding the laboratory materials and I haven't seen or sent a request for the laboratory test.

R: How could the problems you mentioned be addressed? What alternatives or improvements would you suggest?

B10: As I have said before, I dont have any additional information.

R: What do you generally think of the idea of tasking health workers at primary health care level with detecting ,managing ,recording and reporting neglected tropical diseases?

B10: At health post level, if they are tasked with this, they are able to identify and send to the health center. For this reason, the health posts have a great role in my opinion because they have day to day relation with the community as they are doing home to home activities. If

*they find a case, they are immediately sent to the hospital or health center. For this reason, I am expecting there will be a great change with this implementing programme, because insted of working here, the Health Extention Workers are working home to home activities. I suggest that working with them brings a big change in my opinion***.**

R:Do you think there would be any challenge with regard to gender? Would women and men face different challenges caused by NTDs at the hospital*?*

B10: If the activities are not properly run, stigma may be a challenge. It can cause people not to seek care services in my opinion.

R: Related with gender, do you think there are challenges at the hospital?

B10: If the activities are started and they are coming to get services at the hospital, they will not face any challenges. But with these conditions -- like when the patients come to get services and we are sending them back with referral, they may have a challenge. But if the examination is done here, I think they won't have a challenge.

R: What would your recommendations be with regard to integrating the detection, management, reporting and recording of NTDs into primary health care?

B10: The network should be strong and must be wider. The hospital also needs to supervise the health centers accordingly to discuss the necessary points. Also the health center will suopport the health post. This way everything will be integrated.

R: It is good. As you discussed with us for a long time, finally, do you have any comments for the research team

B10: The process of the training, as I have already mentioned before, it brings change and it is good and will be supported in my opinion. This is all. I dont have any additional points.

R: Thank you for your time.

Health worker, hospital

R:Have you heard of the classroom training that was offered to health workers from your hospital about NTDs? Did you attend this training?

B34: Of course I heard about it. But those who attended the traing didn't share any information about the traing and also they are not bringing the hadouts they received at the training. If they bring the manual, I will try to read it and get the information. But becasue they are not bringing the manual, I don't have any information about the training. I want to share with you my previous experience of training. If any professionals reciev training, they

must share the information about the training with their friends. For this one, I don't have any information about this training. They just went home after they were trained.

R: So for this reason, you don't have any information about this training?

B34: Nothing. I don't have any information about the training.

R: It is good. Maybe about neglected tropical diseases -- do you know about neglected tropial diseases?

B34:I know about these neglected tropical disease from school, but I have no knowledge from anywhere else. Other than the knowledge from school, I did't get any information from those who were trained. I heard about them just now when you were talking about the disease.

R:It is good. Those who were trained were provided job aid for neglected tropical diseases. Have you seen them using this job aid?

B34:I don't have any information about this job aid. I did't see those materials.

R: It is like a manual.

B34: It is a manual?

R: Yes, like the IMNCI chartbooklet.

B34: Okay, like IMNCI.

R: This manual is used for for detection,treatment and management of neglected tropical diseases. Have you seen these materials?

B34: I haven't seen them because I have no information about it.

R: Good. We would ike to discuss NTDs like Trachoma, Urinary schistosomiasis, Intestinal, soil-transmited disease, Lyphatic filariasis and podoconosis. Do you know about this kind of diseases?

B34: Yes, about Trachoma.

R: You know about trachoma? You don't know the others?

B34: I know about [...] Elephantiasis, as you said.

R: You told me you know about trachoma. In your routine work, based on what signs and symptoms do you identify trachoma cases?

B34:Yes, by signs and symptoms.

R: What are the signs and symtoms of trachoma cases?

B34: They have discharge and it is communicable. I have seen one case of end-stage trachoma which causes disablity of the eye. He lost one eye and transmited to the other one. Butthanks to surgical management, his other eye was saved.

R: This end-stage case was managed surgically, you said?

B34: Yes. It was transmited from one eye to the other by flies.

R: As you said before, those cases discharge from the eye. What other signs do you know*?*

B34:Redness of the eye.

R: If these kinds of cases happen at this hospital, is it possible to manage these cases at hospital level?

B34: Yes it is possible.

R: How can they manage this kind of case?

B34: Based on signs and symptoms, the physicians know how to manage the cases and according to the management process, it can be managed. If they come early, the case will be managed.

R: If the disease is detected early, it will be managed*?*

B34:For this reason, personal hygiene needs to be followed and accordingly, proper health education needs to be provided. This is the primary prevention. And for secondary prevention, they need to get treatment early.

R: What kind of treatment do you reccomened?

B34: We can give eye drops.

R: Do you think laboratory examination is reccomended for trachoma?

*B34: No, it is not reccomended***.**

R:As you mentioned, you have seen one end-stage case. At what stage is surgical management reccomended?

B34: When they can't see, it is managed surgically. This must be detected early because I have seen a case who lost his eye. In my opinion, the community must have awarness of trachoma and personal hygiene and when cases happen, they must inform the Health Extension Workers and health centres. And the primary hospital, tertiary and so on. And those who work at primary level -- Health Extension Workers must do these activities. If they teach the community about trachoma -- it must be treated before it causes disablity.

R: Do you think surgical management is done here at this hospital?

*B34:It is not done at this hospital because there is no eye department here in our hospital. We send cases to the referral hospita*l**.**

R: Do you have the knowledge to identify and refer cases?

B34:Yes, I do.

R: So if you have this kind of case, what do you do in this department?

B34: First of all, we identify the disease by signs and symptoms .We are working here on the identification part and then send them to the physcians to be managed accordingly. And if a

case needs surgical management, we need to communicate to where the place is where this service is available and send the case through the the referral system.

R: It is good. Trachoma cases may have redness of the eye, mucopurulent discharge ,eye pain, inverted eye lashes. On further examination, symptoms affecting the eyelashes, eyeball and conjuctiva may be seen. Do you have the knowledge to identify this kind of cases?

B34: Yes, I can identyfy the discharge.

R: By obsering these signs and symptoms, is it possible for you to detect trachoma cases?

B34: Yes, I have this knowledge.

R: Good. What makes you confident?

B34: My school knowledge.

R: So you didn't get any related information from the trained colleagues?

B34: No one shared information about this with me. My knowledge is based on my school knowledge.

R: Did you try to get information from those trained colleagues?

B34: I couldn't try because they did not inform me about the traing.

R: It is good. If you have any trachoma cases, where do you send them?

B34: From this department to OPD.

R: You told me before that this kind of cases are not managed here?

B34: We send them to the referral hospital.

R: Do you think that asking health workers at hospital level to detect trachoma is important?

B34: Yes, the training is important.

R:Why is it important?

B34: As I told you before, thesd diseases are neglected. Soon after they have been trained, health workers must start to detect and treat those cases earlier. The training is important in my opinion and in addition to that, other professionals should also be trained. Knowing the signs and symptoms of the disease is helpful to identify where they need to be referred to. For this reason, it is good.

R: What kind of treatment is provided for trachoma? Do you remember the treatment?

B34: Yes, eye drops like dexamethasone,tetracyclene and eye ointment.

R: So befor surgical management, you treat with azithromycine and such drugs?

B34: Azythromycine – yes, it is given for a lot of diseases.

R: The intervention reccomends azythromycine for the stage of trachoma inflammation or trichiasis. Do you think health workers at hospital level can perform these responsibilities?

B34: Actually, there is no eye department here in this hospital. Still, trachoma cases can be managed except surgery. It is possible to manage them here. Actually, there is a lack of space. When the new building is completed, maybe this problem will be solved. Because different professionals are now sent for specialisation for different departments. Once all things are comleted, maybe it will be possible to manage all cases here in the hospital.

R: Do you know about urinary schistosomiasis?

B34: I know from school.

R: What do you remember about urinary schistosomiasis?

*B34: It is found though urine examination and the colour also***.**

R: How do you differentiate cases?

B34: White colour.

R: Do you remember any urinary examinations? For example, if there blood in the urine? And do you remember when this laboratory examinations are done here at the hospital?

B34: I don't know about this.

R:The intervention we are testing suggests that health workers at hospital level should detect urinary schistosomiasis based on the following symptoms :in children up to five years blood in urine;In children over five and adults:blood in urine,pain while urinating ,pelvic pain,genital itching or burning sensation ,involuntary urination when coughing,laughing or jumping.Do you think health workers at the hospital level can perform these responsibilities?

B34: Usually, when these kinds of cases happen, especially those older age groups, when we detect that their problem is related with UTI **[urinary tract infections]***, we send them to the OPD. Children we send to the paediatric OPD to be treated.*

R: What about females?

B34: Females also treated at the OPD.

R: The intervention suggest that a urinary schistosomiasis diagnosis should be confirmed through a urine sedimentation test.is this a test you think can be performed at the hospital?

B34: Yes, it is good to treating after detecting the disease. As I have said before, even though I have no information about the training, this process may help them to diagnose the real cause. You told me now that it looks like the IMNCI chartbooklet and it has signs and

*symptoms of each disease. So after identification of the diseases, we send to the laboratory examination. For this reason, it is very good in my opinion***.**

R: Do you think that you have a knowledge to interprate the lab result?

B34: As have said before, I have knowledge from school.

R: For different cases, there are result from the laboratory department?

B34: Which one? Actually, what do in this department, is to identyfy cases and send them to the physicians and then they treat them accordingly.

R: So you don't have a chance to manage based on the laboratory results?

B34: No, because this is mainly done by the physicians. Here in the liaison department, we identyfy the case and send them to the right department for treatment, including with the laboratory tests.

R: Related with urinary schistosomiasis, urine sedimentation test is suggested to reach the diagnosis. What do you know about this?

B34:I know it is done by lab examination, but I don't know how they do this laboratory test.

R: You don't have any information about lab investigations?

B34:No, I am not sure. If I had attended the training, I'd try to know what kinds of laboratory tests are available or done there.

R:In your experience, if these cases come to the hospital, do you think that there are drugs and materials available here?

B34: Drugs are available because our hospital gives services that involve drugs in three areas: community,inpatient and outpatient. At each level, drugs are distributed. Therefore, we dont have drugs shortages. It depends, of course.For example, if the cases of malaria are very high, drug consumption will also increase. And when these kind of cases come to the hospital in large numbers, they may have enough drugs in my opinion.

R: We talked about trachoma and urinary shistosomiasis. What about the other diseases like lyphatic filariasis, podoconiosisand elephantiasis? Have you seen these cases in your hospital?

B34: Yes, I have seen cases, but not in this hospital, but when I was in **[name of a hospital in another district].**

R: What kind of signs and symptoms do you know for these cases?

B34: For example, swelling of the leg, swelling of the reproductive organs. I have seen that they are given drugs, but there is no cure. But if they are managed early, it may stop at that

level. Again, elephantisis case are more in the rural area. There, they concider this as normal cases. A lot of cases come to the facilities with big swellings. That's what I have seen.

R:What kinds of treatment are here?

B34: **[unintelligable]** *and they remove the dry skin. They also give drugs so the swelling remains as it is.*

R: Have you seen these cases recorded and reported?

B34: No, I've never seen that.

R: Do you think this kinds of cases are coming to this health facility for treatment as you have seen in the other hospital?

B34: No I haven't seen them in this hospital.

R: As you mentioned, this kind of cases may have swellings of male and female reproductive organs and of the breast of the female. Do you think these cases are equally managed with regard to gender?

B34: I think male cases are treated earlier than females because most of the time, females tolerating even if they have a lot of diseases, especialy in the rural area. This may be due to gender violence in my opinion. I have seen that of those that come to get services, almost all are male.

R: Do you think if they come to this facility, might they be discriminated against?

B34: No there couldn't be this kind of discremination because males and females are managed equally. But in my opinion, this may happen in the community.

R: Why might the community discriminat against the people with these diseases?

B34: Because of poor perceptions relating to spiritual reasons, as it may come from God, But as far as the treatment is concerned, there is no discrimination. They treated equally qt the hospital level.

R: Do you have any helpful ideas you want to share with the research team?

B34: My recomendation is that all the trained staff must orient those not trained. Otherwise there might be a treatment gap between those who were trained and those who weren't. The other thing is that they must share the manuals they received during the training. This will be helpful in updating our knowledge and service provision will also be improved. Increasing the acceptance of the services we provide will be hepful in addressing these kinds of diseases.

R: Earlier, you mentioned problems regarding medical materials. Do you have any opinions on how to solve those problems?

B34: Regarding the eye department, the professionals there are sent to school. When they come back, the problem may be solved. Completing the building is also going to help to open this department. Then surgical management can be started and the rest is just managing based on signs and symptoms in my opinion.

R: Thank you for your time.

Health worker, hospital

R: In May this year, classroom training was offered to health workers from your hospital about neglected tropical diseases. Did you attend this training?

B37: Yes I have attended the training.

R: Can you tell me what you learned in this training?

B37: From the training I learned so many things. Starting from the name, the training covered really neglected health problems. These diseases are covered during our formal study. They are common but both the government and the health professionals do not consider them as a main health problem. For example Podoconosis. Both while I was a student and even after graduation, in our routine work, our concern for these diseases was very low, so the training gave a good update. This applies not only to podoconosis but also to other diseases that are included in the NTDs.

R: What did you most like in the training, both in terms of diseases and the method of delivery of the training which helps you remember about those diseases?

B37: I think it is two months ago that we took the training. Yes, there are points I liked. The best thing was the training delivery system or approach. There was some sort of demonstration. The training was intractive. Regarding the diseases, it was about neglected problems, which is also a community problem, but neglected by health professionals and the government.

R: Was there anything you didn't like about the training?

B37: The thing I did not like was the implementation part after the training. After we started our work, the things were not like the training.

R: You mean the implementation part?

B37: Yes. The implementation part almost did not start. Rather, it was just taking the training. So the training organisers should give attention to the implementation part as well as the training. The ogranisation who took responsiblity should provide important materials and better support with supervision. I do not think there is implementation of the project as far as I know.

R: Okay. What do you recommend other than supervision to ensure implementation starts and is done?

B37: For the work it would be good to ensure the materials are in place. Also, there should be routine contact with staff, which is to say supervision. If there is a gap, it should be closed in a timely fashion. In my opinion, this will help the implementation to be fully practiced.

R: After the training, what is your understanding of your role with regard to detecting and managing NTDs?

B37: Yes, at that time, I understand my role in detecting and managing the diseases, but implementation was good enough. Now I changed my responsiblity to pediatric OPD **[Outpatient Department]***, but when I was in the adult OPD, there were 2 or 3 cases of trachoma I detected and managed based on the instructions of this organisation. But here, there are no cases of tracoma. However, STH cases are common.*

R: Were you given any materials during or after the training to help you remember what was taught and to help you detect and manage NTDs?

B37: Yes I had recived training materials and obviously these are helpful even if I do not use them to detect and treat cases. However, I am using them as a guide and reference materials.

R: Have you ever used the job aids and can you show me how to use by taking a single disease as an example?

B37: I faced some cases of trachoma.

R: Trichiasis?

B37: Where do I show?

R: In the job aids.

B37: *Okay, where are trachoma is in the job aid?*

R: It is at the beggining of the table [I showed him the table]. For example, a child comes with redness of the eye. What steps do you follow to reach a diagnosis?

B37: Okay, I will take the history using, then do physical examination and assess the risk factors and then grade the stage of trachoma. Once you have reached the diagnosis by saying TI, TF, TT, CO and so on.

R: Those are the stages of Trachoma?

B37: Yes, staging the Tracoma and finally treat them based on the stage medically with antibiotic if active infection and if it is TT, I will refer them.

R: Does TT mean trichiasis?

B37: Yes, it means trachomal trichiasis. We refer them for surgical managment to referal **[referral hospital]***, but it is not fair. In the general hospital, the surgical managment is very simple, but because the materials are not available, I referred two cases. I did not write my name on the referal paper because I was ashamed to refer those simple cases.*

R: Trichiasis.

B37: Yes, Trichiasis.

R: Do you think the job aids are enough to detect and manage cases of NTDs?

B37: The jobs aids are enougn to detect and manage cases. The problem is the materials for some of the diseases. So there is nothing I want to suggest in terms of additions to the job aids, except the materials for managment of cases.

R: Do you think there is a risk that health workers might over- or under-diagnose neglected tropical diseases if they follow the job aids?

B37: Sorry, I hav not checked the job aids in much detail.

R: The training and the materials that were distributed provide suggestions for how health workers at hospital level should detect and manage six diseases: trachoma, urinary schistosomiasis, intestinal schistosomiasis, soil-transmitted helminth infection, lymphatic

filariasis and podoconiosis. Do you ever see patients with signs and symptoms of any of the diseases?

B37: Yes I face trachoma and STH. Especially STH is very common.

R: So you have more cases of STH?

B37: Yes I face STH, but for me it is important not to consider STH as a "neglected" disease because almost everybody faces this problem in my opinion.

R: Which diseases do you mostly encounter other than trachoma and STH and which NTDs have you never faced?

B37: I have never faced podoconiosis yet.

R: Is it because there are no cases or they are not coming to the health facility?

B37: I think the cases are not coming to the health facility because there are visible cases in the community.

R: Oky is that so.

B37: Yes, that is the case.

R: Do you know how health workers at hospital level should detect trachoma?

B37: Yes the staff have the capacity to detect NTDs including trachoma well.

R: If a patient is diagnosed with trachoma, do you know how those cases should be managed at hospital level?

B37: *Yes, for acute cases, medical treatment is available, but for surgical interventions, even if there are staff with the ability to perform it, we are referring to the referal hospital because of lack of materials.*

R: Do you have professionals who can handle the surgical treatment?

B37: Yes, we have one opthalmic nurse who can perform it if the materials are available.

R: Is there any follow-up?

B37: The patients who had medical treatment will receive follow-up to check for improvment, but for surgical patients, because we are not treating them, follow-up is not given.

R: The intervention we are testing suggests that health workers at hospital level should detect trachoma based on the following symptoms: irritated red eye, mucopurulant discharge, eye pain or inverted eye lashes. On further examination of eyelashes, eyeball and conjunctiva, health workers should be able to diagnose trachomatous inflammation or trichiasis. Do you feel confident that you have the knowledge and skills to perform these responsibilities?

B37: Yes I can confidently manage cases of trachoma mdically. The confidence comes from my formal education. Also the training updated me, so I am confident enough.

R: In your opinion, are there any problems with tasking health workers at health hospital with detecting trachoma as suggested by the intervention?

B37: Yes, the hospital is very crowded even for major public health problems, so maybe infrastructure will be one issue, but still we can try to perform with the available resources. The job aids are reliable to detect cases of NTDs.

R: Even though you said there is no problem regarding the job aids, what alternatives or improvements would you suggest?

B37: For those NTDs, I do not know, but for STH, it is a long lasting problem and for me, they are not neglected. But I think we need another strategy to reduce them. I know there are NGOs and governmental ogranisation in Sidama Zone, who were working on those problems, so we may think of other strategies to solve the root causes of these problems other than this intervention. I always think it maybe because of our life style or something elese.

R: The intervention suggests that patients diagnosed with trachomatous inflammation should receive azithromycin or tetracycline. In addition, patients with trichiasis should receive surgery. Do you feel confident that you have the knowledge and skills to perform these responsibilities?

B37: Yes, I can perform the surgical procedures. I used to perform the procedure when I was in the health center. My confidence is because of my formal education, but not the training. The training did not include surgical managment of trichiasis.

R: In your opinion, what are the implications of performing this task in terms of time and workload?

B37: Why do we think about it like this? I do not think so. This is a community problem, why do we raise the time issue? It is an issue of medical care and our responsiblity. Only we can schedule and arrange the program and I do not believe extra time should be an issue. If we give full treatment, this will help to improve outcomes for the patients and we will have better confidence in the interventions. If the materials are available, I suggest the surgical intervention should be the part of the training and adquate professionals will be available in the managment.

R: Do you know how health workers at hospital level should detect soil-transmitted helminth infections?

B37: Yes it is very easy to detect and manage STH. First we will see the signs and symptoms, for example abdominal cramp, diarrhoea and so on. Then physical examination will be done and laboratory will be sent. The problem in the hospital is that we only have the direct wet mount stool examination test. But if we had the sedmentation test, it would be better. Even if the results are negative **[of the wet mount test]**, *I personally treat the patient based on signs and symptoms.*

R: Would you request a stool sedmentation test ever?

B37: No, because the laboratory department has complained about the workload and the time it takes to test. Even during the training, so I have never requested it. All STHs can be treated with medical treatment and they are usually detected by clinical method.

R: Under what circumstances would you refer the patient? Where would you refer the patient to?

B37: *No referal needed for STH, except for intestinal obstruction, but I have never faced such a case.*

R: Would you recommend any kind of follow-up?

B37: *STH cases need follow up because it may cause malnutrition, especially in children, so they need a follow up visit.*

R: The intervention suggests that soil-transmitted helminth infections should be confirmed through a a stool concentration test. Is this a test you think can be performed at the hospital?

B37: Yes it can be performed in the hospital and it is the best method of diagnosis, but because of the workload it is not possible. So I suggest that this tests should be available for better diagnosis and treatment. It would be good if you communicated this to laboratory staff.

R: The intervention suggests that patients diagnosed with soil-transmitted helminth infection should receive albendazole if the stool concentration test is positive for ascariasis & hookworm infection. Other drugs are recommended if the stool concentration test is positive for other worm infections. Albendazole should be given presumptively if it is not possible to perform a stool concentration test. Do you feel confident that you have the knowledge and skills to perform these responsibilities?

B37: Yes, I can perform them confidently because of my basic formal education. I face STH cases on a daily basis, but I did not use the intervention tools provided by your organisation.

R: In your opinion, what are the implications of performing this task in terms of time and workload?

B37: *Detecting and treating STH will not have an impact on time and workload because these are routine problems, even befor we took the training. The drugs for managment of STH are available in the hospital. Treatment of these cases will improve the outcome of cases and prevent complications.*

R: How could the problems you mentioned be addressed? What alternatives or improvements would you suggest?

B37: My suggestion is about the use of laboratory stool concentration.

R: What do you generally think of the idea of tasking health workers at primary health care level (including health posts and health centres) with detecting, managing, recording and reporting neglected tropical diseases?

B37: Health centers and health postes can perform on NTDs. I do not think there will be a problem or it will be challenging for them. They actually may need capacity building and supervision in the detection and handling of cases.

R: Do you think there would be any challenges with regard to gender? Would women and men face different challenges in accessing care for NTDs at the hospital?

B37: I do not think tere will be a problem in treating patients of both sexes. Even if I don't have evidence to say that, but females are not coming to health facility. For example, Trichiasis is common in females. Females are more expsosed in my experience, but they only come when the case is complicated and they may not come at all because of economic related issues.

R: Do you think there would be any challenges with regard to stigma? Would those affected by disabilities caused by NTDs access care at the hospital?

B37: *I do not face cases related with NTDs with disability. I saw them in the community but they are not coming. Even if they come for other purposes, they came with their faces covered and they feel ashamed.*

R: What could be done to address those challenges?

B37: To solve the gender and stigma issues related with NTDs, it is a matter of time, but involving the community will facilitate change. The Government also should work on those issues of stigma and gender problems.

R: What would your recommendations with regard to integrating the detection, management, recording and reporting of NTDs into primary healthcare?

B37: In the primary healthcare they are already interlinked, so only capacitating the health professionals to integrate NTDs and awarness should be created before integration.

Health worker, hospital

R: In May this year, classroom training was offered to health workers from your hospital about neglected tropical diseases. Did you attend this training?
B44: Regarding malaria?
R: No, on NTDs.
B44: Yes, I recived the training.
R: Can you tell me what you learned in this training?
B44: I didn't have any knowledge about those diseases at all before the training. Therefore, I did not know that those diseases are treatable. But afterwards, I started telling people with signs and symptoms I met on the street to come for treatment and even people who are begging and who have those diseases. Take the disease we call elephantiasis, for example. Even though it cannot be cured, we can still give supportive care to prevent further progression of the disease, like hydration, wound care, and elevating limbs. Before the training I thought this didn't concern me, but now I understand that it does. I talked with my colleagues about it and we were all surprised by what we learned during the training. In my family, there was a case of elephantiasis and when the training made me understand that the disease is treatable, we started treating him for 15 days.
R: Okay, nice. Do you remember which diseases this training covered?
B44: My friends participated in the training.
R: No, I'm asking about the topics covered by the training.
B44: Okay, there is schistosomiasis, elepanthiasis [...]
R: Okay, don't worry. You will remember later. You told me the about the things you liked about the training, but was there anything you didn't like?
B44: No, there was nothing I did not like about the training No, really nothing
R: Did you think the training was relevant for your job? Why or why not?
B44: Yes, very much so. I already told you -- I am telling you honestly how I feel in my heart. I personally benefitted from the training. I started to treat a family member from the second day of the training.
R: What is your understanding of your role with regard to detecting and managing NTDs?
B44 :I learned this during the training. For example, for schistosomiasis I think they have fever and we may request a urine test. We were also given materials for reference. Maybe I need to refer to those again. I forgot what they say.
R: Did you receive those job aid materials during the training?
B44: Yes, I received them. They are at my home.
R: Do you expect that those materials will help you remember what was taught and help you detect and manage NTDs?
B44: Yes, they are very useful. I haven't used them, but I gained knowledge from the training. You know, sometimes we think we know everything, even from school. But I have seen a lot of important things in those job aids. I haven't used them very well, but they are very important.
R: Do you have any additional points about improving the job aids?
B44: No, nothing. I am comfortable with the job aids.

R: Do you know how health workers at hospital level usually detect soil-transmitted helminth infections?

B44: Ehh, [...]

R: Is it possible to detect those infections?

B44: Yes it can be detected. If the signs and symptoms are known, it can be detected.

R: If a patient has signs and symptoms of soil-transmitted helminth infection, do you know how health workers at hospital level usually reach a diagnosis? [Respondent remained silent for a long time] For example, some of the signs and symptoms are bloody diarrhoea, worms coming out of the mouth and so on.

B44: Ehh, [...]

R: So is it possible?

B44: We send them for a stool laboratory test.

R: Okay, a stool test.

B44: Yes, it can be diagnosed in the laboratory.

R: Would you perform any particular clinical examination or request a particular laboratory test?

B44: I working in triage, so we send this kind of cases to OPD **[outpatient department]***. We sometimes request laboratory tests, but not this one.*

R: If a patient is diagnosed with soil-transmitted helminth infection, do you know how those cases are usually managed at hospital level?

B44: We have drugs like albendazole. Yes, it is possible.

R: Under what circumstances would you refer the patient? Where would you refer the patient to?

B44: Yes, that is sometimes needed if they need **[unintelligable]** *and sometimes they also need to be followed up throughout their life. We usually consider it a minor problem, but it can be severe. Sometimes ascaris can even cause pneumonia in children. The worms may come out of the nose while they are sleeping and they will be in pain when they vomit worms. So it is serious, but we take on the case and we usually consider it a minor problem.*

R: So you are saying they need both referal and follow up?

B44: Yes, they need both.

R: The intervention suggests that health workers at hospital level should detect soil-transmitted helminth infections based on whether the area where the patient resides is known to be endemic for the disease and the following symptoms: worms coming out of mouth/nose/anus, non-malaria fever and vomit, diarrhoea, anaemia, malnutrition, abdominal pain, jaundice, ascites, intestinal blockage, rectal prolapse, finger or nail clubbing. Do you think health workers at hospital level can perform these responsibilities?

B44: Yes, it can be treated.

R: In your opinion, what are the implications of performing this task in terms of time and workload?

B44: I think we have the capacity to treat, but the health worker needs to be interested and able to identify the complications. As you said earlier, some of the complications need surgical interventions. But even though there are challenges, this should be performed. In my opinion, it is a must. It is not fair to refer cases because of workload and time constraints.

R: But will managing those cases increase workload?

B44: No, it does not cause additional workload because it is a must to treat. We also need to educate the patients, because this form of vomiting can be traumatic for the patient. I faced such a case when I was working in OPD. An old person came with another medical problem and was vomiting worms. His family was disturbed and left him on his own, so this needs attention and awareness among the community. Especially for patients from rural areas, we have to create awareness among family members well.

R: The intervention suggests that soil-transmitted helminth infections should be confirmed through a a stool concentration test. Have you ever requested it?

B44: Ehh, I forget, but this is a special test.

R: Is this a test you think can be performed at the hospital?

B44: I am not sure of that.

R: Okay.

B44: Maybe they perform it, but I dont know if they perform it or not.

R: In your opinion, are there any problems with performing stool concentration tests at hospital level as suggested by the intervention?

B44: At the hospital major tests like renal function tests are performed, so this can also be performed, I think. Maybe they don't perform it if it is time consuming.

R: Yes, it is time consuming.

B44: In any case, the patient should get appropriate treatment, so in my opinion, it should be performed. These kinds of cases are not that common -- maybe less than 10, so it will not be a problem. When we treat with the usual test, they will return within a week, which also adds workload, so I think this test is very important.

R: Patients diagnosed with soil-transmitted helminth infection should receive albendazole if the stool concentration test is positive for ascariasis & hookworm infection. Other drugs are recommended if the stool concentration test is positive for other worm infections. Albendazole should be given presumptively if it is not possible to perform a stool concentration test. Do you think health workers at hospital level can perform these responsibilities?

B44: Yes, they can.

R: Do you know how health workers at hospital level should detect podoconiosis?

B44: Ye,s of course, we can detect it perfectly.

R: It can be detected?

B44: Yes, there may be a knowledge gap. Like for me, it will be a problem and even some of the doctors can miss it. Sometimes we think we know so much, but giving attention is another issue and we may ignore them.

R: What signs and symptoms would you look out for?

B44: I know the signs and symptoms well, but usually it is not given attention by all professionals.

R: If you suspect that a patient has signs and symptoms of podoconiosis, what would you do to reach a diagnosis?

B44: I can differentiate by signs and symptoms, but I am in doubt whether it can be treated or not.

R: If a patient is diagnosed with podoconiosis, do you know how those cases should be managed at hospital level?

B44: I am not sure how painful it is, but they will have reduced circulation, so they may feel numbness. If the swelling increases, the skin will crack, so they need wide shoes, avoid heat between the legs and they need to elivate the leg while they sleep.

R: You said you faced one such case in your family?

B44: Yes, by coincidence he came to me when I was doing the training, so I thought about how I could help him. I tried to clean his leg wearing gloves, by picking the dirt between his legs. Then I dried it and bound it with gauze, applied vaseline and so on. I tried to apply everything I had learned during the training. I treated him for about 15 days and I saw a change with my own eyes. The oedema did not increase. During the training, they said the leg can be soaked in cholorine. I was very happy with the training.

R: Under what circumstances would you refer the patient? Where would you refer a patient?

B44: If the swelling is large and if there is wound care is needed, because wound care is not given in our set-up.

R: Is it not given at all?

B44: No, it is not given here, because there is no space here.

R: Okay.

B44: Even people with burns are referred to the health centre for wound care after giving first aid and all the important treatments. Sometimes patients are referred to the hospital for further managment.

R: Would you recommend any kind of follow-up?

B44: Absolutely.

R: Why?

B44: Because they have a disablity.

R: The intervention we are testing suggests that health workers at hospital level should detect podoconiosis based on whether the area where the patient resides is known to be endemic for the disease the following symptoms: non-malaria fever and chills and redness of the leg, swelling of a lower limb, pain in a lower limb, itching/burning sensation of the skin on leg, enlargement of the foot, plantar oedema, skin nodules, hyperkeratosis, moss-like papillomata, joint fixation. Do you feel confident that you have the knowledge and skills to perform these responsibilities?

B44: Yes, it can be managed here , but we need to be shown how the treatment is done.

R: In your opinion, what are the implications of performing this task in terms of time and workload?

B44: Yes, there is a lack of space and workforce, but still, we are giving treatment because it cannot be ignored.

R: So how can we improve the services?

B44: We all have to share the responsiblity. **[Respondent's phone rings, interrupting the interview]** *So we have to share responsibility and create a conducive environment. For example, we are saying wound care is needed, but on the other hand, no room is available where the service can be given, so this will cause trauma for the patient. Already patients think they are being ignored and neglected and we add to this by referring unnecessarily. So all of us, maybe including myself, have to talk and search for a solution. Patients should not be sent back. There will be no more than 5 cases. Maybe after the service starts, the patient flow may increase, but for now, it is not difficult to handle this small number of cases.*

R: The intervention suggests that patients diagnosed with acute podoconiosis should receive pain relief with paracetamol, and be advised to hydrate and rest. All patients should be referred to a health post for advise on home management of lymphedoema. Do you feel confident that you have the knowledge and skills to perform these responsibilities?

B44: What did you say?

R: If there are complications like lymphoedema or if patients need surgical interventions.

B44: Yes, it can be done.

R: In your opinion, what are the implications of performing this task in terms of time and workload?

B44: It depends on the case, but the patient should get treatment. It is not fair to send patients back whatever the problem

R: What do you generally think of the idea of tasking health workers at primary health care level (including health posts and health centres) with detecting, managing, recording and reporting neglected tropical diseases?

B44: Yes, if they are tasked with these diseases, it will not be difficult for them. The training provider and the staff who took the training should co-ordinate to form a team and to create a training chain. That may be important. Health centre staff and staff in this hospital have already taken this training, but we have not yet started acting on it. It should not be like this. You are here now to remind us. I'd prefer for all staff to discuss a solution together, rather than conducting separate interviews. We took the training and you also facilitated it, but the main objective is to start giving those services, not just taking the training. So this should be started and then the patients may benefit.

R: **What kind of challenges do you foresee?**

B44: The first thing is a change of attitude.If health workers do that, there will be a solution. But there may also be a lack of man power and materials, so this should also be given attention and priority. I don't think there will be an issue with space, because they already have a room for emergency and doing wound care there. At the health centre, there are fewer patients. Most people do not go to the health centre, so changing this mindset is also important.They have to take responsiblity and ownership of the problems. If materials and sufficient manpower is there, it may not be a problem.

R: Do you think there would be any challenges with regard to gender? Would women and men face different challenges in accessing care for NTDs at the hospital?

B44: Yes, we have this kind of challenge. For example, if a woman is not willing to be seen by a male professional, but the health worker does not leave the room and respect the patient's rights. So professionals also need to change their attitudes. So if we establish separate rooms for these kinds of cases and health staff's behaviour improve, we can allocate both male and female health workers to overcome the problem. It is very difficult to adress this, because sometimes a whole day, week or month can be covered by just one professional, usually male, so it may be very difficult. At the health centre, most of them are female, so female patients may not have a problem there. Not only for NTDs. STI cases may not be comfortable to be seen by health workers of the other sex.

[Respondent is repeatedly called back to her work station]

R: You said previously that there is stigma in the community. The community think of those diseases as hereditary and so on. You also mentioned gender issues. So what could be done to address those challenges?

B44: Using Health Extension Workers to raise awarness and encourage patients to come to health facilities. So community mobilisation and then here **[at the hospital]** *the environment should be conducive for them. All have responsiblity -- the organisation that gave the training and the professionals who took the training. You came and asked about NTDs, but there is still no solution. For example, I had training on gender violence and during the training we were all talking and very irritated, but afterwards, I did not see a change. So change needs to happen for the people who are suffering from this problem. So coordination with Health Extension Workers and staff at the health centre is important.*

R: Is there anything else you would like to share with the research team?

B44: As I said before, a discussion forum is needed. I don't think your interview achieves this. Do not forget the implementation. Strengthen the programme. If we cure a single case he or she will be a witness for the community. Please,I want to say, go with strong commitment.

R: Many thanks for taking the time to participate.

Data management, hospital

R: In May,training was offered to health workers from your hospital about neglected tropical disease. Did you attend this training?

B45: Yes, I attended the training.

R: Can you tell me what you learned in this training?

B45: What I learned in the training -- the main thing I have got from the training is about tropical diseases – awarenes. And the second thing is -- the cases are seen in large numbers, but due to the problem of reccording and reporting, they are not registered accordingly. So it is difficult to know the load of cases in the country and, as I have raised before, what kind of

influence the cases have in the community, in which area the cases happened and in which age groups. I got a lot of information about those diseases.

R: Do you remember which diseases you learned about?

B45:Ehh, yes, I remember [...] For example schistosomiasis, onchocerciasis, those diseaes which are sexuallyl transmited diseases. I also need to mention diseases like **[unintelligible]***,syphilis and other sexually transmited diseases. And a disease which is transmitted by sand flies and also another one caused by a black fly -- like African sleeping sickness. About this one and the others we got knowledge from the training.*

R:It is good. You already mentioned some of the diseases.Can you tell me what you liked and disliked about the training? Do you have any comments?

B45: For the most part, the majority of the training was very important and I don't have any comments on how the training could have been improved. And wherethere is a lack of awareness, this causes problems in our communities. Because recording and reporting are not given focus and learning about this is something I liked about the training. So the diseases are found in the community but because of poor handling of recording and reporting, we do not know the burden of those diseases. That was the interesting part for me.

R: Was there no part of the training you disliked?

B45: No, I didn't dislike anything.

R: Was it important and relevant for your job to get this training?

B45: Yes, it was. As I mentioned before, mainly with regard to developing a work plan. This is something I do in my work, and the main thing when you plan is to know the disease burden. It is so important to know to what extent diseases affect people. So that was one important part for me. It's very important for me to know this. And I also learned that it's very important at country level, regional and organisational, to make decisions based on collected data. This is very helpful for making decisions in my opinion.

R: You say recording data is important. What is your role with regard to this?

B45: My role is to register and report cases from the lower parts and sending them to the relevant authorities.

R: It is very good. The training was about six NTDs: Trachoma, Urinary schistosomiasis, Intestinal schistosomiasis, Soil-transmited helminth infections, Lymphatic filariasis and podoconiosis.Do you ever record and report any of those diseases?

B45: As I said before, at country level, there is a system which is called HMIS. In the health system double reporting and recording are not possible. Diseases at the national level are already registered. So the main thing is that the report produced from the lower levels -- as I have said before -- and there a great problem is that the lower levels of the reporting system are not getting solutions until now. Of course, at the national level, the diseases are registered and tally sheets have been developed, but the system is not well supported with computers. This is known even at the regional level therefore we have difficulities in knowing the burden of diseases. We even told the higher levels in the country, the Ministry of Health and for about four months now, we have not reported diseases and so don't know their burden. And this is our great problem with regard to reporting and reccording.

R: You already mentioned that these activities are not carried out in your department. The other thing is that urinary schistosomiasis is confirmed by labratory investigations like urine sedimentation. The intervention suggests that this result shoud be recorded and reported.

B45: As I said before, my responsibilities are to report those things that are registered and recorded from the lower level to the relevant authorities. Recording is based on guidelines at the lower level. For NTDs there is one assigned coordinator for the clinical department, which is led by a medical doctor. They could tell you if those diseases are registered and reported from the lower level. Once that's concluded, we process the data, interpret the report and send it to decision makers. Here we are only processing and interpreting the reports and we are reporting. But as you said before, in the laboratory and OPD – according to the guidelines – you better ask the clinical wing coordinator.

R: What do you think about this?

B45: Actually, there is a direction from the Ministry of health with around 60 pages. If you want to look at that, you must look page by page. But the Federal Ministry of Health conducted a study and now it is is computerised, When you need to find the name of a disease, you need to click on the first letter and it shows you the name of the diseases and

you just select. For your information, we are connected in a networking. It's been completely installed and it has been discussed with the Regional Health Bureau. They provide the database according to the national disease classifications. Hopefully we'll start using it this week. So the diseases we mentioned are reported to the higher level starting from next week.

R: What does this mean in terms of time and workload?

B45: Whatever it is -- workload or not -- we belive that everything should be registered and this is already a service that is routinely provided. Therefore, I am not expecting this to create workload in my opinion. The national disease classification is available. There is a space for tallying. But if someone wants to find one disease from among 1,648, they will need to look it up. Therefore finding the code creates workload. It may take one and a half hours to find one disease. But if it is computerised, when we write just one letter, we find it easily. So when this problem is solved, I think it would be good if the Regional Health Bureau also took this as a pilot. But otherwise, regarding the skill gap. Training may be needed not only for those neglected diseases. We are planning to conduct a half-day training. When those problems are solved, all problems will be solved.

R:Do you think the data recorded and reported will be reliable?

B45: Frankly speaking,in our report we under-report what we have done, but we do not report more than what we have done. Sometimes, when they are busy, they may miss he reporting. In our last budget year, we saw 239,000 visits. When we assessed the reported data against the actual register, they were almost the same. Therefore the reports we generate are reliable.

R: How could the problems you mentioned be addresed? What alternatives or improvements would you suggest, especialy with regard to time managment?

B45: Here, as you have seen, there are nodes [?] that have already been installed. These nodes [?] are interlinked through this computer with the lower-level service provider and consequently, everything they record at the lower level, I can show here by name and case on my computer at the hospital level. The development of this system is one option of solving this problem and reducing time and workload. We have already started this for other services and we have approval from the Regional Health Bureau. For example, in the card

room this service has already started. Also, laboratory requests and exchanging results now works through this system and even sending images from the X-ray department. So we have started this kind of work and we've had this national guideline from the month of May, so we must enter this updated one – not only for NTDs, but all diseases are entered. As I said, there are about 2,848 diseases. And as we said, six NTDs are already listed. This is our first plan and the second one is – we plan to train not only on NTDs, but also other recording systems and how we are addressing this task in the future. We are putting more emphasis on NTDs and what services are provided in the future. Those two things may solve our problems.

R: And more generally, what do you think about the the idea of tasking health workers at primary healthcare level (including health posts,health centers and hospitals) with detecting, managing recording and reporting neglected tropical diseases?

B45: You'd better raise this point directly with the City Health Administration. As far as I know, they are already trained and I don't think there will be a skills gap. If they have to manage those diseases, then they will manage. And if they need to, they refer cases to the hospital where we can provide technical support to the health centre if needed. When we plan our health centre support, we can include this in our plan for the next year. If those two things are supported, then I believe they will be implemented. The main thing in my opinion is, we need more clarity from the City Administration. That's my comment.

R: Do you think there will be any challenges with regard to gender? Would women and men face different challenges in accessing care for NTDs at the hospital?

B45: Ehh, I don't think they are facing these kinds of challenges. According to the law and regulations, females need to have services such as ANC **[antenatal care]***, emergency maternal cases etc. They should receive those services as a matter of priority. But also male patients, if there is an emergency, they are treated as priority cases for the services they need. There are departments where they identify whether a case is an mergency or not, so our servies are provided fairly. For this reason, I don't think they face any problems related to gender.*

R: What do you recommend with regard to integrating the detection, management and recording of NTDs in to primary health care?

B45: In the national classification of diseases, if these six diseases are not included, you need to liaise with the Ministry of Health to explain the need to have them included. Another recommendation is to provide additional training. In my opinion, there needs to be a focal person at the hospital for those cases and that focal person may need daily follow up if possible. A daily or weekly feedback mechanism should be established. Because when this service is online, it is good to follow up. Even if the report **[unintelligable]** *and with regard to the recording system, if there is daily follow-up, even if the focal person has another job, he must follow those activities. For those fighting diseases, it will be good to put more focus on them, but for these* **[NTDs]***, I do not expect that, so this kind of follow-up and support is mandatory in my opinion.*

R: Is there anything else you would like to share with the research team?

B45: As I said, my opinion is that health professionals are included, but this study may be focusing on the hospital.

R: The health centres are also included and we are concerned with integrating this task.

B45: Integration is essential because these diseases are not common. Most cases are not coming to the health facilities, so they must be assessed through home-to-home services. This may be addressed at health post level who will need more support. And they also need to generate a report to the health centre level or another relevant body. And your organisation **[Malaria Consortium]** *also needs to get a report from those health facilities or departments. The other thing is the habit of barefoot walking needs more focus. Our town is, as you know, a hot area. Therefore those who walk without shoes need more focus, specifically in the rural areas. We need to strengthen those linkages.*

R: Thank you for your time.

Laboratory staff, hospital

R: In May, training was offered to health workers from your hospital about neglected tropical diseases. Did you attend this training?

B47: Yes, I attended.

R: Can you tell me what you learned in this training?

B47: The training involved different health professionals. I am a lab professional. The training was about the so-called neglected problems, their signs and symptoms and how to give care. When I My job includes lab analysis generally and we saw those kinds of things.

R: What did you like most about the training?

B47: I liked that it was short, but included many important things. This means it was focused on diseases with are usually given little attention, so it helped us to give them more attention.

R: Was there anything you didn't like about the training?

B47: There was nothing I disliked: It was a nice training.

R: Were you told what, according to this training, laboratory staff's role would be with regard to performing those tests?

B47: There are many tasks, but let me talk about the job aids with regard to my own job. The job aids are very relevant and helpful, when it comes to implementing, there are issues related to time, materials and other things. They are not applicable and we are not using them. But as job aids, they are helpful.

R: So you are saying it is difficult to apply the tests that were covered during the training?

B47: Yes.

R: Were you told what, according to this training, laboratory staff's role would be with regard to performing those tests?

B47: Yes, I understand my role perfectly.

R: What is that?

B47: We see our role first of all about sample collection. We deal with collecting quality samples. We also look at the time it takes to sample and how this affects detecting the parasite load and so on. Regurding the quality, we look at all the tests. There were also regional lab representatives, so we try to see more quality in our profession.

R: The training covered thee laboratory tests: urinary sedimentation, stool concentration and Circulating Cathodic Antigen (CCA) test. Do you ever perform those tests?

B47: Mainly stool tests. We looked at the stool concentration technique and urine concentration technique and we saw those two tests.

R: Any others?

B47: There was also a rapid antigen test, which is recommended for under fives.

R: Is it the CCA test?

B47: Yes.

R: Do you ever perform those tests?

B47: No.

R: Never?

B47: No, I have never done a CCA.

R: And the other tests, including stool concentration?

B47: No, it is not applicable in the routine set-up.

R: Why is that not applicable?

B47: First, the reagents are not expensive when we compare them with other tests, but with regard to time and manpower , they are not applicable, especially at hospital level.

R: What do you mean by workload?

B47: We have a lack of staff to perform those tests. We also don't have the space. And then the patients expect us to do those tests within a certain time, so if we take longer – there will be many things because of the workload. So if you think about these kinds of things, it will be difficult to apply them.

R: But you know how to perform the tests?

B47: They have to be modified, especially the stool concentration. For urine concentration, it will not be difficult.

R: Was it modified after the training?

B47: No, during the training. What we knew before and what we were told during the training is different, so we are not that familiar with those new steps.

R: Have you ever performed the tests, especially urine sedimentation?

B47: Yes, urine concentration is our routine work. We put them into the centrifuge and the test is done as usual.

R: This means you are doing urine concentration routinely?

B47: Yes.

R: Which diseases can be detected with this test?

B47: It is not mainly used for the parasites, but it is generally done for WBC **[white blood cells]***, epithelial cells, casts and bacteria. Sometimes, we will find parasites by coincidence. For example, if you think about schistosomiasis, the sampling time is not enough, so it is difficult to test for the parasite even if we are performing the test.*

R: Which diseases can be detected with this test according to the training?

B47: Mainly schistosomiasis --urinary shistosomiasis.

R: How difficult is it to perform the test?

B47: No, it is not difficult to perform the test. It is just a time issue, as I said before.

R: The intervention we are testing suggests that laboratory staff at hospital level should use the urine sedimentation test to detect urinary schistosomiasis. Do you feel confident that you have the knowledge and skills to perform this test?

B47: Yes

R: What gives you the confidence?

B47: First, the basic knowledge I got from my formal education and also because it is our routine work. Based on that I can say this.

R: Do health workers at this hospital ever request this test?

B47: Normally it is requested for urine microscope.

R: Not for the parasite.

B47: No, it is not requested. But even if they request it, it is just requested generally and we don't know whether they request it about the parasite or not. It is better to say it is not requested at all.

R: Have you performed tests to detect urinary schistosomiasis recently?

B47: Schistosomiasis is not found here.

R: It is not found.

B47: No, it is not found in the urine.

R: In your opinion, are there any problems with performing urinary sedimentaiton tests at hospital level as suggested by the intervention?

B47: Do you mean for implementation?

R: Yes , at hospital level.

B47: We are taking about urine.

R: Yes, only about urine tests.

B47: Even for urine – first, it needs orientation for lab specialists as for other things -- about the test, when to take sample and the timing. For the clinicians, rather than requesting generally by saying "microscope" or "chemical test", it would be good if they indicated the purpose of the request. That will make them more focused. We don't really know what their interest in the test is, so that would help us focus on what they are interested in.

R: Are infastructure and equipment required to perform this test normally available at this hospital?

B47: For urine, no problem.

R: But tests are performed after sedimentation? Or is the urine examined directly without sedmentation?

B47: No, let me tell you the usual procedure. Normally, when the urine coes to the lab, the first thing we do is a chemical test for the 10 parameters. Then the urine is entered into the centrifuge. And then the sedimented urine will be examined.

R: So you are doing all urine tests in the same way.

B47: Yes, in the same way for all tests.

R: So there is no implication of performing this test in terms of time?

B47: No, there are no implications in terms of time.

R: Do you think the test results are reliable?

B47: Yes. In my opinion, they are reliable.

R: You already told me that the stool concentration test is not done here, but do you know how to perform a stool concentration test?

B47: When I was a student, I performed it, but in my job -- no.

R: Which diseases can be detected with this test?

B47: Stool concentration is done to detect stool parasites.

R: Which parasites?

B47: It is for stool parasites. The concentration is needed because it is difficult to detect parasites if the parasitic load is low. For example, if you take ascaris, they can be detected by direct wet mount, because they have a high parasitic load, but if you take schistosomiasis, they only release around 20 eggs, which is difficult to detect with direct tests. It is very difficult to see schistosomiasis with the wet mount test. So stool concentration is important in this case.

R: So stool concentration helps to detect schistosomiasis?

B47: Yes , but not only schistosomiasis. It can also detect other parasites, so the probability of missing it is low.

R: This means it increases reliablity?

B47: Yes, it increases the sensitivity of the test.

R: What kind of materials and equipment do you need to perform the test?

B47: Maybe reagents, but the new approach is different.

R: You need additional reagents according to the training?

B47: Yes, I think based on the training, additional reagents are needed.

R: For example?

B47: Stool spoon, a clean tube [...]

R: You said you performed this test when you were a student. How much time does it take to perform the test?

B47: Yes, I did it when I was a student , but I don't remember the procedures. But still, it was time consuming.

R: How much time?

B47: It will take more than one hour.

R: More than an hour.

B47: That time will not be accepted by the patients. They will complain when they have to wait longer than expected.

R: If it takes more time, they will complain.

B47: Yes.

R: How difficult is it to perform the test if time is not a problem?

B47: It is not difficult, but still it needs training. But it is not difficult.

R: The intervention suggests that laboratory staff at hospital level should use the stool concentration test to detect intestinal schistosomiasis in patients over five and and soil-transmitted helminth infections in all patients. Do you feel confident that you have the knowledge and skills to perform these tests with the guide of job aids?

B47: Yes, it is possible.

R: What gives you the confidence?

B47: It is from my formal education and the practice and the experience from my routine activities.

R: Do health workers at this hospital ever request this test?

B47: No, it is not requested at all.

R: You only did it when you were at school.

B47: Yes, I only did it at that time.

R: You mentioned problems associated with performing the tests, so how could the problems you mentioned be addressed? What alternatives or improvements would you suggest?

B47: Applying the stool concentration test, as I told you before, is very difficult. There are parameters that should be fulfilled on the part of the clinician, so they need to be very selective in what tests they request.

R: Are you saying that they have to specify exactly which tests are needed?

B47: Not only that, but if they suspect ten patients, then from among those they have to select some which are most likely to have schistosomiasis and they have to look at this critically. The patients sent for testing should be the ones that are almost certainly positive for the requested tests. When the tests are repeatedly negative, it becomes tiresome for us.

R: So how could things be imporved?

B47: In our department, we need additional personnel.

R: You mean additional personnel is needed to make those tests available?

B47: Yes, if you come too early in the morning.

R: I know the situation in the hospital.

B47: We don't get breaks, so it is difficult to do stool concentration.

R: So additional manpower is needed.

B47: Yes. And I would prefer and it would be better to have a separate work station. But if I may make a recommendation, I would say rather than stool concentration, the Kato-Katz method should be used. It will be less sensitive – somewhere between wet mount and stool concentration, but it would be easier to implement. We can even identify the parasite load. I don't know why they didn't consider that if they think about doing research.

R: We will report your recommendation.

B47: Because in 2006, we did surveillance with EPHA **[European Public Health Alliance]** *where we used the Kato-Katz method and it is a productive test.*

R: Can you tell me about this test?

B47: It takes the same amount of time as wet mount time. There is a specific kit and ingredients. I think it is a better test though it costs more.

R: The Kato-Katz test is more expensive?

B47: Yes, but some of the contents of the kits are reusable and it would not require additional staff.

R: So you recommend using that test.

B47: Yes, because in our context, stool concentration is not applicable, so you should recommend a different and more applicable test.

R: You told me you have never performed a Circulating Cathodic Antigen (CCA) test. But which diseases can be detected with this test?

B47: We detect schistosomiasis for under 5s.

R: What kind of materials and equipment do you need to perform the test?

B47: Based on our agreement, is is done in under 5 OPD.

R: How difficult is it to perform the test?

B47: It can be performed because we are already doing our work. It is not difficult to perform if you read up about it.

R: How much time does it take to perform the test?

B47: Maybe ten minutes.

R: Generally, what do you think about the idea of tasking health workers at primary health care level (including health posts and health centres) with detecting, managing, ercording and reporting neglected tropical diseases?

B47: I don't have any other opinions. The suggestions I have given for clinicians here **[at hospital level]** *also applies to them. But as far as the laboratory is concerned, they need training on the tests.*

R: What kind of challenges do you foresee?

B47: I don't think there will be any challenges. They will not face any problems other than the ones I mentioned in our case.

R: Are there any problems which are found in your set-up, but not at the lower levels?

B47: I don't think so.

R: Do you think there would be any challenges with regard to gender? Would women and men face different challenges in accessing care for NTDs at the hospital?

B47: Actually, we do not encounter NTD cases directly, but I don't think there will be any problems regarding health staff.

R: What if they have oedema?

B47: I don't have a problem with that. As *health professionals, we must not do that. The problem is, they are not coming to the health facility. I saw cases in the town, but they are not coming here*.

R: Males or females?

B47: Both of them are not coming. I have never seen this kind of patients.

R: Do you think there would be any challenges with regard to stigma? Would those affected by disabilities caused by NTDs access care at the hospital?

B47: I think they are not coming because maybe they fear they won't be accepted -- and lack of knowledge about the disease, because they think the disease is not curable, so they lose hope.

R: What could be done to address those challenges?

B47: in my opinion, this will be a very easy task for Health Extension Workers.

R: What they can do?

B47: They serve in rural areas and can reach out to those cases and refer them. They can also facilitate them so they receive free services.

R: What would your recommendations with regard to integrating the detection and management of NTDs into primary health care?

B47: To consider NTDs as a routine responsiblity and health problem. The main thing is awareness creation. For example, Health Extension Workers have a detection, referral and managment system for TB, so it would be very nice to apply this system for NTDs also. Whenever a case is found in rural areas, it can be detected, even cases with resistance. For TB, they have a referal link to the places with expert equipment, but referring NTD cases to the nearest facility will not be a problem.

R: Thanks for your time. Is there anything else you would like to share with the research team?

B47: I have already given my recommendations. Even if it is late, strengthen the start of the project and then implementation should be in place.

Health worker, health centre

R: In May this year, classroom training was offered to health workers from your health centre about nelected tropical diseases. Did you attend this training?

C02: Yes, I attended the training at the XXX

R: Which diseases were covered?

C02: Okay [...] podoconiosis, filariasis, leishmanisasis and trachoma, as well as the stagse of trachoma. I learned also the disease transmission ways.

R: What was the most interesting thing you learned at the training?

C02: I have got good knowledge on the five disease from the training. Previously I did not know very well about those diseases, for example differentiating between filariasis and podoconosis was not simple before. But after the training, I understood that we can differentiate them via their signs and symptoms.

R: What did you like about the training?

C02: I liked the training facilitators. They used participatory ways of the training approach. They have good facilitation skills to deliver the topics within a short period of time.

R: Was there anything you didn't like about the training?

C02: I have one comment. After the training, supervision and feedback from your side and from the health center was poor. For example, at kebele level, community sensitisation activities need improvement, because so far, the community has no information about the NTDs at the kebele level. So it would be good to assign one focal person and encourage the trained staff to support, motivate and raise awareness among the community. This needs special attention.

R: After the training, what is your understanding of your role with regard to detecting and managing NTDs?

C02: Currently, I am working for the health centre as a focal community supporter. I have no experience in treating and diagnosis diseases at the health center, so my role and responsibility is referring the cases to the health center during outreach activities. As you know, the nurses and the laboratory technicians have got similar training as me and they received some materials from your organisation to treat and diagnosis the diseases, so I think they are responsible for diagnosing and treating those diseases better than me.

R: Were you given any materials during or after the training to help you remember what was taught and to help you detect and manage NTDs?

C02: Yes, but *as I told you before, I have no exposure or experience of all the NTD materials, like job aids, at this health centre. However, I used and read the materials at home -- what I*

had at hand. I have no information whether the other staff use the materials or not in the health centre during diagnosing and treating the cases.

R: What were those materials?

C02: The materials we were given during the training were the IMNCI booklet, charts and leaflets, but I did not use them to detect and diagnose the diseases at facility level. I saw two diseases, podoconosis and lymphatic filariasis during outreach and linked them to the health centre, so my duties and responsibility was just to refer the cases to the health centre rather than treating the cases at the outreach site.

R: In your opinion, are the job aids user friendly?

C02: All the job aids materials are helpful for all trained health workers who are working with the patient, but I have no contact with the patient. The job aids are good to get adequate knowledge and skills to treat the diseases by using the job aid. The job aids are also important to differentiate the diseases by their symptoms, for example lymphatic flariasis, podoconosis and elephantiasis are clearly stated in the job aids and I used them for this purpose during field work. But I hope the trained health workers also use them to differentiate the symptoms and treatment.

R: The training and the materials that were distributed provide suggestions for how health workers at health centre level should detect and manage six diseases: trachoma, urinary schistosomiasis, intestinal schistosomiasis, soil-transmitted helminth infection, lymphatic filariasis and podoconiosis. Do you ever see patients with signs and symptoms of any of the diseases?

C02: Yes, I have seen three diseases: podoconosis, lymphatic filariasis and Soil-transmitted helminths. I have seen a case of podoconosis just once, but STH is common everywhere.

R: Do you know how health workers at health centre level should detect podoconiosis?

C02: Ok, ehh -- I think the trained staff can differentiate the diseases, but other untrained health workers are working at Adult OPD and they have no information and knowledge to diagnose and treat the NTDs.

R: What signs and symptoms would you look out for?

C02: I understood the common signs and symptoms for the above diseases -- like the symptoms of podoconosis start from the leg and go upwards and involve both legs with oedema and, eh -- I forgot the other symptoms because I am not working routinely at the health centre. We were focusing on malaria and other transmitted diseases, so most of the time, I was working with Health Extension Worker at kebele level.

R: If a patient is diagnosed with podoconiosis, do you know how those cases should be managed at health centre level?

C02: No, I have no experience of managing and diagnosing the disease at this health centre, but I advised the patient to clean and wash their legs at their home and to use shoes and keep their hygiene at all times. I did not refer the cases to the health centre because they have no money and fear to go to the health centre.

R: The intervention we are testing suggests that health workers at health centre level should detect podoconiosis based on whether the area where the patient resides is known to be endemic for the disease the following symptoms: non-malaria fever and chills and redness of the leg, swelling of a lower limb, pain in a lower limb, itching/burning sensation of the skin on leg, enlargement of the foot, plantar oedema, skin nodules, hyperkeratosis, moss-like papillomata, joint fixation. Do you feel confident that you have the knowledge and skills to perform these responsibilities?

C02: I have no confidence or understanding to treat the disease at site level, because currently I do not remember to treat and refer the cases very well. Due to my current working area, I did not give more attention after the training. I am working outside the health center, but if I read about all the cases, I could treat them without hesitation. However, I have no such exposure to treat using the job aids on a daily basis. That is why I forgot the signs and symptoms and their management. As you know, those cases are not common here and I have low exposure to look at the cases and refer them to a higher level.

R: In your opinion, what are the implications of performing this task in terms of time and workload?

C02: I think treating the cases is similar to other diseases. There are no implications regarding time and burden on health workers.

R: If you have seen patients with signs and symptoms of this disease recently, did you proceed with them as suggested by the intervention?

C02: Yes, I have information from the community that some of the cases reside here in **[this area]***, but the patients did not come here* **[to the health centre]** *due to their poor socioeconomic status and fear of other people***.**

R: The intervention suggests that patients diagnosed with acute podoconiosis should receive pain relief with paracetamol, and be advised to hydrate and rest. All patients should be referred to a health post for advise on home management of lymphedoema. Do you feel confident that you have the knowledge and skills to perform these responsibilities?

C02: Now I have no way to manage those disease like before, because I now have the responsibility to prevent and treat at the community level through health education. Most of those cases have low socioeconomic status and they do not even have money to buy soap to keep their personal hygiene at home. I linked them directly to the head of the health centre, but I did not go further because I have no experience of treating the cases with drugs. My confidence and commitment were just to advise and give enough information about the disease. Unfortunately, I have no power to support those cases myself.

R: Do you know how health workers at health centre level should detect soil-transmitted helminth infections?

C02: Normally, we used to differentiate by using laboratory tests. They have similar symptoms like abdominal cramp, abdominal distention, discomfort and loss of appetite. The health worker should send the lab request for stool examination or direct microscopy. There may be other options, but I have no information to diagnose by using other tests. The most important thing is the skill of the health worker to treat and send the request to the lab to diagnose the cases. I hope 70% of the cases are treated and diagnosed by using lab tests.

R: Under what circumstances would they be referred?

C02: Some cases go to **[name of referral hospital]** *on their own. I do not know that cases are referred to a higher level for soil-transmitted helminths.*

R: If a patient is diagnosed with soil-transmitted helminth infection, do you know how those cases should be managed at health centre level?

C02: Yes, I know most cases are treated with drugs after lab results. Some are treated based on signs and symptoms. The prevention will be, especially in rural areas, using boiled water and wearing shoes which may prevent the skin from breaking.

R: The intervention suggests that health workers at health centre level should detect soil-transmitted helminth infections based on whether the area where the patient resides is known to be endemic for the disease and the following symptoms: worms coming out of mouth/nose/anus, non-malaria fever and vomit, diarrhoea, anaemia, malnutrition, abdominal pain, jaundice, ascites, intestinal blockage, rectal prolapse, finger or nail clubbing. Do you feel confident that you have the knowledge and skills to perform these responsibilities?

C02 As I told you, I got the information about NTDs after I got the training from Malaria Consortium, but I was only referring the trachoma cases before, so my confidence was about referring and detecting trachoma with Health Extension Workers. I have not performed other activities so far.

R: The intervention suggests that soil-transmitted helminth infections should be confirmed through a a stool concentration test. Is this a test you think can be performed at the health centre?

C02: I have no confidence to diagnose and test the NTDs here in our laboratory. Our lab technical was trained in the same training with us, but I don't think they have the capacity to detect STH. Maybe due to skill gap, shortage of lab equipment and staff commitment.

R: The intervention suggests that patients diagnosed with soil-transmitted helminth infection should receive albendazole if the stool concentration test is positive for ascariasis & hookworm infection. Other drugs are recommended if the stool concentration test is positive for other worm infections. Albendazole should be given presumptively if it is not possible to perform a stool concentration test. Do you feel confident that you have the knowledge and skills to perform these responsibilities?

C02: The common drugs of choice to treat soil-transmitted helminthiasis is praziquantel, niclosomide, albendazole, mebendazole and piprazine citrate. For Ascariasis, praziquantel

was best, but niclosomide has the effect of fainting, so the health worker did not use it. The best drug for me was praziquantel, but most of the time, the prescribed drug here is albendazole.

R: Why do you choose this drug?

C02: I prefer praziquantel because this drug is better than the other. When I used it before, it was safe and potent in my opinion. Albendazole is common here but praziquantel is not always available here in the health centre. That is why I prefer to use the drug. It is safe, but that does not mean that albendazole is not the drug of choice for intestinal helmenthiasis. Commonly the drug was available here in the health centre. We even provided the drug for under 5 children during MDA **[mass drug administration]**.

R: What do you generally think of the idea of tasking health workers at primary health care level (including health posts and hospitals) with detecting, managing, recording and reporting neglected tropical diseases?

C02: I have got information about those diseases after the training, but I have no detailed idea of detecting and treating the diseases. Previously, I had experience treating trachoma and detecting the cases simply from the community based on the signs and symptoms and referring to the health centre. But for those diseases, I have no experience of referring and detecting the cases right now.

R: Do you think there would be any challenges with regard to gender? Would women and men face different challenges in accessing care for NTDs at the health centre?

C02: Males and females are not equally faced with the diseases. The women need permission to get care from her husband, because the females are influenced by males at home whether or not to disclose the disease.

R: Do you think there would be any challenges with regard to stigma? Would those affected by disabilities caused by NTDs access care at the health centre?

C02: Fear and stigma is there. They have no money to go for treatement and come to the health centre on their own. I have seen cases who feared to come h,ere to the health centre with signs and symptoms in the genital area, so the gender issue was big here. Yes. Most

patients prefer female health workers for female diseases and male health workers for male related diseases.

R: What would your recommendations with regard to integrating the detection, management, recording and reporting of NTDs into primary health care?

C02: I think this intervention needs follow-up and supervision from both sides, Malaria Consortium and our health centre to push staff to detect and diagnose those NTDs better than in their usual work. So I recommend strengthening the follow-up.

R: Is there anything else you would like to share with the research team?

C02: The main point is that follow-up and supervision need to be strengthened. Both sides, the health centre, as well as Malaria Consortium, need to follow-up on those activities. As you know, we are not all on the same page. Some health professionals need higher commitment to detect and diagnose the cases without a push. I also suggest that material support should be provided for those whose legs are affected by these diseases and soap to keep their personal hygiene.

Health worker, health centre

The first part of interview is about the training. In May this year, a training was offered to health workers about neglected tropical diseases. Did you attend this training?

C13: No, I didn't.

R: If you didn't attend the training, did they tell you anything about the training or did they share any experiences about the training?

C13: Yes, on my initiative, I asked them about what they were trained on.

R: What did you hear from them, for example?

C13: For example, ummm I heard some points.

R: What are these "some points"?

C13: For example – leshmaniasis -- lymphatic filariasis, ehh, elephantiasis and such like and how to treat these cases. But it was not in detail.

R: Ok. Do you think that training was important?

C13: Yes!

R: How? How do you think it is important?

C13: It is skill! **[increased tone]** *We add on what we already have. For example, if new cases of elephantiasis come, we don't know how to manage, since there aren't that many cases and there is no training on it. But now, if such case come, we would not be new for such cases and we can help people. So, I think the training is good, especially for new cases.*

R: You have raised one disease. We will discuss some of these diseases later. After sharing the training experience from your colleague, what was the training benefit regarding detecting, diagnosing and managing cases?

C13: It is as I told you. The cases aren't so many.

R: What is your role with regard to detecting, diagnosing and managing cases, including recording?

C13: I didn't understand.

R: You are not trained, but you have experience from your colleagues.

C13: Yes.

R: Even though there are few cases, what would be your role in a case if case comes with regard to detecting and managing NTDs?

C13: Yes. You know, I am not trained. I'll ask those who have got training and will manage as far as I understand.

R: Now we'll talk about job aids that are designed to help you detect and manage NTDs and that have been distributed. Have you seen such job aids or do you have it?

C13: What type of job aids?

R: As we have been discussing, there are few NTDs cases and you are not familiar with them. In case if these cases come, these job aids help health workers to detect and manage NTDs. For example you may find it as filed, in tables and so on.

C13: Yea. Hahaha **[she is laughing]** *I've seen something bound.*

R: What do these job aids talk about?

C13: About diseases. For example, it is me who pinned a poster about leshimaniasis on the wall. I was not familiar with it before, but after posting that, I have got some information about that.

R: You are talking about posters that are about how diseases are transmitted and their impact. Besides that, for example like the iCCM chart booklet that help to assess, diagnose and manage sick children, have you seen such job aids that can help you to detect, manage and record NTDs?

C13: No! **[shakes head in disagreement]** *Oh, excuse me! On the material I've posted, there is information on how to manage cases.* **[interviewee is talking about the poster**]

R: Can you tell us how to use it to manage cases?

C13: No. **[laughs]**

R: Currently six diseases are selected from NTDs because of their relative prevalence and distribution among others. These are: trachoma, urinary schistosomiasis, intestinal schistosomiasis, soil-transmitted helminthic infection, lymphatic filariasis and podoconiosis. Which of these diseases are common in this area or have you seen any of these cases?

C13: I've seen trachoma. Eeeh also elephantiasis.

R: Which elephantiasis? Infectious or non-infectious?

C13: Am not sure but saw patient with swollen leg.

R: Was the swelling in one leg or two legs?

C13: One.

R: Any other?

C13: Other than trachoma and elephantiasis?

R: Yea.

C13: No.

R: What about schistosomiasis and soil transmitted helminthes?

C13: No. Since there is no investigation, we can't roll out.

R: Ok. You told us that you have seen trachoma cases. So, let's talk about that. At health centre level, how can trachoma be detected?

C13: Yea, since it causes blindness. For example, last time, a woman with her child came to me and I realised that it was trachoma because a white material was covering her eye. She told me that the father of her child also had a similar condition that her child had too. So, I understood that I could be trachoma.

R: What other signs and symptoms other the one you mentioned?

C13: Ehh, one of her eyes was almost stopping seeing and her child's eyes too. So I sent them to the hospital. I put them on TTC **[tetracyclene]** *and sent them to hospital suspecting trachoma.*

R: What about other health workers? How do they detect trachoma?

C13: **[laughs]** *In that it causes severe pain around the eye and then blindness. If the disease progresses in this way, it can be trachoma.*

R: Ok. How we can confirm that the disease is trachoma at health center level?

C13: We suspect, we don't confirm.

R: What about those who have got training?

C13: I've never seen that. I've been here for one year.

R: How you manage or treat trachoma cases?

C13: At health center level, there is nothing except providing antibiotics. We also advise them to keep their hygiene and to adhere to medication instructions. And to go to the eye clinic if the problem persists.

R: What type of antibiotics are prescribed?

C13: Tetracycline, that is what is available and I know.

R: At what level do you refer trachoma cases to a higher level?

C13: If it is causing blindness. Before it reaches that stage, it is very painful and runny.

R: So you refer if a client has runny eyes? What else? How you decide whether to treat at the health center or to refer?

C13: For example, I put them on antibiotics for about three days and tell them to wash their eye with clean water and to return if the problem persists.

R: Ok. That is if the problem persists. What about signs and symptoms? Is it possible to refer before putting on antibiotics?

C13: If it is causing blindness. If there is a vision problem.

R: Where do you refer trachoma cases?

C13: To the hospital, usually in Hawassa.

R: Do you specify where to go? For example where did you refer the case you referred last time?

C13: I referred to **[name of specialist eye clinic]** *because I personally knew it. I asked her if she can afford to go to the clinic and she reported that she can. So I told her to go there. Depending on their capacity, we tell them where to go, including* **[name of general hospital]** *or* **[name of referral hospital]** *because there might be specialists.*

R: How do you follow up clients? For example how do you know whether that woman received services or not?

C13: We don't follow, except when they come to us. I'm not sure if that woman went or not. **[laughs]**

R: The intervention we are testing suggests that health workers at health centre level should detect trachoma based on the following symptoms: irritated red eye, mucopurulant discharge, eye pain or inverted eye lashes. On further examination of eyelashes, eyeball and conjunctiva, health workers should be able to diagnose trachomatous inflammation or trichiasis. Do you feel confident that you have the knowledge and skills to perform these responsibilities?

C13: I do based on the knowledge and skill I have got during my higher education and a little from experience sharing. I've never attended training that could help to manage such cases in better ways, but am doing as much as my capacity allows me to do.

R: If that is so, what type of training? In what way should it be organised to better detect and manage trachoma?

C13: First, it is important if there is training that helps us to better diagnose and treat trachoma. Second, it is better if you share know how from those who have got training. It will help us to detect and manage trachoma cases and also to refer for further care. In addition, it would be better if reading materials like posters are availed to get some important messages about these diseases. If these things are availed, we'll read them and would have better knowledge and skills and serve the community in better way. I think it would be better.

R: In your opinion, what are the implications of performing this task in terms of time and workload?

C13: Ohh it would be good! It would be good if materials are availed and we could manage cases, rather than referring to the higher level. It would be good both for our skill and the community service.

R: The intervention suggests that patients diagnosed with trachomatous inflammation should receive azithromycin or tetracycline. In addition, patients with trichiasis should receive surgery if the lower lid is affected. Trichiasis patients whose upper lid is affected should be referred to the hospital for surgery. Do you feel confident that you can do this?

C13: I manage them. You know people come at the last critical stage when their eye becomes red, runny and the eyelashes grow inwards. They don't come at early stages, thinking that it will resolve naturally. That is why most cases are not managed here.

R: How you express your confidence on this? To manage or refer?

C13: I manage those that must be managed at health centre level and refer those that should be referred. I give medicines and psychological support and also counsel to wash with clean water.

R: What gives you the confidence?

C13: **[laughs]** *I read like on my phone, guidelines. I also see cases. In addition, I ask those who I believe are knowledgeable when they return from training.*

R: Now, we'll continue talking about soil-transmitted helminthic infection. Do you know how health workers at health centre level should detect soil-transmitted helminthic infections?

C13: First of all, we identify by signs and symptoms: abdominal pain, bellyache, diarrhoea, vomiting and so on. Then we request a lab test and based on the result, we detect it.

R: You started mentioning some of the signs and symptoms of soil-transmitted helminthic infections. May you add other signs and symptoms that you remember?

C13: Severe abdominal pain, cramp -- if it is giardia, there might be sound, diarrhoea more than three or four times a day, vomiting and such like. And when we see such symptoms, we send them directly to the laboratory. And based on the results, we treat cases.

R: What kind of laboratory test is done for such cases?

C13: They do microscopic examination. No other test is available.

R: How they do microscopic examination

C13: They know how to examine and identify species.

R: Do you know how they process it? Maybe have you seen while they were processing or did you hear how they process it?

C13: **[laughs]** *They just take a few samples, add reagent on it and after covering it, they see under the microscope.* **[refers to direct microscope test, not concentration technique]**

R: If a patient is diagnosed with soil-transmitted helminthic infection, do you know how those cases should be managed at health centre level?

C13: Yes!

R: What type of treatment you provide?

C13: For example for what?

R: It can be for example hookworm.

C13: Albendazole for three days, praziquentel, metronidaz -- [**did not finish "metronidazole"**] *and such like.*

R: By the way, which diseases are called soil-transmitted helminthes? Tell me those you remember.

C13: You tell me! **[laughs]**

R: Just what you remember. Those diseases transmitted from soil; can be feco-oral or entering through foot skin.

C13: In most cases, we see bacterial cases. They **[the laboratory staff]** *see and say "full of bacteria" and we treat them with cotri* **[cotrimoxazole]**.

R: Would cases of soil-transmitted helminthic infections be referred?

C13: If it is above our capacity.

R: When would you say it is above our capacity?

C13: We have not faced such cases. Some cases come with excessive diarrhoea, and we put them on fluids and if they don't respond, we refer. But I've not seen such cases being referred.

R: How is follow-up done for these cases?

C13: There is no follow-up system. We don't follow them. Sometimes I ask them when they come back if they had finished the prescribed medicines.

R: You have mentioned some of signs and symptoms of STHs infections. In addition to signs and symptoms you mentioned, the intervention suggests that health workers at health centre level should detect soil-transmitted helminthic infections based on whether the area where the patient resides is known to be endemic for the disease and the following symptoms: worms coming out of mouth/nose/anus, non-malaria fever and vomit, diarrhea, anaemia, malnutrition, abdominal pain, jaundice, ascites, intestinal blockage, rectal prolapse, finger or nail clubbing. Do you feel confident that you have the knowledge and skills to perform these responsibilities?

C13: I treat, yea. I've been treating, but I've never faced cases that developed ascites and jaundice. In the future, I hope I would treat all cases. But how could it be with this limited available supply!

R: How did you develop this confidence?

C13: It is as I told you earlier. It is my duty and I've learned to serve the community, so I am confident. In addition, I am interested in that and committed to serve. There is nothing other than these pushing me to improve my knowledge and skill.

R: What do you think are the implications of performing this task in terms of time and workload?

C13: If all things are organised, like laboratory service to investigate, pharmaceuticals supply -- it would be okay.

R: Are you saying that there is shortage of materials to detect and manage these diseases?

C13: Yes! For example, in the laboratory, it is as I told you before. In the pharmacy, there is shortage of drugs. Of course there are drugs which are common, but there is no brand drug.

R: If possible, may you elaborate what is missing specifically both for laboratory and pharmacy?

C13: Drugs may not exist continously. For example, ORS **[oral rehydration salt]** *even may not be available. You may lack fluids. We only investigate by stool examination, but it may need a CBC test* **[complete blood cell count]**. *If these all materials are availed, we can perform all activities. There are tough health workers.*

R: Do you know the stool concentration test? [interviewee shakes her head to indicate no]. By this method, a large amount of stool is taken and centrifuged to concentrate the ova of parasites. The intervention suggests that soil-transmitted helminthic infections should be confirmed through a stool concentration test. Is this a test you think can be performed at the health centre?

C13: In the current situation! I told you things should be fulfilled. It is done as I told you.

R: Tell us how you manage patients you suspect for STHs. You may use the latest scenario you faced, including laboratory investigation and result.

C13: Last time, I saw a child with these signs and symptoms. I requested lab investigation and stool examination showed ascaris. So, I gave albendazole and added ORS. That is all, because we don't follow.

R: Have you faced a scenario that you suspected STHs but the result was negative?

C13: Yes! Sometimes, people who have signs and symptoms are reported negative; but in that situation, I treat clinically.

R: What do you give clinically?

C13: For example last time, a woman came complaining about abdominal pain without diarrhoea. I put her on Tindazole bid for three days suspecting giardia.

R: What about when test result is positive?

C13: Albendazole, praziquentel and other deworming drugs.

R: How confident you are to prescribe these drugs for STHs?

C13: If I got a positive result! **[laughs]**

R: Finally, as you said, you have not attended the training and those who have got training also did not inform you. The intervention we are evaluating focuses on NTDs which had got little attention previously and focus on the way to improve detection, management and recording of the few cases coming to the facility. For this, training has been given and job aids has also been distributed. So, what challenges do you think are there with this regard? And what are your recommendations?

C13: As I told you, things should be fulfilled. For example, if you attend training and things are not fulfilled, you will do nothing. You will even forget what you were trained on unless you practice it. In addition, people are not coming. They go to other facilities. So, I recommend fulfilling laboratory and pharmacy supplies and also training those who have not attended the training. For example, I would be very happy if I attend training.

R: While designing an intervention to prevent and control NTDs, do you think that there would be any challenges with regard to gender?

C13: I've never faced any. So, I don't know.

R: Is there anything else you would like to share with the research team?

C13: It would be better if you come to teach rather than only asking! **[laughs]**

R: We have finished our questions. We thank you for your time and responses.

Health worker, health centre

The first part of interview is about the training. In May this year, a training was offered to health workers about neglected tropical diseases. Did you attend this training?

C15: Yes.

R: How was the training?

C15: The training was informative. At that time, we covered things that were not covered in the curriculum. I took it as an education opportunity.

R: What was covered?

C15: Things which can be managed with little effort that have stigmatic effects on individuals -- that can be from society and also from health workers. Because health workers had no knowledge about these things as it **[NTD]** *was not included in the curriculum.*

R: What did you like about the training?

C15: What I liked was that the training focused on the poor. The focus of training on healthcare provision for poor people is good, as there are people in our country who can't afford medical expenses. So the training was good because it enabled providers to give services for the poor, as those diseases affect the poor.

R: What about things you disliked?

C15: Eh, things I disliked -- I think there was nothing that I disliked.

R: No problem. We are collecting this to improve for our next steps.

C15: Everything was okay, but I can compare two trainings sessions. The second phase was nice because a patient was available and we were trained practically. There was a Podoconiosis case and we practically took their history and managed the case.

R: Are you saying that you disliked the first round of training because there was no practical demonstration?

C15: Yes. I mean compared with the second one. Both were fine.

R: Which diseases were covered during the training?

C15: Neglected tropical diseases like Podoconiosis, Schistosomiasis, lymphatic filariasis, trachoma and others. By the way, these are common in Ethiopia.

R: Do you think the training was relevant for your job?

C15: Yes, very important. It improves our confidence. It is only when the health worker is confident that patients from the community will trust us. Patients will come believing that there is healthcare. We were advising patients **[unintelligible]** *because of low awareness.*

R: Okay. What is your understanding of your role with regard to detecting and managing NTDs?

C15: I'm an Emergency OPD Coordinator. We treat Podoconiosis in emergency OPD. I also do that. Since I'm a Coordinator, I'm always available to detect cases when they come. In addition, as healthcare work is group work, those who couldn't **[attend the training]**, *can ask others and manage in groups.*

R: Now, we will go to the second section, which is about job aids. You know job aids are materials that help to perform activities. Were you given any materials after the training to help you remember what was taught and to help you detect and manage NTDs?

C15: We have planned to give training on awareness creation. The plan was first to train health workers, and then Health Extension Workers and the Health Development Army at kebele level. But people come and ask about reforms to evaluate health centre. We were running here and there and our plan failed. **[The respondent was referring to government reforms to the way health services are evaluated.]**

R: So what materials were given after the training?

C15: We took a manual.

R: What type of manual?

C15: A manual which helps how to treat cases. It is like a small book.

R: Ok. What else?

C15: Nothing. Oh, pictures and also a book. These are the only ones I received.

R: Are the job aids user friendly?

C15: Yes, I use it.

R: So you have this? [researcher shows a job aid]

C15: Yes.

R: For example, can you show us how to classify and manage a febrile under-five child who has no malaria using this guide?

C15: I don't usually use it. **[shakes head]**

R: Have you ever tried to use it?

C15: No. As I told you earlier, I'm busy. I go here and there and couldn't.

R: So it's because you are busy?

C15: Yes.

R: The next point is on specific diseases detection and management. Here, six diseases are categorized in to two:

Group 1: trachoma, lymphatic filariasis, podoconiosis;

Group 2: urinary schistosomisis, intestinal schistosomiasis, soil-transmitted helminthic infection.

Do you ever see patients with signs and symptoms of any of the diseases?

C15: I've seen podoconiosis.

R: What about from group two?

C15: Mansoni.

R: Do you mean Schistosoma mansoni?

C15: Yes.

R: Now, we will talk about podoconiosis and intestinal schistosomiasis. How frequent are these diseases?

C15: Podoconiosis is not that common. There are only a few. I'm told that there were 50, 29 and 21 in different kebeles. Nowadays, I don't see these cases.

R: Why do you think that happens?

C15: First, we said that it is a disease of the poor. So, there may be no helpers to bring cases to the health facility. Second, since no cases have been treated, they think that there is no treatment.

R: Let's us talk about intestinal schistosomiasis. Do you know how health workers at health centre level should detect intestinal schistosomiasis?

C15: By laboratory test. By stool examination.

R: What are the signs and symptoms?

C15: Abdominal cramp. If there is no other complaint, we request stool examination.

R: What else, other than abdominal cramp?

C15: Blood in stool; vomiting because of abdominal cramp.

R: You said that stool examination should be done to diagnose intestinal schistosomiasis. What type of stool examination should be done?

C15: I'm not lab technician **[laughs]**. *They know it specifically. I'm not sure.*

R: If a patient is diagnosed with intestinal schistosomiasis, do you know how those cases should be managed at health centre level?

C15: Yes.

R: What treatment would you provide?

C15: Praziquantel.

R: Under what circumstances would you refer the patient?

C15: We don't refer them.

R: So what you told us is what is happening at this health centre?

C15: Yes.

R: The intervention we are testing suggests that health workers at health centre level should detect intestinal schistosomiasis based on whether the area where the patient resides is known to be endemic for the disease and the following symptoms: diarrhoea, anaemia, malnutrition, abdominal pain, jaundice, ascites, intestinal blockage. Do you feel confident that you have the knowledge and skills to perform these responsibilities?

C15: Yes. Cases with these symptoms are treated accordingly. For example, anaemia is secondary to other diseases. We think about what could probably have caused the anaemia. For example, hookworm can cause anaemia. Another is malnutrition.

R: Do you have confidence to treat intestinal schistosomiasis?

C15: Yes.

R: What gives you the confidence?

C15: Education, training, and experience sharing. Also peer discussion.

R: When did you last manage a case?

C15: Quite long ago. I'm not sure.

R: Are there any problems with tasking health workers at health centre level with detecting intestinal schistosomiasis as suggested by the intervention?

C15: Materials.

R: What type of materials?

C15: For example, materials required to manage podoconiosis and intestinal schistosomiasis.

R: Specifically what do you think can affect intestinal schistosomiasis detection and management?

C15: In that regard, no problem.

R: It can be from any prospective: provider, supply, community etc.

C15: No problem.

R: The intervention suggests that an intestinal schistosomiasis diagnosis should be confirmed through a CCA test for patients up to five years or a stool concentration test for patients over five. Do you know the stool concentration test?

C15: No.

R: Stool concentration test is test by which larger volume of stool than routine stool examination is taken and centrifuged to concentrate parasites, not to miss small amounts. Non important materials are discarded and sediment is examined for parasites. Are these tests you think can be performed at the health centre?

C15: It is not done in the health centre.

R: Do you think it is possible to perform it?

C15: [...] Yes. I think it is possible.

R: Do you feel you have the knowledge and skills to interpret the test results?

C15: They are not performing it.

R: Why is that, in your opinion?

C15: They didn't practice. They are performing the direct one **[direct microscopy]**, *without concentration.*

R: Any other problems?

C15: No. They also had learned this at school, but I think they perform the direct one because it is easy. It would be better if they could lead us. When we say 'stool exam', they should say 'which test?'. Of course our lab request form also doesn't have the stool concentration test.

R: The intervention suggests that patients diagnosed with intestinal schistosomiasis should receive praziquantel if the CCA or stool concentration tests are positive, including patients up to five years. Patients over five years with any of the symptoms of intestinal schistosomiasis should be given praziquantel presumptively if it is not possible to perform a CCA or stool concentration test. Do you feel confident that you have the knowledge and skills to perform these responsibilities?

C15: Praziquantel has been provided in mass drug administration. During that time, there was a tool to measure height and to estimate the dose. But in its **[measuring tool]** *absence, it would be difficult to know dose. In addition, there should be a lab result to prescribe praziquantel.*

R: For example, what would you do if a person you suspected for intestinal schistosomiasis ends with a negative result?

C15: I will consider anti-helminths but not rash to praziquantel. I will consider albendazole and mebendazole.

R: What about praziquantel?

C15: To tell you the truth, we fear it.

R: Why?

C15: We don't have the measuring tool to estimate the dose, because people say it makes people faint.

R: Are there any problems with tasking health workers at health centre level with managing intestinal schistosomiasis as suggested by the intervention?

C15: Though confidence varies from person to person, it is possible to treat schisto cases. There is no problem.

R: Now, we will talk about podoconiosis. How do health workers at health centre level detect podoconiosis?

C15: When cases come, first we see signs and symptoms and then we can consider differential diagnoses. Swelling is considered to do be a differential diagnosis.

R: What signs and symptoms would you look out for?

C15: Podo? Eh, for example, swelling starts from the foot and is bilateral, affecting both legs. Lymphatic filariasis swelling starts here **[points at his thigh]** *and goes down. So, where the swelling starts helps us to differentiate.*

R: Any other methods to diagnose? Other than clinical signs and symptoms?

C15: We do tests for others and if others exist, we come to it.

R: What about treatment?

C15: I think we can give.

R: Do you give?

C15: Cases are not coming.

R: Why?

C15: Because they don't know that there is healthcare for it. It may be due to low awareness.

R: Like as we have talked about intestinal schistosomiasis, we will talk about what intervention says about podo. The intervention we are testing suggests that health workers at health centre level should detect podoconiosis based on whether the area where the patient resides is known to be endemic for the disease the following symptoms: non-malaria fever and chills and redness of the leg, swelling of a lower limb, pain in a lower limb, itching/burning sensation of the skin on leg, enlargement of the foot, plantar oedema, skin nodules, hyperkeratosis, moss-like papillomata, joint fixation. Do you feel confident that you have the knowledge and skills to perform these responsibilities?

C15: Yes.

R: What can affect podo detection?

C15: Shortage of some materials.

R: Like what?

C15: Patient supply. The cases of podo are poor. They may not come to us every time. They need supplies.

R: What about at facility level?

C15: No problem here. We have materials to clean. It is not that difficult. But I think it should be made available to patients at their home.

R: The intervention suggests that patients diagnosed with acute podoconiosis should receive pain relief with paracetamol, and be advised to hydrate and rest. All patients should be referred to a health post for advise on home management of lymphedoema. Do you feel confident that you have the knowledge and skills to perform these responsibilities?

C15: Knowledge to refer to health post?

R: First to manage here and as you said earlier, then to provide care nearer their home at health post level.

C15: Since we started only recently, we have not managed cases in that way.

R: What if a case comes now? Including others' skills?

C15: If you say that, we'll do it in the future.

R: Didn't you hear that during training?

C15: I might have missed it. At home level, caregivers can help the patient by elevating legs, tying with bandages and such like. I don't know if they should be referred to the health post.

R: What if you did refer to the health post?

C15: As I said, if materials are available, they can manage it.

R: Now we'll go to last section. Not the last question. We have been talking about strengthening primary healthcare to integrate NTD care with routine services by giving more attention. What do you generally think of the idea of tasking health workers at

primary health care level (including health posts and the Health Development Army) with detecting, managing, recording and reporting neglected tropical diseases?

C15: There is no recording specific to NTDs. And it's also not integrated into HMIS. Except the recording, there is no problem.

R: What about detection and management?

C15: I can do that! I told you earlier. Healthcare is group work. If we work in groups, I think we can perform.

R: What kind of challenges do you foresee? To detect, manage and record. Not only about podo and schisto, but also other NTDs like trachoma and six diseases we selected for intervention, for example.

C15: I don't see any challenges. If all providers are informed, there will be no challenge.

R: What are the implications of performing this task in terms of time and workload?

C15: No problem. I work 24 hours. I see no problem.

R: What about gender issues? Do you think patients may have preferences regarding the sex of the provider?

C15: No problem. There are such problems in the delivery area. Some women like male providers, while others prefer females. No problem with NTDs.

R: What about stigma?

C15: They may not have many friends. In most cases, they are alone. There are also people who insulting them.

R: How these problem can be addressed?

C15: First, cases should be treated. Second, they should be educated. If they get awareness, I think things will change.

R: Is there anything else you would like to share with the research team?

C15: There should be follow up on how and where trained providers are assigned and how they are performing.

R: This is all we want to talk about. Many thanks for taking the time to participate.

Data manager, Health Centre

The first part of the interview is about training. In May this year, a training was offered by Malaria Consortium to health workers about neglected tropical diseases. Did you attend this training?

C16: Yes.

R: What points were covered? May you tell us the contents of training?

C16: It was about NTDs. More specifically, it was about elephantiasis, onchocerciasis, soil transmitted helminthic diseases and trachoma.

R: What else?

C16: Leishmaniasis.

R: What was the most interesting thing you learned at the training?

C16: Some of these are infectious while others are non-infectious. Previously, these diseases had got little attention. So, it was to work on these diseases with higher attention in the future, as they are causing problems in the community. In past, we also didn't give attention to these diseases. At that time **[the training]**, *we agreed to work on them with more attention. So, it was good.*

R: What did you like about the training?

C16: Because of the lack of attention, we were not familiar with these diseases, except soil transmitted helminthic diseases. However, even though we have got training, we are not reporting diseases since last December. We were told that the reporting format will be changed. We collect data and file it within the health centre, but don't report to higher level. In the past, we didn't follow manuals. But now, using the materials provided, they **[health workers]** *are detecting all diseases. Guidelines have been provided to both under five and adult OPD and diseases are detected according to the guidelines. In the past, there was no guideline and people diagnoses traditionally, using the knowledge they gained in school.*

R: What was the thing you didn't like about the training?

C16: No, nothing. The duration was a little short. It was good.

R: Do you think the training was relevant for your job?

C16: Yes!

R: How?

C16: It helps on how to detect diseases. In addition, it helps on how to record and report these diseases. I saw these things, so it is important.

R: What is your role when these diseases are detected, managed, recorded and reported?

C16: As HMIS focal, I verify reports going back to registers and tallies. Since I am coordinating a "quality improvement" initiative, I monitor if all cases detected are properly recorded or not.

R: We have been talking about training. Now, we'll talk few things about recording and reporting. During the training, you covered six diseases: trachoma, urinary schistosomiasis, intestinal schistosomiasis, soil-transmitted helminthic infections, lymphatic filariasis and podoconiosis. Do you ever record and report any of those diseases?

C16: Till now, I've been reporting soil-transmitted helminthic infections. I've never recorded the others.

R: How frequently do you report soil-transmitted helminthic infections?

C16: Soil-transmitted helminthic infection is common here. It is common. In this area, people don't construct latrines well because of underground water and there is a latrine coverage gap. Though I am not sure if it is due to that, soil-transmitted helminthic infections are common. Every month, we report not less than 30 cases.

R: How exactly do you record and report those diseases?

C16: No problem. They **[health workers]** *continuously register and report it to us* **[HMIS]**.

R: Why do you think other NTD diseases are not recorded and reported?

C16: In the past, I think it was due to providers' awareness. The community also seeks health when there is movement on our side. People focus on acute infectious diseases. So, there is a chance that cases in the community are undiagnosed. In addition, some cases don't want to be exposed. These people can be reached when people go to kebele.

R: For example which diseases?

C16: For example elephantiasis. People don't want to be exposed. In addition, they think that there is no treatment. But since the training, manuals are there and they are registering when there is case.

R: The intervention we are testing suggests that trachoma cases should be recorded as "confirmed trachoma" if diagnosed based on a list of signs and symptoms. In your experience, does this currently happen at this health centre?

C16: In the past, they recorded it as a bacterial infection. But now, if cases come, they record like that.

R: Can we say that trachoma cases are not coming?

C16: In the past, there was no equipment and investigation. Last time, equipment was availed and since that time, they started identifying.

R: If so, did they report "confirmed trachoma" to you?

C16: No, they have not reported any.

R: Why do you think it is like that?

C16: There might be a gap. I'm not sure if cases are not coming, but it is not reported.

R: Do you think that it is challenging to classify trachoma as "confirmed" by assessing signs and symptoms?

C16: Though it is possible to classify, I prefer it to be examined by device.

R: The intervention suggests that urinary schistosomiasis cases should be recorded as "confirmed urinary schistosomiasis" if confirmed by a urinary sedimentation test or "suspected urinary schistosomiasis" if the disease is suspected but not confirmed through a laboratory test. In your experience, does this currently happen at this health centre?

C16: No, not done

R: Why do you think is that?

C16: It may be because there is no case. There might be cases, but it was only very recently that the laboratory technicians were oriented. I think it was in late June. It is only after **[name of person who conducted dedicated laboratory training on NTDs]***'s programme that we started giving attention to these diseases. We usually give attention to malaria. Now, we are giving attention to these diseases since malaria is decreasing.*

R: When exactly did the laboratory service start?

C16: It is less than a month. I think in early August.

R: The intervention suggests that intestinal schistosomiasis cases should be recorded as "confirmed intestinal schistosomiasis" if confirmed by stool concentration test (other tests which are not available here) or "suspected helminthiasis" if the disease is suspected but not confirmed through a laboratory test. In your experience, does this currently happen at this health centre?

C16: It is similar. I've never received such a report.

R: What about soil-transmitted helminthic infection?

C16: It is not in that way. They report cases by age category and sex. When there is electric power, stool examination is performed and the species identified by examination is recorded. They don't report as confirmed or suspected case.

R: What about when recording?

C16: Still similar. They register identified species.

R: What if parasites are not seen, that is, the test is negative?

C16: They may consider other differential diagnoses. **[laughs]**

R: Finally, how do they report it?

C16: Previously, it was reported as "helminthiasis", but now -- By the way, we are not reporting diseases to health office.

R: Though you are not sending disease reports to the higher level, you keep reports at health centre level. How do the health workers record it before sending their report to you?

C16: They report it by dividing into two: intestinal parasitosis and helminthiasis.

R: What about suspected?

C16: It is not reported.

R: What about lymphatic filariasis?

C16: It also had no attention in the past. I think there is no case. I've never received the report.

R: What about podoconiosis?

C16: Similar.

R: Okay. The next section is about your overall perception of the intervention. What do you generally think of tasking data management staff at health centre level with recording and reporting neglected tropical diseases?

C16: Currently, we are only reporting service delivery and not reporting diseases. We are waiting for the new report format of diseases to send to the higher level. Disease reports are filed at health centre level. When there is a case, we compile a report. In my opinion, I think this needs further strengthening.

R: In your opinion, what are the implications of performing this task in terms of time and workload? If we perform with emphasis?

C16: It is a matter of attention. I don't think it is challenging.

R: Even though you are not reporting it to the higher level, do you think the data recorded and reported to you are reliable?

C16: There is no problem.

R: How do you evaluate the idea of tasking health workers at primary health care level (including health posts, HDA, and Health centre) with detecting, managing, recording and reporting neglected tropical diseases?

C16: We usually review activities and performance by the "performance review team" **[a team from different departments that review institutional performance on a monthly, quarterly and half-year basis].**

R: Do you think the primary health care system has the capacity to take on these roles and responsibilities?

C16: Yes, they may have it. But some need training. For example, confirming trachoma using manuals and devices.

R: Do you think there would be any challenges with regard to gender?

C16: I don't think so. It would not be a challenge. Maybe at home, they may resist healthcare seeking.

R: What does resist mean?

C16: As I told you before, they give priority for infectious and acute diseases. It is about the attention they give to diseases.

R: Think of urinary schistosomiasis for example, do you think it would be challenge for women?

C16: No, there is no problem.

R: What about stigma?

C16: There is no problem.

R: Is there anything else you would like to share with the research team?

C16: I feel that we should work hard with the higher capacity we now have compared with before. The support and follow-up is also good. So, I hope we will do better in future.

Health worker, health centre

R: In May this year, classroom training was offered to health workers from your health centre about nelected tropical diseases. Did you attend this training?

C19: Yes, the training was focused on NTDs -- on how to work and get the diseases from the community to detect and treat those diseases, as well as the way of referring cases to the higher level. These are some of the topics I got from the training. Now we are implemeting the activities here **[in the health centre]**. *As you know, at the health centre there is hot water and a lot of people with NTDs came to use the hot water for their illness* **[refers to the use of hot water to wash limbs affected by lymphoedema]**. *At the health centre, we discussed as a group to identify the source of the cases and the way to address the issue in detail. Now the health workers are working in an integrated fashion with Health Extension Workers to address the problem in our catchment area.*

R: What did you like from the training on common NTDs in this area?

C19: All the topics and the training facilitators were very good in my opinion. And it is going to help the community. As you know, those diseases were not given attention by people here, but now patient management gets more attention.

R: Do you remember which diseases were covered?

C19: For example, I remember diseases like leg swelling, breast swelling and cracked legs.

R: Could you tell us the name of the diseases?

C19: Ehh, I forgot the name of the disease. But I enjoyed the training very much.

R: What do you suggest to improve the training in the future?

C19: I suggest to continue this study and strengthen the activities by letting all health workers and Health Extension Workers participate together.

R: Do you think the training is helpful for your current job?

C19: The training was helpful to treat the cases and to know more about neglected tropical diseases, but I have no experience of those diseases in the health centre.

R: What is your role after getting the training?

C19: My role is great, but I haven't given more attention to supporting the women in my department with such diseases with sufficient commitment.

R: Do you think all health workers who were trained at the health centre, including yourself, are partcipating in detecting and managing NTDs?

C19: Yes, by integrating with the 1-to-5 community development team in the community, but I still haven't seen or detected cases in the health centre because there is a shortage of manpower in my department to apply my knowledge of NTDs. Therefore I have no experience of managing NTDs.

R: Were you given any materials during or after the training to help you remember what was taught and to help you detect and manage NTDs?

C19: Yes, I got the training materials, like job aids, a manual, pen, pencil, pictures and different reporting forms. We can access the materials easily and detect the diseases easily from the manual. Some of the training materials also even available in my room.

R: Do you think the job aids are user-friendly?

C19: The materials are clear and simple to use for all health workers if the health workers pay attention at all time. The staff need to concentrate and properly read, then act to improve for the future.

R: Are there any things that could be improved?

C19: The materials were not placed adequately into some of the rooms, so the head of the health centre and focal person for NTDs will need to arrange and place those materials into all rooms. That will be important.

R: The training and the materials that were distributed provide suggestions for how health workers at health centre level should detect and manage six diseases: trachoma, urinary schistosomiasis, intestinal schistosomiasis, soil-transmitted helminth infection, lymphatic filariasis and podoconiosis. Do you ever see patients with signs and symptoms of any of those diseases?

C19: I have never seen those diseases at the health centre.

R: Are there any diseases you would be comfortable talking about in more detail?

C19: I am willing to talk about trachoma and soil-transmitted heleminthiasis.

R: Do you know how health workers at health centre level should detect trachoma? And what are the signs and symptoms?

C19: Well, itching, redness and swelling of the eye, and the eye moves in an outward direction **[refers to protruded eye balls].**

R: Do you think those cases can be treated by health centre staff?

C19: Yes, the health center staff can treat those cases if the materials are available in the centre.

R: If a patient is diagnosed with trachoma, do you know how those cases should be managed at health centre level?

C19: Yes, they can be treated by health workers, but I don't think the materials like drugs are here in the health center unless they are referred to the hospital.

R: What drug should be given to the patient?

C19: I would advise them to wash their face and provide TTC **[tetracyclene]** *and Gentamycin. Iif there is a response after giving the antibiotic, I will refer them to the hospital.*

R: The intervention we are testing suggests that health workers at health centre level should detect trachoma based on the following symptoms: irritated red eye, mucopurulant discharge, eye pain or inverted eye lashes. On further examination of eyelashes, eyeball and conjunctiva, health workers should be able to diagnose trachomatous inflammation or trichiasis. Do you feel confident that you have the knowledge and skills to perform these responsibilities?

C19: There is a knowledge gap on my side, as well as in the health center more generally. It would be better to treat such cases at the center in a separate room by trained staff. I will try to read the job aids, but at the moment I have no confidence to treat and diagnose trachoma. Yes, there is a gap on my side.

R: The intervention suggests that patients diagnosed with trachomatous inflammation should receive azithromycin or tetracycline. In addition, patients with trichiasis should receive surgery if the lower lid is affected. Trichiasis patients whose upper lid is affected should be referred to the hospital for surgery. Do you feel confident that you have the knowledge and skills to perform these responsibilities?

C19: I don't think surgery is used for trachoma, but the first thing is treating them with antibiotic. If the patient is unable to see because fat is covering their eye, they should be referred for surgical management. Sometimes trachoma can be managed with antibiotics and cleaning the face with soap – that may be enough. However, I have no experience with prescribing antibiotics at the health centre. I am not familiar with medical related issues in my department, so I will refer those cases to the adult OPD **[Outpatient Department]** *for medical treatment.*

R: Do you think drugs are available at the health centre?

C19: Yes, some drugs like TTC and Gentamycin are available here at the health center.

R: What is the last stage of trachoma?

C19: Trachoma leads to blindness. The challenge used to be that health workers didn't work with maximum effort to detect and manage NTD, so staff need close supervision and follow-up. They also need to work in an integrated fashion with other staff, as well as with Health Extension Workers. That will be important. However, I have not seen these cases because such cases are treated in OPD. They are not coming to my department with NTDs.

R: Do you know how health workers at health centre level should detect soil-transmitted helminth infections?

C19: Yes, it is a disease associated with soil and hygiene problems here in our area.

R: What are the signs and symptoms?

C19: Abdominal swelling, loss of apetite, nausea and so on. And most of them can be detected by stool examination.

R: If a patient is diagnosed with soil-transmitted helminth infection, do you know how those cases should be managed at health centre level?

C19: Stool examination can detect the disease and it can be treated based on the disease type with albendazole and metronidazole, but we have no experience in this regard.

R: Would you refer such cases?

C19: No referral is needed for soil-transmitted helminthiasis. The laboratory staff are also there to investigate and test for the disease.

R: The intervention suggests that health workers at health centre level should detect soil-transmitted helminth infections based on whether the area where the patient resides is known to be endemic for the disease and the following symptoms: worms coming out of mouth/nose/anus, non-malaria fever and vomit, diarrhoea, anaemia, malnutrition, abdominal pain, jaundice, ascites, intestinal blockage, rectal prolapse, finger or nail clubbing. Do you feel confident that you have the knowledge and skills to perform these responsibilities?

C19: No, I don't have the knowledge and skills to manage and treat those cases. There is a gap on our side to treat NTDs. I have not been giving enough attention and commitment to treating and detecting those diseases. I will read and update myself to address my knowledge gap in the future.

R: Do you have any recommendations?

C19: Generally, it is simple and does not need much support from others, so the only thing is commitment. That will solve the problem.

R: The intervention suggests that soil-transmitted helminth infections should be confirmed through a a stool concentration test. Is this a test you think can be performed at the health centre?

C19: The lab staff have not been performing the test, but I know they were trained to do the stool concentration test. The head of the health centre hasn't worked closely with lab staff and I think only two people were trained from among our lab staff. I don't think all the others, who were not trained, have information and awareness to do the stool concentration test at the health centre. I've never asked the lab staff about the new concentration test.

R: The intervention suggests that patients diagnosed with soil-transmitted helminth infection should receive albendazole if the stool concentration test is positive for ascariasis & hookworm infection. Other drugs are recommended if the stool concentration test is positive for other worm infections. Albendazole should be given

presumptively if it is not possible to perform a stool concentration test. Do you feel confident that you have the knowledge and skills to perform these responsibilities?

C19: Those cases are treated in the OPD and we have not performed them yet. However, it is simple to do the test and to manage accordingly. But all women with complaint of STH are treated at OPD level.

R: Do you think there will be any challenges regarding workload? Or materials?

C19: No, all NTDs can be treated at the health centre because the drugs are available and there is enough manpower.

R: What do you generally think of the idea of tasking health workers at primary health care level (including health posts and hospitals) with detecting, managing, recording and reporting neglected tropical diseases? What is your overall feedback?

C19: Generally, the activities have started here in the community, but previously, such activities were not performed. But now, the 1-to-5 networks work in an integrated fashion with the Health Development Army team and they work in the community in an organised way.

R: Are there any challenges?

C19: If the health centre is ready and staff commitment is there, it will be fine. But the trained staff are not the only persons to bring change.

R: What about the workload?

C19: Yes, the activities need commitment and create workload for staff because health workers need to reach the community t conduct follow-up at home. However, most of the, we work at facility level -- with high commitment

R: Do you expect any challenges with regard to gender or stigma?

C19: Yes, regarding stigma – first, people are not open to talk about those diseases to the Health Extension Workers. Most patients do not mention the signs and symptoms of NTDs becasue they are afraid of discrimination.

R: And gender?

C19: Gender issues, yes -- sometimes it is difficult for female patients to talk to male health workers, but the main thing is that the health worker's attitude in handling those cases. It is not a major issue in our set-up .

R: Is there anything else you would like to share with the research team?

C19: So far, here in **[name of the health centre]***, the project activities have been implemented in a very good manner, so we have to strengthen and sustain project implementation in this area because it is important for the community. Another thing, your follow-up is very important because you are motivating staff to work more and remind us to read. We need to change and most cases are not coming to the health centre, so the Health Extension Workers and community support staff should work together and mobilise the community, telling them about the reality is going to bevery important in the future.*

Health worker, health centre

R: In May, training was given on neglected tropical disease. Did you participate in this training?

C22: Yes, I participated.

R: If you participated in the training, what did you learn?

C22: First, I learnt what "neglected tropical disease" means, what kinds of and which diseases are contained, where this disease is found and which symptoms are seen. According to our level, I also learnt how to treat these diseases. In two days of training I learnt all these things.

R: What interested you in this training?

C22: *I had not known about his disease before, because there were no cases. Even though the training was theoretical, it made an image in our minds of neglected tropical diseases, because they showed us videos and photos. So I think the training was nice.*

R: Can you list some of the diseases you remember from the training?

C22: There was malaria and there was also lymphatic filariasis. I don't remember the others because you out me on the spot.

R: You can tell me later if you remember them. What did you like about this training?

C22: When I was a child, I saw people affected by podoconiosis, so I liked this training especially because it covered this disease.

R: What didn't you like about this training?

C22: Theer was nothing I didn't like. Even the trainers were very nice and polite. Their training style was very good, so it was a very nice training and I was very interested in these two days of training.

R: Which things would you want to be improved?

C22: Things that I want them to improve are -- the training was short, and we took it for two days, so I would want it to be more regular. What I mean is -- we took this training, but at the same time, other people who don't have the information about those diseases have to be

called from their facilities to receive this information. And not only health workers, but also the community needs to participate through committees.

R: Was this training useful for your daily work?

C22: Yes, it helps a lot. When NTD cases come to us, we can detect them or consider whether the case is related with an NTD or not.

R: What is your role related to detecting and managing those diseases?

C22: I have a role, but there are no cases here. Even if there is case **[in the community]***, they won't come here. I work in the health centre as a community supporter and I do tasks at kebele* **[village]** *level through household contacts. I work on helping NTD cases by detecting them, but if they need to be dealt with above our level, we need to organise a referral system to higher levels, like the health centres or hospitals.*

R: Now I want to ask you about the materials you received during the training. Did they help you in detecting cases and making your work easier?

C22: Yes, we use the materials even if there are no cases, because they help us to remember the diseases and to get information. We use them as references.

R: Tell me what kind of materials you received, please.

C22: Some of the materials were pictorial and they showed how and when the diseases affect their victims.

R: Were there any other materials?

C22: Yes, there was a manual.

R: Were there any leaflets?

C22: For example, pictorial document was organised in one page and it was with plastic and paper inside **[laminated]**.

R: Do you have all those materials here with you?

C22: Yes, they are all here.

R: So if a child under 5 comes with non-malaria fever, can show us how you you can use those materials?

C22: By the way, I do not work with under-5s.

R: But you work in the adults room.

C22: Yes, but I do not work inside the health centre. I work as health centre Focal Community Supporter, so I work outside the health centre.

R: You work in shifts, don't you?

C22: Yes, we rotate once every three months.

R: So you haven't worked in OPD for three months?

C22: No.

R: Are those materials easy to read?

C22: Yes,they are easy to read because only the main points are written out.

R: What about the content? Is it clear and easy to understand?

C22: Yes it is, because it is written in simple language and it is short and brief.

R: Could these job aids be improved?

C22: The job aids are not packaged together. They are not organised as one job aid. They need to be packaged as one job aid the future, becasue we need to us it as our reference for other staff too.

R: The intervention we are testing suggests that health workers at health centre level should detect podoconiosis based on whether the area where the patient resides is known to be endemic for the disease the following symptoms: non-malaria fever and chills and redness of the leg, swelling of a lower limb, pain in a lower limb, itching/burning sensation of the skin on leg, enlargement of the foot, plantar oedema, skin nodules, hyperkeratosis, moss-like papillomata, joint fixation. Do you feel confident that you have the knowledge and skills to perform these responsibilities?

C22: Yes. **[laughs]** *Even if it is not treated, I have seen podoconiosis.*

R: How often have you seen it?

C22: Two times.

R: From schistosomiasis and STH, choose the one you know best and we will discuss this disease more.

C22: I don't get a chance to see these diseases.

R: What about the rest of the diseases?

C22: [...] By the way, I used to work in an emergency ward, but I don't get a chance to see these diseases.

R: Which of these diseases do you see the most?

C22: I think I am most exposed to soil-transmitted helminths.

R: Okay, we will talk about your knowledge of podoconiosis and soil-transmitted helminths then.

C22: Can we talk about trachoma rather than soil-transmitted helminths?

R: The choice is yours, but they are in the same group with podoconosis, so we have to select one from each group. What is done in this health center to detect and treat the diseases?

C22: To be honest, I don't get this kind of experience.

R: You can tell us what you have seen from your colleagues. Can you tell us the symptoms of soil-transmitted helminths?

C22: Swelling.

R: What kind of swelling?

C22: For example swelling of the leg -- and wounds.

R: I think you are talking about podoconiosis. We are not talking about this now. Let's talk about STH, that is diseases such as hook worm, ascaris and other worms.

C22: Oh, STH – okay.

R: Yes, the ones that can penetrate through the skin. Let's talk about the common symptoms.

C22: Commonly seen symptoms are diarrhoea, aneamia, abdominal swelling – those are are the symptoms.

R: Can you add more?

C22: There can be bleeding.

R: What do you use to diagnose this disease?

C22: First I focus on the history. For example, if it is a case of diarrhoea, I first check the type of diarrheoa and its frequency and then I diagnose it with a laboratory test. According to our standard, we use stool and blood examination.

R: Why do you use blood? To diagnose bloody diarrhoea?

C22: Not only that, but also the parasites affect the blood, so it is used in order to see the heamoglobin levels.

R: When you send it to the laboratory, what kind of tests do they do?

C22: Stool examination. It is listed on the request form.

R: What kind of stool test is being done by the lab technicians when you send it? What do you think the lab technicians do after you send the stool sample?

C22: Ehh, it can be hook worm -- especially worms like ascarsis -- and they detect those.

R: No, I mean the procedure they follow to test the stool.

C22: The procedure [...]

R: I am just asking if you know it.

C22: No, I don't know the exact procedure.

R: I am asking you because some of them use a microscope and the others use a centrifuge.

C22: I think they use slides under the microscope. I've seen them using a centrifuge, but they mostly use the microscope.

R: How can you treat these diseases?

C22: Which diseases?

R: Those you send to the laboratory. How can you treat them?

C22: We order medicines or give treatment based on the laboratory report.

R: What medicine do you give commonly for each disease?

C22: For example ascaris?

R: What medicine do you order for it?

C22: If it is Ascarsis [...]

R: Just tell us what you do normally. There is no exact answer for it.

C22: We use albendazol, mebendazol and others.

R: Do you think that patients may need to be referred to other facilities because the cases are above the health centre's capacity?

C22: From here – depends on the parasite load and also if they are aneamic and need a blood transfusion. They can be referred to another health centre under these conditions, but we have never done that. We treat people if it is within our capacity.

R: Sometimes the symptoms can be that patients have worms coming out of the mouth and nose. There may be vomiting, diarrhoea, aneamia, malnutrition, abdominal pain, jaundice, ascites and these things can cause intestinal blockage. To detect these signs and symptoms do you think that you have the confidence and knowledge?

C22: Yes, I think so.

R: What makes you confident?

C22: Firstly, I think I got it after I gratuated through learning from experience. I mean I got the knowledge from my education and from the work I do here and what I see. And from the trainings I have taken.

R: In terms of diagnosing, laboratory and manging these diseases, what problems can happen in this health centre?

C22: When we send a request to the lab, there should be enough materials. Sometimes they are not fully equipped with materials and sometimes there is a shortage of medicine when we want to order. And in the laboratory, the technician sometimes lacks awareness and if the technician s don't detect the disease, this can also be a problem.

R: Do you think that the stool concentration test is done in this health centre?

C22: The lab techincian showed us how to use it in the training. After that, two professionals took another training and they showed us here at the health centre. So this work should start soon, but it is was not done previously.

R: So when they start using the stool concentration test and the results come to you, do you think you have the knowledge to interpret and work with them?

C22: Yes, from reading the manual we received during the training, which gave me a broad understanding.

R: Are there any problems with the stool concentration test? It can be on the resources or on the professionals' side.

C22: I think there is no problem in terms of resources, but the staff may not have the knowledge or they may be negligent. They have not fully understand and may not want to improve their professional knowledge.

R: Why do you think that may happen?

C22: I don't know. Maybe because there is no one who can supervised them. I mean the supervisor to the case team is the one who can control them. I think they may not have full knowledge becasue of a knowledge gap, but if he controls them well, tells them how to do their work and motivates them, they will do their work well.

R: You said you will give medicine based on the lab report, so what if the result is negative? Can you treat it clinically? What kind of medicines do you prescribe?

C22: First I need to check if they have started **[performing the stool concentration test]**. *If I suspect the disease, I will see the status of the patient and treat them clinically.*

R: Do you believe that you have the confidence to treat the patient?

C22: Yes, I do

R: Which medicine do you prescribe?

C22: We use the medicines that we have -- like albendazol, melbendazol and other medicines that are in our health centre.

R: Let's move to the next disease, podoconiosis. Has this disease ever been detected here?

C22: It has never been detected here.

R: Can you tell us the symptoms of podoconiosis?

C22: As I told you before, its symptoms are swelling of the legs. It can be caused when people go barefoot, especially on red soil and the skin can be torn.

R: How do you differentiate podoconiosis from lymphatic filariasis?

C22: Podoconiosis affects one side.

R: How is podoconiosis managed and what kind of medicine is given for it?

C22: If a podocoliosis case is found in our health centre, we manage it by telling them to be hygienic, which means telling them to wash their legs daily and using salt as a washing agent.

R: Do you give medicine?

C22: Mostly we don't use medicine, but if there is a wound, we dress it.

R: What do you recommend in terms of follow-up for the patients?

C22: Treating them in a fixed place until they recover. There has to be frequent follow-up and they need their own shoes of their size. So that's all.

R: You told us the symptoms of podoconiosis, but I want to add to those. There can be redness and swelling of the leg, pain and itching and also inflammation. Podoconiosis is bilateral and the swelling starts from the bottom to top, while LF goes from top to bottom. As you said, there are nodes and patients' legs become very dry and the skin can crack. Do you think you have the confidence to detect this disease in the community?

C22: Yes. Creating awareness in the community is good because if you just tell the person that they have the disease, they simply won't believe you. So creating awareness among community about what podoconiosis is, telling them how it is transmited and prevented through the health extension package, and after that, telling them that the disease can be cured – that creates awareness and convinces them to come to health centres to be treated.

R: What are the challenge at the health centre?

C22: The problem is not at the health centre, but it starts from the community.

R: How?

C22: I mean when a person comes here, that person is scared about what the community will say about him because of the disease. So there should be a different place where the patient can rest, and also specially trained health workers need to be deployed at this site for the patients.

R: When podoconiosis reaches the acute stage, you can give patients pain relief like paracentamol. Then you'll advise them to go back to the health centre community support. Do they do that?

C22: The patients need follow-up and they may not come for appointments. But the Health Extension Workers know the environment as well -- even the house of the patient, so they will go to their houses if they don't come for the appointments. If this can be included in the health centre community support, it will be good for the patients, because it is close to where they live. It needs commitment.

R: Are there any improvements you would like to see in terms of podoconiosis detection and managment? Are there any stigma issue as you said and how can these be avioded?

C22: Especially in this place, there aren't too many places. But if there is a campaign and it is known how many cases there are and they let the health centre know, it will be good because health workers will do their job if the cases come.

R: Finally, what is your general opinion on detecting and managing NTDs at the health centre?

C22: I don't think most of the health workers are aware of those diseases, so knowledge about NTDs is important. Some health workers were trained by your organisation. The others need to read the manuals, job aids and documents that were provided. They need to take the time to do this. In my opinion, there needs to be a safe place here at the health centre and when cases come, they are treated and every health worker needs to be aware of how to treat these diseases. I mean when a trained health worker treats and provides care, then other staff need to observe and participate, so they have the knowledge when new cases come.

R: What do you think about the increase in the flow of cases and the workload of health workers?

C22: You mean those working inside the health centre?

R: Yes.

C22: Many activities are done here even if there is high workload, it is seen as a routine activity. If it is seen as routine, it does not affect workload. I believe it does not affect workload.

R: To address these things, what should be done in your opinion?

C22: First, support is needed from health centres, the woreda **[district]***, the city and so on. There has to be support and when support is provided, all health workers will pay attention. So this has to be taken on. I mean training is needed, training should be given and when new technologies or administrative processes are invented, health workers need to organise and work together. If this is done, I think we can do good work.*

R: Do you think that gender can be a challenge? For example, if a person has scrotal swelling, the person in the community will have difficult relations with girls. Second, when he comes to the health centre, the gender of the health worker treating him may have an effect. Do you think this may keep them from coming to the health center?

C22: Yes, it keeps them from coming to health centres. First, there is fear. It's because they are afraid to talk about it and they don't want other people, even health workers, to know. But after learning about a patient, we have to teach him that there is a treatment and a cure for this disease and then bring the patient to the health centre. But these people are afraid to come because they are scared of showing their body parts.

R: What are your suggestions? Do you want to share anything with Malaria Consortium?

C22: First, I want to appreciate that you give us attention in your work. But if a case is found, you do not only work on helping the patient to be treated but also on what work needs to be done in health centres. As I said, health workers have to see how patients are treated in health centres rather than taking theoretical lessons. And these NTD cases are neglected and the community doesn't give them attention, so in my opinion, these cases need their own sites at the health centre to do them justice. And we need to create a referral system to the higher level, which should be clearly understood by all staff.

R: Thank you for your time.

Laboratory staff, health centre

R: The first part of interview is about the training. A few weeks ago, training was offered to health workers from your health centre about neglected tropical diseases. Did you attend this training?

C24: Yes, I did.

R: What did you learn in this training?

C24: First of all, the training was very interesting. And what we learned was about neglected tropical diseases, like leishmaniasis, **[schistosoma]** *mansoni, how to prevent eye disease and others.*

R: What else?

C24: The other thing was about what we do in the laboratory. There is a disease called schistosoma. We learned about that -- the procedures we use to detect schistosoma. It was very interesting.

R: You've mentioned leishimaniasis, schistosomiasis and trachoma so far. Anything else?

C24: We learned about many diseases.

R: Can you tell us about all those diseases?

C24: We also learned about TB. We learned ways of TB transmission and so on.

R: What was the most interesting thing you learned at the training?

C24: The approach and the way they presented. The trainers were very nice. We got some good knowledge. I'm very happy with that. I believe I have got good knowledge now. I saw many things that I did not understand previously and we didn't practice in the past. So I learned a lot and I'm happy about that.

R: Was there anything you didn't like about the training?

C24: Nothing.

R: You can tell us if there were any problems.It will help us to organise such trainings in the future.

C24: I'm happy with everything.

R: Do you think the training was relevant for your job?

C24: Yes.

R: Why?

C24: As I told you, I did not have any information about these diseases. Now, I've got good things that will help me to perform better.

R: What are those good things?

C24: For example, in the past, we didn't perform the stool concentration technique. But the skills I got from attending the training enables me to perform the stool concentration method. We also didn't know how to examine urine for schistosomiasis in the past. Now, after the training, I know how to do those tests.

R: After the training, what is your understanding of your role in performing those tests?

C24: I perform those tests. I'd be happy if requests came **[from other departments]** *and we could perform those tests. But we have not received any requests as yet. We are still doing our previous routine techniques.*

R: Do they know that you can do these tests? Did you tell them?

C24: There were health workers who attended the training. I'm waiting to get requests from them.

R: Are you saying that these techniques have not been put into practice?

C24: Yes.

R: The training covered two laboratory tests: urinary sedimentation and stool concentration. But you are not performing these tests. Which diseases can be detected with this test?

C24: Schistosomiasis.

R: Can you tell us the full name?

C24: Heamatobium mansoni [...]

R: Don't worry, just tell us what you know.

C24: We examine for eggs.

R: Egg of what?

C24: Schistosoma mansoni heamatobium.

R: Which one can be found in urine?

C24: Schistosoma heamatobium.

R: What kind of materials and equipment do you need to perform the test?

C24: A clean urine cup to collect the sample, test tubes [...]

R: What else? Can you do the urine concentration technique only using a urine cup and test tube?

C24: We need a microscope, slide and cover slide.

R: How much time does it take to perform the test?

C24: 30 minutes.

R: How difficult is it to perform the test?

C24: It is not difficult.

R: Do you know how to perform it?

C24: Yes. The previous direct test is very easy. We take urine and immediately we centrifuge it. But in this case, we put urine in a dark place. We don't directly centrifuge as with the direct technique. This new one takes some time. Otherwise, it is easy.

R: You told us that you are performing usual routine tests. The intervention we are testing suggests that laboratory staff at health centre level should use the urine sedimentation test to detect urinary schistosomiasis. Do you feel confident that you have the knowledge and skills to perform this test?

C24: Yes.

R: What gives you the confidence?

C24: The training I attended.

R: What else?

C24: Previously, I used to work in a private health facility. That experience also helped me, I think.

R: In your opinion, are there any problems with performing urinary sedimentation tests at health centre level as suggested by the intervention?

C24: No, I don't think there are any challenges. We can do the tests if they are requested.

R: In your opinion, if you start performing urine sedimentation, what would be the implications of performing this test in terms of time and workload?

C24: Personally, I don't think it would collide with other activities. Because we can perform other activities while running this test. So I don't agree that there are time or workload related challenges to performing this test.

R: You mentionjed earlier that it takes more time than the routine test?

C24: Yes, but we can do other activities while we are waiting for this test to be completed. So it doesn't matter.

R: In your experience, are infrastructure and equipment required to perform this test normally available at this health centre?

C24: Yes. Microscope, slides, test tubes -- all are available.

R: Do you think the test results are reliable?

C24: Yes. I believe it is better than the routine one.

R: My next question is about intestinal schistosomiasis. The intervention suggests that laboratory staff at health centre level should use the stool concentration test to detect intestinal schistosomiasis in patients over five and soil-transmitted helminthic infections in all patients. Do you feel confident that you have the knowledge and skills to perform these tests?

C24: Yes.

R: What gives you the confidence?

C24: Again, the training I attended.

R: How do you perform it?

C24: First we take 10ml detergent solution and 4ml water to prepare a solution. Then we take 10ml solution from what we have prepared and add stool into it. Then we mix and centrifuge.

R: In your experience, are infrastructure and equipment required to perform this test normally available at this health centre?

C24: Yes.

R: If so, do you perform it?

C24: No.

R: Why not?

C24: As I told you, health workers are not sending request to do this test.

R: Why do you think is that?

C24: I don't know. It is better if they answer this question. We are doing the previous routine one. We have not received any requests for the concentration technique.

R: Could you perform stool concentration when you receive request asking for "stool examination"?

C24: It is possible, but we do the direct one, because those requests don't indicatethe concentration technique.

R: What arethe problems with performing stool concentration tests at health centre level as suggested by the intervention?

C24: There's no problem. All materials are also available.

R: Have you heard about the Circulating Cathodic Antigen (CCA) test?

C24: No, I haven't.

R: What do you generally think of tasking laboratory staff at health centre level with performing tests to detect neglected tropical diseases?

C24: I think it is possible and we can do it.

R: In general, what do you think about the idea of tasking health workers at primary health care level (including health posts and hospitals) with detecting, managing, recording and reporting neglected tropical diseases?

C24: I don't see any problems. Except the issue of time, there is no problem. It is possible to do all these activities at health centre level.

R: What about others' capacity to do these activities?

C24: They can do it. If those who did not attend the last training get such trainin in the futureg, I believe that they can do it.

R: If that is so, why have they not started implementing this after the training?

C24: We are waiting for requests. There are health workers who attended the training with us. I don't know why they are not sending the requests.

R: Do you think there would be any challenges with regard to gender?

C24: No. There is no such thing.

R: Would women and men face different challenges in accessing care for NTDs at the health centre?

C24: No.

R: Do you think there would be any challenges with regard to stigma?

C24: Yes. There are cases who hide themselves from the community. Some stay in their home. So I think there might be stigma related challenges.

R: What should be done about this?

C24: Of course there is health education. I think health workers should teach the community.

R: As you have mentioned, there are people who teach on this issue in the community, for example the Health Development Army. What do you think is the problem with the current approach?

C24: Yes, they are teaching, but the problem persists. Still people with such problems fear to use healthcare. People go from this health centre, from the health post and the Health Development Army also teach, but we haven't been able to solve the issue. So I think we need to strengthen awareness creation.

R: Is there anything else you would like to share with the research team?

C24: Such training is very important for us. So if we get such updates and trainings, it will be helpful and I am happy with this.

R: Thank you.

Laboratory staff, health centre

R: A few weeks ago, training was offered to health workers from your health centre about nelected tropical diseases. Did you attended this training?

C26: No.

R: Did anyone tell you about this training?

C26: No, nothing. I did not hear anything from my friends, but I found some leaflets and some reading materials in the lab. I haven't looked at them in detail, but I saw from the manual they were about some diseases like hook worm and Ascariasis. I didn't ask them about the training, as some of my friends are not willing to share the information, especially after getting training. Most of them were silent. I don't now why.

R: Do you think the training is important for your work?

C26: Yes. Previously, I attended training on detection of leishmaniasis in **[name of city in the region]** *and I was trained on detecting malaria -- the Plasmodium vivax and falciparum species. And I have got good information and knowledge from that training. If I could get the training on NTDs here, it would be good, but so far, I haven't received any information about it.*

R: Do you know what the training was about? Which tests were covered and which diseases are detected with those tests?

C26: I have no information about the specific dieases from those who were trained.

R: The training covered two laboratory tests: urinary sedimentation and stool concentration. Do you ever perform those tests?

C26: No, I haven't performed stool and urine tests here, but we some on-the-job demonstration from **[name of person overseeing laboratory staff at the Regional Health Bureau]** *the other week. But I haven't performed stool and urine sedimentation tests due to my workload and becasue I do not have time. The patients are not willing to wait for so many hours. My friends have also not performed those tests for similar reasons.*

R: Why are those tests not performed?

C26: The procedure takes a long time. Especially the stool test, which can take one hour. But we have the materials to do the test. For the urine test, I don't know the exact time.

R: Do you know the procedure for the urine sedementaion test?

C26: No, it is not performed here. But I used to know about the stool concentration test.

R: The intervention we are testing suggests that laboratory staff at health centre level should use the urine sedimentation test to detect urinary schistosomiasis. Do you feel confident that you have the knowledge and skills to perform this test?

C26: No. First of all, I didn't get the training. I have seen the schistosomiasis manual here, but those who were trained never shared their experience from the training. They just placed the job aids in the room, but no experience was shared and they did not show me the steps and procedures for the stool and urine sedimentation tests.

R: What about other lab staff?

C26: The other health staff from the OPD **[Outpatient Department]** *haven't requested stool and urine sedimentation tests and also we lab staff have no experience of doing the test becasue we did not have the reagent and we found it difficult to adapt.*

R: Are there any challenges you encounter?

C26: No problem --no problem to do the procedure, but there aren't enough materials and --I don't know the procedure in detail and I think it took too much time. I don't think the materials are available at the health center to do the procedure perfectly. And it takes a long time.

R: Which test is the best for you?

C26: I think sedementation test is better.

R: How can we solve those problems you mentioned above?

C26: I think we need to request and collect the materials from the hospital or purchase the materials we are missing.

R: The intervention suggests that laboratory staff at health centre level should use the stool concentration test to detect intestinal schistosomiasis in patients over five and and soil-transmitted helminth infections in all patients. Do you feel confident that you have the knowledge and skills to perform these tests?

C26: Yes, if the cases are coming here to the health centre and the health workers request those tests, then I have got this experience through my daily work, especially for hookworm and trichuriasis, which are common.

R: Do you have a job aid to differenciate?

C26: We have different pictures to differentciate those species in the laboratory department, which we received from the zonal department.

R: Do health workers at this health centre ever request those tests?

C26: Yes, very often health workers from different departments request a stool examination test **[refers to direct microscopy]***, but I have not seen the stool concentration test here at the health center because there is no request form. There is also a lack of awareness of the stool sedimentation test on the part of health workers. I think there would be no challenge to do this test here.*

R: And the urine test?

C26: The test needs a lot of time and that has implications for our work, because some patients complain if we take a long time.

R: Do you have equipment required to perform this test normally available at this health centre?

C26: Yes, there are enough materials for sthe tool consentration test at the health centre.

R: Do you think the test results are reliable?

C26: Yes, it is a reliable test.

R: The intervention also suggests that laboratory staff at health centre level should use a Circulating Cathodic Antigen (CCA) test to detect intestinal schistosomiasis in patients up to five years. Test kits were provided to the health centre as part of the intervention. Do you feel confident that you have the knowledge and skills to perform this test?

*C26: What do you say? CCCA? I have no information about this and nobody performs this test here at the health centre***.**

R: What do you generally think of tasking laboratory staff at health centre level with performing tests to detect neglected tropical diseases?

C26: Those who attended the training must share the information with untrained staff. That will be important. And those trained health workers and lab staff need to discuss with each other and work together. That will be important in the future.

R: What do you think about the idea of tasking health workers at primary health care level (including health posts and hospitals) with detecting, managing, recording and reporting neglected tropical diseases?

C26: It is good to recorded and report such NTDs cases. I have not faced any challenge in this regard. All worked activities are recorded and reported here at the health centre.

R: Do you expect any issues relating to gender?

C26: The gender issue may or may not affect people. It is not a common issue for us because I have not seen such NTDs here at the health centre.

R: Is there anything else you would like to share with the research team?

C26: I think the training is good for us, so if you provide the NTDs training, we will have the knowledge and will support each other in the lab with regard to the examinations and stocking common lab materials. Therefore, I recommend that staff who were trained should share what they got from the training with those who were not trained. This will create transparency wand it will be important for us. All these things are interesting and I would like to thank you.

R: Thank you.

Health worker, health centre

R: The first part of the interview focuses on the training. In May this year, a training was offered to health workers about neglected tropical diseases. Did you attend this training?

C27: No, I didn't attend.

R: Did anyone tell you about this training?

C27: No, no one told me about what they had learned.

R: Did you ask those who attended training something about the training?

C27: I don't lie. I didn't ask **[shakes head]**. *I don't know what they learned in the training.*

R: The next part is about job aids. Have you received any job aids that can help you detect and manage NTDs?

C27: Yes. They put some materials.

R: What are these materials?

C27: Guidelines on tropical diseases.

R: What else?

C27: Related to this training, that is the only thing I've seen.

R: Where did you see that guideline?

C27: It is available in the under-five room.

R: Have you seen other materials there? May be posters or on the tables?

C27: No **[shakes head]**.

R: Did any one tell you how to use it?

C27: No one told me about it. They just put it there.

R: Did you read it? What does it contain?

C27: No **[shakes head]**. *It is not clear to me.*

R: Did you read it?

C27: I saw justthe highlights this morning. But not in detail.

R: Why it was not clear to you?

C27: It has arrows, but I didn't understand what the arrows refer to. Not to tell a lie, I didn't look at it in detail.

R: Thanks. There are many neglected tropical diseases. The training and the materials that were distributed provide suggestions for how health workers at health centre level should detect and manage six diseases: trachoma, urinary schistosomiasis, intestinal schistosomiasis, soil-transmitted helminthic infection, lymphatic filariasis and podoconiosis. Do you ever see patients with signs and symptoms of any of the diseases?

C27: In the under-five clinic, ascaris are common. Sometimes giardia because children cannot maintain good hygiene and sanitation.

R: What about others?

C27: I'm not familiar with these diseases. I've never seen such cases, except hearing about the science and seeing them on posters.

R: You are familiar with ascaris patients, so let's talk about soil-transmitted helminthic infections later. Now, choose one disease from trachoma, lymphatic filariasis or podoconiosis.

C27: Trachoma.

R: Let's talk about trachoma then. Do you know how health workers at health centre level should detect trachoma?

C27: No. I've never managed trachoma cases, but sometimes I see conjunctivitis in children.

R: What are the signs and symptoms of trachoma?

C27: Plucking of eyelashes, redness of the eye [...] I don't know any others.

R: Just tell us what you know. Don't worry, we are not testing you. There are no right or wrong answers.

C27: I don't know any others.

R: Okay, so if you see these symptoms, you suspect trachoma?

C27: Yes.

R: How do you diagnose it? [respondent remains silent] At health centre level, is it possible for you to say it is trachoma if you see these symptoms?

C27: I can't say.

R: What about other providers?

C27: I don't know about others.

R: If it is trachoma, is there drug for it?

C27: Yes. We can put them on antibiotics like TTC **[tetracyclene]** *for some time and if it doesn't resolve, we refer. In addition, we advise to maintain good personal hygiene.*

R: You said that if problem doesn't resolve after treatment with TTC, you refer. Are there any other circumstances when you would refer the patient? [no response] Where would you refer the patient?

C27: Hospital or private clinic.

R: Trachoma cases or other cases?

C27: Others.

R: What about trachoma?

C27: If I face a trachoma case, I'll refer to the referral hospital. But I've never seen a case.

R: The intervention we are testing suggests that health workers at health centre level should detect trachoma based on the following symptoms: irritated red eye, mucopurulant discharge, eye pain or inverted eye lashes. On further examination of eyelashes, eyeball and conjunctiva, health workers should be able to diagnose trachomatous inflammation or trichiasis. Do you feel confident that you have the knowledge and skills to perform these responsibilities?

C27: I hope my confidence will improve in the future.

R: What if you face a case today? Are you confident to do this?

C27: If I understood it better, I'd be confident.

R: But now, could you diagnose it as I described?

C27: Yes.

R: What gives you the confidence?

C27: I usually do my best. I make an effort to diagnose diseases, but I think I should read more.

R: We would like to know how you feel now.

C27: Previously, I did not use to have the knowledge. But from what you have told me, now I can do it.

R: Why didn't you have the knowledge?

C27: Because there was no case. In addition, because of my poor reading habits.

R: Any other reasons?

C27: That is all.

R: In your opinion, are there any problems with tasking health workers at health centre level with detecting trachoma as suggested by the intervention?

C27: **[shakes head]** *I don't think so.*

R: We have been talking about detection of trachoma. Now let's talk about treatment. Earlier, you mentioned TTC. In addition to that, the intervention suggests that patients diagnosed with trachomatous inflammation should receive azithromycin or tetracycline. In addition, patients with trichiasis should receive surgery if the lower lid is affected. Trichiasis patients whose upper lid is affected should be referred to the hospital for surgery. Do you feel confident that you have the knowledge and skills to perform these responsibilities?

C27: If I read up on it, I can do it.

R: And now? Do you have the knowledge and skills to perform these responsibilities?

C27: Now, I can't do these things, starting from no where. I haven't read the manual and have never faced a case, so I can't comment on it.

R: Now let's discuss soil-transmitted helminthic infections. Do you know how health workers at health centre level should detect soil-transmitted helminth infections?

C27: We assess signs and symptoms and then we request stool examination from the lab. Then depending on lab results, we give treatment.

R: What signs and symptoms would you look out for?

C27: Abdominal cramp, sometimes cough, for example if it is ascaris -- and others like fever.

R: If you suspect that a patient has signs and symptoms of soil-transmitted helminth infections, what would you do to reach a diagnosis?

C27: Stool examination.

R: What type of stool examination?

C27: I don't know.

R: If a patient is diagnosed with soil-transmitted helminth infection, do you know how those cases should be managed at health centre level?

C27: Yes.

R: What treatment would you provide?

C27: As I told you earlier, ascaris is common in under fives. As you know, ascaris can be treated by albendazole or mebendazole.

R: How do you give it?

C27: I don't give it to under two years. Because they can't give stool for diagnosis. And also I don't give it clinically. So, if they are above two and confirmed by lab -- for example ascaris -- I give 4mg of albendazole single dose. If it is syrup, I give 20ml.

R: What if other than ascaris?

C27: So far, I have only treated ascaris and giardia. I haven't faced any others. Sometimes hookworm.

R: What you do if it is hookworm?

C27: It is similar, but if the child is aneamic, we give treatment for aneamia.

R: What else?

C27: That's all I know.

R: Can a person be referred due to these diseases?

C27: I don't know. But I think if it is severe -- like if we suspect that ascaris have migrated to the liver. But I've never faced such things and have never referred them.

R: The intervention suggests that health workers at health centre level should detect soil-transmitted helminth infections based on whether the area where the patient resides is known to be endemic for the disease and the following symptoms: worms coming out of mouth/nose/anus, non-malaria fever and vomit, diarrhoea, anaemia, malnutrition, abdominal pain, jaundice, ascites, intestinal blockage, rectal prolapse, finger or nail clubbing. Do you feel confident that you have the knowledge and skills to perform these responsibilities?

C27: In this case, I am confident. Because we are working on it. So I feel I'm confident.

R: What gives you the confidence? You have already mentioned that you are working on it. What else?

C27: I have read about it.

R: In your opinion, are there any problems with tasking health workers at health centre level with detecting soil-transmitted helminthic infections as suggested by the intervention?

C27: No.

R: As you said earlier, when you suspect these parasites, you request a lab test. The intervention suggests that soil-transmitted helminth infections should be confirmed through a stool concentration test. Do you think that this test can be performed at the health centre? [no response] Do you know the stool concentration test? [participant shakes head and researcher briefly explains the technique] Do you think this test can be performed at the health centre?

C27: I'm not sure. Since I don't work there **[at the lab]***, I don't have any knowledge about that.*

R: The intervention suggests that patients diagnosed with soil-transmitted helminthic infection should receive albendazole if the stool concentration test is positive for ascariasis & hookworm infection. Other drugs are recommended if the stool concentration test is positive for other worm infections. Albendazole should be given

presumptively if it is not possible to perform a stool concentration test. Do you feel confident that you have the knowledge and skills to perform these responsibilities?

C27: If I suspect a case, yes. That is if children are above four and they didn't get deworming within the last six months. I never give for children under two years, because the guideline also puts a contraindication for children under two years.

R: We are not dealing only with ascaris. Different drugs are prescribed for different parasites. Are there any challenges about following the guidelines I described earlier? [no response] It can be anything you can think of. It can be in terms of supply, provider, lab etc.

C27: I don't know.

R: You know there are many NTDs. The six I mentioned are just those relatively common in our area and we are trying to integrate them with other routine services. What do you generally think of the idea of tasking health workers at primary health care level (including health posts and hospitals) with detecting, managing, recording and reporting neglected tropical diseases? [no response] Do you think it is possible? Can the health centre perform this?

C27: If we give attention, we can do everything. I think it is possible.

R: Do you think that primary health care level has the capacity to do this? For example your health centre?

C27: Unless the providers ignore it, I don't think there will be any difficulties.

R: What challenges might you encounter?

C27: I don't see any challenges.

R: Do you think there would be any challenges with regard to gender?

C27: Pardon?

R: Do you think these diseases attack male and female differently, or do you think male and female cases of these diseases access health care differently or do you think cases of these diseases prefer sex of provider etc. Tell us what you think. [shakes head] For

example, do you think a female with genital infection would show it to male provider or vice versa?

C27: No problem. No problem.

R: Do you think there would be any challenges with regard to stigma?

C27: Stigma [...]

R: For example do you think that patients in the community with elephantiasis fear to get healthcare? It can be cultural or something else.

C27: I don't know.

R: Is there anything else you would like to share with the research team?

C27: It would be good if we did everything you raised. But it would better if you considered healthworkers who still have a knowledge gap. So it would be good if you provided training so that we can do all of these things. In addition, your support should be continuous.

R: Thank you.

HEW Worker

R: In May this year, classroom training was offered to Health Extension Workers from your health post about neglected tropical diseases. Did you attend this training?

P01: Yes, I attended the training.

R: Can you tell me what you learned in this training?

P01: The training was about neglected diseases. I learned about the kind of the diseases which were neglected -- how those diseases are transmitted and how they affect society.

R: Do you remember which diseases this training covered?

P01: Yes, I remember. We were trained on trachoma, abdominal diseases which make people vomit and have diarrhoea, soil-tramsitted diseases, diseases which make children urinate blood, or make the flow of urine interrupted when a man urinates, a disease wich affects women's womb, and also about the disease which makes men's scrotum swell, and a disease wich causes swollen legs in men or women. We learned about this kind of diseases.

R: What did you like about the training?

P01: The training was interesting. The most important thing I learned was, for example -- my understanding of elephantiasis transmission before the trainig about was that it can only be transmitted from person to person through hereditary ways, but now, after the training, I understand that the disease can be transmitted by certain flies and that disease transimission can be prevented by using bed nets. And also I understand that the disease is communicable from one person to another not only through hereditary ways. Prevesiously,our focus was on other diseases, like thyphoid, malaria and others, but know we learned about these neglected diseases. This is the additional thing we learned, but of couse, it is the kind of disease we had to cover as part of our previous work activities.

R: Was there anything you didn't like about the training?

P01: Actually, the training was good, but one thing I recommend for the trainig is -- like we were trained, I propose that the training should be delivered to the Health Development Army and other kebele leaders. They should be trained in one place, because I think this would create ab better understanding of the diseaseamong all community members and it would help to reach the whole community in a short period of the time.

R: Did you think the training was relevant for your job?

P01: Yes, the training was relevant to my work.

R: How?

P01: If we could actually implement all we learned -- for example, bed net coverage can be incresased when the commmunity is aware of how the disease which makes the legs swell is transmitted. Also, previously, our understanding – and the community's understanding of the disease which makes people urinate blood was that it is related to the kidney and no other causes, but now I understand that the disease can be transmitted when a person is attacked by a snail when they wash or swim in water. You know, I remember my experience with one child I observed urinate urine the colour of blood. I suspected that the child has kidney disease because at the time I did not know the disease can be associated with other factors. Overall, our awareness and the awareness of the community about these diseases increased after the training.

R:After the training, what is your understanding of your role with regard to detecting and managing NTDs.

P01: After the training, I understood that my main role will be to create awareness about NTDs. For example, when I find a person with a swollen leg, I teach them to wash their legs regularly, to elevate the affected lim. And if the person has suspected trachoma, I teach them personal hygiene and advise them to visit a health centre. If the eye condition is complicated, the patient will receive surgery at a hospital. We also treat children who have abdominal pain and cramps, providing mebendazole and albendazole. We also provide TTC **[tetracyclene]** *ointment as first-level trachoma treatment.*

R: Were you given any materials during or after the training to help you remember what was taught and to help you detect and manage NTDs?

P01: Yes, I received certain materials.

R: What kind of materials did you recieve?

P01: I received guidlines which tell us how diseases are identified by signs and symptoms, how patients are referred and how patients can be treated. And also, they gave us teaching materials with pictures and a reporting form.

R: Did you use those job aids?

P01: Yes I use them.

R: What do you like and dislike about the job aids?

P01: Everything about the job aid is good.

R: What is peculiar thing you liked of the job aid?

P01: As I said, all parts of the job aid are good, but one thing I recommend is -- as you know, currently we have two manuals. One is yours and the other manual is for integrated management of neonatal child illness. So for example, if I want to diagnose malaria, I use the IMNCI protocol to identfy the disease. Also there is a manual from your organisation, so when we get NTD cases, we should look at your manual. Hence I recommend that both manuals be merged into the IMNCI manual, because that will save time in managing cases.

R: The training and the materials that were distributed provide suggestions for how Health Extension Workers should detect and manage six diseases: trachoma, urinary schistosomiasis, intestinal schistosomiasis, soil-transmitted helminth infection, lymphatic filariasis and podoconiosis. Do you ever see patients with signs and symptoms of any of the diseases?

P01: Actually I have observed all those diseases you mentioned. I even diagnosed some of the diseases in my catchment area -- diseases like soil-transmitted helminth infection. I found a patient and treated them using albendazole and mebendazole. And I also treated a suspected trachoma patient with TTC oitment.

R:Do you know how Health Extension Workers should detect trachoma?

P01: *Yes, I do.*

R: What signs and symptoms would you look out for?

P01: The signs and symptoms of trachoma are -- their eye are filled with white secretion, irritated eyes, eyes become red, the upper eye lid is inverted, tears flow from their eyes and they have difficulty seeing light.

R: If you suspect a patient might have trachoma, what would you do?

P01: I would make an effort to help the person affected by trachoma as best I can. For example, I would teach them to wash their face and eyes regularly, using water and soap. I would advise them not share towels among the family. If the status of the eye is not complicated, I would give the patient TTC ointment and if the patient comes back and the condition has not improved by the TTC ointment, I would advise them and refer them to the health centre.

R: Under what circumstances would you refer the patient? Where would you refer the patient to?

P01: When I find a patient with inverted eyelids, I refer them to the health center [...] and I also refer when I get patients with red eyes.

R: Would you recommend any kind of follow-up?

P01: Yes, I reccomend to follow up patients I referred and treated because that is a routine activity in my job. I should follow the status of the patient by visiting their household or if the patient can visit the health post, we follw their status at the health post.

R: The intervention we are testing suggests that Health Extension Workers should detect trachoma based on the following symptoms: irritated red eye, mucopurulant discharge or inverted eye lashes. Do you think it is possible for Health Extension Workers to perform these responsibilities?

P01: Yes, they can perform the activities, but I think additional training may be required.

R: Why do you think additional training should be delivered?

P01: I feel I didn't mention the signs and symptoms of trachoma in response to the questions you asked. I think this is due to a lack of knowledge of this disease. And I couldn't tell you with confidence when I should refer a trachoma patient to the health centre.

R: How would you say the intervention is different from what you normally do?

P01: I think the intervention is not different from what we used to do before. Maybe one way in which it is different is that previously, we did not record and refer patients to the health centre. Now this intervention has helped us give more emphasis to trachoma compared with before.

R: In your opinion, are there any problems with tasking Health Extension Workers with detecting trachoma as suggested by the intervention?

P01: I don't think Health Extension Workers face any problems in detecting trachoma, because it is similar to what we used to do at household level previously. When we visit the household, we can detect and identfy the disease.

R: In your opinion, what are the implications of performing this task in terms of time and workload?

P01: Actuallly, working on trachoma is good because it helps the community to get better diagnosis and detection, but in terms of time and workload, it takes more time and effort for us, because we are assigned to extra work beyond the 16 to 18 health extension packages we have been delivering. Therefore, I recommend that additional Health Extension Workers should be deployed. Alternatively, staff from the health centre should help us to work on NTDs with us.

R: The intervention suggests that patients with signs and symptoms of trachoma should be referred to the health centre for further assessment. Do you think people will follow your advice?

P01: Yes, they will follow our advice.

R: Why?

P01: Because when we advise, we teach them very well. Also, there is no one who like to lose their eye, hence I think they will accept our advice. In my experience, people I refer go to the health center.

R: What could be done to ensure people with signs and symptoms of trachoma receive good care?

P01: In my opinion, as I told you earlier, people we detect and refer dovisit the health centre, but the challenge is that almost all of those affected by trachoma are poor. Therefore, some might not go to the health centre and receive good care. So I recommend that your organisation and the government should support those people and provide diagnosis and treatment free of charge for the poor. You know, if the case is not complicated, we can treat it with TTC ointment at the health post, but when suspected patients are referred to the health centre, they will be asked to payfor all eye disease services, so that may deter patients from going there. In addition to financial support, I think we also need to create awareness among the widercommunity to ensure patients receive good care.

R: Do you know how Health Extension Workers should detect lymphatic filariasis?

P01: Yes, I know how LF should be detected.

R: What signs and symptoms would you look out for?

P01: The signs and smptoms of LF are swelling of both limbs or legs, fever and sometimes patients shiver or feel cold. These are the signs I know.

R: If you suspect a patient might have lymphatic filariasis, what would you do?

P01: When I find a person who I suspect may have the disease, I teach them and advise them to wash their limbs regularly, to wear boots on their feet and elevate their leg. In addition, even though we didn't receive the lotion that's applied to the skin of affected people, the NTD trainers told us that there's a certain type of oil or lotion. If that lotion is available, we should advise thatthe affected person should apply it to the cracked part of their skin. And also I advise them to move, for example by walking.

R: Would you provide any treatment?

P01: No, I don't treat them.

R: Why not?

P01: Our role is teaching and providing advice, detecting and referring when we suspect a case.

R: Under what circumstances would you refer the patient? Where would you refer the patient to?

P01: Immediately as I find a patient, I refer them to the health centre, because my role is to detect and advise them to go to the health centre.

R: Would you recommend any kind of follow-up?

P01: Yes, similar to what we do during our household visit to check implementation of the health extension package at household level, we follow up the patients we referred, because we have to see the progress of the patient. But I haven't started following suspected patients because of lack of time. We haven't even finished orienting the Health Development Army about NTDs.

R: Do you know how to advise patients on the home management of the disabilities casued by lymphatic filariasis?

P01: Actually, I haven't received training on how to advise patients at home, but I advise them to wash their legs regularly if they can. And if they can't, I advise their family to wash their leg. I also advise them to elevate their leg when they sleep and rest at home. And I teach the community not to marginalise this kind of patient and I advise the patients to walk.

R: The intervention we are testing suggests that Health Extension Workers should detectlymphatic filariasis based on whether the area where the patient resides is known to be endemic for the disease the following symptoms: swelling of a lower limb, elephantiasis, swelling of scrotum/vulva, hydrocele. Do you think it is possible for Health Extension Workers to perform these responsibilities?

P01: Yes, I think they can perform these activities because it is possible to accomplish any task asssigned from anybody.

R: How would you say the intervention is different from what you normally do?

P01: What I learned from the intervention and what is different – before the training, I was not aware of the importance of elevating limbs, but now I understand how important this is. Before the training I only knew that pregnant women should elevate their legs, but I did not know that men affected by this disease should also do that. And I didn't know that wearing shoes helps LF patients.

R: In your opinion, are there any problems with tasking Health Extension Workers with detecting lymphatic filariasis as suggested by the intervention?

P01: No, I dont think they face any problems in detecting those cases, because we are conducting many activities in the community without any problems and hope we will not face any challenges.

R:In your opinion, what are the implications of performing this task in terms of time and workload?

P01: Yes, it takes time and causes workload. As I told you earlier, doing this activity adds to our workload because it takes time to detect, create awareness and report the cases.

R: How could the problems you mentioned be addressed? What alternatives or improvements would you suggest?

P01: Similar to the recommendation I proposed for trachoma -- additional manpower and staff from the health centre should be involved in case detection together with us.

R: The intervention suggests that patients with acute lymphatic filariasis should be referred to the health centre for further assessment. Patients diagnosed with chronic lymphatic filariasiswill be referred back from the health centre to the health post, where they will receive guidance on home management of the disabilities caused by the disease. Do you think it is possible for Health Extension Workers to perform these responsibilities?

P01: Yes, they can perform them, because as I mentioned earlier, we advise and teach families and victims to keep their personal hygiene and advise the family to help them, so I think we can do that.

R: How would you say the intervention is different from what you normally do?

P01: Actually, referring LF victims back to the health post is new for us. I hope we can visit patients at their home and teach families and the person affected to wash their leg regualrly, wear shoes and advise the family to help the patient.

R: In your opinion, are there any problems with tasking Health Extension Workers with managing lymphatic filariasis as suggested by the intervention?

P01: Health Extension Workers can perform any activity. I don't think they will face any problems in managing LF.

R: In your experience, are infastructure and equipment required to perform this task normally available at this health post?

P01: Actually, I haven't faced this kind of patient at the health post so far. But if these patients visit the health post, there is nothing I can do at health post level. I can only teach and create awareness about the disease. But as per the training, ointment, oil, soap, a dish for leg washing and bandages should be available at health post and household level, because these materials are essential to manage LF.

R: The intervention we are testing also assigns certain tasks to the Health Development Army. After the classroom training on neglected tropical diseases for Health Extension Workers, you were asked to inform the Health Development Army of their roles and responsibilities with regard to neglected tropical diseases in this intervention. Did you share this information with the Health Development Army?

P01: Yes, I shared the information with the Health Development Army.

R: How did you organise this?

P01: We oriented the Health Development Army each of them at their households. In our kebele, there are 52 HDA team leaders, but so far we have only reached and oriented 40.

R: Why haven't you been able to reach all HDAs?

P01: What are you saying? This work is very hard and challenging, so due to lack of time, we have not yet reached all households.

R: How could the process of sharing information about neglected tropical diseases with the Health Development Army have been improved?

P01: As I said, distributing the materials and orienting each of them sepatately was very tough work. The process of disseminating information would be simpler if it was arranged to give training at one place or venue.

R: Why was it not possible to invite all HDAs to one place and orient them?

P01: What? If I invite the HDA for orientation, I should prepare tea and coffee. In addition, they don't come to the orientation unless a certain amount of transport allowance is given to them. That's why we had to orient them at household level.

R: In your understanding, what is the role of the Health Development Army with regard to neglected tropical diseases?

P01: In my understanding, the Health Development Army can reach 1-to-5 network leaders and the people under their network and they create awareness among the women I cannot reach due to lack of time. Also, as they are from the same community, they detect and report the NTDs monthly to the health post.

R: In your experience, is this happening in the catchment area of this health post?

P01: Yes, some of the leaders are sending their monthly reports.

R: Do you ever see patients who have been encouraged to seek care at the health post by the Health Development Army?

P01: Yes, I have seen patients referred by HDA leades. For example, I treated a suspected trachoma patient with TTC oitment at the health post. Another time, there was a LF patient who was referred to me by an HDA leader, but as I cannot treat LF patients with swollen legs, I referred them to the health centre.

R: How often does this happen?

P01: Not a lot. It's mostly tachoma patients visiting our health post who were refered by HDA leaders.

R: In your experience, what challenges are the Health Development Army facing in detecting signs and symptoms of NTDs and ecouraging people to seek care?

P01: Actually, the HDA are doing fascinating things, but in my experience, one HDA leader came to me and reported that her father-in-law had a disease affecting his scrotum, "How can I teach him and refer?"

R: Why could she not teach him?

P01: You know, culturally in our community, women fear to talk with their mother and father-in-law. In addition, if the disease is around the genital area, they are afraid of talking with men. And sometimes diseases around women's womb are not easily identfied because women hide the disease fearing their secret may be revealed.

R: Do you discuss the challenges the Health Development Army are facing with regard to neglected tropical diseases during your regular interactions with the Health Development Army?

P01: As I told you earlier, some HDA leaders come to us to tell us the challenges they face in detecting NTDs. But as you know, this intervention started only a few months ago, so we have not yet started discussing the challenges in our regular meetings.

R: What do you generally think of the idea of tasking the Health Development Army with detecting people with signs and symptoms of neglected tropical diseases? Do you think the Health Development Army has the capacity to take on these roles and responsibilities?

P01: I don't think HDA leaders have the capacity to detect the cases exactly.

R: Why?

P01: As you know, the orientation we gave them was not very deep. Also the topic was covered in a short period of time and can be understood differently among the HDA leaders, because their level of understanding can be different. Some can understand easily what is taught, but some might not. To strengthen the capacity of the HDA leaders, in my opinion, you need to give intensive training gathering them all at one venue – the way we were trained.

R: Do you think there are any challenges with regard to gender? Would women and men face different challenges in interacting with the Health Development Army about NTDs?

P01: Yes, as I told you, HDAs may find it challenging to teach, advise and refer men who have problems around the genital area and sometimes even females may hide their problems from HDA leaders.

R: Do you think there would be any challenges with regard to stigma? Would those affected by disabilities caused by NTDs be detected by the Health Development Army?

P01: Yes, HDA can detect people affected by NTDs, because I don't think the community marginalises or stigmatises NTD patients. But often there may be people who stigmatise.

R: What could be done to address those challenges?

P01: In my opinion, to avoid stigmatisation, you need to work on awareness creation among the wider community.

R: Thank you very much.

HEW

R: In May this year, classroom training was offered to Health Extension Workers from your health post about neglected tropical diseases. Did you attend this training?

P02: Yes, I attended the training.

R: Can you tell me what you learned in this training? What did the training focus on?

P02: The training focused on diseases that are neglected by the government and the community. Those diseses affect the community a lot. The community has been attacked by those diseases, but the government and the community did not pay much attention and the community did not receive treatment because the diseases attack slowly. But now the government gives attention to those diseases with us to create awareness among the community through the Health Development Army that there is treatment for those five diseases and to seek medical treatment.

R: Do you remember which diseases this training covered?

P02: Trachoma, soil-transmitted worms, schistosomiasis, communicable leg swelling, non-communicable leg swelling and so on. We were trained on those disease in this project.

R: What did you like about the training

P02: What I liked about the training is that I learned about diseases that need attention. These diseases affect the community even though we did not pay attention. While there are treatments for those diseases, we heard that the community came to us and said, "I have abdominal cramps" and so on, but there was a big problem because we did not give those

diseases attention. For me, what I like the most is that I now know about those neglected diseases and I am ready to teach the community.

R: Was there anything you didn't like about the training?

P02: What I didn't like was – we were two Health Extension Workers and there were also health workers. The health workers work at the health centre, but as for us, Health Extension Workers have so many duties and we were also expected to distribute those training materials and educate the community by visiting their homes. Even if it's not really difficult to distribute to the Health Development Army, we were tasked with distributing to the 1-to-5 networks. There are too many of them and we were expected to collect forms, train and distribute materials to all those 1-to-5 networks. This is difficult because of the workload we have at the health post.

R: So this programme caused additional workload?

P02: Yes.

R. How could the training have been improved?

P02: There was nothing to improve. The training was good.

R: Did you think the training was relevant for your job? Did you benefit from it?

P02: Yes, I benefited from the training.

R: How?

P02: The benefit I got was actually what we leaned at school, but we expected further training. Currently we work by linkjng our work with what we learned in the training.

R: After the training, what is your understanding of your role with regard to detecting and managing NTDs?

P02: In the community, when they have such problems, I try to provide a solution by educating about those diseases that used to be neglected.

R: For example, what can you do about those disease after this training?

P02: For example, if it is trachoma, I now know that there are acute and chronic cases. Those acute cases are simple to manage and the chronic cases need surgery. Lymphatic filariasis attacks slowly and the treatment has a very slow effect. And also I know about the treatment

for scrotal swelling and breast swelling. For scrotal swelling there is surgery, but for breast swelling there isn't.

R: What is your overall role in conrolling these diseases?

P02: We are working on the prevention of those diseases.

R: So what do you do after this training?

P02: We educate the community to keep personal hygiene. For trachoma, to prevent breeding of flies in the environment and to always wash their faces and hands with soap. And for those intestinal parasites, to have a toilet and to use it and wash their hands after using the toilet.

R: Do you do anything other than educating?

P02: As for the treatment, we tend to refer them to the next level, but in case individuals refuse to go to the referral facility, I will give albendazole as treatment and I'll refer them for follow-up if the cases become severe. Then we push them to get treatment from a health facility. If it is schistosomiasis, I can give praziquantel. And we also do a campaign for intestinal parasites once a year for men and women over five.

R: Were you given any materials during or after the training to help you remember what was taught and to help you detect and manage NTDs?

P02: Yes, we receive some materials.

R: Did you receive those materials?

P02: Yes, we received a manual for treatment of patients over five years to help us decide if we can treat. And if it is beyond our capacity, we refer to the next level. Previously, we worked on treating intestinal parasites and we referred for further investigation. Now, by using this job aid and the family folder, we treat and refer cases. It helps us manage our work properly. Our responsibility is mainly to refer, but if they refuse to go, we can give albendazole for intestinal parasites.

R: Do you use the job aid?

P02: Yes, actually we used to work on under-fives using a chart booklet, but for this job aid, they put a symbol to identify NTDs. Previously, we did not focus on over-fives, but now this job aid helps us to see these symptoms in patients over five.

R: What do you like and dislike about the job aids?

P02: We gain knowlegde about those neglected diseases and how to identify them, even if we are not treating them. That's what I like about the job aids. In the past, we provided albendazole during campaigns, but now we are treating those children under five and people over five who clearly have abdominal problems, except pregnant women in the first trimester. We knew how important deworming campaigns were, but now we clearly know when and why we deliver those drugs. There is nothing I dislike about the job aid.

R: How could the job aids be improved?

P02: There is nothing to be improved. I am comfortable with them.

R: The training and the materials that were distributed provide suggestions for how Health Extension Workers should detect and manage six diseases: trachoma, urinary schistosomiasis, intestinal schistosomiasis, soil-transmitted helminth infection, lymphatic filariasis and podoconiosis.Do you ever see patients with signs and symptoms of any of the diseases?

P02: Yes, I have seen trachoma, intestinal parasites that cause addominal bloating and diarrhoea. There is also a person in my village who has lymphatic filariasis. And there is a person who had a swollen scrotum and needed to be referred to the hospital for surgery with the help of – I think it was a Malaria Consortium project – and he became healthy. Some people also have non-communicable leg swelling, but they've not received any help until now. Among the neglected diseases I have not yet seen is schistosomiasis. Usually the health workers finds that by linking it with intestinal parasites.

R: Most diseases do you see most commonly?

P02: Most of the time, people come to us saying they have abdominal cramps, diarhoea and so on. Thanks to God's help, the numbers are now decreasing. Most of the time, we see those who have abdominal problems such as abdominal cramp, diarhoea and vomiting.

R: Is there a trachoma case in the community?

P02: Yes, there are many trachoma cases at the moment.

R: How often does this happen?

P02: At the moment, there are many cases in the community, especially after the training for the Health Development Army who are referring many cases with signs and symptoms to us.

R: Why do you think this is the case?

P02: I think this happened after the Health Development Army were trained and created awarness about the disease.

R: Do you know how Health Extension Workers should detect trachoma?

P02: Yes.

R: What signs and symptoms would you look out for?

P02: Broken eye lashes, itching, fear of light, swelling, discharge from the eye and changing eye colour. Those are the signs and symptoms of trachoma.

R: If you suspect a patient might have trachoma, what would you do?

P02: My job regarding trachoma is just to refer. I advise them to wash their hands and face with water and soap.

R: What do you do if such cases come to you?

P02: If the cases are mild, I try to treat with TTC **[tetracyclene]** eye ointment. We giveTTC to children as well as adults, but children respond to the treatment faster than adults.

R: What else?

P02: We give vitamin A to 9-month-old children.

R: Would you provide any treatment?

P02: Yes, I provide TTC eye oitment.

R: For whom?

P02: For the children and for adults as well.

R: Under what circumstances would you refer the patient?

P02: When the trachoma cases are stage 2.

R: When do we say "stage 2"?

P02: Fearing of light, when the colour of the eye changes – generally, we are expected to refer all trachoma cases.

R: Where would you refer the patient to?

P02: To the health centre after the Health Development Army have referred them to me. Based on my knowlegde, I will advise those patients to wash their faces regularly and then my job is just to refer them to the health centre.

R: Would you recommend any kind of follow-up?

P02: Yes, I will follow as usual after the patient has received treatment at the health centre. Since the case was referred by me, I have the responsibilty to follow up on those patients.

R: The intervention we are testing suggests that Health Extension Workers should detect trachoma based on the following symptoms: irritated red eye, mucopurulant discharge or inverted eye lashes. Do you think it is possible for Health Extension Worker to perform these responsibilities?

P02: Yes we can, but only if we attend training. We can manage first-stage trachoma cases if they give us the responsibility to do so, especially those cases that come seeking treatment from us.

R: Which symptoms are treated by you?

P02: I can only treat eye irritation. That's the symptom Health Extension Workers can deal with.

R: How would you say the intervention is different from what you normally do?

P02: Now, after the training, we are identfy the signs and symptoms of trachoma.

R: In your opinion, are there any problems with tasking Health Extension with detecting trachoma as suggested by the intervention?

P02: No, there are no problems in identifying such cases. I think as trained Health Extension Workers, it should not be a big deal to identify the signs and symptoms of trachoma.

R: In your opinion, what are the implications of performing this task in terms of time and workload?

P02: As you know, when the work increases, the workload increases. But there aren't many of those cases and so it does not take up much time. But if the numbers increase, it will take more time.

R: The intervention suggests that patients with signs and symptoms of trachoma should be referred to the health centre for further assessment. Do you think people will follow your advice?

P02: Yes they agree with our recommendation. If we write a referral, they accept it. If we refer to a respected health facility, they accept and go there. No one refuses.

R: Why is that?

P02: Because we advise to follow the referral. If the cases are not treated early, it can cause a lot of problems. It can even cause blindness.

R: They trust you?

P02: Yes they trust us and accept what we say.

R: What could be done to ensure people with signs and symptoms of trachoma receive good care?

P02: Sometimes, when people go to the health centre for the services, they say that they did not receive attention.

R: Why is that?

P02: The health workers did not give them the treatment they needed when they went to see them. Nowadays, people form an opinion on the health worker's knowledge. After taking medicine, they come to us showing us the drug they received at the health centre. Sometimes they say they received this drug without laboratory investigation.

R: Do you believe that the health centre is providing good services for trachoma?

P02: No, I don't. They may not have drugs at the health centre. There's a lady who had trachoma and we referred her to the health centre who then referred her to **[name of district hospital]**. She is feeling better, but the she is not healed.

R: Do you think that at health centre level trachoma can be treated?

P02: No, I think it is not possible for the health centre here to treat trachoma.

R. Which other diseases do you see?

P02: I've seen soil-transmitted helminths.

R: What are the signs and symptoms of soil-transmitted helminth infection?

P02: Vomiting of worms through nose and mouth. The finger nails become disfigured. Children or adults become thin, which can be clearly observed. Yellowish discolouration of the eye, diarrhea with blood. The anus may be inverted and they have vomiting and nausea in response to everything -- loss of appetite.

R: What do you do if you see such cases?

P02: When I see such cases -- when some of my community members refused to go to another health facility, we tried to treat with albendazole and ORS **[oral rehydration solution]**. *After the treatment, so many people came and said they feel better now.*

R: What do you do for those who have signs and symptoms?

P02: After the training, I just refer the cases to health centre. But if they refuse to go to the health centre, we give them albendazole. My main job in this case is just referring.

R: Do you give albendazole to both children and adults?

P02: Actually we are treating diarrhoea in children. We had a manual for treatment of diarrhoea.

R: Is it the current job aid?

P02: No, the previous IMNCI guideline. We treat diarrhoea without dehydration -- plan A and plan B and also provide zinc.

R: Is it the same management for children and adults who have diarrhoea?

P02: No, I refer children if they have diarrhoea. According to the training, I am expected to refer the cases to the next health centre because the person should be seen by a laboratory

R: Do you follow up with those patients?

P02: Yes, we follow up. For example, if the case is malaria, we make an appointment after two days. If it is pneumonia, two days. But with these cases, I don't know how to follow up cases, because the place where the treatment is given is different from the place where the follow-up is done. We try to follow up whether they received treatment or not.

R: You told me the worms come through the nose, diarrhoea, aneamia and weight loss. Can Health Extension workers identify those cases?

P02: Yes, according to the training we can identify them.

R: After the training, did the way you treat cases change?

P02: Yes, it did.

R: What do you do now?

P02: Previously, I treated those cases without confidence, but now I have the confidence to refer such cases with knowledge.

R: Anything else?

P02: The organisation **[Malaria Consortium]** *asked about our interest to treat at health post level if they bring the medicine for this project, but we said if the government does not clearly assign this task to us, we should not treat cases. As far as I know, we now refer cases. And thanks to the help of God, the numbers are decreasing.*

R: The intervention suggests that patients with worms coming out of their mouth/nose/anus, as well as patients up to five years with mild abdominal pain should receive albendazole. All other patients should be referred to the health centre for further assessment. Patients who state that they do not want to be referred, except those who are in the first trimester of their pregnancy and those with fever and vomit, jaundice, ascites, intestinal blockage and rectal prolapse, should be given albendazole presumptively. Do you think it is possible for Health Extension Worker to perform these responsibilities?

P02: Yes, Health Extension Workers can do that.

R: In your opinion, are there any problems with tasking Health Extension Workers with detecting soil-transmitted helminth infections as suggested by the intervention?

P02: After the training, there is nothing difficult to do for Health Extension Workers about intestinal parasites.

R: In your opinion, what are the implications of performing this task in terms of time and workload?

P02: It does not take much time.

R: Do you have the drug to treat soil-transmitted helminth infection at health post level?

P02: We have albendazole which was left over from the campaign. Our work is just referring cases.

R: The intervention we are testing also assigns certain tasks to the Health Development Army. After the classroom training on neglected tropical diseases for Health Extension Workers, you were asked to inform the Health Development Army of their roles and

responsibilities with regard to neglected tropical diseases in this intervention. Did you share this information with the Health Development Army?

P02: Yes, we did.

R: In your understanding, what is the role of the Health Development Army with regard to neglected tropical diseases?

P02: According to the training they had, they are going to identify the signs and symptoms and refer cases. And also, they tally what they saw and report to us.

R: What else?

P02: They educate the community about those neglected diseases by telling them that there is treatment for the diseases and that they are now giving those diseases attention.

R. After the training that you had, did you train the Health Development Army?

P02: Yes, we did.

R: How did you train them?

P02: As I told you, training them was difficult because they live in different areas, so we visited them house-to-house to show them the manual. We trained them in their homes, but I think the training is not enough for them.

R: Among your Health Development Army, how many did you train?

P02: We not only trained the Health Development Army, but also the 1-to-5 army. We trained all the Health Development Army and the Health Development Army leaders who are also 1-to-5 army leaders. In general, we trained the entire Health Development Army in this kebele. From among 180 1-to-5 army leaders, we trained 136.

R. How could the problems you mentioned be addressed? What alternatives or improvements would you suggest?

P02: Actually, I don't know why you delivered it in this way, but it is better to train them all in one place as you did for us and ask them to focus on the diseases in front of them. When we train them, as you know, many are mothers and too busy with their duties. We are also travelling to their homes and may forget to tell them the details about the diseases.

R: Do you ever see patients who have been encouraged to seek care at the health post by the Health Development Army? How often does this happen?

P02: Yes. They even identify cases that are above our capacity, like uterine problems or problems with the reproductive organs, vaginal bleeding, abortion and also cases of utero-vaginal prolapse. So because of what we told them, this is the kind of things they are doing.

R: Have the Health Development Army reported any cases yet?

P02: To tell you the truth, they are expected to send report to us, but so far, they are not reporting. No one has come to us with reports. They ask us if we have medicine at the health post and when we reply that the treatment is available at the health centre, they may send them to the health centre, bypassing us. Sometimes patients with abdominal problems come to us. But we don't see trachoma cases.

R: Why do you think that is?

P02: I suspect they send them to the health centre. Some of the patients say they tried a lot of things to cure their problems, but things did not improve, so they want no more hassle. So for that reason the community may refuse their services and go to the health facility with their problems. If it is possible and there is time, it would be better to train them together with us. I faced a challenge when we trained the Health Development Army and distributed the materials and forms as well, I asked them to please write on disease on the report but still she brought back the whole document as it was, even though she is educated.

R: Why?

P02: Because she did not understand what the form is used for. We train them as well as you could train them, but some of them did not understand what they learnt. There is a knowledge gap and they do not understand easily about those neglected diseases. The Health Development Army need to be trained occasionally to understand those diseases.

R: In your experience, what challenges are the Health Development Army facing in detecting signs and symptoms of NTDs and ecouraging people to seek care?

P02: Yes, there are problems. She may face an individual with a swollen scrotum but he may not accept her advice. He may be the Health Development Army member's father-in-law and she may try to tell him in different ways, but he may not completely accept her advice.

R: Do you discuss the challenges the Health Development Army are facing with regard to neglected tropical diseases during your regular interactions with the Health Development Army?

P02: Yes. As you know, different Health Development Army members have different levels of understanding and education, so they may face different challenges. We try to discuss with them according to the training they've had.

R: What challenges do you expect?

P02: There may be different problems relating to the sex organs, such as vaginal bleeding and itching due to sexually transmitted diseases. If they do not have enough knowledge, they may think it is a neglected tropical disease. Those who have utero-vaginal diseases, they are not categorised as negleted tropical diseases. So there's a lack of knowledge to clearly identify signs and symptoms.

R: Anything else?

P02: It is not so important to tell everything.

R: Can you tell me more?

P02: They said, "You are working for your own benefit. You receive a payment for your visit." They did not consider that they benefit from the training. The Health Development Army do not get incentives from the government as they are delivering a free service.

R.What do you generally think of the idea of tasking the Health Development Army with detecting people with signs and symptoms of neglected tropical diseases?

P02: I think the Health Development Army can detect people with signs and symptoms of neglected tropical diseases, but they may face a challenge with identifying cases of scrotal swelling. But they haven't reported those diseases and they are not committed to reporting. The reports they submit are incomplete, for example age, type of problem.

R: Do you think the Health Development Army has the capacity to take on these roles and responsibilities?

P02: Yes, they can, except for the negligence and lack of understanding of some Health Development Army members about neglected tropical diseases.

R: What kind of challenges do you foresee?

P02: As I told you, they may face challenges when asking about scrotal swelling since they are female. There is a case of an old man with a scrotal swelling. Training them again would be a solution to the problem.

R: Do you think there are any challenges with regard to gender?

P02: Yes, there is a problem in asking and educating males about scrotal swelling when Health Development Army members are all female. Another challeng is that the community may come to us and ask that for their benefit, they should get paid.

R: How could you adress such challenges?

P02: It is better to adress cases of scrotal swelling through their wives by telling them about the causes and its treatment.

R: Do you think there will be any challenges with regard to stigma?

P02: Yes. There's a patient who has a swollen leg. I think it's communicable. She does not leave her home. We reported the case, but still there was no solution for her problem. We provided a drug during a campaign, but her condition is getting worse. She does not go out. She lives with her daughter.

R: Is that because of stigma?

P02: I think she may experience stigma if she went out.

R.What could be done to address those challenges?

P02: Educating the community about neglected diseases.

R: What do you generally think of the idea of tasking health workers at primary health care level (including health centres and hospitals) with detecting, managing, recording and reporting neglected tropical diseases?

P02: As I told you, the Health Development Army send people to us and we are expected to refer them to the health centre. At the health centre, if they can, they manage the cases themselves or they refer them to the hospital for better treatment.

R: Do you think the primary health care system has the capacity to take on these roles and responsibilities?

P02: Yes, they do. At the health centre, they can manage what they can at their level before referring to the next level. The government has the power to do that.

R: What kind of challenges do you foresee?

P02: People may face lack of treatment when they go to the health facility and that will disappoint them.

R: Is there anything else you would like to share with the research team?

P02: I have nothing to add, but we need follow-up and further training on those neglected diseases.
